# *De novo* transcriptomes of six calanoid copepods (Crustacea): a resource for the discovery of novel genes

**DOI:** 10.1038/s41597-023-02130-1

**Published:** 2023-04-27

**Authors:** Daniel K. Hartline, Matthew C. Cieslak, Ann M. Castelfranco, Brandon Lieberman, Vittoria Roncalli, Petra H. Lenz

**Affiliations:** 1 Pacific Biosciences Research Center, University of Hawai’i at Mānoa, 1993 East-West Rd., Honolulu, HI 96822 USA; 2 Integrative Marine Ecology Department, Stazione Zoologica Anton Dohrn, Naples, Italy

**Keywords:** Molecular ecology, Transcriptomics

## Abstract

This study presents eight new high-quality *de novo* transcriptomes from six co-occurring species of calanoid copepods, the first published for *Neocalanus plumchrus*, *N. cristatus, Eucalanus bungii* and *Metridia pacifica* and additional ones for *N. flemingeri* and *Calanus marshallae*. They are ecologically-important members of sub-arctic North Pacific marine zooplankton communities. ‘Omics data for this diverse and numerous taxonomic group are sparse and difficult to obtain. Total RNA from single individuals was used to construct gene libraries that were sequenced on an Illumina Next-Seq platform. Quality filtered reads were assembled with Trinity software and validated using multiple criteria. The study’s primary purpose is to provide a resource for gene expression studies. The integrated database can be used for quantitative inter- and intra-species comparisons of gene expression patterns across biological processes. An example of an additional use is provided for discovering novel and evolutionarily-significant proteins within the Calanoida. A workflow was designed to find and characterize unannotated transcripts with homologies across *de novo* assemblies that have also been shown to be eco-responsive.

## Background & Summary

Transcriptomics, the application of high-throughput sequencing of millions of short nucleotide sequences generated from mRNA, has transformed studies of the physiological ecology of marine organisms^1–5^. Gene expression profiles of one or more species have resulted in new insights into physiological acclimatization, metabolic trade-offs and resilience, causes of high-mortality events and niche partitioning between similar species. However, progress in the application of environmental transcriptomics has been hampered by a scarcity of genomic resources, especially for co-occurring zooplankton species. Furthermore, existing references for gene expression studies are of variable quality and many genes, including eco-responsive genes remain unannotated^5^. Here, we present eight *de novo* transcriptomes for six calanoid copepod species that co-occur across the Sub-arctic North Pacific. The goals were to (1) generate high-quality assemblies, (2) assess their value as references for cross-species comparisons; and (3) to develop a workflow to search for and identify novel predicted proteins that are unique to copepods.

Calanoida is an order within the Copepoda, the second largest sub-class of the Crustacea. Calanoids are small (≤15 mm) free-living, mostly planktonic organisms that inhabit marine and freshwaters worldwide^6,7^. Abundant and ecologically important, they are a key link between phytoplankton and the upper trophic levels, such as larval fishes and other planktivores including invertebrates, sea birds and marine mammals^7^. Relative to their importance they are understudied: their natural habitats are difficult to access and few species are amenable to laboratory cultivation. Nevertheless, calanoids are known for many unique adaptations (high transparency, fluorescent proteins, bioluminescence, myelin, unique sensory structures) that have set them apart from other better studied arthropods^8–12^. These cellular properties depend on the evolution of novel proteins. Thus, it is not surprising that *de novo* assemblies of copepods always include a significant number of predicted proteins that are unannotated with unknown function^13–15^. A significant number of these are differentially-expressed eco-responsive genes^3,16^, yet no studies have used available copepod transcriptomes to search for taxon-specific proteins with unknown function.

In contrast to terrestrial arthropods, genomes of only a few marine copepods have been fully sequenced, assembled and annotated^17–20^. However, *de novo* assemblies of expressed transcripts of non-model organisms provide an alternative solution to generate reference transcriptomes for gene expression studies and the discovery of novel proteins. The six species in this study are important members of sub-arctic pelagic communities, inhabiting both oceanic regions and coastal sounds and fjords in the North Pacific and in the Bering Sea. The species account for nearly 80% of the annual calanoid copepod production in the region^21^. Climatic events like heat waves and global warming have raised concerns about the stability of the zooplankton community. How the community might change requires a better understanding of the ecophysiology of all six species across a range of environmental conditions.

The six species in this study present a taxonomic range that increases opportunities for finding new proteins, both shared or group-specific. They include three species in the genus *Neocalanus* (*N. flemingeri*, *N. plumchrus*, *N. cristatus*), one additional member of the family Calanidae (*Calanus marshallae*), one member of the non-calanid myelinate calanoids (“Myelinata”; the eucalanid *Eucalanus bungii*) and a more basal and amyelinate species, *Metridia pacifica* (Table 1). Taxonomically, the species belong to three calanoid superfamilies, with different adaptations (Table 1)^7,22–24^. The four species in the family Calanidae belong to a group of lipid-rich copepods that are a key food source for upper trophic levels. Their complex life cycle includes a seasonal developmental arrest and dormancy (“diapause”) at depths of 200 m or deeper^25^. The amyelinate *M. pacifica* is known for its significant diel vertical migration and bioluminescence^26^. The sixth species, *E. bungii*, in addition to being myelinate, is characterized by its transparency and very long first antennae. Behaviourally *E. bungii* hovers in place generating feeding currents to bring food particles to its mouth^27^.

**Table 1:** Summary of Alaskan calanoid species and collection details used to generate the RNA-Seq data.

Species	Superfamily & Family	Myelin Y/N	Stage	Collection date, station & depth	Abbreviation	NCBI TSA#
*Neocalanus flemingeri*	Megacalanoidea^1^ Calanidae	Y	Pre-adult (CV)	9/23/2018, PWS3 400–600 m	Nf2018	GJSD00000000
				4/30/2019, PWS2 0–100 m	Nf2019	GJRT00000000
*Neocalanus plumchrus*	Megacalanoidea Calanidae	Y	Adult male (CVI)	9/21/2015, PWS2 500–700 m	*Np2015	GJRU00000000
*Neocalanus cristatus*	Megacalanoidea Calanidae	Y	Pre-adult (CV)	9/21/2017, PWS2 400–600 m	*Nc2017	GJRH00000000
*Calanus marshallae*	Megacalanoidea Calanidae	Y	Pre-adult (CV)	9/23/2018, PWS3 400–600 m	*Cm2018	GJRL00000000
				9/20/2017, PWS2 0–100 m	Cm2017	GJRF00000000
*Eucalanus bungii*	Eucalanoidea Eucalanidae	Y	Pre-adult (CV)	9/20/2017, PWS2 400–600 m	*Eb2017	GJRG00000000
*Metridia pacifica*	Augaptiloidea^2^ Metridinidae	N	Adult female (CVI)	9/20/2017, PWS2 400–600 m	*Mp2017	GJAO00000000

Taxonomic classification of species and superfamily designation according to Andronov (1974)^33^. State of myelination according to Davis *et al*. (1999) and Lenz & Hartline (2017)^9,10^.

^1^Megacalanoidea = Calanoidea

^2^Augaptiloidea = Arietelloidea

*Transcriptomes included in the 6-Calanoida OrthoVenn2 uploads (also included in the uploads is the *N. flemingeri* reference transcriptome “Nf_ref,” TSA series GHLB01000000; see Methods).

## Methods

### Work-flow

**Fig. 1:**
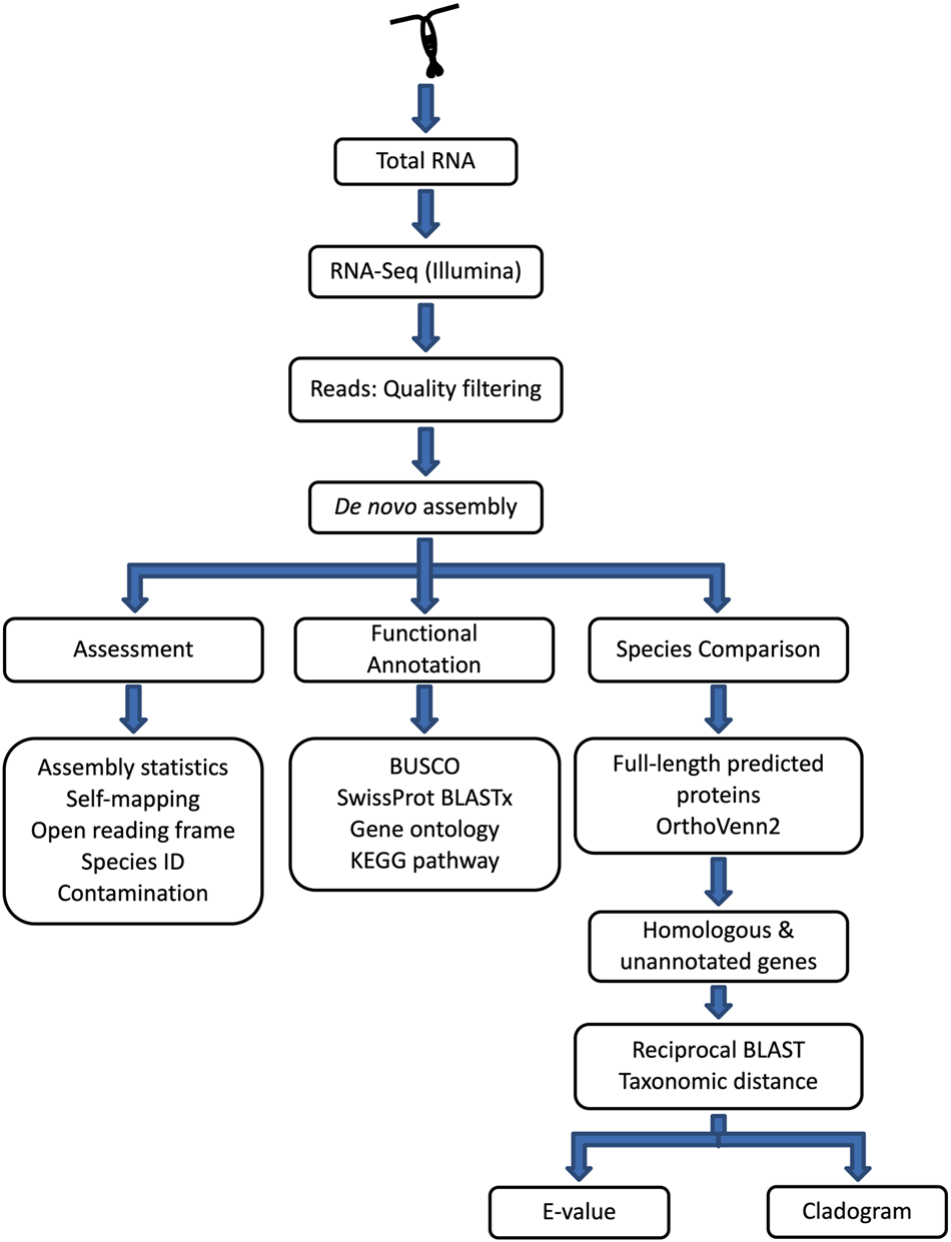
Flow chart of the processing of individual copepods for RNA-seq and data analyses. After processing short sequence reads (150 bp, paired-end) for quality and Trinity assembly, *de novo* transcriptomes were assessed for quality (left), annotated (center) and compared across species (right). The flow on the right branch focuses on the analysis of full-length predicted proteins and includes the identification of orthologous genes and the search for novel proteins.

A diagram of the workflow used in this study is presented in Fig. 1. *De novo* transcriptomes were assembled from short-read sequences generated from RNA extracted from field-collected individuals. Each assembly was assessed for quality using multiple indicators, and assembled transcripts were annotated based on sequence similarity. Cross-species comparisons focused on identifying annotated and unannotated orthologous genes among the six species. In downstream analysis, selected unannotated genes were examined for their eco-responsiveness, their presence as orthologs across taxonomic groups and functional motifs in their predicted proteins.

### Sample collection

Zooplankton were collected from depth (2015, 2017, 2018, and 2019) during the cruises of the Seward Line Long-term Observation Program (LTOP) (http://www.sfos.uaf.edu/sewardline/) at two stations in Prince William Sound: “PWS2” (Lat: 60°32.1′ N, Long: 147°48.2′ W, depth 798 m) and “PWS3” (Lat: 60°40.0′ N, Long: 147°40.0′ W, depth 742 m). Table 1 lists the collection date, station and depth stratum for each individual. Zooplankton collections were made using either a QuadNet with two 150 µm and two 53 µm mesh nets (April collection), or a multiple opening and closing plankton net (0.25 m^2^ cross-sectional area; 150 μm mesh nets; Multinet-Midi, Hydro-Bios; September collections). The QuadNet net was towed vertically from 100 to 0 m, while the Multinet was towed either vertically or obliquely sampling depth strata between 700 m and the surface. The live zooplankton collections were immediately diluted into ambient seawater and maintained at collection temperatures (5 – 7 °C). Copepods were removed from the diluted samples using a small ladle, and sorted under a dissection microscope to select individuals from the target species (Table 1). Briefly, live and undamaged individuals were identified and staged using morphological criteria, with species identification being confirmed through the COI sequence in the assembled transcriptomes. Identified copepods were isolated using a wide-bore pipette into a dish with filtered seawater before transferring them into microcentrifuge tubes (1.6 ml) with 0.5 ml of RNAlater Stabilization Reagent (QIAGEN). Preserved copepods were frozen first in −20 °C during the cruises, and then transferred to −80 °C until further processing.

### Total RNA extraction, library construction, RNA sequencing and quality control

For each target species, total RNA was extracted from individuals using QIAGEN RNeasy Plus Mini Kit (catalog # 74134) in combination with a Qiashredder column (catalog # 79654). Selection for sequencing was based on high RNA yields and purity of extraction (RIN >8). One individual was selected from each species, except for two where two individuals were sequenced from the same species. The final list included pre-adults (CV) for *Neocalanus flemingeri* (n = 2), *N. cristatus* (n = 1)*, C. marshallae* (n = 2), *E. bungii* (n = 1), an adult male (developmental stage CVI) for *N. plumchrus* (n = 1) and an adult female for *M. pacifica* (n = 1). Total RNA was shipped on dry ice to the Georgia Genomics Bioinformatics Core (https://dna.uga.edu) for RNA-Seq. There, double-stranded cDNA libraries (KAPA Stranded mRNA-Seq Kit, with KAPA mRNA Capture Beads (cat KK8421) from each individual were multiplexed and sequenced using an Illumina Next-Seq. 500 instrument (High-Output Flow Cell, 150 bp, paired end). Quality of each RNA-Seq library was reviewed with the FastQC software^28^. From each RNA-Seq library, low quality reads were removed using FASTQ Toolkit (v. 2.2.5 within BaseSpace). Illumina adaptors, reads < 50 bp long, reads with an average Phred score < 30 and the first 12 bp from each read, were removed from each library. The same workflow was applied to all eight datasets.

### *De novo* assembly, mapping, core-gene statistics

Individual *de novo* transcriptomes were generated from each dataset at the National Center for Genome Analysis Support’s (NCGAS; Indiana University, Bloomington, IN, USA) Mason Linux cluster using Trinity software (v. 2.4.0, except *N. plumchrus*, v. 2.0.6)^29^. Initial evaluation involved self-mapping of reads against the respective *de novo* assembly using Bowtie2 software (v. 2.3.5.1)^30^. Completeness of each *de novo* assembly was evaluated using Benchmarking Universal Single-Copy Orthologs (*BUSCO*) software^31^ by searching each assembly for the presence of eukaryote “core” genes using the Arthropoda database as reference (BUSCO version 5.3.2, dataset: arthropoda_odb10 (2020-09-10, 90 genomes, 1,013 BUSCOs).

### Functional annotation

Assemblies were functionally annotated against the NCBI Swiss-Prot protein and UniProt databases. Initial annotations were obtained by using the BLASTx algorithm on a local BLAST webserver with a Beowulf cluster using the Swiss-Prot protein database (downloaded February 2021) as reference and a threshold E-value of 10^−5^. Transcripts with BLAST annotations were then searched against the Gene Ontology (GO) and the Kyoto Encyclopedia of Genes and Genomes (KEGG) pathway databases using UniProt^32^.

### Species confirmation and contamination testing

The annotation results were also used to confirm species identity and to provide an initial assessment of possible contaminants. Sequences annotated as “COX1” (cytochrome oxidase subunit 1 or “COI”) were retrieved from each file, followed by a confirmatory BLASTn into the NCBI nucleotide collection (nr/nt) database. BLAST results for each top hit were checked for a species match, transcript coverage, E-value and percent identity (>97% identity required). To search further for fragments of foreign COI sequences, an artificial “species filter” that quantifies such contaminants^33^, was constructed from the full-length COI sequences of each of the species presented here, as well as likely contaminants (See Supplementary Information SD1, Part II). Raw reads from each transcriptome were mapped against the artificial one, and the counts attracted to the COI of each species were used as a measure of contamination level. In addition, each assembly was searched manually for ribosomal RNA sequences. The BLASTn algorithm was run using species-specific 18 S rRNA sequences as queries to: (1) obtain the native sequence(s) most closely matching the rRNA reference; and (2) to search for rRNA sequences indicating possible foreign contamination. MAFFT (v. 7.511) alignments were used to identify sequences with identities in the low 90% range or below (“% ID to native” column in Supplementary Information SD2) as candidates for foreign rRNA. Such candidates were then checked for taxon identification and magnitude of contamination using a reciprocal BLAST (BLASTn) and relative abundance in the count file generated by Bowtie2.

### Protein prediction

The assembled transcriptomes were further validated by tallying the numbers of predicted proteins they contained and the homologies among them, both within and between species. For each assembly, open reading frames (ORFs) were identified using TransDecoder (v. 5.5.0; setting: report only the single best ORF)^29^. The resulting translated transcriptomes were filtered for predicted full-length (“complete”) proteins.

### Homolog clustering

Prevalence of homologs, especially among closely-related species, was used as a further validation of transcriptome quality. Groups of predicted proteins with homologies among selected combinations of the new transcriptomes were generated using OrthoVenn2, a web-based tool designed to find sets of homologous proteins, termed “clusters”^34^. When homology clusters needed a common reference, we included a vetted transcriptome previously-published for a stage CV *N. flemingeri* (NCBI accession series GHLB01000000, BioProject PRJNA496596)^3^. To estimate the reproducibility of separate assemblies of the same species, we generated two intraspecies cluster sets, one for *N. flemingeri* (n = 3) and one for *C. marshallae* (n = 2). To estimate the extent of homologies between transcriptomes of more distantly-related species, we generated heterospecific cluster sets for each of the assemblies paired with the *N. flemingeri* reference transcriptome. Finally, to survey the consistency of homologies across different phylogenetic breadths in these transcriptomes, we generated cluster sets from multi-species selections of transcriptomes representing four taxonomic categories: within genus (n = 3, *Neocalanus*), within family (n = 4, Calanidae: *Neocalanus* species plus *C. marshallae*), within the Myelinata (n = 5, Calanidae + *E. bungii*), and within order (n = 6, Calanoida: Myelinata + *M. pacifica*).

### Bioprospecting for novel proteins

To illustrate the potential for these transcriptomes to be useful in discovering novel proteins responsive to changes in environmental conditions, we focused on multi-species clusters of homologs for which OrthoVenn2 failed to find annotations in SwissProt. Starting with the clusters in the 6-species Calanoida set just described, which we will refer to as the “primary set,” we selected three secondary subsets of clusters having the three taxonomic coverages just described: *Neocalanus*, Calanidae, and Myelinata. For each of these subsets, we retrieved from OrthoVenn2 a “clusters shared by all” list - a list of homologous clusters containing representation from all of the selected species (see Supplementary Information SF1 for examples). While these subsets contained all clusters in the primary set with homologs (orthologs) shared among the targeted secondary species, some of the clusters also included homologs from outside of the targeted taxa. To restrict the list of candidate clusters to a taxonomically homogeneous set that lacked such “foreign” homologs, we searched the lists and removed any such outsiders; i.e. from the Myelinata subset we removed clusters that contained homologs in the amyelinate *M. pacifica* transcriptome; from the Calanidae subset, *M. pacifica* and *E. bungii*-containing clusters; and from the *Neocalanus* subset, *M. pacifica*, *E. bungii* and *C. marshallae* clusters.

The resulting lists in each of the three taxonomic categories, which are enriched for potential novel and taxonomically-unique proteins, were then further refined by searching for environmentally-regulated proteins. Each list was compared with lists of translated non-annotated *N. flemingeri* genes that had been identified in previous studies as differentially expressed in response to environmental conditions^3^. To pinpoint where these candidate novel proteins might have evolved, we put each through a tBLASTn taxonomic scan of transcriptomes in the NCBI TSA databases, searching for similar sequences in transcriptomes in taxonomic categories with increasing phylogenetic distance from *N. flemingeri*. Two measures were employed to refine the assessment of novelty: first, the E-value of the best match (top hit) for each search was used as a measure of similarity for that target taxonomic category. Novel proteins were expected to have a high similarity among those taxa possessing them and a much lower similarity for taxa lacking them. Thus, a sharp rise in the similarity (i.e. decrease in E-value) with taxonomic proximity to *N. flemingeri* was taken as a likely phylogenetic point of emergence for the novel protein. Second, this predicted emergence-point was referenced to a cladogram constructed from the top hits, translated into proteins. The translated proteins were aligned using MAFFT software^35,36^ and then manually trimmed to remove non-conserved regions. The cladogram was constructed from these sequences with the software package for phylogenetic analysis using Bayesian Markov chain Monte Carlo methods, MrBayes 3.2^37–39^, with two independent runs of four chains each and 10,000,000 generations (the initial 25% discarded as burn-in) using the WAG substitution model of protein evolution^40^ and a gamma distribution of rates. Maximum likelihood bootstrap values were obtained using RAxML 8 with 1,000 bootstrap replicates using the WAG substitution model and a gamma distribution of rates^41^. The consensus tree from MrBayes was visualized in FigTree v. 1.4.4 (http://tree.bio.ed.ac.uk/software/figtree/) and the bootstrap values were reported. The cladograms were compared with the known phylogenetic relationships among the component species. The preferred outgroup(s) for each cladogram was taken from the crustacean category (excluding Copepoda) or from the Arthropoda (excluding Crustacea). If neither category produced a satisfactory outgroup, one from a greater taxonomic distance was used. Selected sequences were examined for function-related motifs as a step toward determining possible roles for the non-annotated proteins. Motif searches were performed *via* three web-based search portals: UCL’s PSIPRED and MEMSAT (http://bioinf.cs.ucl.ac.uk/psipred/); Kyoto University Bioinformatics Center’s MotifSearch (https://www.genome.jp/tools/motif/), and Swiss Institute of Bioinformatics’ MotifScan (https://myhits.sib.swiss/cgi-bin/motif_scan).

## Data Records

The raw full-length data (Table 1) were deposited in the NCBI Sequence Read Archive https://identifiers.org/ncbi/insdc.sra:SRP289633 (2020)^42^. The respective TSA accession numbers are *N. plumchrus* (GJRU00000000), *N. flemingeri* (GJRT01000000, GJSD01000000), *N. cristatus* (GJRH00000000), *E. bungii* (GJRG00000000), *M. pacifica* (GJAO00000000), *C. marshallae* (GJRL00000000, GJRF00000000)^43–50^. Additional metadata and functional annotations of each transcriptome assembly are available at BCO-DMO (Project: 720280)^51^.

## Technical Validation

*Quality control*, de novo *assembly, mapping and core gene statistic:* Sequencing yields, assembly statistics and transcriptome completeness for the eight datasets are summarized in Table 2. The number of cleaned reads retained for the assembly exceeded 90% and ranged from 45 to 73 million (Table 2). Seven transcriptomes had N50 values that exceeded 1,000 bp. The longest transcript in all seven assemblies was at least 13,000 bp with transcripts >20,000 bp in *C. marshallae, N. cristatus* and *N. plumchrus*. The number of Trinity transcripts ranged from 66,838 to 119,923 (Table 2). While seven assemblies were very similar, the statistics for *E. bungii* suggests that the reads from this species did not assemble as well as the others: there were fewer transcripts and transcript lengths were shorter (N25, N50, N75, maximum length, see Table 2). Self-mapping rate for the assemblies averaged 89% and ranged from 84% to 92% (Table 2). Based on the BUSCO analysis, the number of “complete” orthologous BUSCO “core” genes ranged from 70% to 94%, with only one species (*E. bungii*) failing to attain at least 85% of “complete” core genes. The transcriptomes had an additional 3% to 14% “fragmented” “core” transcripts (Table 2).

**Table 2:** Summary of *de novo* assembly and annotation statistics for eight new transcriptomes.

	Neocalanus				Calanus		Eucalanus	Metridia	
	Nf2018	Nf2019	Np2015	Nc2017	Cm2018	Cm2017	Eb2017		Mp2017
***Assembly***									
Raw Reads (M)	74.7	66.6	49.2	69.3	77.9	64.4	59.8		78.4
Cleaned Reads (M)	69.7	62.8	45.2	65.4	73.1	60.4	56.1		73.8
Transcripts (#)	88,487	66,838	73,996	91,755	88,936	73,943	38,485		119,923
Trinity “genes”	41,791	35,202	-*	44,321	45,421	39,883	25,920		52,373
Min. length (bp)	301	301	301	301	301	301	301		301
Max. length (bp)	16,387	13,803	25,001	24,728	25,012	14,912	13,180		15,704
N50	1,173	1,106	1,273	1,311	1,163	1,141	960		1,426
N25	1,994	1,812	2,377	2,484	2,000	1,980	1,652		2,282
N75	674	640	656	695	663	642	542		803
GC (%)	44.7%	45.7%	44.5%	44.8%	45.0%	44.2%	47.8%		43.7%
***Self-mapping***									
Overall mapping (%)	86.6%	90.4%	88.2%	92.1%	91.0%	84.0%	85.1%		92.2%
Mapping >1 (%)	55.2%	60.8%	39.1%	55.1%	60.6%	47.1%	39.9%		66.4%
*BUSCO(n* = *1013)*									
Complete (#)	921 (91%)	872 (86%)	933 (92%)	931 (92%)	921 (91%)	860 (85%)	704 (70%)		955 (94%)
Fragmented (#)	48 (4.7%)	73 (7.2%)	37 (3.7%)	44 (4.3%)	49 (4.8%)	80 (7.9%)	143 (14%)		31 (3.1%)
Missing (#)	44 (4.4%)	68 (6.7%)	43 (4.2%)	38 (3.8%)	43 (4.3%)	73 (7.2%)	166 (16%)		27 (2.6%)
***Functional Annotation***									
Transcripts with BLAST hits (#)	40,093	32,612	33,195	40,798	40,442	35,190	17,952		52,890
Transcripts with GO terms (#)	39,318	32,009	32,539	40,022	39,728	34,570	17,659		52,002
Transcripts with EC numbers (#)	14,890	12,657	11,901	14,626	14,591	13,765	6,242		18,813
***Transdecoder***									
Transcripts with coding regions (#)	74,723	58,104	62,070	78,736	77,350	63,780	30,286		96,499
Transcripts with coding regions (%)	84.4%	86.9%	83.8%	85.8%	86.9%	86.2%	78.7%		80.5%
# complete predicted proteins	23,760	16,599	17,650	23,048	22,391	15,969	6,825		37,058
% complete predicted proteins	31.8%	28.6%	28.4%	29.3%	28.9%	25.0%	22.5%		38.4%

Columns are organized by genus and species. Abbreviation names for each transcriptome correspond to species and collection information in Table 1. *Neocalanus flemingeri* (Nf2018, Nf2019), *N. plumchrus* (Np2015), *N. cristatus* (Nc2017), *Calanus marshallae* (Cm2018, Cm2017), *Eucalanus bungii* (Eb2017), *Metridia pacifica* (Mp2017). Assembly data include assembly statistics, self-mapping percentages, presence of BUSCO genes, functional annotation (BLAST results [SwissProt], gene ontology terms [GO] and enzyme commission numbers (EC) and predicted proteins (TransDecoder). *Trinity version used for this assembly did not provide a number for predicted Trinity “genes”.

### Functional annotation

**Fig. 2:**
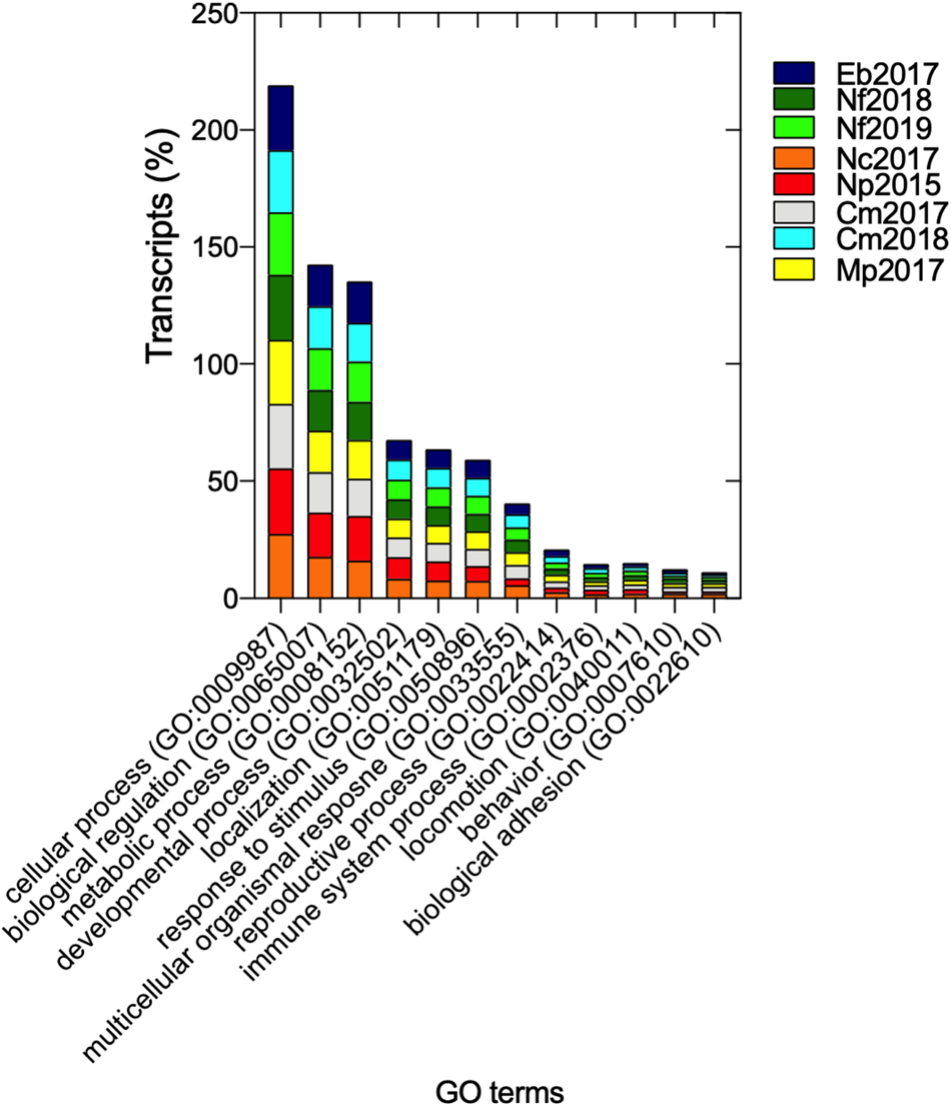
Distribution of Gene Ontology terms (GO). Stacked bar plot showing for the eight transcriptomes relative % of transcripts annotated to GO terms within the biological process category. For each transcriptome, percentages were calculated as the number of transcripts annotated to a given GO term divided by the total number of GO annotated transcripts in the transcriptome.

The functional annotation of the transcripts using the Swiss-Prot database identified over 30,000 significant BLAST hits for seven of the eight assemblies (Table 2). The assembly with the lowest number of assembled transcripts (*E. bungii*) returned fewer than 18,000 hits. The percentage of transcripts with significant Swiss-Prot hits was similar across all eight assemblies, ranging between 44% (*M. pacifica*, Mp2017) and 49% (*N. flemingeri*, Nf2019). Most of the transcripts (>95%) with BLAST hits were also annotated with gene ontology (GO) terms with a smaller number annotated with KEGG enzyme commission numbers (EC, Table 2). The distribution of GO terms across major biological processes was similar in the eight transcriptomes (Fig. 2), as expected based on other copepod *de novo* assemblies^13,52^. While the assembly for *E. bungii* was less complete, the transcripts were of high quality, and we retained this transcriptome in the downstream analyses.

### Species confirmation

COI sequences from the transcriptomes (Supplementary Information SD1) received confirmatory top species hits with >97% identity for five species even when the assembled transcript was short (Table 3). The COI sequence of the sixth species, *C. marshallae* is very similar to that of *C. glacialis* (>97%), raising questions about whether they are separate species or members of a species complex^53,54^. While very similar, the sequences and other posted *C. marshallae* complete and partial sequences (e.g. accession numbers GJQX01213347, KX675691 and KP241590), showed consistent differences with respect to *C. glacialis* sequences, especially in two locations (nucleotides 489 and 627) within the full-length 1,556 bp mtCOI sequence (Supplementary Information SD1). Thus, while very similar there appeared to be consistent genetic differences between the two species. Our analysis is consistent with a recent phylogenetic analysis using 191 genes^55^.

**Table 3:** Species COI confirmation.

Transcriptome	NCBI accession #	Top nr/nt species	% ID	nr/nt accession
Nf2019	GJRT01007932	*N. flemingeri*	98.9%	AB093141
Nf2018	GJSD01085072	*N. flemingeri*	98.6%	AB093141
Np2015	GJRU01046086	*N. plumchrus*	97.7%	AB093143
Nc2017	GJRH01042514	*N. cristatus*	99.0%	AB091773
	GJRH01042515		99.5%	“
	GJRH01042516		99.9%	“
	GJRH01042517		99.5%	“
	GJRH01042519		99.8%	“
	GJRH01042520		99.5%	“
Cm2018	GJRL01066504	C. glacialis*	98.8%	MG001883*
	GJRL01051353	“	99.0%	“
	GJRF01035907	“	99.1%	“
Cm2017	GJRF01071120	C. glacialis*	98.7%	MG001883*
	GJRF01071121	*C. marshallae*	99.4%	AF332768
Eb2017	GJRG01024003	*E. bungii*	98.6%	AB091772
Mp2017	GJAO01037389	*M. pacifica*	99.8%	AB379983
	GJAO01037394		99.8%	“
	GJAO01037399		99.8%	“

Accession numbers for transcripts in each transcriptome that annotated as mitochondrial COI sequences in BLASTx into SwissProt (see Table 1 for transcriptome codes). Last three columns are values returned by the top hit in a BLASTn of the sequence in Column 2 into NCBI nr/nt database: the species ID, the % identity and the accession number. E-values for all BLASTs were 0.0 except for GJRF01071121 ( = 3E-172). Note that the top hits for 4 of the *C. marshallae* sequences were identified as the closely-related *C. glacialis* (the representation of *C. marshallae* sequences in nt/nr is small). Two of these, indicated with *, while ranking lower in total scores, had *C. marshallae* hits with 100% identity and E-scores < e-100.

### Testing for contamination

Heterospecific contamination can occur in *de novo* assemblies from field collections and must be routinely checked for^33^. A cursory assessment of the 18 S ribosomal RNA sequences suggested some contamination from foreign sources (Supplementary Information SD2). The levels of contamination were extremely low (<1%), however, the level was 5.5% for reads mapping to a *C. glacialis* sequence present in the *M. pacifica* transcriptome. This led us to perform an additional analysis to check for cross-contamination between species by mapping the RNA-Seq data to a “species-filter” (see Methods). Bowtie mapping resulted in ca. a million reads from the *E. bungii* transcriptome mapping to its COI sequence, while none mapped to a human COI sequence or any other species in the species filter. Similarly, with almost half a million *M. pacifica* reads mapping to its COI sequence, only a total of 9 mapped to the suspected *C. marshallae/C. glacialis* COI sequences (see Supplementary Information SD1 Part II for filter composition and numerical details). We conclude that there was either no or negligible contamination from these sources with some counts even arising from cross-mapping.

### Protein prediction

TransDecoder found open reading frames (ORFs) of 100 amino acids or longer in more than 78% of the transcripts in all transcriptomes, with 22% to 38% predicted to be full length (Table 2). The full-length proteins were analyzed further using the OrthoVenn2 clustering algorithms as described in the Methods and summarized in Table 4.

**Table 4:** OrthoVenn2 results for comparisons across *de novo* transcriptomes.

Comparison	Intra-specific only		Multi-species primary^1^			
	*C. marshallae*	*N. flemingeri*	Neocalanus	Calanida	Myelinata	Calanoida
# transcriptomes	2	3	3	4	5	6
Transcriptomes	Cm2018	Nf-Ref	Nf-ref	Nf-ref	Nf-ref	Nf-ref
	Cm2017	Nf2018	Np2015	Np2015	Np2015	Np2015
		Nf2019	Nc2017	Nc2017	Nc2017	Nc2017
				Cm2018	Cm2018	Cm2018
					Eb2017	Eb2017
						Mp2019
Total Clusters	10,453	12,504	12,587	15,168	15,402	19,231
Clusters shared by 2 or more	7,174	9,343	9,435	11,567	11,699	12,630
	68.6%	74.7%	75.0%	76.2%	76.0%	65.7%
Clusters shared by all	7,174	4,686	4,366	3,519	1,635	1,387
	68.6%	37.5%	34.7%	23.2%	10.6%	7.2%

Enumeration of clusters of predicted proteins homologous across different sets of the transcriptomes presented in this paper. Transcriptomes uploaded to OrthoVenn2 in the groups indicated in each column, coded as in Table 1 (Nf-ref is the reference *N. flemingeri* transcriptome of Roncalli *et al*. 2019)^43^. Total Clusters: Number returned by OrthoVenn2 in its summary statement. Clusters shared by 2 or more: From the OrthoVenn2 diagram labeled “*Number of elements: Specific (1), or shared by 2, 3,…lists*” summing all except the “Specific (*1*)” values. Clusters shared by all: From the highest element order in the diagram (also the value given for the intersection of clusters from all transcriptomes in the Venn diagram produced by OrthoVenn2).

^1^See Supplementary Information SD3M for corresponding Venn diagrams.

### Intraspecific homologs

Within-species homologies among transcriptomes were expected to be high and an indicator of similarity across assemblies. As shown in Table 4, the percentage of the OrthoVenn2 clusters shared between the two *C. marshallae* transcriptomes approached 70%. A similar percentage was observed between at least two of the three *N. flemingeri* transcriptomes (Table 4; Supplementary Information SD3M). Interestingly, when partial-protein sequences were included in the OrthoVenn2 uploads, the shared fraction remained similar. Merging multiple transcriptomes with software such as CAP3 can be implemented by a user to produce a more complete reference at the expense of obscuring the hierarchical information provided by Trinity. Mergers also add genetic diversity owing to the use of more than one individual.

### Interspecific comparisons

As described in the Methods section, the previously-vetted *N. flemingeri* transcriptome^3^ was translated and used as a common reference since it had been used in gene expression studies, allowing identification of genes responsive to changes in environmental conditions. The numbers of complete-protein homolog clusters that were shared between this reference and each of the new transcriptomes ranged from 40% to 70% (Supplementary Information SD3), with the highest percentage shared between the *N. flemingeri* reference and its and conspecifics and two congeners, and the lowest percentages with the two more distantly-related taxa (*E. bungii* and *M. pacifica*). In the survey of the overall taxonomic extent of homologies among the primary cluster sets from the four multi-species sets of transcriptomes (Table 4), the fractions of clusters shared by all of the members (“overlap clusters”) ranged from 7.2% for the set including all 6 species of the order Calanoida, to 35% for those comprising the three species of the *Neocalanus* genus. The fraction was greater the tighter the taxonomic coverage, as would be expected. Thus, while there were the expected differences among the assembled complete proteins correlating with phylogenetic distance, the large number of overlapping clusters indicates that the individual assemblies produced mutually-consistent transcriptomes.

## Usage Notes

### Reference transcriptomes for ecophysiology

The *de novo* transcriptomes were generated from individuals belonging to six key zooplankton species of the sub-arctic waters of the Gulf of Alaska: *Neocalanus flemingeri*, *N. plumchrus*, *N. cristatus*, *Calanus marshallae, Eucalanus bungii* and *Metridia pacifica*. The assemblies and their functional annotation provide references for future RNA-Seq studies focused either on single or multiple species, as well as in community studies based on metatranscriptomics^17^. The predicted sequences can be used to design species-specific primers for PCR-based studies focused on biomarkers^56^. Homologous genes identified across transcriptomes from these six co-occurring species can be used to generate a set of references for interspecies comparisons. While there are more studies that are generating RNA-Seq data for bulk samples of planktonic organisms, interspecies comparisons, especially for zooplankton, continue to be challenging given the absence of robust community-specific references. By focusing on key species from one region, these *de novo* transcriptomes open opportunities to examine species-specific responses and niche separation under natural and/or experimental conditions.

### Resource for protein discovery

*De novo* transcriptomes are composed of predicted expressed transcripts assembled from short sequences. These transcripts are then translated into predicted proteins, which may or may not be real. In spite of this limitation, transcriptomes have been rich data sources for protein discovery^57–61^. Here, we illustrate how non-annotated transcripts with predicted homologous proteins in multiple transcriptomes might be used in the search for protein discovery (Table 5). In the single-species cluster sets for *C. marshallae* and *N. flemingeri*, around a third failed annotation through lack of significant BLAST hits in SwissProt. In the multi-species sets, the 6-calanoid primary set as well as the three secondary sets derived from it (see Supplementary Information ST1 for cluster compositions), the percentages of non-annotated clusters ranged from 15% to 20%. After removing clusters with membership outside of the selected secondary taxonomic category, as described in Methods, only a few hundred prime candidates remained, inviting further investigation (Table 5, “non-annotated taxonomically homogeneous” row).

**Table 5:** OrthoVenn2 results obtained when the predicted proteins of a primary set of homologous clusters is subsampled to generate “secondary” subsets.

	Intra-specific (primary)^1^		Secondary subsets^1^			Primary^1^
Taxonomic coverage	*C. marshallae*	*N. flemingeri*	Neocalanus	Calanidae	Myelinata	Calanoida
# transciptomes	2	3	3	4	5	6
Transcriptomes	Cm2018	Nf-Ref	Nf-ref	Nf-ref	Nf-ref	Nf-ref
	Cm2017	Nf2018	Np2015d	Np2015	Np2015	Np2015
		Nf2019	Nc2017	Nc2017	Nc2017	Nc2017
				Cm2018	Cm2018	Cm2018
					Eb2017	Eb2017
						Mp2019
# in “all” category of subset	(7,174)	(4,686)	4,299	3,562	1,638	(1,387)
# non-annotated in “all” category	2,857	1,601	643	714	343	239
	39.8%	34.2%	15.0%	20.0%	20.9%	17.2%
# non -annotated taxonomically homogeneous			240	316	104	
			37.3%	44.3%	30.3%	
#DEGs^2^			73	59	22	

Those clusters shared by all of the transcriptomes in the subset are enumerated in the “all” rows in the table (proteins irrespective of annotation status, and separately, just those not annotated in SwissProt by OrthoVenn2: see Methods). Numbers in parentheses are repeats of the same categories as in Table 4.Those sets are still heterogeneous with respect to taxonomic coverage. The taxonomically homogeneous non-annotated “all” category of the secondary subsets is generated by purging clusters that include “foreign” species, so only clusters restricted to the transcriptomes listed remain. Note that owing to the different procedures for generating “primary” vs “secondary” sets, the “all” categories in the multispecies sets differ somewhat from the corresponding ones of Table 4.

^1^See Supplement SF1 for corresponding Venn diagrams

^2^See Supplement ST2 for accession numbers.

### Environmentally-responsive genes among the non-annotated predicted proteins

We compared the candidate genes to lists of environmentally-responsive transcripts of pre-adult *N. flemingeri* (DEGs) reported from a spatial transect in the Gulf of Alaska and an interannual comparison (2015–2017) within Prince William Sound^3,5^. Among the DEGs were 101 and 83 non-annotated transcripts/clusters along the spatial gradient and the interannual comparison, respectively (Table 5). Thirty transcripts were differentially-expressed under both conditions (Supplementary Information ST2), highlighting the genes’ likely importance in the ecophysiology of this species. The availability of homologs in the other transcriptomes not only means that these genes are “real”, but also raises the question whether they may be eco-responsive in the co-occurring species under similar conditions.

### Comparisons with a rapidly-evolving gene

**Fig. 3:**
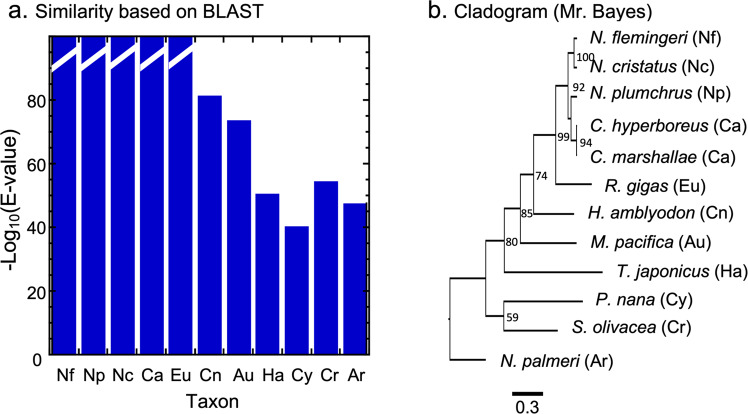
Taxonomic-similarity profile (**a**) and corresponding MrBayes cladogram (**b**) for a rapidly-evolving protein (glutathione S-transferase: GST, omega variant). (**a**) Bar graph of similarity [defined as -log_10_(E-value)] of the *N. flemingeri* homolog of the translated GST omega reference sequence (accession # GHLB01049544) in a taxonomic BLAST-scan into NCBI’s TSA database with top-hit transcripts at different phylogenetic distances. (**b**) Cladogram generated by MrBayes for the top-hit sequences shown in  (**a**). Numbers at nodes are bootstrap values (>50) computed by RAxML. Scale bar: 0.3 estimated substitutions per site. Despite the rapid evolution of this protein, differences between adjacent taxonomic categories in the bar graph are modest (<25 log units of E-value) and the stem-lengths in the cladogram are <0.2 within the Crustacea and 0.4 between the insect homolog and the Crustacea. Taxon codes for bars: Nf: *Neocalanus flemingeri*; Np: *N. plumchrus*; Nc: *N. cristatus*; Ca: family Calanidae (excluding *Neocalanus*) *Calanus marshallae*, *C. hyperboreus*; Eu: superfamily Eucalanoidea *Rhincalanus gigas*; Cn: superfamily Centropagoidea (Diaptomoidea) *Hemidiaptomus amblyodon*; Au: superfamily Augaptiloidea (Arieteloidea), *Metridia pacifica*; Ha: order Harpacticoida, *Tigriopus japonicus*; Cy: order Cyclopoida, *Paracyclopina nana*; Cr: class Crustacea (excluding Copepoda), *Scylla olivacea*; Ar: phylum Arthropoda (excluding Crustacea) *Nionia palmeri*. Sequences of predicted proteins, accession numbers and E-values are given in the Supplementary Information (SD4).

We took a further step toward testing ways of validating the novelty of these unannotated clusters of homologous environmentally-responsive predicted proteins by searching databases for similar proteins at different phylogenetic distances from the cluster. In order to provide some context, we first characterized the similarity profile of an annotated transcript from the glutathione S-transferases (GST) family as a reference. The GSTs belong to a gene family known to be rapidly evolving^62^. Using the BLAST-scan algorithm described in Methods, we took the omega GST from *N. flemingeri* as a query to identify the most similar homologous transcripts in calanoid and other copepods, crustaceans and arthropods. The bar graph in Fig. 3a shows that the similarity [ = -log_10_(E-value)] declines gradually as a function of phylogenetic distance from the *N. flemingeri* query. The cladogram in Fig. 3b, based on the same hits, shows nested clades with moderately-well-supported branching (bootstrap values >70) that includes homologs from all the calanoids as well as one from the harpacticoid, *Tigriopus*. Amino-acid substitution rates (branch lengths) between clades nested within this large clade are modest (~0.2–0.3). The cladogram largely parallels the evolutionary relationships among the Copepoda, especially the calanoid families, as would be expected for a broadly-distributed protein that changed in concert with the species’ evolution^6,22,23^.

**Fig. 4:**
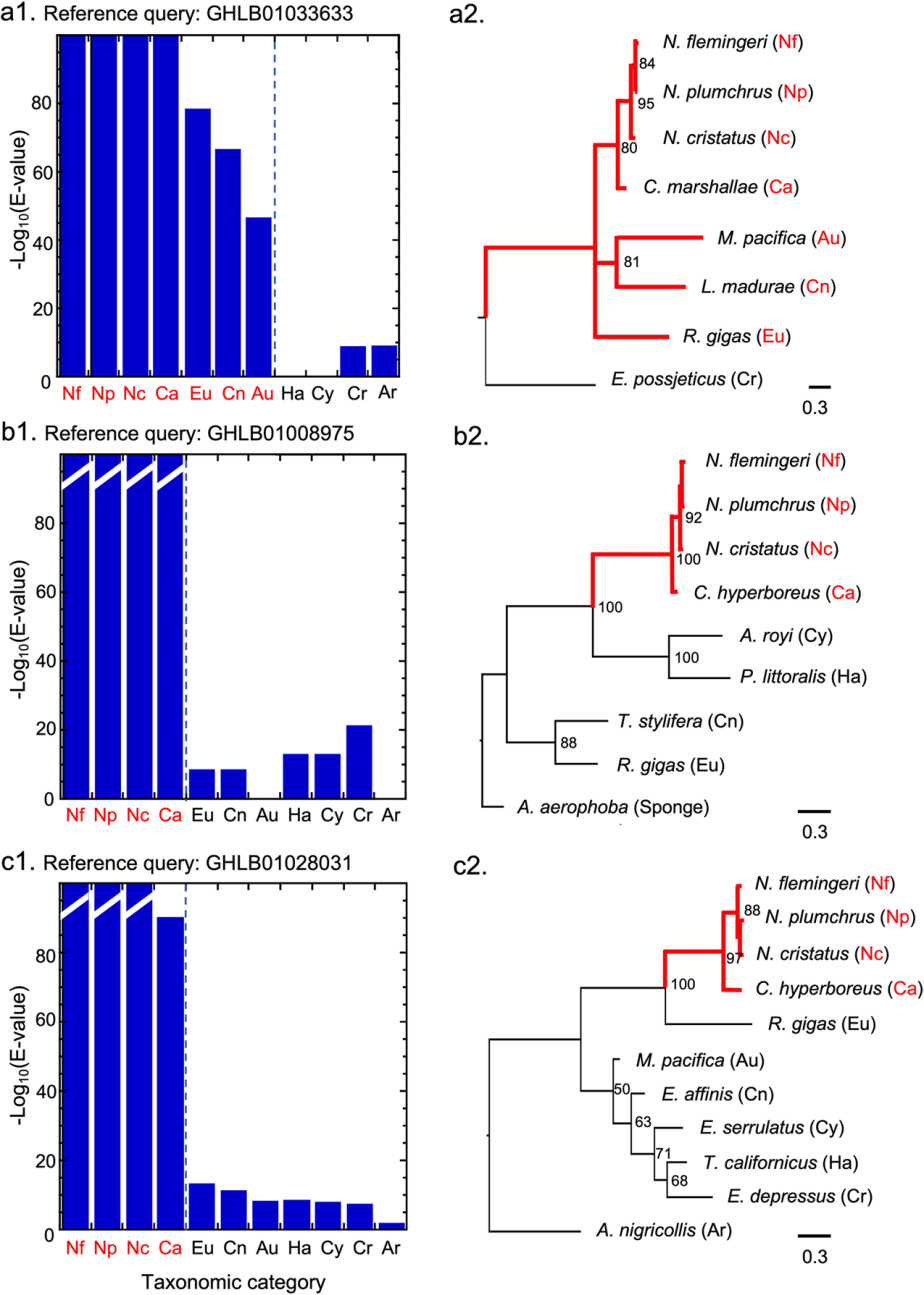
Taxonomic-similarity profiles and corresponding MrBayes cladograms of three predicted proteins without functional annotation. Left panels: Bar graphs of similarities in taxonomic BLAST-scans of the *N. flemingeri* query sequence with top-hit transcripts in available TSA transcriptomes [-log_10_(E-value)]. Abbreviations (x-axis) are the same as in Fig. 3. A sharp increase in similarity with increasing proximity to the query indicated at the top is hypothesized to be the taxon of evolutionary emergence of each non-annotated cluster (broken line). Right panels: Cladogram branch lengths are proportional to the evolutionary distance from the clade’s branch point (scale bars: 0.3 estimated substitutions per site). Taxonomic categories corresponding to the abrupt change in similarity on the left panel colored in red. Red branches indicate taxa proposed to possess a novel version of the set of homologs as identified by the corresponding similarity bar-plot profile on the left. Where possible, the outgroup used to root the tree was either the crustacean homolog (excluding Copepoda) or the arthropod homolog (excluding Crustacea). In (**b**), no adequate homolog was identified in either out-group category, so a more distant outgroup was used (a sponge). In Panels a and b, hits were missing for certain taxonomic categories: Harpacticoida and Cyclopoida for (**a**) and Augaptiloidea and Arthropoda for (**b**). In some cases, the top-hit sequence was for a partial protein that was too short to be included in a reliable cladogram, in which case the next most similar hit was used. Accession numbers for the *N. flemingeri* query sequence are given above each bar graph. Species and sequence accession numbers: (**a**) Nf: *Neocalanus flemingeri*; Np: *Neocalanus plumchrus*; Nc: *Neocalanus cristatus*, Ca: *Calanus marshallae*; Eu: *Rhincalanus gigas*; Cn: *Labidocera madurae*; Au: *Metridia pacifica*; Cr: *Eogammarus possjeticus*. (**b**) Nf: *Neocalanus flemingeri*; Np: *Neocalanus plumchrus*; Nc: *Neocalanus cristatus*, Ca: *Calanus hyperboreus*; Eu: *Rhincalanus gigas*; Cn: *Temora stylifera*; Ha: *Platychelipus littoralis*; Cy: *Apocyclops royi*; Sponge: *Aplysina aerophoba*. (**c**) Nf: *Neocalanus flemingeri*; Np: *Neocalanus plumchrus*; Nc: *Neocalanus cristatus*, Ca: *Calanus hyperboreus*; Eu: *Rhincalanus gigas*; Cn: *Eurytemora affinis*; Au: *Metridia pacifica*; Ha: *Tigriopus californicus*; Cy: *Eucyclops serrulatus*; Cr: *Eurypanopeus depressus*; Ar: *Anoplogonius nigricollis*. Sequences of predicted proteins and E-values are given in the Supplementary Information (SD4). Additional examples are provided in Supplementary Information SF2.

Environmentally-responsive predicted proteins of *N. flemingeri* that lack annotations can be compared with the GST pattern. As examples, taxonomic BLAST-scans into NCBI (TSA) of three such sequences are presented in Fig. 4. In contrast to the omega GST pattern, the predicted proteins were highly similar within a narrow taxonomic range, with a sharp decline in similarity [-log_10_(E-value)] outside of the group (Fig. 4 left panels, set 1). The high-similarity group for Fig. 4a1 included only hits from the order Calanoida. Significantly, the BLAST got no hits among the Harpacticoida or Cyclopoida, giving further emphasis to the uniqueness of that protein group. The high-similarity groups in Figure 4b1, c1 were even more restricted, being confined solely to calanid proteins. Most of the nodes leading to the *N. flemingeri* query sequence and its cluster of homologs received bootstrap support similar to that of the GST (>80). The support was particularly strong (100) for the clades that included the high-similarity (calanid) taxa in Figure 4b2, c2. For Fig. 4a2, while the branch pattern in the cladogram produced by MrBayes grouped the calanoids appropriately, bootstrap support was not strong. In all three cladogram examples, substitution rates (branch lengths) between the high-similarity taxa and those outside the group were notably longer than for the GST (>0.5). Thus both the sharp rises in the BLAST-scan similarity with taxonomic proximity to *N. flemingeri* and the long branch lengths for the corresponding points in the cladograms are consistent with a phylogenetic point of emergence of a novel protein within the Copepoda.

### Functional motifs of candidate novel proteins

**Fig. 5:**
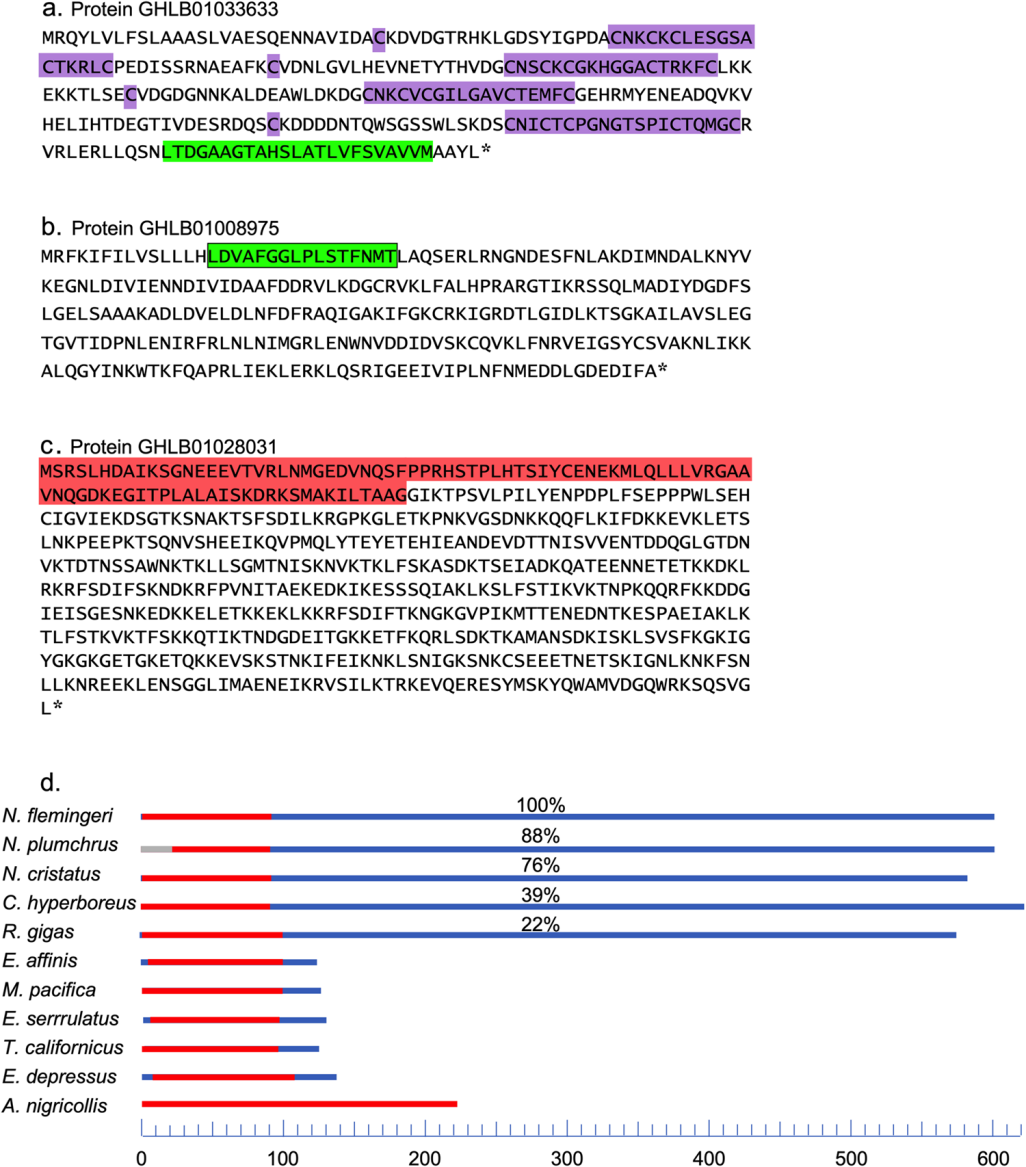
Motifs contained in the sequences for three *N. flemingeri* proteins of Fig. 4 as identified by motif-search programs (see Methods). (**a**) Pacifastin-inhibiting motifs: residues 47–70; C80 & 97–124; C128 & 145–168, C200 & 211–241 (violet highlight); Pore-forming motif: residues 258–273 (green highlight). (**b**) Pore-lining motif: residues 15–30 (green). (**c**) Ankyrin repeat motif: residues 1–191 (red highlight). (**d**) Ankyrin motifs in the sequences of the different taxonomic categories of Fig. 4c. Lengths of the different regions in protein homologs containing ankyrin motifs (red portion of bar) in the taxonomic groups and sequences from Fig. 4c. Non-ankyrin regions in blue (residue scale in decades at bottom in light blue). Note the long C-terminal segments in the Myelinata (upper 5 bars), which are absent from all other taxonomic categories. Percentages indicate percent identity between the sequence and the translated *N. flemingeri* query.

The presence of functional motifs — sequences of amino acids that have been shown in other studies to endow proteins with known properties — can provide some insights into function. All three putative novel proteins from Fig. 4 contained functional motifs as shown in Fig. 5. The protein of Fig. 5a appears to be a member the pacifastin family, as it contains the typical repeated 6-cysteine-residue motifs (Fig. 5a, violet highlight). The pacifastins are arthropod serine peptidase inhibitors that have been shown to have a role in the immune response^63^. This copepod sequence features four inhibitor motifs. Interestingly, the malacostracan (amphipod) homolog possesses many more (17; Supplementary Information SD4). The predicted protein of Fig. 5b includes a channel-pore-lining motif (green highlight), suggesting a role as a trans-membrane ion channel. Finally, in the protein of Fig. 5c, the N-terminal portion contains an ankyrin repeat motif (red highlights), which is highly conserved throughout the Arthropoda. A comparison across homologous sequences showed that the predicted proteins of the five myelinate species possess an extensive C-terminal segment that is missing from the amyelinates and more distantly-related taxa. This is shown diagrammatically in Fig. 5d which separates the conserved portion of the protein (red) from the C-terminal extension (blue) (see SD4 for all sequences).

## Supplementary information

Supplementay Material

## Data Availability

Parameters to software tools involved are described below: FastQC: version 0.11.8 FASTQ Toolkit (BaseSpace): version 2.2.5, parameters: trim of first 12 bp, Quality cutoff (Phred score <30), adapter removal Trinity: version 2.0.6 (*N. plumchrus*) and version 2.4.0 (all others), parameters: –seqType fq–CPU 32–max_memory 200 G –min_contig_length 300 –normalize_max_read_cov50 Bowtie: version 2.1.0, default parameters BUSCO: version 5.3.2, dataset: arthropoda_odb10 (Creation date: 2020-09-10, number of genomes: 90, number of BUSCOs: 1013). Sequence Manipulation Suite (SMS): (https://sites.ualberta.ca/~stothard/javascript/rev_comp.html) TransDecoder: version 3.0.0, default parameters (open reading frame > 100 amino acid) Transeq: Search and Sequence Analysis Tools Services from EMBL-EBI in 2022 https://europepmc.org/ MAFFT: version 7.511 (https://mafft.cbrc.jp/alignment/software/) BLASTp: (local Beowulf Linux computer cluster) against NCBI Swiss-Prot database: version February 2015, parameters: E-value of 10^−6^ cutoff UniProt: (http://www.uniprot.org/help/uniprotkb), February 2021, parameters: Gene Ontology and the KEGG (Kyoto Encyclopedia of Genes and Genomes; https://www.kegg.jp) databases OrthoVenn2: version 2018. Inflation value 1.5; E-value 1e-15 except as indicated. https://orthovenn2.bioinfotoolkits.net/home MrBayes: version 3.2, parameters: lset rates = gamma ngammacat = 4; prset aamodelpr = fixed(wag); mcmc ngen = 10000000 relburnin = yes burninfrac = 0.25 printfreq = 1000 samplefreq = 1000 nchains = 4 savebrlens = yes RAxML: version 8.2.12, parameters: 1,000 bootstrap replicates (-# 1000), WAG substitution model and gamma distribution of rates (-m PROTGAMMAIWAGF) FigTree: version 1.4.4 (url: http://tree.bio.ed.ac.uk/software/figtree/)
