# Supplementary material for: De novo transcriptomes of six calanoid copepods (Crustacea): a resource for the discovery of novel genes
Source: Sci Data. 2023 Apr 27;10:242. doi: 10.1038/s41597-023-02130-1 (PMC10140051; doi:10.1038/s41597-023-02130-1)
Supplement: Supplementary file 1 — Supplementay Material [file 41597_2023_2130_MOESM1_ESM.pdf]

Supplementary Information - Index

Supplement SD1 (COIs) (2 parts)

SD1 Part I - species-specific COI sequences for the 8 new transcriptomes presented in the main text.

SD1 Part II - COI sequences used to construct an "artificial transcriptome" as a species filter, as described in main text Methods.

Supplement-SD2. rRNA Contaminants. Document includes a selection of representative candidates for contaminant sequences found in the 8 transcriptomes

Supplement SD3: OrthoVenn2 statistics. Document includes summary tables and Venn diagrams produced by OrthoVenn2 for the cluster sets of homologs in various combinations of the eight transcriptomes described in the main text.

Supplement-SD4: BLAST-scans and cladogram data. Document presents BLAST-scan similarity and top-hit sequences for the exemplar rapidly-evolving GST omega protein of Fig. 3 of the main text, for the three exemplar transcripts used in Fig. 4, and for the three used in Supplementary figure SF4.

Supplement SF1: Venn diagrams by OrthoVenn2 of homology clusters from Table 5 of the main text.

Supplement SF2 - 3 additional examples of BLAST-scans and cladograms of non-annotated environmentally-reactive proteins.

Supplement ST1 - Cluster compositions for 3 taxonomic coverages of the secondary subsets of Table 5, Main text: Myelinata, Calanidae and *Neocalanus*.

Supplement ST2 - Accession numbers for the non-annotated differentially-expressed genes (transcrupts) of *Neocalanus flemingeri* from which selection of exemplars was made in the main text.

Supplement SD1 (COIs) (2 parts)

SD1 Part I: .fasta-format files for the species-specific COI sequences of the 8 *de novo* transcriptomes presented in this paper. Accession numbers are for the TSA-deposited sequences as assembled (reverse complemented in some cases). Mouse-over for URL. Short transcriptome codes from Table 1 ("Abbreviation" column) at the end of the titles. These may represent fragments of the full COI and/or may include extensions to other parts of the mitochondrial chromosome. The sequences below have been restricted to just the COI portion, amalgamating fragments where necessary. The translation to protein (EMBOSS TranSeq: https://www.ebi.ac.uk/Tools/st/emboss_transeq/) is included as a routine measure to minimize the chances of spurious stop codons or frame shifts, as is sometimes found in posted transcriptomes (e.g. GJQX01164994.1). Green highlight indicates the start codon; red the stop.

***Neocalamnus flemingeri* CV2019_PWS2_partial COI (Nf2019)**

>Nf2019_COI_**GJRT01007932** (DN24762_c0_g2_i1) *Neocalanus flemingeri* 815bp
ATAGTGTTAATATATAAGGTTAAGTGGTTATGATCATGTAATCATAAAGATATTGGTACC
CTATACTTATTAGCTGGAGCTTGGTCAGGTATAATTGGCACTGGGCTGAGGATAATCATC
CGATTAGAATTAGGTCAGGCAGGTTCTCTTATTGGAGATGATCAGATTTATAATGTCGTA
GTTACTGCTCATGCATTTATTATAATTTTTTTATGGTTATACCAATCCTTATTGGGGGG
TTCGGTAACTGGTTAGTACCTCTAATACTAGGAGCGGCCGATATGGCATTTCCACGTATA
AATAATATAAGGTTTTGGTTCTTAATACCAGCTTTGATTATGCTTCTGTCTAGATCTTTA
GTTGAAAGTGGAGCAGGGACAGGGTGAACAGTCTATCCTCCCCTCTCTAGAAATATTGCC
CATGCGGGAGGTTCTGTAGACTTCGCTATTTTCTCACTTCACTTGGCAGGTGTGAGATCT
ATTTTAGGGGCCGTAAACTTCATTAGGACCCTGGGAAACTTGCGAGTATTTGGTATATTA
TTAGACCGAATACCTTTATTTGCCTGAGCTGTTCTTATTACTGCTGTTCTCCTTCTCCTG
TCTTTACCGGTATTAGCTGGAGCTATTACAATATTGTTAACAGATCGTAACCTAAATACT
TCTTTCTATGATGTTGGGGGGGGCGGTGACCCTATTCTATACCAGCATCTATTTTGGTTC
TTTGGTCACCCTGAGGTATATATTCTGATTCTTCCTGGATTTGGTCTAATTTCACACATT
GTCGCGCAGGAGAGGGGCAAAAAGGAAACATTTGG

>Nf2019_COI_GJRT01007932_1
MVLMYKVKWLWSCNHKDIGTLYLLAGAWSGMIGTGLSMIIRLELGQAGSLIGDDQIYNVV
VTAHAFIMIFFMVMPILIGGFGNWLVPLMLGAADMAFPRMNNMSFWFLMPALIMLLSSSL
VESGAGTGWTVYPPLSSNIAHAGGSVDFAIFSLHLAGVSSILGAVNFISTLGNLRVFGML
LDRMPLFAWAVLITAVLLLLSLPVLAGAITMLLTDRNLNTSFYDVGGGGDPILYQHLFWF
FGHPEVYILILPGFGLISHIVAQESGKKETFG

***Neocalanus flemingeri* CV2018_48_S2COI (Nf2018)**

>Nf2018_COI_GJSD01085072 (DN28514_c1_g1_i1) 1551bp
ATAGTGTTAATATATAAGGTTAAGTGGTTATGATCATGTAATCATAAAGATATTGGTACC
CTATACTTATTAGCTGGAGCTTGGTCAGGTATAATTGGCACTGGGCTGAGGATAATCATC
CGATTAGAATTAGGTCAGGCAGGTTCTCTTATTGGAGATGATCAGATTTATAATGTCGTA
GTTACTGCTCATGCATTTATTATAATTTTTTTATGGTTATACCAATCCTTATTGGGGGG
TTCGGTAACTGGTTAGTACCTCTAATACTAGGAGCGGCCGATATGGCATTTCCACGTATA
AATAATATAAGGTTTTGGTTCTTAATACCAGCTTTGATTATGCTTCTGTCTAGATCTTTA
GTTGAAAGTGGAGCAGGGACAGGGTGAACAGTCTATCCTCCCCTCTCTAGAAATATTGCC
CATGCGGGAGGTTCTGTAGACTTCGCTATTTTCTCACTTCACTTGGCAGGTGTGAGATCT
ATTTTAGGGGCCGTAAACTTCATTAGGACCCTGGGAAACTTGCGAGTATTTGGTATATTA
TTAGACCGAATACCTTTATTTGCCTGAGCTGTTCTTATTACTGCTGTTCTCCTTCTCCTG
TCTTTACCGGTATTAGCTGGAGCTATTACAATATTGTTAACAGATCGTAACCTGAATACT
TCTTTCTATGATGTTGGGGGGGGCGGTGACCCTATTCTATACCAGCATCTATTTTGGTTC
TTTGGTCACCCTGAGGTATATATTCTGATTCTTCCTGGATTTGGTCTAATTTCACACATT
GTCGCGCAGGAGAGGGGTAAAAAGGAAACATTTGGTGTACTGGGTATAATCTATGCCATG
TTAGCCATTGGAGTACTAGGATTTGTTGTATGGGCCCATCACATGTTTACTGTGGGGATG

GATGCTGACACGCGTGCTTACTTTACAACAGCTACAATAATTATTGCCGTACCTACCGGT
ATTAAAGTCTTCAGGTGATTGGGGACCTTCCACGGTGTCCGATTCTCTATGTCTCCATCA
TTACTCTGATCTTTAGGGTTCGTTTTTCTTTTTACTGTGGGAGGGTTAACAGGAATTGTA
TTATCAAACTCTTCTTTAGATGTTGTCCTCCACGATACTTACTATGTTGTTGCTCACTTC
CATTATGTGTTAAGAATGGGCGCTGTTTTCGCGCTGATGGCAGCATTTGTAAATTGATTT
CCCCTTATGGTAGGTCTAACAATAAATCCCAAGTGATTAAAGATCCAGTTCATAATAATA
TTTGTAGGAGTAAACCTCACCTTCTTCCCCATACATTTTTTAGGTTTGGCAGGTATACCT
CGTCGGTATTCAGACTACCCTGACAGGTTTATATATTGAAATGTATTCGCAAGTGTGGGT
TCTATTGTATCTGTTGTATCTGTTATATTTTTTCTGTTTATTCTCTGGGAGGCTTTTGTA
AGGCACCGGCCAGCAATCTCTAGTAACCATATAAGTACTTCACTTGAAATAATACATTCT
TTCCCCCCTATAAACCATAGCTATACATCTATTCCTATAGTTGTGTCATAA

>Nf2018_COI_GJSD01085072_1
MVLMYKVKWLWSCNHKDIGTLYLLAGAWSGMIGTGLSMIIRLELGQAGSLIGDDQIYNVV
VTAHAFIMIFFMVMPILIGGFGNWLVPLMLGAADMAFPRMNNMSFWFLMPALIMLLSSSL
VESGAGTGWTVYPPLSSNIAHAGGSVDFAIFSLHLAGVSSILGAVNFISTLGNLRVFGML
LDRMPLFAWAVLITAVLLLLSLPVLAGAITMLLTDRNLNTSFYDVGGGGDPILYQHLFWF
FGHPEVYILILPGFGLISHIVAQESGKKETFGVLGMIYAMLAIGVLGFVVWAHHMFTVGM
DADTRAYFTTATMIIAVPTGIKVFSWLGTFHGVRFSMSPSLLWSLGFVFLFTVGGLTGIV
LSNSSLDVVLHDTYYVVAHFHYVLSMGAVFALMAAFVNWFPLMVGLTMNPKWLKIQFMMM
FVGVNLTFFPMHFLGLAGMPRRYSDYPDSFMYWNVFASVGSIVSVVSVMFFLFILWEAFV
SHRPAISSNHMSTSLEMMHSFPPMNHSYTSIPMVVS*

***Neocalanus plumchrus* male2015_R1 COI** (Np2015)

>Np2015_COI_GJRU01046086 TR23204_c0_g1_i1 len=1551
ATAGTGTTAATATATAAAGTTAAGTGGTTATGGTCATGTAACCATAAAGATATTGGTACC
TTATACTTATTAGCTGGGGCCTGGTCAGGTATAATTGGTACCGGATTAAGAATAATCATC
CGCCTAGAGTTAGGTCAAGCTGGCTCACTTATTGGGGATGATCAGATTTATAATGTTGTG
GTTACTGCTCACGCATTTATCATAATTTTTTTCATGGTTATACCAATCCTAATTGGTGGG
TTCGGTAATTGACTTGTCCCTTTAATACTGGGGGCAGCAGATATGGCGTTTCCTCGTATA
AATAACATAAGGTTTTGGTTTTTGATACCAGCCTTAATTATACTTTTATCGAGATCTTTA
GTTGAAAGGGGAGCGGGAACAGGGTGAACCGTATACCCGCCTCTATCTAGAAACATTGCC
CATGCTGGAGGTTCTGTGGACTTTGCTATTTTTTCCCTTCACTTGGCGGGTGTTAGGTCT
ATTTTAGGAGCTGTAAATTTTATCAGAACCCTTGGGAATTTGCGAGTATTTGGAATACTA
TTAGACCGTATACCTTTGTTTGCCTGAGCTGTTCTTATCACGGCCGTACTATTACTATTA
TCTTTGCCAGTATTAGCCGGTGCCATTACTATACTCCTAACAGACCGTAATCTTAATACT
TCATTTTATGATGTCGGGGGAGGAGGTGACCCTATTCTATACCAACACTTATTTTGATTC
TTTGGGCACCCTGAAGTTTACATTTTAATTCTTCCAGGATTTGGGTTAATTTCACATATT
GTGGCTCAAGAAAGTGGTAAAAAAGAGACATTTGGTGTTCTTGGAATAATTTATGCTATA
TTAGCTATTGGGGTGTTAGGCTTTGTTGTGTGGGCACACCATATATTTACTGTGGGGATG
GACGCCGATACACGTGCCTACTTTACAACTGCTACAATAATTATTGCGGTCCCTACGGGA
ATTAAAGTATTTAGGTGATTAGGAACCTTTCATGGTGTCCGATTCTCTATATCACCTTCA
TTACTTTGATCTTTAGGGTTTGTCTTTCTTTTTACTGTGGGAGGATTAACAGGAATCGTA
CTGTCTAACTCGTCTTTAGACGTAGTACTTCATGATACCTATTATGTTGTTGCACATTTT
CACTATGTTTTAAGAATAGGAGCTGTTTTTGCCCTGATAGCGGCATTTGTCAATTGATTC
CCTCTTATGGTGGGGTTAACAATAAATCCTAAGTGGTTGAAGATCCAGTTTATAATAATA
TTTGTCGGAGTAAATCTAACTTTTTTCCCAATACATTTCTTAGGTCTGGCAGGTATACCT
CGCCGGTATTCTGATTATCCTGATAGATTTATATACTGAAACGTATTCGCTAGTGTTGGA
TCTATTGTATCCGTTGTATCAGTTATATTTTTTCTGTTTATCCTTTGGGAAGCCTTTGTA
AGACATCGGCCAGCAATTTCAAGGAACCATATAAGCACCTCACTTGAGATAATACATTCT
TTTCCCCCTATAAATCATAGCTATACATCTATCCCTATAGTTGTATCATAA

>Np2015_COI_GJRU01046086_1
MVLMYKVKWLWSCNHKDIGTLYLLAGAWSGMIGTGLSMIIRLELGQAGSLIGDDQIYNVV

VTAHAFIMIFFMVMPILIGGFGNWLVPLMLGAADMAFPRMNNMSFWFLMPALIMLLSSSL
VESGAGTGWTVYPPLSSNIAHAGGSVDFAIFSLHLAGVSSILGAVNFISTLGNLRVFGML
LDRMPLFAWAVLITAVLLLLSLPVLAGAITMLLTDRNLNTSFYDVGGGGDPILYQHLFWF
FGHPEVYILILPGFGLISHIVAQESGKKETFGVLGMIYAMLAIGVLGFVVWAHHMFTVGM
DADTRAYFTTATMIIAVPTGIKVFSWLGTFHGVRFSMSPSLLWSLGFVFLFTVGGLTGIV
LSNSSLDVVLHDTYYVVAHFHYVLSMGAVFALMAAFVNWFPLMVGLTMNPKWLKIQFMMM
FVGVNLTFFPMHFLGLAGMPRRYSDYPDSFMYWNVFASVGSIVSVVSVMFFLFILWEAFV
SHRPAISSNHMSTSLEMMHSFPPMNHSYTSIPMVVS*

***Neocalanus cristatus* CV2017_82-S6 COI** (Nc2017)

>Nc2017_COI_GJRH01042516.1 (DN22388_c0_g1_i3) nt1114-2664--> len= 1551bp
ATAGTGTTAATATATAAGGTTAAGTGATTATGGTCTTGTAATCATAAAGATATTGGTACT
TTATATTTATTAGCTGGTGCCTGGTCAGGTATAATTGGTACGGGTTTAAGAATAATCATT
CGGCTAGAGCTCGGTCAGGCGGGCTCATTAATCGGTGATGATCAGATTTATAATGTAGTA
GTTACTGCTCACGCATTTATCATAATTTTTTTTATGGTAATACCTATTTTAATTGGAGGG
TTTGGTAACTGGCTAGTGCCACTAATATTGGGTGCGGCAGACATGGCATTCCCCCGTATA
AATAATATAAGGTTCTGATTCTTGATACCAGCATTAATTATACTATTATCTAGCTCTTTA
GTGGAAAGTGGAGCAGGAACCGGGTGAACGGTGTACCCTCCCCTATCTAGTAATATTGCT
CATGCTGGCGGGTCTGTGGATTTTGCTATTTTCTCATTACATCTTGCGGGTGTGAGGTCT
ATTTTAGGTGCTGTAAATTTTATTAGAACCCTAGGAAACTTGCGGGTATTTGGAATACTA
TTAGATCGGATACCTCTTTTTGCCTGGGCTGTACTTATTACTGCCGTCTTGCTACTATTA
TCTCTACCTGTATTAGCTGGGGCTATTACAATACTACTCACAGACCGTAACTTAAATACC
TCATTTTATGATGTCGGGGGGGGTGGAGATCCTATCTTATACCAACACTTGTTTTGGTTT
TTTGGGCATCCTGAGGTATATATTCTAATTCTCCCAGGATTTGGGCTAATCTCACATATC
GTAGCTCAGGAGAGTGGTAAAAAAGAAACATTTGGAGTATTAGGAATAATTTATGCTATA
TTAGCAATTGGGGTCCTAGGGTTTGTAGTATGAGCTCACCATATGTTTACTGTCGGAATA
GACGCAGATACACGGGCCTATTTTACTACTGCCACAATGATTATTGCCGTGCCAACTGGT
ATTAAGGTGTTCAGGTGGTTAGGTACATTTCACGGTGTGCGATTTTCTATATCCCCCTCA
CTACTGTGATCCTTAGGGTTCGTATTCCTTTTTACCGTGGGGGGCCTAACAGGCATTGTT
TTATCTAATTCGTCATTAGATGTTGTACTTCATGATACATATTATGTTGTCGCACATTTC
CACTATGTACTAAGAATAGGAGCTGTTTTTGCATTAATAGCGGCATTTGTAAACTGGTTT
CCATTAATGGTAGGATTAACAATAAACCCTAAGTGACTAAAAATTCAATTTATAATAATA
TTTGTGGGTGTAAATCTTACTTTTTTCCCTATACATTTCTTAGGTTTAGCGGGAATGCCT
CGTCGTTATTCTGATTATCCTGATAGATTTATATATTGAAACGTGTTTGCCAGAGTTGGT
TCTATTGTATCTGTTGTATCTGTAATGTTTTTCCTGTTTATTTTGTGAGAAGCATTTGTC
AGCCATCGGCCCGCAATTTCAAGTAACCATATAAGAACTTCGCTTGAAATGATACACTCT
TTCCCCCCCATAAACCATAGTTACACTTCAATTCCTATAGTTGTTTCTTAA

>Nc2017_COI_GJRH01042516.1_1
MVLMYKVKWLWSCNHKDIGTLYLLAGAWSGMIGTGLSMIIRLELGQAGSLIGDDQIYNVV
VTAHAFIMIFFMVMPILIGGFGNWLVPLMLGAADMAFPRMNNMSFWFLMPALIMLLSSSL
VESGAGTGWTVYPPLSSNIAHAGGSVDFAIFSLHLAGVSSILGAVNFISTLGNLRVFGML
LDRMPLFAWAVLITAVLLLLSLPVLAGAITMLLTDRNLNTSFYDVGGGGDPILYQHLFWF
FGHPEVYILILPGFGLISHIVAQESGKKETFGVLGMIYAMLAIGVLGFVVWAHHMFTVGM
DADTRAYFTTATMIIAVPTGIKVFSWLGTFHGVRFSMSPSLLWSLGFVFLFTVGGLTGIV
LSNSSLDVVLHDTYYVVAHFHYVLSMGAVFALMAAFVNWFPLMVGLTMNPKWLKIQFMMM
FVGVNLTFFPMHFLGLAGMPRRYSDYPDSFMYWNVFASVGSIVSVVSVMFFLFILWEAFV
SHRPAISSNHMSTSLEMMHSFPPMNHSYTSIPMVVS*

***Calanus marshallae* CV2018 COI** (Cm2018)

This sequence is an amalgamation of two separate fragments, accession numbers given, with a 172nt bridge between the two provided by a consensus sequence of several passes of a PacBio run

>Cm2018_COI_GJRL01066504+GJRL01051353 (DN26660_c0_g2_i1& DN27669_c2_g3_i2_n1_21-S1) len=1557bp (Bridge: T692-G862 from Pacbio assembly) G484 and G627 are diagnostic for *C. marshallae*, distinguishing it from *C. glacialis*.
ATGGGAAGATTTACAGTTAAATGACTATGATCATGTAATCATAAGGACATTGGCACCCTA
TATTTATTGGCTGGGGCGTATTCTGGTATAATTGGCACAGGGCTGAGTATGATTATTCGA
CTAGAGCTGGGCCAAGCAGGCTCACTTATTGGAGATGATCAGATCTATAACGTAGTGGTA
ACAGCCCACGCGTTTATCATAATCTTCTTTATAGTAATACCCATCTTAATTGGCGGGTTT
GGAAACTGGTTAGTTCCTTTAATGTTGGGTGCGGCGGACATGGCTTTCCCCCGAATAAAT
AACATGAGTTTCTGATTTCTTATGCCAGCACTAATCATGCTCCTATCAAGATCCTTAGTG
GAGAGGGGCGCAGGAACGGGTTGAACTGTTTATCCTCCCTTATCAAGTAATATTGCACAT
GCGGGGGCTTCTGTGGATTTTGCTATCTTCTCCCTGCACTTAGCCGGAGTTAGCTCGATC
TTGGGGGCTGTAAATTTTATTAGTACTTTGGGGAACCTTCGTGTGTTTGGTATACTTTTA
GACCGTATGCCACTATTTGCTTGGGCGGTACTCATTACCGCCGTCCTTCTCCTATTATCA
TTACCCGTCCTAGCAGGAGCTATCACGATATTATTAACAGATCGAAACCTAAATACGACC
TTCTATGACGTGGGGGGGGGGGAGATCCTATCTTATACCAGCACCTATTTTGATTTTTT
GGCCACCCTGAGGTGTATATTCTAATTTTRCCCGGATTTGGACTCATCTCTCACATTGTG
GCCCAAGAGAGTGGAAAAAGGAAACATTCGGAGTATTAGGGATAATCTACGCAATATTG
GCAATTGGTATTTTAGGGTTCGTAGTCTGGGCACACCATATATTCACTGTTGGTATGGAT
GCCGACACTCGTGCCTATTTTACGACGGCAACAATGATTATCGCGGTTCCCACGGGAATC
AAAGTTTTTAGGTGATTGGGAACATTTCACGGGGTGCGGTTCTCAATATCTCCCTCGTTG
TATTGATCTCTTGGGTTTGTCTTCCTATTTACGGTGGGGGGCCTAACAGGTATTGTATTA
TCTAATTCGTCACTAGACGTCGTGCTTCATGATACGTATTACGTCGTGGCGCATTTTCAC
TACGTACTCAGTATGGGCGCCGTGTTTGCATTAATAGCTGCCTTCATCAACTGATTCCCC
CTTATAGTAGGATTAACAATAAACCCTAAATGGTTGAAAGCGCAGTTTATTATAATATTC
ACAGGGGTAAATATAACTTTTTTCCCCATACATTTTTTAGGGTTGGCTGGGATGCCTCGA
CGGTATTCTGATTACCCTGACAGATTTATATACTGAAACGTGTTCGCCAGAGTAGGATCA
ATCGTGTCGATTATATCTGTCATATTTTTCCTATTTATCTTGTGAGAGTCCCTAGCTAGG
CACCGCCCTGCTATTTCTAGTAATCATTTGAGAACCTCAGTAGAACTCATACACTCTTTT
CCACCCATAGGTCACAGGTATGCCTCTATTCCTGTCCTAAGGGTCCAACGTGGCTAA

>Cm2018_COI_GJRL01066504+GJRL01051353_1
MGSFTVKWLWSCNHKDIGTLYLLAGAYSGMIGTGLSMIIRLELGQAGSLIGDDQIYNVVV
TAHAFIMIFFMVMPILIGGFGNWLVPLMLGAADMAFPRMNNMSFWFLMPALIMLLSSSLV
ESGAGTGWTVYPPLSSNIAHAGASVDFAIFSLHLAGVSSILGAVNFISTLGNLRVFGMLL
DRMPLFAWAVLITAVLLLLSLPVLAGAITMLLTDRNLNTTFYDVGGGGDPILYQHLFWFF
GHPEVYILI**L**PGFGLISHIVAQESGKKETFGVLGMIYAMLAIGILGFVVWAHHMFTVGMD
ADTRAYFTTATMIIAVPTGIKVFSWLGTFHGVRFSMSPSLYWSLGFVFLFTVGGLTGIVL
SNSSLDVVLHDTYYVVAHFHYVLSMGAVFALMAAFINWFPLMVGLTMNPKWLKAQFIMMF
TGVNMTFFPMHFLGLAGMPRRYSDYPDSFMYWNVFASVGSIVSIMSVMFFLFILWESLAS
HRPAISSNHLSTSVELMHSFPPMGHSYASIPVLSVQRG*

***Calanus marshallae* CV2017** (Cm2017)

This sequence is an amalgamation of three fragments, accession numbers given, with a 126nt bridge between the last two provided by a consensus sequence of several passes of a PacBio run

>Cm2017_COI_GJRF01071120+GJRF01071121+pacbio+GJRF01035907 (DN31579_c0_g1_i1 + g2_i1 + DN31398_c0_g1_i2) 1557bp; Bridge: A690-A815 from PacBio)
ATGGGAAGATTTACAGTTAAATGACTATGATCA**T**GTAATCATAAGGACATTGGCACCCTA
TATTTATTGGCTGGGGCGTATTCTGGTATAATTGGCACAGGGCTGAGTATGATTATTCGA
CTAGAGCTGGGCCAAGCAGGCTCACTTATTGGAGATGATCAGATCTATAACGTAGTGGTA
ACAGCCCACGCGTTTATCATAATCTTCTTTATAGTAATACCCATCTTAATTGGCGGGTTT
GGAAACTGGTTAGTTCCTTTAATGTTGGGTGCGGCGGACATGGCTTTCCCCCGAATAAAT
AACATGAGTTTCTGATTTCTTATGCCAGCACTAATCATGCTCCTATCAAGATCCTTAGTG
GAGAGGGGCGCAGGAACGGGTTGAACTGTTTATCCTCCCTTATCAAGTAATATTGCACAT
GCGGGGGCTTCTGTGGATTTTGCTATCTTCTCCCTGCACTTAGCCGGAGTTAGCTCGATC
TTGGGGGCTGTAAATTTTATTAGTACTTTGGGGAACCTTCGTGTGTTTGGTATACTTTTA
GACCGTATGCCACTATTTGCTTGGGCGGTACTCATTACCGCCGTCCTTCTCCTATTATCA

TTACCCGTCCTAGCAGGAGCTATCACGATATTATTAACAGATCGAAACCTAAATACGACC
TTCTATGACGTGGGGGGGGGGGGAGATCCTATCTTATACCAGCACCTATTTTGATTTTTT
GGCCACCCTGAGGTGTATATTCTAATTTTACCCGGATTTGGACTCATCTCTCACATTGTG
GCCCAAGAGAGTGGGAAAAAGGAAACATTCGGAGTATTAGGGATAATCTACGCAATATTG
GCAATTGGTATTTTAGGGTTCGTAGTCTGGGCACACCATATATTCACTGTTGGTATGGAT
GCCGACACTCGTGCCTATTTTACGACGGCAACAATGATTATCGCGGTTCCCACGGGAATC
AAAGTTTTTAGGTGATTGGGAACATTTCACGGGGTGCGGTTCTCAATATCTCCCTCGTTG
TATTGATCTCTTGGGTTTGTCTTCCTATTTACGGTGGGGGGCCTAACAGGTATTGTATTA
TCTAATTCGTCACTAGACGTCGTGCTTCATGATACGTATTACGTCGTGGCGCATTTTCAC
TACGTACTCAGTATGGGCGCCGTGTTTGCATTAATAGCTGCCTTCATCAACTGATTCCCC
CTTATAGTAGGATTAACAATAAACCCTAAATGGTTGAAAGCGCAGTTTATTATAATATTC
ACAGGGGTAAATATAACTTTTTTCCCCATACATTTTTTAGGGTTGGCTGGGATGCCTCGA
CGGTATTCTGATTACCCTGACAGATTTATATACTGAAACGTGTTCGCCAGAGTAGGATCA
ATCGTGTCGATTATATCTGTCATATTTTTCCTATTTATCTTGTGAGAGTCCCTAGCTAGG
CACCGCCCTGCTATTTCTAGTAATCATTTGAGAACCTCAGTAGAACTCATACACTCTTTT
CCACCCATAGGTCACAGGTATGCCTCTATTCCTGTCCTAAGGGTCCAACGTGGCTAA

>Cm2017_COI_GJRF01071120+
MGSFTVKWLWSCNHKDIGTLYLLAGAYSGMIGTGLSMIIRLELGQAGSLIGDDQIYNVVV
TAHAFIMIFFMVMPILIGGFGNWLVPLMLGAADMAFPRMNNMSFWFLMPALIMLLSSSLV
ESGAGTGWTVYPPLSSNIAHAGASVDFAIFSLHLAGVSSILGAVNFISTLGNLRVFGMLL
DRMPLFAWAVLITAVLLLLSLPVLAGAITMLLTDRNLNTTFYDVGGGGDPILYQHLFWFF
GHPEVYILILPGFGLISHIVAQESGKKETFGVLGMIYAMLAIGILGFVVWAHHMFTVGMD
ADTRAYFTTATMIIAVPTGIKVFSWLGTFHGVRFSMSPSLYWSLGFVFLFTVGGLTGIVL
SNSSLDVVLHDTYYVVAHFHYVLSMGAVFALMAAFINWFPLMVGLTMNPKWLKAQFIMMF
TGVNMTFFPMHFLGLAGMPRRYSDYPDSFMYWNVFASVGSIVSIMSVMFFLFILWESLAS
HRPAISSNHLSTSVELMHSFPPMGHSYASIPVLSVQRG*

***Eucalanus bungii* CV2017** (Eb2017)

>Eb2017_COI_GJRG01024003 (DN23311_c1_g2_i1) r/c len=1533 bp [ATC=invertebrate alternative start codon]
ATCAAATGACTATGGTCCTGTAACCATAAAGACATCGGAACGTTATACCTCTTAGCTGGG
GCGTGGGCAGGAATAGTAGGAACTGGACTAAGTATCTTAATTCGGTTAGAACTTGGCCAG
GCAGGGTCCCTCATTGGGGACGATCAAATTTACAATGTAGTCGTTACGGCTCATGCTTTC
ATCATAATTTTTTTCATGGTCATGCCAGTCCTTATTGGAGGGTTTGGAAACTGACTAGTG
CCACTTATACTGGGGGCAGCCGATATGGCCTTCCCTCGAATAAATAATATAAGGTTTTGA
TTTTTACTCCCTGCCTTAATTATGCTCTTATCAAGAGCTTTGGTAGAGAGAGGCGCCGGT
ACAGGTTGAACTGTCTACCCTCCACTATCAAGAAATGTCGCCCACGCTGGAGCTTCAGTG
GATTTTGCAATTTTCTCACTCCACCTGGCAGGTGTGAGATCCATTTTAGGCGCTGTCAAT
TTCATTAGGACAGTAGGAAATCTTCGAGTTTTTGGCATGCTTCTGGACCGGATACCTCTG
TTTGCCTGAGCTGTCGTGATTACAGCGGTCTTACTTCTGCTCTCCCTCCCTGTGCTAGCC
GGAGCTATTACTATGCTACTCACTGACCGTAACCTCAACACCTCCTTCTACGACGTAGGT
GGTGGCGGGGATCCTATTTTATACCAGCACTTGTTTTGATTTTTTGGTCACCCTGAGGTC
TATATTCTCATTCTACCTGCCTTCGGACTAATTTCTCACATTGTGGCTCAAGAGAGAGGT
AAGAAAGAGACTTTTGGCGTGCTAGGGATGATTTACGCCATACTGGCTATCGGTGTTTTA
GGGTTTGTTGTCTGAGCACACCACATGTTCACGGTTGGTATGGACGCTGACACGCGTGCG
TATTTTACAACTGCAACAATAATTATTGCGGTTCCAACGGGTATTAAAATTTTTAGTTGA
CTTGGAACTTTCCATGGGGTGCGATTTAGGATATCGCCAGCGCTCGTTTGAAGGCTCGGC
TTTGTTTTCCTGTTCACTGTCGGGGGGCTCACAGGTATTGTGCTATCTAATTCATCCATT
GACGTAGTCTTGCACGACACGTACTATGTAGTCGCGCACTTCCATTACGTGCTCAGAATG
GGCGCGGTATTTGGCTTAATGGCCGGGATTGTTAACTGATTCCCATTAATAGTGGGGTTA
ACTATGAACCCTAAATGGCTCAAGGCTCAGTTTTTTATAATGTTTGTCGGGGTCAATGTC
ACTTTTTTCCCGATACATTTCCTAGGCCTGGCTGGAATGCCTCGCCGATACTCAGACTTC
CCTGACACCCTTCTATATTGAAACGTCGTCGCAAGGGTGGGGTCTGTTATCTCAGTAATT
TCTGTTCTGTTTTTTCTTTTTATTTTATGAGAAGCCTGAGCTAGGCATCGCCCAGCTATC
TCCAGGTGCCACATGAGAACAAATTTAGAGTTGATACATTCTTTCCCCCCAATGAATCAC

AGGTACACCTCTATTCCAGTTATCAGGTATTAA

>Eb2017_COI_GJRG01024003_1
IKWLWSCNHKDIGTLYLLAGAWAGMVGTGLSILIRLELGQAGSLIGDDQIYNVVVTAHAF
IMIFFMVMPVLIGGFGNWLVPLMLGAADMAFPRMNNMSFWFLLPALIMLLSSALVESGAG
TGWTVYPPLSSNVAHAGASVDFAIFSLHLAGVSSILGAVNFISTVGNLRVFGMLLDRMPL
FAWAVVITAVLLLLSLPVLAGAITMLLTDRNLNTSFYDVGGGGDPILYQHLFWFFGHPEV
YILILPAFGLISHIVAQESGKKETFGVLGMIYAMLAIGVLGFVVWAHHMFTVGMDADTRA
YFTTATMIIAVPTGIKIFSWLGTFHGVRFSMSPALVWSLGFVFLFTVGGLTGIVLSNSSI
DVVLHDTYYVVAHFHYVLSMGAVFGLMAGIVNWFPLMVGLTMNPKWLKAQFFMMFVGVNV
TFFPMHFLGLAGMPRRYSDFPDTLLYWNVVASVGSVISVISVLFFLFILWEAWASHRPAI
SSCHMSTNLELMHSFPPMNHSYTSIPVISY*

Metridia pacifica **AF2017** (Mp2017)

>Mp2017_COI_GJAO01037389 (DN22824_c0_g1_i1) len=1551 [ATT=invertebrate alternative start codon]
ATTAACAGGTACAAGTTAAAATGACTGTGGTCTTGTAACCATAAAGACATTGGCACATTA
TATATGGTAGCAGGGGCATGAAGAGGGATGGTGGGCACAGGCTTAAGGGTTTTAATTCGC
CTAGAGTTAGGCCAGCCAGGATCTTTAATTGGGGACGATCAGATTTATAATGTTGTAGTG
ACAGCTCATGCTTTTATTATAATTTTTTTTATAGTTATGCCTATTTTAATTGGGGGATTT
GGAAACTGGCTAGTGCCCTTAATACTAGGGGCTGCGGATATGGCTTTCCCCCGGCTGAAT
AACATAAGGTTTTGATTTTTAGTACCTGCCTTAGTACTCTTATTAACGAGGGCGCTAGTG
GAAAGAGGGGCCGGTACTGGGTGAACTGTCTACCCCCCCCTGGCAAGGAATGTGGCGCAT
GCCGGGAGCTCTGTAGACTTCGCCATTTTTTCGCTTCATTTGGCAGGGGTCTCTTCAATC
TTAGGGGCAATTAATTTTATTAGAACCCTGGGTAATCTCCGGGTGTTTGGAATATTCTTA
GACCGGATGCCTTTGTTCGCATGAGCTGTCTTAATCACAACTATTTTACTACTTCTTTCC
CTTCCTGTTTTAGCCGGGGCTATTACAATGCTTTTGACAGATCGTAACTTAAACACCACC
TTCTATGACGTAGGAGGGGGAGGGGACCCTATTCTTTACCAGCACCTGTTTTGATTTTTT
GGGCACCCCGAGGTTTATATTTTAATTCTCCCGGGGTTTGGATTAATTTCCCACATAGTG
GCTCAAGAAAGGGGTAAAAGAGAGACCTTTGGGGTTCTAGGGATGATCTATGCAATATTA
GCAATTGGGGTTTTAGGGTTTGTAGTATGGGCCCATCATATATTCACGGTGGGCATAGAT
GCAGATACTCGAGCCTATTTTACGACTGCTACTATAATTATTGCAGTTCCCACAGGGATT
AAAATTTTTAGATGATTGGGCACTTTTCATGGGGTTCGGTTTATCTTGTCCCCCTCTTTA
ATATGGGCGCTCGGGTTCGTCTTTTTATTTACAGTTGGGGGGCTCACGGGGATTGTCTTA
TCTAACTCTTCTTTAGATGTGGTATTACATGACACCTACTATGTAGTAGCCCATTTTCAC
TATGTATTAAGTATAGGGGCAGTATTTGCACTAATAGGGGCGTTAATTAACTGGTTCCCT
TTAATAGTGGGTCTAACTATAAACCCTGTATGAACAAAAATCCAGTTTTTATTAATATTT
GTAGGGGTAAATATTACTTTTTTCCCTATGCATTTTTTAGGCCTGGCAGGCATACCGCGG
CGGTATTCGGACTACCCTGATACCTTTGCGACCTGAAATGTTATCGCAAGAATAGGCTCC
ATTGTCTCTGTTATTTCAGTAATAGGTCTCCTGTTTATTCTGTGGGAGGCTTTGGCAAGA
CACCGCCCTGCAATTTCTAGTTGTCATTTAAACACAAGAATTGAAATAATACACTCTTTC
CCCCCTACGGCCCATAGTTATTCTTCTGTGCCGGGGGTAGCCTTATCCTAG

>Mp2017_COI_GJAO01037389_1 (DN22824_c0_g1_i1) len=1551
INSYKLKWLWSCNHKDIGTLYMVAGAWSGMVGTGLSVLIRLELGQPGSLIGDDQIYNVVV
TAHAFIMIFFMVMPILIGGFGNWLVPLMLGAADMAFPRLNNMSFWFLVPALVLLLTSALV
ESGAGTGWTVYPPLASNVAHAGSSVDFAIFSLHLAGVSSILGAINFISTLGNLRVFGMFL
DRMPLFAWAVLITTILLLLSLPVLAGAITMLLTDRNLNTTFYDVGGGGDPILYQHLFWFF
GHPEVYILILPGFGLISHMVAQESGKSETFGVLGMIYAMLAIGVLGFVVWAHHMFTVGMD
ADTRAYFTTATMIIAVPTGIKIFSWLGTFHGVRFILSPSLMWALGFVFLFTVGGLTGIVL
SNSSLDVVLHDTYYVVAHFHYVLSMGAVFALMGALINWFPLMVGLTMNPVWTKIQFLLMF
VGVNITFFPMHFLGLAGMPRRYSDYPDTFATWNVIASMGSIVSVISVMGLLFILWEALAS
HRPAISSCHLNTSIEMMHSFPPTAHSYSSVPGVALS*

==

Supplement SD1 Part II: This section lists the COI sequences used in a multi-species artificial "species filter" transcriptome against which short-sequence reads from a sequencing output can be mapped (Bowtie) to assess how many are matched with the COI sequences of represented species. The last three columns give the Bowtie mapping counts of reads mapped onto the transcriptome from three of the transcriptomes we present in the main text that were suspected to have contamination. The low number of cross-specific counts did not support a conclusion of extensive contamination with the target species.

Species	Accession #	COI portion[1]	Np2015	Eb2017	Mp2017
Calanus finmarchicus	MG001885.1	506 - 2062c	0	0	0
Calanus glacialis	MG001883.1	4968 - 6524	0	1	4
Calanus helgolandicus	DN19458_c1_g1_i5[2]	345-1892	0	0	0
Calanus hyperboreus	JX678968	1 - 1546	0	0	0
Calanus marshallae	GJRL01066504+<br>GJRL01051353	1619 - 2308c<br>1 - 695	0	3	5
Calanus pacificus	GJQY01198008	1632 - 3185	0	0	0
Calanus sinicus	GU355641	3660 - 5207	0	0	0
Neocalanus flemingeri	GJSD01085072	1106 - 2656	24	4	3
Neocalanus plumchrus	GJRU01046086	1037 - 2587	536,588	84	0
Neocalanus cristatus	GJRH01042516	1114-2664	0	2	0
Eucalanus bungii	AB091772	c1744-3276	0	831,457	1
Rhincalanus gigas	GIVD01010071	1030-2589	0	0	0
Metridia pacifica	GJAO01037389	7166-8716	0	1	494,531
Paracyclopina nana	EU877959	1 - 1560	0	0	0
Homo sapiens	NC012920	5904 - 7445	--	0	0
Trichoplusia ni	AB158623	1 - 792	--	0	0

[1]The "COI portion" indicates the inclusive base-pair range within the longer mitochondrial sequence with the NCBI accession umber given in Column 2. A "c" at the end indicates incorporation into the artificial transcriptome of the reverse-complement of the .fasta sequence listed under the accession number.

[2]*C. helgolandicus* COI sequence from a contaminant found by Lenz et al 2021 (*Comm. Biol.* **4:**426):

```
>Calhe_COI_LF3_DN19458_c1_g1_i5 (345-1892)
ATAAAAGCCTTCACGGTTAAATGACTATGGTCATGTAATCATAAGGATATTGGCACATTA
TATTTATTGGCCGGTGCGTACTCAGGAATAATCGGTACGGGACTCAGTATAATTATTCGT
CTAGAATTAGGTCAAGCTGGGTCTTTAATTGGAGATGATCAAGTATATAACGTTGTAGTA
ACTGCACACGCATTTATTATAATTTTTTTTATAGTTATGCCTATTTTAATTGGAGGATTT
GGAAACTGATTGGTCCCTTTAATATTGGGTGCAGCAGATATGGCATTTCCTCGTATAAAT
AATATAAGATTCTGGTTCTTAATGCCAGCTTTAATTATACTTTTGTCAAGATCTCTGGTT
GAAAGGGGCGCAGGTACTGGGTGAACCGTGTACCCCCCCCTATCCAGAAATGTAGCCCAT
GCTGGAGCTTCTGTCGACTTTGCTATTTTTTCGTTACATTTAGCTGGGGTGAGATCTATT
TTAGGGGCTGTAAATTTTATTAGAACCCTTGGCAATCTTCGAGTGTTTGGTATATTACTT
GATCGAATGCCTCTTTTTGCCTGGGCTGTTCTAATTACTGCGGTCTTACTTCTCTTATCT
CTCCCTGTTTTGGCCGGGGCAATTACAATACTACTTACAGACCGAAACCTAAATACGACA
TTTTATGATGTAGGGGGCGGAGGAGACCCTATTTTATATCAGCACCTATTTTGATTTTTT
GGCCACCCTGAAGTTTATATTTTAATTTTACCAGGATTTGGACTAATTTCACACATTGTC
GCGCAAGAGAGGGGAAAGAAAGAAACATTCGGAGTTCTTGGTATAATTTATGCCATACTT
GCAATTGGGATTTTAGGCTTTGTTGTATGGGCACACCATATATTTACAGTTGGTATAGAT
GCAGACACTCGAGCATATTTTACAACTGCCACAATGATTATTGCTGTGCCAACAGGAATT
```

Supplement-SD1

AAGATTTTTAGCTGACTAGGAACGTTTCACGGGGTGCGGTTCTCTATGTCCCCCTCACTT
TATTGGGCTCTAGGTTTTGTGTTTTTATTTACCGTGGGGGGCCTGACAGGAATTGTTCTT
TCCAATTCATCCCTGGATGTGGTTCTTCATGACACTTACTATGTTGTAGCCCACTTTCAC
TATGTACTTAGAATAGGGGCTGTTTTTGCACTAATGGCTGCGCTCATTAATTGATTTCCG
TTAATGGTAGGACTAACTATAAACCCTAAGTGGCTGAAGGCTCAGTTTATTATAATGTTT
ATTGGGGTCAATCTTACTTTTTTCCCCATACACTTTCTCGGACTTGCGGGCATACCTCGT
CGGTATTCTGACTACCCTGACAGCTTTATATACTGAAACGTATTTGCCAGGGTTGGATCT
ATTATCTCAATTATAGCCGTCATGTTTTTTTTATTTATTCTGTGGGAGTCTCTGGTGAGC
CATCGTCCAGCTATTTCTAGGCACCACTTAAGAACGTCTATTGAATTTATACACTCTTTC
CCTCCAATGGGCCACAGCTATGCATCTATCCCAGTGTTAAGGGTTTAG

Supplement-SD2. rRNA Contaminants

This document presents representative candidates for contaminants in the 8 transcriptomes of the main text. It is by no means exhaustive. Many sequences belong to potential food taxa. As shown in the main text, no evidence for such contamination was found in the COI sequences of the transcriptomes. As shown here, the situation was different when ribosomal RNAs were examined. However, rRNAs are so well conserved, even in portions across species as widely different as humans and copepods, that a similarity in sequence, especially for short sequences, can give a false signal. Thus the following analysis only produces candidates as exemplars. For more extensive applications of these transcriptomes, a more thorough search for contaminants, and then a testing of other sequences for possible origin, may be a desirable cautious approach.

The table below was constructed by searching NCBI for reliable 18S ribosomal RNA reference sequences for each of the species with transcriptomes of Column 1 (resulting sequence in Column 3). A BLASTn was run with this reference as a query into: 1) that transcriptome to obtain the native sequence(s) most closely matching the rRNA reference piece (indicated under each transcriptome in Column 1) and 2) the NCBI nr/nt database, to search for candidates for foreign contamination (Column 4). MAFFT alignments were used to identify sequences with identities in the low 90% range or below (Column 5, "% ID to native") as candidates for such foreign rRNA. These were reBLASTed into NCBI nr/nt, reporting the hit accession number and species in Columns 6 and 7, respectively, if there was a good match in the high 90% range. The identity (%ID) for foreign candidates from the reBLASTs (if good), as well as the corresponding Bowtie counts mapped to the foreign sequences in the transcriptome are given in Columns 8 and 9 respectively. For comparison, the counts for Bowtie mapping to the native sequences are given in Column 2.

In most cases, the Bowtie mapping rate to the foreign sequence fell well below that of the native (>2 orders of magnitude), suggesting that the contamination was not severe. One notable exception was one of the primate (including *H. sapiens*) sequences, a possible introduction (along with some insect sequences) by the processing facility. Another notable "contaminant" was of the *M. pacifica* transcriptome, traced to a *C. marshallae/C. glacialis* 18S rRNA, but again, attracting relatively low Bowtie counts compared with the native sequence. 18S-contamination was notably absent in the *N. plumchrus* male, which, being non-feeding, was probably less prone to having residues of food items included.

Transcriptome<br>Native rRNA transcript	Count Native	Reference query	Foreign contaminant from BLASTn	% ID to Native.	reBLAST (nr)	Contaminant species	%ID contam	Count contam
Nf2019 GJRT00000000		AF514339 (Nf 18S)	GJRT01000519	87.1%	HQ222525	*uncult. stramenopile*	91.9%	1,135
GJRT01026318	1,367,520							
GJRT01026319	759,441							
Nf2018 GJSD00000000		AF514339 (Nf 18S)	GJSD01004437	77.9%	JF698956	*Chaetoceros sp* 18S	96.4%	6,844
GJSD01042493 18s	2,389,203							--
GJSD01085099 5.8S&28S	3,311,633							--
Np2015 GJRU00000000		AF514340 (Np 18S)	no contaminants		--	--	--	
GJRU01032636 18S+5.8S+38S	137,985							
Nc2017 GJRH00000000		AF514344 (Nc 18S)	GJRH01019373	74.6%	MN197881	*Ulnaria acus*	98.9%	146
GJRH01001704	324,870							
Cm2017 GJRF00000000		MF993123 (Cg 18S)	GJRF01070873	84.9%	MH764816	*Thalassiosira sp*	98.1%	918
GJRF01019735 18S	1,278,330							
GJRF01070873 18S	841,260							
Cm2018 GJRL00000000		MF993123 (Cg 18S)	GJRL01046521	76.7%	HM536166	*Nucleo-cercomonas*	93.5%	74
GJRL01046522 18S	316,976							
Eb2017[1] GJRG00000000		GU969202 (Ee 18S)	GJRG01018095	66.4%	KY962518	*H. sapiens* 18S	99.3%	403
GJRG01024192+	739,683		GJRG01018784[2]	76.7%	"	" 18S	96.6%[2]	32,838
GJRG01018787+	695,073		GJRG01023900	--	"	" 28S[3]	99.7%	155
GJRG01004333	245,790		GJRG01024528	--	"	" 28S[3]	100%	159
			GJRG01018788	78.0%	KX580625	*Lecythium hyalinum*	94.4%	6,559
Mp2017 GJAO00000000		**AB625956** (Mp 18S)	GJAO01028946	93.1%	MF993123	*C. glacialis* 18S	99.5%	(3,158)
GJAO01028956[4]	99,198							

Supplementary Information-SD2

Coverage >98%; E-values =0.0 for all; %ID as reported by NCBI nr/nt reBLASTs of the subject sequence obtained from BLASTing the designated transcriptome with the rRNA query.

Notes:

[1] *E. bungii* has fragmented 18S gene; Matches from 3 non-adjacent pieces totaling 1085 bp of 1805 span used for native entries

[2] *E bungii* GJRG01018784 was almost 100% identical with human 18S rRNA except for the last 82 base pairs, which are quite divergent and presumed to be chimaeric.

[3] These two primate sequences map to different parts of the 28S region of human rRNA

[4] *M. pacifica* GJAO01028956 has a chimaeric 3' end (a fungal sequence) which was removed for comparisons, but could not be separated for the Bowtie counts; the 18S portion was short (1195nt)

Column contents

1 - Transcrptome - Native rRNA: The source transcriptome for the sequences listed below it. Below: "native" sequences with indicated portions of the rRNA against which reads were mapped with Bowtie.
2 - Count Native - Bowtie mapping counts against the sequences in Col. 1
3 - Reference query: The NCBI rRNA sequence used as a query to retrieve the homologous sequence from the Col. 1 transcriptome.
4 - Foreign contaminant from BLASTn - High-scoring hit in a BLASTn of the Reference query (Col 3) into Col. 1 transcriptome.
5 - % ID to native - Percentage of identity in BLAST alignment of the foreign (Col 4) transcript with the native (Col. 1) transcript
6 - ReBLAST (nr) - "Annotation" sequence for the foreign hit (Col. 4) in a BLASTn into NCBI Nucleotide collection (nr/nt) database.
7 - Contaminant species - The species (if identified) for the top reBLAST hit of Col. 6.
8 - %ID contam - Percent identity of the contaminant sequence in the copepod transcriptome with the NCBI rRNA sequence for the species match
9 - Count contam - Number of reads that map to the contaminant sequence (a measure of the level of contamination)

Supplement SD-3: OrthoVenn2 statistics

This document contains summary tables produced by (or from) OrthoVenn2 for the cluster sets of homologs in various combinations of the eight TransDecoder-translated transcriptomes of the main text. Most represent single transcriptomes paired with that of the *N. flemingeri* reference transcriptome ("Nf_ref"). Tables are of the following general format:

Species<br>E cutoff	Cluster count	Protein count	Total proteins in tran.	Total clusters in tran.	Singletons
All overlap					
Sp201xc					
Sp201yc					
Nf_refc					

All overlap row: In the Cluster count cell the number indicates how many homolog clusters had predicted proteins in all of the uploaded transcriptomes (3 in this example). This is the number that appears in the central intersection field of the Venn diagrams. The corresponding number in the Protein count column specifies how many predicted proteins, in all, contributed to those clusters.

Species codes for the different uploaded transcriptomes in the first column are given in the main text (e.g. Table 1). The appended letter "c" indicates that only the TransDecoder complete-proteins were used.

Greyed count cells: These cells enumerate the cluster and protein counts for the component transcriptomes that ***lack*** shared representation (orthologs) among any other of the uploaded transcriptomes (but homologs *within* the same transcriptome - *i.e.* paralogs - are included). These correspond to the numbers in the outer non-overlap fields of the Venn diagrams.

Columns 5 and 4: The rows give the total number of clusters or proteins contained in clusters in the specified transcriptome, whether shared or not. Clusters must include more than a single translated transcript.

Column 6 (*Singletons*): For each transcriptome, these cells give the number of singleton proteins (proteins with no homologs in either the same or other transcriptomes).

Note that the number of clusters that are only partially shared among transcriptomes can be calculated from the total, minus the "all ovelap" minus the number in the grey cell. Partially-shared proteins can be obtained with a similar calculation, including subtraction of singletons. The E-value for homology in OrthoVenn2 was set to 1e-10 for the pairwise runs (unless otherwise specified) and 1e-15 for runs with >2 transcriptomes (indicated in the upper left cell).

Supplementary Information SD3

Blue font indicates conspecific matches

A. *N. flemingeri* Nf2019 vs Nf_ref

Species 1e-10	Cluster count	Protein count	Total proteins in tran.	Total clusters in tran.	Singletons
Overlap	5,530	15,691			
Nf2019c	1,692	5,062	16,599	7,222	3,240
Nf_refc	671	1,923	11,188	6,201	1,871

7,893 (70.1% overlap [shared])

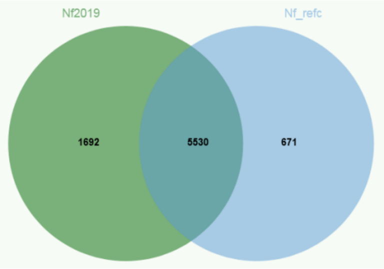

B. *N. flemingeri* stage Nf2018 vs Nf_ref

Species 1e-10	Cluster count	Protein count	Total proteins in tran.	Total clusters in tran.	Singletons
Overlap	5,805	17,425			
Nf2018c	3,012	9,749	23,760	8,817	4,346
Nf_refc	648	1,817	11,188	6,453	1,611

9,465 (61.3% overlap [shared])

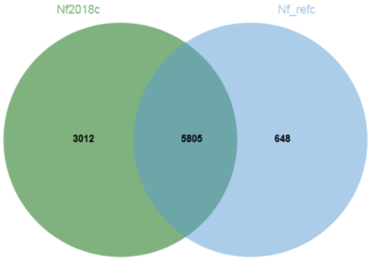

C. *N. plumchrus* Np2015 vs Nf_ref

Species 1e-10	Cluster count	Protein count	Total proteins in tran.	Total clusters in tran.	Singletons
Overlap	5,023	14,409			
Np2015c	2,198	6,617	17,650	7,221	3,639
Nf_refc	850	2,481	11,188	5,873	1,692

8,071 (62.2% overlap [shared])

D. *N. cristatus* stage Nc2017 vs Nf_ref

Species 1e-10	Cluster count	Protein count	Total proteins in tran.	Total clusters in tran.	Singletons
Overlap	5,592	17,409			
Nc2017c	2,798	9,317	23,048	8,390	4,297
Nf_refc	631	1,827	11,188	6,223	1,386

9,021 (62.0% overlap [shared])

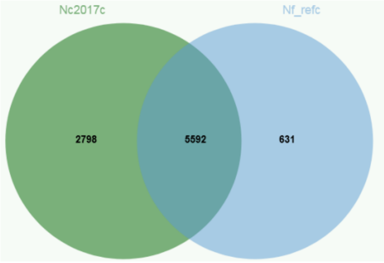

E. *Calanus marshallae* Cm2017 vs Nf_ref

Species 1e-10	Cluster count	Protein count	Total proteins in tran.	Total clusters in tran.	Singletons
Overlap	4,500	15,617			
Cm2017c	1,634	4,973	15,969	6,134	2,611
Nf_refc	769	2,230	11,188	5,269	1,726

6,903 (65.2% overlap [shared])

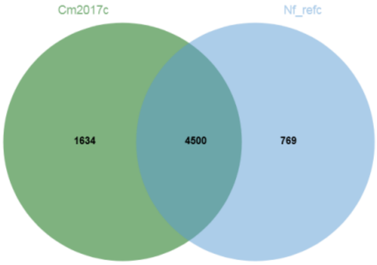

Supplementary Information SD3

F. *Calanus marshallae* Cm2018 vs Nf_ref

Species 1e-10	Cluster count	Protein count	Total clusters in tran.	Total proteins in tran.	Singletons
Overlap	4,964	17,209			
Cm2018c	2,655	9,119	22,391	7,619	3,908
Nf_refc	652	1,911	11,188	5,616	1,432

8,271 (60.0% overlap [shared])

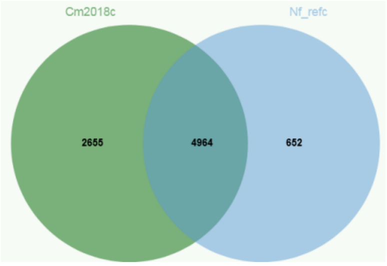

G. *Eucalanus bungii* sEb2017 vs Nf_ref

Species 1e-10	Cluster count	Protein count	Total proteins in tran.	Total clusters in tran.	Singletons
Overlap	2,323	8,122			
Eb2017c	565	1,677	6,825	2,888	1,101
Nf_refc	1,443	4,791	11,188	3,766	2,322

4,331 (53.6% overlap [shared])

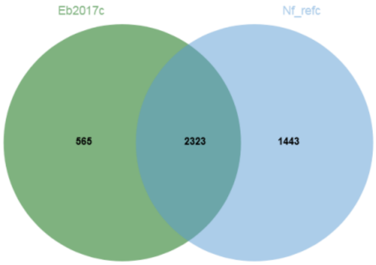

H. *Metridia pacifica* Mp2017 vs Nf_ref

Species 1e-10	Cluster count	Protein count	Total proteins in tran.	Total clusters in tran.	Singletons
Overlap	4,067	19,025			
Mp2017c	5,104	21,644	37,058	9,171	3,314
Nf_refc	858	2,801	11,188	4,925	1,462

10,029 (40.6% overlap [shared])

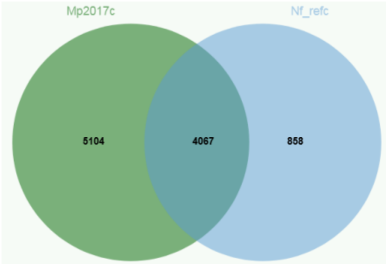

J. 6 calanoid species submitted simultaneously to OrthoVenn

Species 1e-15	Cluster count	Protein count	Total proteins in sp.	Total clusters in sp.	Singletons
Overlap	1387	15,321			
Nf_refc	257	658	11,188	6,663	892 (8%)
Np2015d	686	1187	17650	9,671	1,838 (10%)
Nc2017c	969	2,632	23,048	11,032	2,553 (11%)
Cm2018c	1,077	3,298	22,391	10,111	2,436 (11%)
Eb2017c	255	771	6,825	3,499	559 (8%)
Mp2017c	3,357	13,554	37,058	10,886	2,775 (7%)

# "overlap" primary clusters				
Con-sp.	Con-gen.	Calanid	Myelinate	Calanoid
4,299				
3,562				
1,638				
1,387				

K. *Calanus marshallae:* Cm2017 vs Cm2018

Species 1e-15	Cluster count	Protein count	Total proteins in sp.	Total clusters in sp.	Singletons
Both	7,174	21,208			
Cm2017c	1,016	2,883	15,969	8,190	2,496
Cm2018c	2,263	7,262	22,391	9437	4,511

10,453 (68.6% overlap [shared])

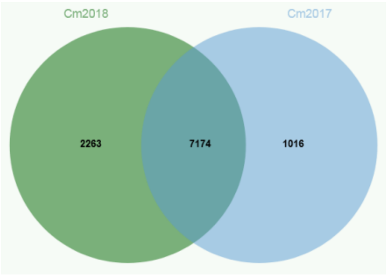

Supplementary Information SD3

L. *Neocalanus flemingeri*, primary Nf_refc, Nf2018c, Nf2019c

Species 1e-15	Cluster count	Protein count	Total proteins in sp.	Total clusters in sp.	Singletons
All ovrlp	4,686	20,789			
Nf_refc	393	1,044	11188	7001	1394
Nf2018c	1,973	5,807	23760	10498	4002
Nf2019c	795	2,174	16599	9034	2364

7,947 (59.7% overlap [shared])

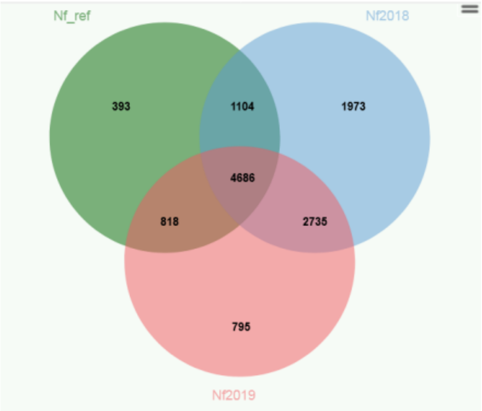

M. Venn diagrams for homologous primary clusters among indicated combinations of transcriptomes

1. *Neocalanus* primary set

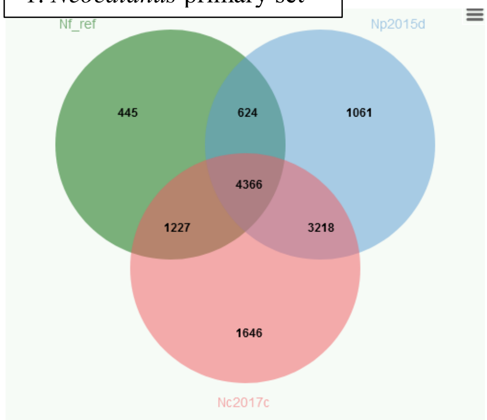

2. Calanidae primary set

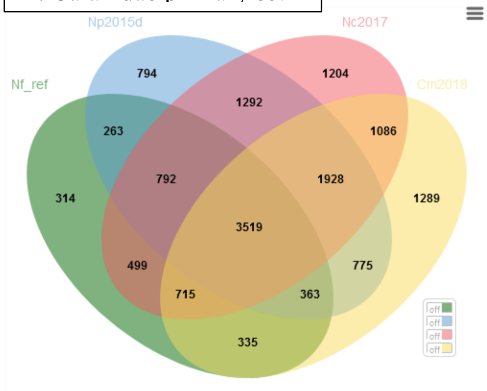

3. Myelinata primary set

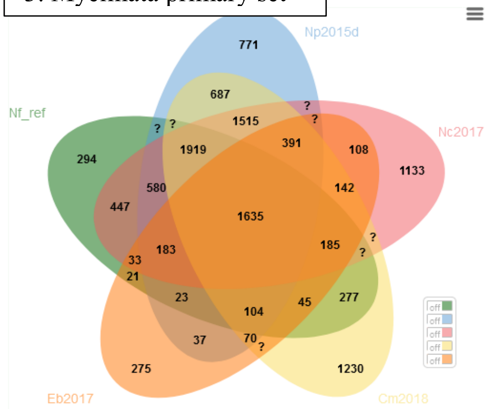

4. Calanoida primary set

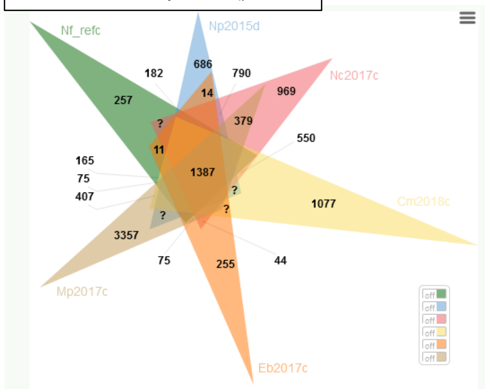

Contributing transcriptomes: Nf_ref+Np2015+Nc2017 (=*Necalanus* set); +Cm2018 (=Calanidae); +Eb2017 (=Myelinata); +Mp2017 (=Calanoida). [see Table 1 for transcriptome codes & NCBI TSA#s]] The central intersection ("all overlap") field in each diagram contains the number listed in Table 4 as "Clusters shared by all." For Panels M1-M3, since the numbers are from primary cluster sets, they are similar but differ somewhat from those of the parallel secondary subsets in SuppFig S1 as a consequence of the way orthologies are constructed.

Supplementary Information SD4

Supplement-SD4: BLASTscans and cladogram data

INDEX

Fig 3 data GHLB01049544 - GST
Fig 4A&5A data - GHLB01033633 Keilin - [Pacifastin-inhibiting motifs]
Fig 4B&5B data - GHLB01008975
Fig 4C&5C data - GHLB01028031 - Ankyrin
SF2A data - GHLB01008164
SF2B data - GHLB01035467
SF2C data - GHLB01048443
SF2D data - GHLB01018689

This supplementary document presents BLAST-scan similarity and top-hit sequence data for

a) the exemplar rapidly-evolving GST omega protein homolog of *N. flemingeri* shown in Fig. 3 of the main text,

b) three exemplar non-annotated differentially-expressed transcripts in *N. flemingeri* used in Fig. 4 as well as for Supplementary figure SF4.

It presents data of three sorts:

1. The tables for each figure-panel below present BLAST-scan results for the *N. flemingeri* query sequence into NCBI data bases, with columns for:
    a. Taxonomic category to which the BLAST results were confined,
    b. The genus name for the species of the hit (see the list above for full species name)
    c. The 2-letter taxonomic category code ("Bar") used to label bars and branches in Figs. 3&4 (see captions)
    d. The values returned by the BLAST for max score, total score, % coverage of the alignment, E-value for the alignment, % identity of the aligned subject, the total nucleotide length of the subject sequence and the NCBI accession number for the hit.
2. Translated amino acid sequences for the proteins represented by the nucleic acid hit.
3. Notes on aberrancies and other points of interest for the BLASTs

Technical note:

TN1: The tBLASTn-scans of Figures 3 and 4 have been utilized as a means of assessing the similarity (measured as $-\log_{10}$(E-value) of the BLAST results) between a reference translated protein sequence from a *Neocalanus flemingeri* transcriptome (Roncalli et al 2019; 2022a) and representative putative homologs in taxonomic categories at different phylogenetic distances from it. The goal has been to locate the distance at which there is an abrupt change in similarity as indicative of the point of emergence of a novel version along the homolog line. This approach has been validated by the observation of a general decline in similarity with phylogenetic distance and the occurrence of such abrupt transitions in certain candidate novel proteins differentially expressed in *N. flemingeri* under different environmental conditions. However cases are noted below in which the decline with distance is not clear cut, and some caveats in the application of the approach need to be kept in mind:

TN2: The approach depends on the integrity of the target transcriptomes in the BLAST-scan. A target transcriptome contaminated with a homologous transcript or parts thereof from a more closely related species than those covered by the target taxonomic category will yield a spuriously high similarity. Several such sources of such contamination have been noted:

a) Misidentification of the species of the transcript submitted to NCBI (all transcriptomes of this paper have been validated through their possession of a single correct COI sequence - see Methods).
b) Inclusion of an incorrectly-identified species of an individual in a transcriptome made from several individuals. In this paper, we often find that the top hit in the Eucalanoiidean superfamily is not from our *Eucalanus bungii* transcriptome (series GJRG01*) but from a *Rhincalanus gigas* one (series GIVD01*) that in addition to a correct COI sequence (GIVD01010071), also has a foreign COI (GIVD01038829) from a co-occurring calanoid (*Calanoides acutus*). Thus we routinely check a separately-assembled uncontaminated *R. gigas* transcriptome ("IT") from publicly-available reads posted by the Stazione Zoologica Anton Dohrn to ascertain that it gets a hit of about the same E-value.
c) Inclusion of RNA from a foreign species accompanying a correctly-identified individual from which a TSA transcriptome is generated. Among the possible cases are cross-species mating (e.g. in the confines of a concentrated net haul) and material in the gut of or attached to the body of a correctly-identified individual. This appeared to be the explanation for an exceptionally good hit on a ctenophore sequence with the *N. flemingery* Fig. 4B sequence query (GHLB01008975): 4e-122 for GHXS01063460. Further investigation revealed that the same transcriptome contained a copepod COI sequence as well (*Calanus pacificus*, probably: GHXS01065124, 99.5% identical to *C. pacificus* COI AF332763)

TN3: A second issue can arise with the tBLASTn method. This translates a protein into a nucleotide sequence which is then compared with a nucleotide sequence in TSA or nr/nt. In so doing, the characteristics of an open reading frame - the ATG methionine start codon and ~3 stop codons are obliterated, so the sequence alignments used by BLAST to assess similarity (E-values and % identity) are dependent only on the nucleotides. Thus occasionally the alignment will contain a stop codon in the middle of an amino acid sequence. If the rest of the alignment is good, it is not always clear how to treat such aberrations, but they make suspect the alignment and the similarity so assessed. The Crustacean hit for Fig. 4B, on *Scylla parmiziana,* was omitted owing to this issue (see *Scylla* outgroup below)

TN4: Frame shifts and chimaerizations usually reduce similarity scores, so sequences lacking such problems will usually give better values. Nonetheless, possibilities for such aberrations in target sequences need to be kept in mind in assessing similarity plots. For example, nucleotides 672-675 missing from the COI sequence of *Calanus marshallae* GJQX01164994 give rise to a predicted partially incorrect truncated partial protein, illustratng the need to confirm both the nuclotide and the prredicted protein sequence of a COI in species identification.

======================== Fig 3 data GST ===================

Data for Figure 3 of main text (GST omega)

Bar	Species	Accession
Nf	*Neocalanus flemingeri*	GHLB01049544
Np	*Neocalanus plumchrus*	GJRU01029434
Nc	*Neocalanus cristatus*	GJRH01070990
Ca	*Calanus hyperboreus*	GJRE01205220
Ca	*Calanus marshallae*	GJRL01056349
Eu	*Rhincalanus gigas*	GIVD01080712
Cn	*Hemidiaptomus amblyodon*	GCIW01017523
Au	*Metridia pacifica*	GJAO01099734
Ha	*Tigriopus japonicus*	EU747055
Cy	*Paracyclopina nana*	KF516611
Cr	*Scylla olivacea*	GDRN01060985
Ar	*Nionia palmeri*	GEKN01052209

tBLASTn scan GHLB01049544

Taxonomic category	Species	Bar	Max score	Total score	Cov.	E-value	% ID	Acc L	Accession
neofl_CV-2015-GAK1-S83R1	DN16733_c0_g1_i4	Nf ☑	526	526	100%	0.0	100.00%	945	GHLB01049544 ☑
nplum-male-2015-R1	TR15069:c0_g1_i1	Np ☑	429	429	100%	2e-151	79.77%	853	GJRU01029434 ☑
n-cris_CV2017_82-S6	DN22217_c0_g1_i1	Nc ☑	499	499	100%	1e-178	93.77%	911	GJRH01070990 ☑
TSA Megacalanoidea	*C. hyperboreus*	Ca ☑	434	434	100%	4e-153	80.93%	863	GJRE01205220 ☑
TSA Calanidae	*C. glacialis*		434	434	100%	9e-153	80.93%	886	HBXE01011775
c-marsh_CV2018_n1-21-S1	DN								GJRL01056349
TSA Eucalanoidea	*R. gigas**	Eu ☑	321	321	98%	2e-108	63.10%	898	GIVD01080712 ☑
ebungii_CV2017	DN19697_c0_g1_i1		334	626	96%	3e-108	63.31%	2080	GJRG01021810
TSA Centropagoidea	*Hemidiaptomus*	Cn ☑	254	254	100%	5e-82	51.35%	925	GCIW01017523 ☑
	Labidocera		240	240	98%	2e-76	48.24%	895	GFWO01186678
TSA Augaptiloidea	*P. xiphias*		202	202	90%	4e-62	45.06%	764	GFCI01445276
mpacifica_AF2017	DN23797_c1_g2_i2	Au ☑	234	234	96%	3e-74	51.21%	849	GJAO01099734 ☑
TSA Harpacticoida	*Tigriopus*		175	175	95%	2e-51	36.18%	753	GCHA01005284
nr/nt	Tigca GST omega	Ha ☑	159	159	95%	4e-48	36.69%	956	EU747055 ☑
TSA Cyclopoida	*Paracyclopina*		148	148	93%	4e-41	34.41%	729	GCJT01030634
nr/nt	GST omega	Cy ☑	147	147	93%	8e-45	34.41%	726	KF516611 ☑
TSA Crustacea - not Cope	*Scylla olivacea*	Cr ☑	187	187	94%	3e-55	41.63%	929	GDRN01060985 ☑
			185	185	93%	2e-53	41.32%	1120	GDRN01060986
	Amphibalanus		180	180	94%	1e-51	41.20%	1092	GHIG01180979
nr/nt Crustacea - not Cope	*Peneus* pyrimidodiazapine syn.		180	180	93%	2e-53	40.50%	868	XM_043020976
	Amphibalanus pyr		172	172	96%	3e-49	39.06%	1233	XM_043386189
	Daphnia GSTω		171	171	97%	4e-49	38.58%	1090	MN730116
TSA Arthropoda not Crust.	*Nionia*	Ar ☑	181	181	94%	5e-52	42.28%	946	GEKN01052209 ☑
	Saluda		183	183	94%	2e-51	42.04%	1292	GDER01027559
nr/nt Arthropoda not Crust	*Halyomorpha* pyr		175	175	95%	2e-49	40.89%	977	XM_024360854.1
	Cephus GSTω		171	171	94%	5e-49	40.00%	729	KX609439.1
Top NCBI nr/nt	*Eurytemora*		231	231	98%	7e-72	48.22%	732	MW149319.1
Uniprot cnfrm. Nf BLAST	GST omega2					1e-49			E9GI93

*R. gigas transcriptome GIVD01 has COI evidence of *a* Calanoides contamination, so is cross-checked with the E bungii GJRG01 sequence and an in-house uncontaminated *R. gigas* sequence.

>**Uniprot_Omega2_tr|E9GI93**|E9GI93_DAPPU **Glutathione S-transferase omega2** isoform a OS=Daphnia pulex OX=6669 GN=DAPPUDRAFT_318250 PE=2 SV=1; **Len=254aa**
MSGVFFPISETKHLAPGSTCPEVTPGLMRLYNMKFCPYAQRTRLVLAAKRIPNEVVNINL
VAKPDWYFARNPLGKVPCLEFDGKVIFESLITCDYLDEVYPSSPMLNSTDPFRKAQDRIL
IEMFNAVNSNLYKLYRCSLDDESTWKGAIVGIHKGLQIFEDDLAKREIQFFGGQNPGIVD
YMIWPWMERLPSLPILSHEMLKVQMENYPSLVKWFDAMKEDDAVKESFISPENHAKFITS
FLAGNPEYDMEINK

>**Nf-GST_GHLB01049544** .1_4 TSA: **Neocalanus flemingeri** TRINITY_DN16733_c0_g1_i4, transcribed; RNA seq. **Len=256aa** 101/254 (38%)ID to Dappu;5'UTR: W*YKQDV*VTDNTVVCRLSTVSPVYITR*IQALRIQT*KVITSK

MSSPIWDVPHLASGAPEPSADPDKVTVYNMRFCPFAERTILVLLAKKLPFDVVNINLKKK
PDWFVEKTWGTVSVVRYKGAYVMESLVNCDFLDELEPSTALHPKDPVEKAMGRLLVEKFG
KMRTPYYGVLLAKGDNPEEVKMKRLEMFNEVKKTLEIMENELKKKETMFFSGGSVGMTDL
MVWPWIERLTTAYRVLFPGENLDIPKEMISLLAWIKNMWEVPAIKAYGLKGDNHAKFYAQ
AASENCDYDMLLTKGA*

>**Np-GST_GJRU01029434** .1_3 TSA: **Neocalanus plumchrus** isolate Monoisolate TR15069:c0_g1_i1, transcribed RNA seq.; **len=255** 5'UTR: Q*WRGLHTTH*ALL*PVRR
MSSPIWNVPHLSAGVPEPSADPDRVTVYNMRFCPFAQRTILVLLSKRIPFDVVNINLKKK
PDWFVEKTWGTVSVVRYKGEYIMESLVNSDFLDELYPSTALHPKDPLDKAMGRLLVEKFA
KMIKPYYGVMTVKGETPEEAKAKRLEKFDELKKTLDCMDKELKKKGTKFFSGGSFGMTDL
MVWPWIERLPEYSVLFPGEKLDIPKEMTSLLAWIRNMWEVPAVKAYGLNGETHAKFHSQY
FSEHCDFDMLLTKGA*

>Nc_GST_GJRH01070990 .1_6 TSA: **Neocalanus cristatus** isolate Monoisolate TRINITY_DN22217_c0_g1_i1, transcribed RNA sequence; 5;UTR: TEHTQTR*ISALRIQT*QIFTSK
MSSPIWDVPHLASGAPEPAADPDKVTVYNMRFCPFAERTILVLLAKKIPFDVVNINLKKK
PNWFVEKTWGTVSVVRYKGEYIMESLVNCDFLDELEPSTTLHPKDPLEKAMGRLLVEKCG
KMRTAYYGVLLAKGDNPEEVKMKRLEMFNEVKKTLEIMEKDLKKKGTMFFSGGSVGMTDL
MVWPWIERLTTAYRVLFPGENLDIPKEMTSLLAWINNMWEVPAVKAYGLKGENHAKFYAQ
AASENCDYDMLLTKGA*

>**Chyp_GST_GJRE01205220** .1_1 TSA: **Calanus hyperboreus** DN17338_c0_g1_i1_Chype_021, transcribed RNA sequence unstopped 5'UTR: PVVTTLVSK
MSSPVWNVPHLAAGDAEPEADPDRVTVYNMRFCPFAERTILVLLTKNIPFDVVNINLKKK
PDWFVEKTWGTVSVVKYKGEYIMESLVNSDFVDELEPATALHSKDPIEKALGRLLVEKFG
KMRPPYYGVIMAKGDTPEEVNKKRMEMWSEVKKTLDCMDKELKKKGSKFFSGGSVGMTDL
MVWPWIERLAVFNVLLPGGYLDIPKEMTSILAWIKNMWEVPAIKAYGLKGETHAKFYKQY
ASENCDYDMLLTKGA*

>**Cm2018_GST_GJRL01056349** .1_4 **DN24989_c0_g9_i1** TSA: **Calanus marshallae** isolate Monoisolate TRINITY_DN24989_c0_g9_i1, transcribed RNA sequence; Unstoppered 5'UTR: QQSVCFPTLASS
MSSLAWDVPHLAAGALEPEADPDRVTVYNMRFCPFAERTILVLLTKNIPFNVVNINLKKK
PDWFIEKTWGTVSVVKYKEEYIMESLVNSDFVDELEPATALHPLDPLEKAKGRLLVEKFG
KMRSPYYGVMLAKGDSPEEVTKKRLEMFNEVKKTLDCMDKELKNKGSKFFSGDSVGMTDL
MVWPWIERLPCYNVLMPGANLDIPAELASLLAWIKNMWEEPAVKAYGLDGETHAKFYMQS
SSDNCNYDMLLN*

>**Rgigas_GST_GIVD01080712** .1_1 TSA: Rhincalanus gigas contig137856.1, transcribed RNA sequence 5'UTR: LQSI*LHPTLLQL*IELQVGNNTVK; 98% align with Rgigas-IT (Clc01)
MSSGKPWNVAHQETGAPEPNVNPDKITVYNMRFCPFAQRTILILLAKKIPFDVVNINLSR
KPEWFVENTWGAVSVVRYKGHHIMESLVNSDFVDELYPETQLHSSDPAEKAKGRLLVEQY
GKMRSSYYPLLRTGSAEERQKLFGEVLKTLELMDSELEKKRTTFYSGDKVGMTDLMVWPW
IERLPNLDIMFSGENLVIPDTMSSLKRWISSMWEVPAVADYGLTPNQHQEFYKGYISGSP
DYDALLTGGA*

>**Eb_GST_GJRG01021810** .1_5 TSA: **Eucalanus bungii** isolate Monoisolate TRINITY_DN19697_c0_g1_i1, transcribed RNA sequence; **note YDMLL motif**; in contrast, BLAST alignment in yellow and MAFFT in grey & yellow; 166/250 (66%) ID to Rgigas
LASTVSLTPTTEKTAPVHLKKLSVNMSQYTKKPWDVPHLETGAPEPSVDPNRITVYNMRF
CPFAERTMLVLLAKNLSFDVVNINLVKKPEWFKEKTWGVVSVVRYKGDYIMESLVNSDFV
DELYPETSLHPADPFEKANGRLLVEKFGKIGGLFFTCMRASKETPEETAKERQRIFSDMR
KIFESMDKELAKNKPSTTAASCMPAMMARVNGGYTGSLHGDFISGSKAGMTDLMLWPFFE
RIPSLDVMYPGEGLGIPEEFKNLTAWVSAMKKTPAVTAYGLPSEQHAQFYKTYASGPSYD
MLLPAAEKALSIKKPWDVPHLATGSTKPSVDQDRITVYNMRFCPFAQRTMLVLLAKNLPF

DVVNINLSRKPEWFVENTWGTVSVVRYKNNYIMESLVNCDFIDQLHPNTSLHPVDPAEKA
KGRLLVELYGKIRTSYYPCLRVSKETKEETVKERQRLFQEIKKTLETMERELAERKTTFF
SGQQVGMADLMVWPWMERLPVLDVMFPGEGLVIPGELKSLKKWITAMWAVPAVAVYGIEP
EDHVKFYAGYASGNPPYDMLISS*

>**Cn_GST_GCIW01017523** .1_2 TSA: **Hemidiaptomus amblyodon** strain wildtype C182822_a_21_0_l_925, transcribed RNA sequence; lots of Met's in this and Eb
LIHFYKRKRMTGQPWDIPHLAAGSSAPVIDPEKVTVYNMRFCPFAERTMLVLLAKNIPFE
VVNINLKKKPDWFTEKTFGLVSVVLYKGDFIMESLINCDFLDEEFPTLRLHPVDPAQKAK
DRLVVEIFNKMMGPFYKSFMAKTAEERTSTYKEVVVVLEKIEEELKKRGTKYFCGEEPGM
LDYMIWPWFERFAANSLMFPELALPAHLTSLQDWEKAMWTTGPVSQYGLPPETLVRFTQE
YMNKPDNPDYDFLLKESK*

>**Mp_GST_GJAO01099734** .1_3 **DN23797_c1_g2_i2** TSA: **Metridia pacifica** isolate Monoisolate TRINITY_DN23797_c1_g2_i2, transcribed RNA seq. stopperred 5'UTR: KLHCLSITL*KHRECPDPPPVS*NPFS
MAHWDSTPHLSQGSPAPIINPDIMTVYNMRFCPFAERTILVLLAKELPFQVININLTNKP
DWFLEQTAGKVPVLLHKGQVILESLVTCNYLDENFPSIPLHPKDERRKANDVSLVESFTG
SLASIYKVSLATSPEDRLENFKEVVKMLQEINSVLKIRGGTFIGGESPAMSDLMIWPWME
RIAAFPHMFPGEDLIPPSNLENLIQWIGAMLEVPAVKAYRVNPEDMAKFYSTVKRDYDMF
LKYK*

>**Ha_GST_GCHA01005284**.1_1 TSA: **Tigriopus japonicus** TJ_CDS_05285 transcribed RNA sequence; 250 aa; non-stoppered but maybe OK -
MNGINTKHFETGSKCPSIDPEKLTVYNMRYCPFAQRTILVLLKKNIPFDTINVSLSKKPE
WFLERNPLGKVPTIQIGDKIIYESIITVDYIDQVYGGETLNQKDPYQDALDRMLIERFSQ
ALPAHYRLYYGQPSEVTPEQRKAWSDEMLEKLHVLEEALGERQTPYFGGDSVKMVDYMIW
PWFERILAMPAFAPECALDPKRFARVVKWIDLMEQDSAVKAYRISSEDFLEFRRSKLEGN
TNYNIIADRQ*

>**Tigja_EU747055 Tigriopus japonicus GSTomega annotated**
L=280
MALSTRLGLFLGAQNTLSGSLRLKMSSWSPSLLNNMNSRHLGANDPMPPINPDVYTVFNL
RFCPYAQRTILFLLAKQIPFENVNIDLKNKPGWFLAINPLGKVPTLVRGSAVIYESLICD
DFLEEEHPETKRLLRDTPLERAMDKLLVERSKFFYGPLHGLMKVKTQEDLEKVKGQVQGG
IQILGDELRSRGTDFFGGSMPGMVDYSIWPWFERFNAFAQLSKIDLNDPTLQSWMSKMFE
DEAVRYYRIEDNDHTEFWRGFAQGQQNYDILYQKNYSQKL*

>**Cy_GST_GCJT01030634** .1_1 TSA: **Paracyclopina nana** Pnana_mRNA_33695 transcribed RNA sequence [lots more of this length]
MVRWSKHLDSSDECPKIDKNKLTLFNMRYCPYAQRTVLALEAKKIPYDMINVNLKDRPQW
FLNKNPMGKVPTIQVGDEIFYESHPVNDYLDEVFPGRKLNPAKAEERAKDRMFLAHYDNA
IGLFYKIILSESDREENIKKFNEKMEFFEKELAKRESKFLSKENEPGMLDYMTWPWMERV
EIIPSLFSDLPEILPTSSFPRINAWIKAMKEDPVVHGYILTLEEHTAFFKSYMEQSPNYD
PQ*

>**Paracy_KF516611 Paracyclopina nana GSTomega annotated** L=242
MVRWSKHLDSSDECPKIDKNKLTLFNMRYCPYAQRTVLALEAKKIPYDMINVNLKDRPQW
FLNKNPMGKVPTIQVGDEIFYESHPVNDYLDEVFPGRKLNPAKAEERAKDRMFLAHYDNA
IGLFYKIILSESDREENIKKFNEKMEFFEKELAKRESKFLSKENEPGMLDYMTWPWMERV
EIIPSLFSDLPEILPTSSFPRINAWIKAMKEDPVVHGYILTLEEHTAFFKSYMEQSPNYD
PQ*

>**Cr_GST_GDRN01060985** .1_5 TSA: **Scylla olivacea** Ref_Crab_Transcript_61140_929 transcribed RNA sequence; 5'UTR: LPSLSPIPHHHYRHPATTPPVTICQSLQGCIERRSVQRPASA
MSLKHLATGSTCPPAVAGLLRCYNMRFCPFAQRACIILAAKNVKHEIVNINLKKKPEWF

FEKSPLGKVPSIELDGQIMSESLVICDYLDEVYPDPPLHPTDPWRKGQDRMFLEIFGKV
TGAMYKVYFSRGDQDILSKGFTDIQAGLDPFEAELTKRNTKFFGGDKPGMLDYMIWPWM
ERLPVAQKFGGERALPENCYPKLFSWMEDMKQDPAVKATYLSPETHYKYLLTSISGSPD
YDMQLTMPCHL*

>**Cr_GST_GDRN01060986**.1_4 TSA: **Scylla olivacea** Ref_Crab_Transcript_61141_1120 transcribed RNA sequence; 6' UTR: LPSLSPIPHHHYRHPATTPPVTICQSLQGCIERRSVQRPASA
MSLKHLATGSTCPPAVAGLLRCYNMRFCPFAQRACIILAAKNVKHEIVNINLKKKPEWFF
EKSPLGKVPSIELDGQIMSESLVICDYLDEVYPDPPLHPTDPWRKGQDRMFLEIFGKVTG
AMYKVYFSRGDQDILSKGFTDIQAGLDPFEAELTKRNTKFFGGDKPGMLDYMIWPWMERL
PVAQKFGGERALPENCYPKLFSWMEDMKQDPAVKATYLSPETHYKYLLTSISGSPDYDMQ
V*

>**Cr_GST_GHIG01180979** .1_3 TSA: **Amphibalanus improvisus** c227519_g1_i2, transcribed RNA sequence; 5'UTR: SDVCSSDRERRARKARNTAEQNVIRDN
MGVTSETKHLSAGSECPPLQPGTLRCYSMKFCPYAQRTRLVLAAKGVPHEIVNINLKTKP
EWYLAKSATGGKVPALELDGNYVPESLITSDLLEELYPEPALYPKDPWRKAQDRLLVEVF
GGSISTFYQMYRAFGDADKIRELSAEHAKKIQFFEKELIKRGTPYFFGDRPGMVDLMIWP
WVERFPAARKMGVTSLPQGEMPPPPTEIKYFHEWVMRMAQNPIVKSCLTSTEDHMKFIKG
FTSGNPDYDMELSSV*

>**Ar_GST_Nion_GEKN01052209 Nionia palmeri** GSTomega L=238
MPQHLGQGSTEPPLSPGKVRLYGMRFCPYDHRVHLALLAKKIPHDCVWIDLRNKPDWYVK
KIPSTRVPGLFIDGEYLYESLIICDFLDEKFPDRPLHSRDPLSKAKDRILLENFGKVSTA
YYKVVNNPTPSTQDFDIVVKELVELDKELDGRGSKFFGGGEPGMLDYMIWPWFERLSAGK
ITFGDDFDLPKDKLKHLALWMKAMEEDDVVKEYYLRPEIYSPFLKGYATGTADYNTPV*

=========================== Fig 4A&5A data ======= index =========

Data for Figure 4A of main text: Keilin (Pacifastin-inhibition) - GHLB01033633 [old Fig 2C]

Bar	Species	Accession
Nf	Neocalanus flemingeri	GHLB01033633
Np	Neocalanus plumchrus	GJRU01069908
Nc	Neocalanus cristatus	GJRH01072522
Ca	Calanus marshallae	GJQX01129624
Eu	Rhincalanus gigas	GIVD01076962
Cn	Labidocera madurae	GFWO01143977
Au	Metridia pacifica	GJAO01102519
Cr	Eogammarus possjeticus	GGQT01075462

tBLASTn-scan GHLB01033633.1 Fig 4A&5A data

Top hit	Species	Bar	Max score	Total Score	Cov.	E-value	% ID	Acc L	Accession
neofl_CV-2015-GAK1	Nf_rf DN8009	Nf	540	540	100%	1e-200	95.31%	1065	GHLB01033633.1
nplum-male-2015-R1	Np TR35732	Np	526	526	100%	1e-200	92.06%	1094	GJRU01069908.1
n-cris_CV2017_82-S6	Nc DN184	Nc	502	502	100%	7e-179	88.45%	1014	GJRH01072522.1
TSA Megacalanoidea	*C. hyperbor.*		471	471	100%	7e-167	82.31%	966	GJRE01211717.1
c-marsh	Cmar007	Ca	459	459	100%	4e-161	79.06%	1098	GJQX01129624.1
TSA Eucalanoidea	*R. gigas*	Eu	249	249	83%	1e-79	51.07%	938	GIVD01076962.1
Rgigas-IT	Rgigas-IT					1e-78	55%		DN12767_c0_g1_i1
ebungii_CV2017	Eb DN13382*		94.0	216	80%	2e-21	45.63%	385	GJRG01033830.1

TSA Centropagoidea	*L. madurae**	Cn	225	295	91%	1e-67	47.62%	1570	GFWO01143977.1
TSA Augaptiloidea	*P. xiphias**		155	155	88%	1e-42	38.04%	973	GFCI01106192.1
mpacifica_AF2017	Mp DN17111*	Au	165	239	93%	5e-47	39.54%	892	GJAO01102519.1
TSA Harpacticoida	no signif		0			1			
TSA Cyclopoida	no signif		0			1			
TSA Crustacea - not Cope.	*Eogammarus*	Cr	68.2	120†	71%	1e-09	32.54%	9560	GGQT01075462.1
TSA Arthropoda not Crust.	*Dasyhelea* ‡		79.0	282	81%	8e-10	31.36%	3830	GGAN01006129.1
Top NCBI nr/nt	*E. affinis* kielin/chordin‡		223	305	91%	2e-68	46.46%	1256	XM_023490416.1

>**Nf_ref_**GHLB01033633_**DN8009_c0_g1_i1**.p1 _~~TRINITY_DN8009_c0_g1_i1.p1 ORF type:complete len:278 (+),score=62.67 TRINITY_DN8009_c0_g1_i1:184-1017(+) **6-Cys-sequences = Pacifastin by motifs & SwissProt reBLAST**
MRQYLVLFSLAAASLVAESQENNAVIDACKDVDGTRHKLGDSYIGPDACNKCKCLESGSA
CTKRLCPEDISSRNAEAFKCVDNLGVLHEVNETYTHVDGCNSCKCGKHGGACTRKFCLKK
EKKTLSECVDGDGNNKALDEAWLDKDGCNKCVCGILGAVCTEMFCGEHRMYENEADQVKV
HELIHTDEGTIVDESRDQSCKDDDDNTQWSGSSWLSKDSCNICTCPGNGTSPICTQMGCR
VRLERLLQSNLTDGAAGTAHSLATLVFSVAVVMAAYL

>**Np_**GJRU01069908_**TR35732_c0_g1_i2**.p1 GENE.TR35732_c0_g1_i2~~TR35732_c0_g1_i2.p1 ORF type:complete len:278 (+),score=54.68 TR35732_c0_g1_i2:177-1010(+)
MRQYLVLFSLAAASLVAESQENNAVIDACKDVDGTRHKLGDSYIGPDACNKCKCLESGSA
CTKRLCPEDISSRNAEAFKCVDNLGVLHDVNQTYTHVDGCNSCKCGKHGGACTRKFCLKK
EEKTLSECVDGDGNNKALDEAWLDKDGCNKCVCGILGTVCTEMFCGEHRMYENEADQVKV
HELIHTDEGTIVDESGDQFCKDDDDNTQWSGSSWLSKDSCNICTCPGNGTSPICTQMGCR
VRLERLLQSNLTDGSAGTAHSLATLMLSVAVVMAAYL*

>**Nc_**GJRH01072522_**DN184_c0_g1_i1**.p1 TRINITY_DN184_c0_g1~~TRINITY_DN184_c0_g1_i1.p1 ORF type:complete len:278 (-),score=70.41 TRINITY_DN184_c0_g1_i1:75-908(-)
MRQYLVLFSLAAASLVAESQENNAVIDACKDVDGTRHKLGDSYIGPDACNKCKCLESGSA
CTKRLCPEDKTSRNAEAFKCVDNLGVLHEVNQTYTHVDGCNSCKCRKYGGACTRKFCLIK
EEKTLSECVDGDGNNKALDEAWLDKDGCNKCVCGILGTVCTEMFCGEHRMYENEADQVKV
HELIHTDEGTIVDESGDQPCKDDDDKTQWPGSSWLSKDSCNICTCPGNGTSPICTMMGCR
VRVERLLQSNLTDGAAVTAHSLVTLMLSVAVVIAAYL*

>**Cm_GJQX01129624** TSA: **Calanus marshallae** DN14359_c0_g1_i1_Cmar_007; Len=277aa
MRQYLFIVVFAALSLAEDSPDNNQVVDACKDVDGTLHKLGDSYIGPDACNKCKCLKSGSA
CTKRLCPEDSSSRSAEAFKCVDNMGVLHEVNQSYTHVDGCNTCKCMEHGGACTRKFCLKK
EERTLSSCVDADGNAKLLDEAWLDKDGCNKCVCGILGAVCTEMFCGEHRMYENEADQVKV
HEVIHTDQGDIVDESGDQPCKDEDDNDQWPGNSWLSKDSCNICTCPGNGTSPICTQMGCR
VRLERLLQSNLTGGVALKTNNLVTILLIVSVVFAAFL*

>**Eu_GIVD01076962** _2 TSA: Rhincalanus gigas cntg131293 - MAFFT homologous region in yellow
MTMLGLIMAVILVSSQVKGEDEADQSGVLSHCVDVDGTKHRLAEAYIGQDGCNRCKCQEKGEG
CTKKLCPTDVDSRAAEAFKCVDNMGVLHEENETYTHVDGCNTCTCMGDKGGSCTRRFCFA
KKDKSLSGCVDHDGIKREPSTEWQHKDGCNKCICGVLGPICTMSYCTHDTYWEPEVPKKE
ASPVVEKSPSEATTDSKPCKDEEGNDVSLGGVWLTGDNCNICQCKGNVSEPLCSNVGCRV
RLERLVQSDPSLTGLEPGQHEDHKNGSAYINGX

>**Cn_GFWO01143977** _2 r/c TSA: Labidocera madurae TR60262:c1_g1_i2
TKLILLDSL
LQEIKMFVIFALLLVFSGTSLSQELRESCVDVQGVKHKLHDSYIGADGCNKCKCLEGGSA

CTKKFCPPGTPRIAAEANKCIDNQGNLHADGESYTHVDGCNTCICRPFGGSCTRKFCLKE
LKESSLCLDPQGNPRKEGESWLAQDQCNKCQCGVMGPVCTKTKCISKDPPKEPRDGEIVD
ETGDSPCRLEDGSTKFPGDSWLTADSCNICTCTGMNGNIDCTAEGCRVRFSRLMDPQKKE
DGGEGSSGTKMTASLALAISFSALLL*

>Au_GJAO01102519 _3 r/c TSA: Metridia pacifica DN17111_c0_g1_i1
KLILLDSLLQENNTEIM
LSPFFLFLCLTACSLGEELSNVGGSYCFDGNGKDHAVGEQYSPDGCNTCTCMQIDGGEPK
ERCTRMYCGNPTIRKPEEEEKLANKCVDNMGKIHQVGHTYTHVDGCNECRCMSFGGACTR
KFCLPFNKNKKCYDKEGKERSEADKWTQGSCNKCVCGTFGAVCTERCDIVDESGDQPCRD
YNGTSKMPGDQWLSPDNCNTCICKGDGSMPICTRMACGARLAKLLKSASPLTGGNNGVGT
LKLNLTIMSMLVCSIVAIIL*

>Cr_GGQT01075462 r/c TSA: Eogammarus possjeticus Cluster-13637.44399; **17 Pacifastin-inhibiting motifs!! C-[9-12]x-CNWC[R/S/T]C-[7-8x]-CTRRxC**
LEAVACLRREAGVPHRTMKA
VLVGVVLGLGLLSLATTAQGGAVGAEAQCTEGSQWHHQCNKCWCIEGLPACTKMGCAGGR
FNIVDDSCKDGSRWLKDDCNWCSCIDGAAVCTHRACLPDWARGRSLEATDDEQAVTRSGG
DAAAGRSSSAPVCEPATTNDRWRDGCNWCRCSETGVGMCTRRGCPQAIVQRLQGTKECEG
SPRWRRDCNWCTCSDDGFADCTDDVCPSKPDLQPLPASTTSLRSGAAAAPAAGAGDTEAT
CRVTENGDRWREDCNWCRCLEGKSACTRRGCPPALRARLEGTQECEGEAQWKHGANWCTC
SNERAVCTGRAQINREDYLQSNSIQDSAYEPECTDGSAWTESCNSCRCNNGASTCTRRGC
LRVEADTCVEGSTWKTDACNWCTCSASGVPACTRRACVGDSTISARSSLAVSAAPVAATL
SAIDEGASCIDGSRWKKDGCNWCSCVGGRGVCTRRACLPADESRTPSLQCVEGSSWSDGC
NNCHCSNSLAVCTKRACVNKVTIPDRRGLAVAPDMSRPQVQDRPRRPISIPRDDRDCVPG
SRFSRDGCNWCQCGNNGVAACTIMACFKKIGDDEKMCEEGSRWKQDCNWCNCVDGRGVCT
ELACLPAMLGLSPAPETEGQTKQEPELAPDAECSEGSRWREDCNWCSCQNGRGVCTRRAC
IPGRSSDADTEPQCVAGSRWKADCNWCQCSDTGIGLCTLKACVPSRASTAAAASDPQPIA
VVGTGRSLSGGSSTLRGECKPGSVFRQRCNRCRCSDQGLKLCTKRLCNPRADYSSEPQCE
GDAVFKDEQNCNWCHCHNGIAACTLKLCFDVTHRPRALGGGSARSLSAAADDSTQQTAQV
PEGATEECTEGTSWVEDCNSCQCRNGLKTCTRRLCLTQQVRPQASDDAAAAADAAAEPDD
TSRCQKPVDSGPCFASFRMFRFNPDTNRCEAFLYGGCAGNDNRFATPELCQQACGGAAPV
LDTTCDRTRCPEMRELDYYSAKGCTPVYDAGRCCATSFFCTETRSEIASPGQCLYQGTVY
NSGDSVPVLDNCSRCRCGGGRIHCASIECPSLFRGHQPGCRGLYRPGECCEYDQECDAPP
NPLEPSSPAVTRDAVSCEAEGKSYREADQMYFDHSPCQKCVCGPDYTGPFGAGCQKINCG
FDFRYQDRIDDGCVPLFYEERCCPIGFICPGDTEIAEDQSQANPDGVTENVCTFNGTTVA
VGRELATTEENKKCSCKTPPDVTCVQTDD*

OUTGROUP tBLASTn alignment

This alignment gives a visualization of how the tBLASTn scan selected the subject sequence as its top hit in the Crustacea-but-not-Copepoda taxon. This protein, with only 33% identity and an E-value >1e-10, seems distant as a homolog of the *N. flemingeri* query, but 40/75 (53%) identity in the 4 Pacifastin-inhibiting motifs (underlined), which strengthens the case for homology:

Query: Nf_ref_DN8009_c0_g1_i1
TSA: Eogammarus possjeticus Cluster-13637.44399 transcribed RNA sequence ProteinLink
Sequence ID: GGQT01075462.1 Length: 9560 Number of Matches: 2 Range 1: 7669 to 8250

Score	Expect	Method	Identities	Positives	Gaps	Frame
68.2 bits(165)	1e-09	Compositional matrix adjust.	68/209(33%)	90/209(43%)	27/209(12%)	-3

```
Query   47     DACNKCKCLESG-SACTKRLCPED--ISSRNAEAFKCVDNLGVLHEVNETYTHVDG----  99
               DACN C C  SG  ACT+R C  D  IS+R++ A        L  ++E  + +DG
Sbjct   8250   DACNWCTCSASGVPACTRRACVGDSTISARSSLAVSAAPVAATLSAIDEGASCIDGSRWK  8071
```

Supplementary Information SD4

```
Query  100   ---CNSCKCGKHGGACTRKFCLKK-EKKTLS-ECVDGDGNNKALDEAWLDKDGCNKCVCG  154
                CN C C    G CTR+ CL   E +T S +CV+G         +W   DGCN C C
Sbjct  8070  KDGCNWCSCVGGRGVCTRRACLPADESRTPSLQCVEG--------SSW--SDGCNNCHCS  7921

Query  155   ILGAVCTEMFCGEHRMYENEADQVKVHELIHTDEGTIVDESRDQSCKDDDDNTQWSGSSW  214
                AVCT+  C       +        ++       + D  R       DD      GS
Sbjct  7920  NSLAVCTKRACVNKVTIPDRRGLAVAPDMSRPQ---VQDRPRRPISIPRDDRDCVPGSR-  7753

Query  215   LSKDSCNICTCPGNGTSPICTQMGCRVRL  243
              S+D CN C C  NG +  CT M C  ++
Sbjct  7752  FSRDGCNWCQCGNNGVA-ACTIMACFKKI  7669
```

NOTES:

1. Motifs: Protein GHLB01008975 was characterized by various numbers of Pacifastin motifs:

Calanoid: 4 motifs: C-K/V*-D-17x-CNxCxCTCT-3x-C [motifs 1 & 4=K; 2&3=V]

Crustacea: 17 motifs: C-[9-12]x-CNWC[R/S/T]C-[7-8]x-CTRRxC

========================== **Fig4B&5B data** ====== index ==========

Data for Figure 4B of main text (Protein GHLB01008975)

Bar	Species	Accession
Nf	*Neocalanus flemingeri*	GHLB01008975
Np	*Neocalanus plumchrus*	GJRU01029534
Nc	*Neocalanus cristatus*	GJRH01050899
Ca	*Calanus hyperboreus*	GJRE01167337
Eu	*Rhincalanus giga*	GIVD01061381
Cn	*Temora stylifera*	GJGX01080229
Ha	*Platychelipus littoralis*	GHXK01214142
Cy	*Apocyclops royi*	GHAJ01003083
Out	*Aplysina aerophoba*	HBWR010030842 (sponge)

BLAST-scan (tBLASTn primary Nf-ref sequence into focused TSA ROI).

Cala emergence

Top hit	Species	Bar	Max score	Total score	Cov.	E-value	% ID	Acc L	Accession
neofl_CV-2015-GAK1-S83R1	Nf DN13923	Nf	559	559	100%	1e-200	95.89%	1000	GHLB01008975
nplum-male-2015-R1	Np TR19104	Np	542	542	99%	1e-200	92.78%	1288	GJRU01029534
n-cris_CV2017_82-S6	Nc DN21324	Nc	531	531	100%	1e-200	90.75%	1153	GJRH01050899
TSA Megacalanoidea	*C. hyperbor*	Ca	474	474	100%	3e-167	79.45%	1033	GJRE01167337
Cmar_005	DN27030_c0_g1_i1		440	440	94%	1e-154	76.62%	906	GJQX01057268
c-marsh_CV2017_n2-62-S4*	DN22196_c0_g1_i1		439	439	94%	4e-154☑	76.62%	942	GJRF01022292
Cmar_007	DN14729_c0_g1_i1		434	434	95%	4e-152	75.90%	898	GJQX01101247
TSA Eucalanoidea	*R. gigas*		61.6	61.6	29%	2e-09	30.23%	348	GIVD01181919
		Eu	62.0	62.0	61%†	3e-08	29.90%	1130	GIVD01061381†
			53.5	53.5	53%	2e-05	26.63%	1191	GIVD01057278
Rgigas-IT	Rgigas-IT					6e-08☑	27%		DN15580_c1_g1_i4
ebungii_CV2017	no hits								

TSA Centropagoidea	*Temora sp.* ‡	Cn	69.3<br>66.6	69.3<br>66.6	48%<br>60%	1e-11<br>9e-10	30.46%<br>26.49%	497<br>1080	GINW01341388.1<br>GJGX01080229.1
TSA Augaptiloidea	no signif								
mpacifica_AF2017	no signif					1			
TSA Harpacticoida	*Platychelipus*	Ha	79.3	79.3	59%	7e-14	24.62%	1389	GHXK01214142.1
TSA Cyclopoida	*Apocyclops*	Cy	79.3	79.3	89%	9e-14	23.26%	2216	GHAJ01003083.1
TSA Crustacea - not Cope.	*Scylla*		101	101	66%	5e-22	30.77%	827	GEUT01004470.1
Hit 2 (BLAST alignments)	*paramamosain*[1]		100	100	67%	7e-22	30.15%	823	GEUT01009610.1
Hit 3			102	102	67%	8e-22	27.00%	1119	GIXE01050736.1
Hit 4; consensus			96.7	96.7	67%	2e-20	26.26%	811	GIXE01070519.1
Hit 5			96.7	96.7	67%	3e-20	29.65%	897	GIXE01063667.1
Hit 6			94.7	94.7	67%	2e-19	25.50%	959	GEUT01011635.1
TSA Arthropoda not Crust.	no signif		0						
nr/nt Arthropoda not Crust.	no signif								
Top NCBI nr/nt	*Lepeophtheirus unchar.*		80.1	80.1	81%	2e-12	22.30%	2181	XM_040710225.1
Sole protostome not Cr	Barentsia		73.9	73.9	68%	2e-11	23.30%	691	GIMW01066412.1
TSA, nr/nt Deutero.	no hits								
TSA Animalia/not Nephrozoa	*Hormiphora calif.*[2]		356	356	68%	4e-122[2]	84.08%	672	GHXS01063460.1
	Hormiphora calif		166	166	32%	7e-49[2]	80.85%	380	GHXS01063461.1
	Symsagitifera[3]		81.3	81.3	46%	3e-14	33.10%	1211	GFRZ01167984.1
	Aplysina[4]	Out			52%	2e-09	25.00%	900	HBWR010030842.1

* This is the non-standard Cm transcriptome [also lower-sim Cm2017]

†Better fit based on presence of highly similar sequence in in R. gigas-IT transcriptome that 1919 is lacking

‡ Top 2 hits were too short, so the third was used

[1] *Scylla* sequence does not translate into any significant protein nor do any of the other 7 *Scylla* hits (no other crustacean hits). Translation frame 1 does a reasonable MAFFT fit with few gaps, but still has lots of stop codons. Strange sequence, should omit. See tBLASTn alignments below

[2] *Hormiphora*: Comb-jellies prey on copepods: copepod seq. contaminated the comb jelly transcriptome. SeeTechnical Note 2c above

[3] *Symsagitta* - "Mint sauce worm" a platyhelminth with symbiotic green *Tetraselmis*. - not a good ooutgroup

[4] *Aplysina aerophoba* (sponge) - the best outgroup found

>Nf-ref_GHLB01008975_DN13923_c0_g1_i1 Neocalanus flemingeri NCBI nr/nt: Lepeophtheirus unchar.; cov 81%, 2e-12, 22.3%ID,AccL 2181, XM_040710225
MRFKIFILVSLLLHLDVAFGGLPLSTFNMTLAQSERLRNGNDESFNLAKDIMNDALKNYV
KEGNLDIVIENNDIVIDAAFDDRVLKDGCRVKLFALHPRARGTIKRSSQLMADIYDGDFS
LGELSAAAKADLDVELDLNFDFRAQIGAKIFGKCRKIGRDTLGIDLKTSGKAILAVSLEG
TGVTIDPNLENIRFRLNLNIMGRLENWNVDDIDVSKCQVKLFNRVEIGSYCSVAKNLIKK
ALQGYINKWTKFQAPRLIEKLERKLQSRIGEEIVIPLNFNMEDDLGDEDIFA*

>Np2015_GJRU01029534_TR15104_c0_g1_i5.p1 GENE.TR15104_c0_g1_i5
MRFRIFILVSLLLHFDVAFGGLPLSSFNMTLAQSERLRNGNDESFNLAKDIMNDALKNYV
KEGNLDIVIENNDIVIDAAFDDRVLKDGCRVKLFALHPRARGTIKRSSKLMADIYDGDFS
LGELSAAAKADLDVELDLNFDFRAQIGAKIFGKCRKIGRDTLGIDLKTSGKAILAVSLEG
TGVTIDPNLENIRFRLNLNIMGRLENWNVDDIDVSKCQVKLFNRVEIGSYCSVAKNLIQK
ALQGYINKWTTFQAPRLIEKLERKLQSRIGEEIVIPLNFNMDDDLENEDIFD*

>**Nc2017_GJRH01050899 DN21324_c0_g1_i1**
MRFKIFILVSLLLDFDAVFGGLPLSTFNMTLEQSERLRNGNDESISLAKDIINDALKNYV
KEGNLDIVIENNDIVIDATFDDRVLKDGCRVKLFALHPRARGTIKRSSKLMADIYDGDFS
LGEFSAAAKADLDVQLDLNFDFRTQIGAKIFGKCRKIGRDTLGIDLKTSGKAILAVSLEG
TGVTIDPNLENIRFRLNLNIMGRLENWNVDDIDVSKCQVKLFNRVEIGSYCSVAKNLIKN
ALQGYINKWTKFQAPRLIEKLERKLQSRIGEEIVIPLNFNMEDDLGDEDIFP*

>**Chype_GJRE01167337 Calanus hyperboreus** .1_4 r/c TSA: Calanus hyperboreus
TRINITY_DN6662_c0_g1_i1
MKCNICYFVCVIFNFNLVLGGLPLSSFNLTLEQSEQLRNGNDESINLAKDIMNDALKKYV
KEGNLNIVIENNDIIIDAAFEDRVLKDRCSLKLFALHPRAKGTIKRSSQLMADIYDGDFS
LGELSAAAKADLDVQLDLNFDFRAQIGAKIFGKCRKIGRDTLGIDLKTSGKAILAVALEG
TDLTIGPNLENIKFKLNLNILGRLENWNVDDLDVSKCQVKLFNRVEIGSYCSAARSLIRE
ALQGYINKWTKFEAPRLIEKLERKIQSRIGEEIVIPLNFNLENDLEDDDIFTL*

>**Rgigas_GIVD01061381 Rhincalanus gigas** _2 .1 TSA: Rhincalanus gigas contig104246.1,
transcribed RNA sequence
MRLTTTILLLCISRFSEAGFRLTSIELDEDTFQTCTYCDKRSDRAGGRASANALREFMTR
NHLDIVLNKRDVMLEKRLPNEGIPTGHSCKYRAEARNVIGRALMIPGTVNLGPEGVGYVD
EIKNSIAAADVAHAVEAEFDVRVRAGAKIFGSCIQIGRKTCRTNGSSEGINRVTAILAAS
NTLVECVNDQEHLSFNIDVTVDDITNLDTYKDIVVGKQNTCDLNVFGIKIGSINSKIRDY
ANKYLEGNEGIRSLRGPELVAELERVLGVELGSVVSIPITFRGSPRSCSSRRKRSTSRCT
ARKSCPDGYSRIGNTEKCQKYMGMKRPSCPITGSNIYSKKFGTMTLYWCQTPMV*

>**Temora_GJGX01080229** _2 TSA: **Temora stylifera** TRINITY_DN47023_c1_g3_i2, transcribed RNA
sequence; 3' partial
MLLTALFISGIYVNHSVGFILPSLELDEVSLNQTSKDPTEVS
KAVAAALRKFMTTNHVKIAVNGGEVRVSQSYPNEGIHTGHSCSKTAEAQDVRATAYMIPG
TVELDPAGVIDLGSNSIALAEVKHAVQVTLNVRVRFGTKIFGKCKNVGRKTCATDGYSEG
LNKISVNLVSSNVIVECIADFQHLTFNLDVAVVNERQDATYSGIEVGKKSGCNLGILGIN
IGSINTKVQQYASRYIASGIKFQNLRGPALVAELENKLGVQLGSTVSIPLNKEDGTPRYC
GPPTTTTTTTTKWLCASLDPKCFRLLLHPPPPLLPPVQKTQTSFPTCREFRGAGHPGLAS

>**Platy_GHXK01214142 Platychelipus littoralis** _3 TSA: Plit_DN14909_c0_g1_i1, transcribed RNA
sequence
MIVAQFDESGLSFPIQTLILGSDAFAAIRNNRNRQKNNYIVTQILNNQLREFIDRATTTI
SIADGEVVLDTHAPPMEIVNQCKATVRTGHIKTTVKVLKSSVLKSSTKISAEKDMWRASL
FLGGDLDVSLKAHAFVKARLGKRIFHRCLTKYRNYFPLSILGRGSIWIGAKIFATDVRVE
ERATPPNQAQYYEDPSQMPETIQYLVFKFQIQLNAKIRKWSVDHLGITGCDIKFLGVKII
SFCGTIKKRIYEYAHGFTNEFSEIHMPRLLDRIEQLLHQRVGDEIAIPLLLVDDRQEFVF
TLIEKASNVAKLKGDLIEDLGQLAGEVAKNFGLDDEEGKGFGNIAGTVAENLGFGQGGDN
SFKGLLALG*

>**Apocyc_GHAJ01003083 Apocyclops royi** TSA: TRINITY_DN44858_c0_g1_i1, transcribed RNA sequence
MIPLKELDRTPPLSWNGSGKSSPEKSSKTSEPEIFAGTLVIADDVLERADKENESLER
FKENDVKSTMSTSELKSTSDDVESTITPTFSTADEDFATESSSLLQDIVFFSLGVSSENQ
TQSLTEDSDSILENPNFEVDPTVEPLFFSTFQPEDFASTFANLPEMSLDEDDEPTTTISN
LGSKEECCRCPTGDIEQTRMKTFKEENSSRRGKQSFEDDKRNEIETEIQTEKETETQTET
ETEAQTETDTESGTEPTTSDGGSFDVFGLFDNPLVAYLVRLQQIYTLDLEVLDRLIALLK
GVNVEVIPAIMEALEPILPILLMDSIKTVDLKDLVSHLAHTVQKSSFQVSAITNFKNFDF
INVGPLNVRPTDFLQEDDFYRIIRTVIADYDHTGLQFPIQKIILGEHALKMFGSPENRNL
VSWILARKLYDYLESGNFVVEISDGEVMLDAHLPPAEVVDRCDTTLKLGAAHAKIRARST
STLHTTGIVPGTVGDPTALIVRGDVDVDVGVNAAVYARFGKNLFGHCFTKIRNNFPFSLL
SHGKAWVGARIVTSDVRIERRPPHTGQESEPLKRRILDLNKLLPEAAEGEKGASDGLKPF
LVFKINIRLEYQLVSFNVDHVGIDNCDLKFLGIKVFSACGLIQKTVKDNLKKYTRDLSEF
QAPKILHEIERLLRYKIGDEIAIPILLVDEKNELLTSLIKKADEVTKLKSDLLNDVSLLV

DVLSLLPKQ*

>Aplys_HBWR010030842.1_3 TSA: **Aplysina aerophoba**, TRINITY_DN15199_c0_g1_i1, transcribed RNA seq. 48/212 (23%) core homolog
EVQVSQSFPNEDINNSCSKRAKARNVVGKSQMIPNSVIMNKTVFAYNDLFKFAVAGADVA
AKVTFIADIRIRFGAKVLGKCIKVGRKTCGVKAVSNGINEILITLQGSNIQVLEFNGQEH
LVFKVGAKVFGKAKEGAFPPLSVSRTESCNIKILGIKVASINSYISKYSKRYFSNNSAYK
ELATPKLIKKLEQVIGAKLGDEVRIPIKIKGGNRRKRSVKISQCPKKCPSGWDRVGNEKQ
CVKHFGTNSVNCKSLNSEAQLRIIESIPGVKLHMCVIPF*

========== this Scylla sequence omitted because of mid-sequence stop codons === index ==
>Scylla_Hit4_GIXE01070519 consensus [red highlights filled from Nf seq]
IFFLFLQITLALKDVKVaISQKqFDEFKKPGNHDPLAQSLIQKPFqDFFDNPENQITISP
DDYSFSLKMADVQIDSSCGHRITCINNMVYGKINrGTKFTGKVEFDQNKLKMVAQANVNA
TIDIKTDVRLRVGFKLFGKCKRLLrKTVGINVISTGETVLGIEIqANNAKIEpIDGKYYL
VFNISYNVYGSVKSWNFDdIKANNCKIKIFGIRIASICGYVEKKVRQMGTKYINKATLLQ
IPKIIEKIQAKLEKKIGDTIKLPLKIIEKL

========================== **Fig 4C&5C data** ====== index ==========

Data for Figure 4C of main text [~~old 4E~~]. old 3H Protein **GHLB01028031 - Ankyrin**

"Ankyrins mediate the attachment of integral membrane proteins to the spectrin-actin based membrane cytoskeleton" (Wikipedia)

Bar	Species	Accession
Nf	*Neocalanus flemingeri*	GHLB01028031
Np	*Neocalanus plumchrus*	GJRU01068766
Nc	*Neocalanus cristatus*	GJRH01089088
Ca	*Calanus hyperboreus*	GJRE01151720
Eu	*Rhincalanus gigas*	GIVD01020829
Cn	*Eurytemora affinis*	GBGO01039159
Au	*Metridia pacifica*	GJAO01063710
Ha	*Tigriopus californicus*	JW521714
Cy	*Eucyclops serrulatus*	GARW01019055
Cr	*Eurypanopeus depressus*	GFJG01087429 (mud crab)
Ar	*Anoplogonius nigricollis*	GCYN01014357 (hemipteran)

`BLAST-scan` **Cala emergence, not Neo**

Taxonomic category Top hit 2022-05-14	Species	Bar	Cov.	E-value	% ID	Acc L	Accession	Notes
neofl_CV-2015-GAK1-S83R1	Nf DN15921		100%	1e-200	89.98%	1981	GHLB01028031.1	long seq
nplum-male-2015-R1	Np TR35457		100%	1e-200	80.83%	1985	GJRU01068766.1	
n-cris_CV2017_82-S6	Nc DN25221 i2		39%	2e-147	90.04%	783	GJRH01089088.1	3' end
			47%	5e-129	70.86%	1110	GJRH01089090.1	3' partial (+)
			52%	1e-122	71.83%	1002	GJRH01046914.1	5' partial (-)
			49%	3e-122	72.18%	1002	GJRH01046910.1	
TSA Megacalanoidea	*C. hyperb.*		100%	5e-91	36.42%	2069	GJRE01151720.1	
c-marsh_CV2018_n1-21-S1	Cm DN30439		55%	8e-57	41.85%	1279	GJRF01004686.1	
TSA Eucalanoidea	*R gigas*		16%	5e-14	39.58%	2145	GIVD01020829.1	
ebungii_CV2017	DN15092		16%	8e-11	38.54%	552	GJRG01030191.1	
TSA Centropagoidea	*E. affinis*		12%	5e-12	47.14%	614	GBGO01039159.1	M39L
	Pseudodiap		16%	1e-09	36.73%	1182	GHWV01116112.1	M116L
	Temora		12%	2e-10	42.86%	779	GJGX01169445.1	
TSA Augaptiloidea	*P. xiphias*		11%	5e-08	44.78%	356	GFCI01461428.1	
mpacifica_AF2017	Mp DN15316		12%	5e-09	45.07%	876	GJAO01063710.1	
TSA Harpacticoida	*Tisbe furcata*		16%	3e-09	37.89%	542	GCIT01014633.1	
	Tig calif.		16%	4e-10	36.84%	414	JW521714.1	
TSA Cyclopoida	*Eucyclops*		12%	1e-08	42.86%	1644	GARW01019055.1	
TSA Crustacea - not Cope.	*Eurypanopeus*		16%	4e-08	36.84%	832	GFJG01087429.1	
TSA Arthropoda not Crust.	*Anoplogonius**		12%	0.011	38.03%	999	GCYN01014357.1	
Top NCBI nr/nt	*E. affinis*		12%	3e-10	47.14%	386	XM_023467508.1	26S proteasaome

`* Hemipteran`

Primary protein sequences
NOTE: Initial aa well conserved, but lower taxa have M→I or L

>**Nf-ref**_GHLB01028031.1 DN15921_c0_g1_i1.p1 **Neocalanus flemingeri Len=579aa** [+**21aa of conserved 40aa N-term segment** missing but present in other isoforms]; NCBI nr/nt: *Eurytemora* 26S proteasome non-ATPase regulatory subunit 10-like 12% cov, 3e-10, 47%ID XM_023467508 [**region of max homology highlighted**] seems like a prime candidate for emergence ~*Neocalanus*; **Nice Calfi match first 95aa (underlined)**; 32% ID overall; 80% ID *N. plum*.; 90% in short (231aa) *N. cristatus* match)
MSRSLHDAIKSGNEEEVTVRLNMGEDVNQSFPPRHSTPLHTSIYCENEKMLQLLLVRGAA
VNQGDKEGITPLALAISKDRKSMAKILTAAGGIKTPSVLPILYENPDPLFSEPPPWLSEH
CIGVIEKDSGTKSNAKTSFSDILKRGPKGLETKPNKVGSDNKKQQFLKIFDKKEVKLETS
LNKPEEPKTSQNVSHEEIKQVPMQLYTEYETEHIEANDEVDTTNISVVENTDDQGLGTDN
VKTDTNSSAWNKTKLLSGMTNISKNVKTKLFSKASDKTSEIADKQATEENNETETKKDKL
RKRFSDIFSKNDKRFPVNITAEKEDKIKESSSQIAKLKSLFSTIKVKTNPKQQRFKKDDG
IEISGESNKEDKKELETKKEKLKKRFSDIFTKNGKGVPIKMTTENEDNTKESPAEIAKLK
TLFSTKVKTFSKKQTIKTNDGDEITGKKETFKQRLSDKTKAMANSDKISKLSVSFKGKIG
YGKGKGETGKETQKKEVSKSTNKIFEIKNKLSNIGKSNKCSEEETNETSKIGNLKNKFSN
LLKNREEKLENSGGLIMAENEIKRVSILKTRKEVQERESYMSKYQWAMVDGQWRKSQSVG
L*

>**Np**_GJRU01068766 _6 TSA: **Neocalanus plumchrus Len=580 aa** N-terminal missing
MSRSLHDAIKSGNEEEVTVRLN
MGEDVNQSFPPRHSTPLHTSIYCENEKMVQLLLVRGAAVNQGDKEGITPLALAISKDRKS
MAKILTAAGGIKTPSVLPILDENPDPLFTEPPPWLAEHCIGVIDKDSVTKSHAKTSFSDI
LKRGPKDLEIKPNKLGSDNKKQQFLKIFDKKEVKLETSSNKPESPNISQNLSHEEIKQVP
MQLYTEYETEYIEDNDEVDTTNIDVVESTDDQGLETEIVKTETISSAWNKTKLLSGMTNI
SKAFKTKLVSKTSDTTSEIADKQATEENNETETKKDKLKKRFSDIFSKNV**KGYP**VNIATD
NEDKIKESSSQIAKLKSLFSKIKVKTYPNQQPMKKDDGIEISGESNKEDNKESETKKEEF
KKRFSNIFTKNGKGVPINMTTENEDNTKESPAQIAKLKTLFSTKVKTFSKKQTIKTNDED
EITGKKETFKQRLSDKTKAMTNSDKIKKLSVSFKGKIGDGKEKVETGKEIQKKEVSKSTN
KIFDIKHKLSNIGKSNKCPEEETKETSNIGNLKNRFSNLLKNREEKLENSGGLIMAENEI
KRVSILKTRKEVQERESYMSKYQWAMVDGQWRKSQSVGL*

>**Nc2017_GJRH01089090.**1_3 TSA: **Neocalanus cristatus** TRINITY_DN25221_c0_**g2_i4, len=362** aa in ORF
NNQVIIF**MSRSLHEAIKSGNEEEVTVRLNMGEDVNQSFPPRHSTPLH**TSIYCENEKMMQL
LLVRGATVNQGDKEGITPLALAISKDRRSMVTILTAAGGIKTPSVLPILDENPDPLFSEP
PPWLSENCIGVIKKDSVTKSHVKTSFSEILKRGTKGLENKPDRLGSDNKKQQFLKMFDKK
EVKLATSSDKPEALIISQNFSDEGLKQVPMQLYTEYETKHIEDNEVDTTNIAVKESNINP
DLETKNEKDETKYLTGERILSLAGMKKISTNIKANILSKTSYKTSEITNDQATDYETNHE
EAKDEFSTTNISVLESTDTQDLENKNEKTETKLSSWNKTKILSTMKNISNNIKANFLSKT
YNKTSQNSDX

>Nc2017_GJRH01046914_DN22182_c0_**g1_i7** TSA ***Neocalanus cristatus* len=264**aa
MTTENEDKIKESSSQIAKLKSLFSTIKVKTNPKQQPMKKDDGIETSGESNKEDNKESGTK
KEKLKKRFSNIFTNNGKGVPIKMTTANEDKIKESPAQISKLKTLFSSKVKTFSKKQTIKT
NDGDEITGKKETFKQRLSDKTKARKNRQGKTGKETQTKEVSKSTNKIFEIKHKLSNIRKS
NKCSEEKTNETSNIGNLKNRFSNLLKNREEKLENSGGLIMAENEIKRVSILKTRKEVQER
ESYMSKYQWAMVDGQWRKSQSVGL*

>**Nc2017**_GJRH01046910.1 DN22182_c0_**g1_i3** Neocalanus cristatus [alignment includes whole transcript] [ORF seems identical to 914]
MTTENEDKIKESSSQIAKLKSLFSTIKVKTNPKQQPMKKDDGIETSGESNQEDKKESETK
KEKLKKRFSNIFTKNGKVVPIKMTTENEDKIKESPAQISKLKTLFSSKVKTFSKKQTIKT

NDGDEITGKKETFKQRLSDKTKARKNRQGKTGKETQTKEVSKSTNKIFEIKHKLSNIRKS
NKCSEEKTNETSNIGNLKNRFSNLLKNREEKLENSGGLIMAENEIKRVSILKTRKEVQER
ESYMSKYQWAMVDGQWRKSQSVGL*

>**Nc2017amlg_GJRH01089090+46914 Neocalanus cristatus Len=582aa** DN25221_c0_**g2_i4**+g1_i7; **APPROXIMATE amalgmation of disjunct pieces, maybe 2 genes??** 5'UTR: NNQVIIF
MSRSLHEAIKSGNEEEVTVRLNMGEDVNQSFPPRHSTPLHTSIYCENEKMMQLLLVRGAT
VNQGDKEGITPLALAISKDRRSMVTILTAAGGIKTPSVLPILDENPDPLFSEPPPWLSEN
CIGVIKKDSVTKSHVKTSFSEILKRGTKGLENKPDRLGSDNKKQQFLKMFDKKEVKLATS
SDKPEALIISQNFSDEGLKQVPMQLYTEYETKHIEDNEVDTTNIAVKESNINPDLETKNE
KDETKYLTGERILSLAGMKKISTNIKANILSKTSYKTSEITNDQATDYETNHEEAKDEFS
TTNISVLESTDTQDLENKMTTENEDKIKESSSQIAKLKSLFSTIKVKTNPKQQPMKKDDG
IETSGESNKEDNKESGTKKEKLKKRFSNIFTNNGKGVPIKMTTANEDKIKESPAQISKLK
TLFSSKVKTFSKKQTIKTNDGDEITGKKETFKQRLSDKTKARKNRQGKTGKETQTKEVSK
STNKIFEIKHKLSNIRKSNKCSEEKTNETSNIGNLKNRFSNLLKNREEKLENSGGLIMAE
NEIKRVSILKTRKEVQERESYMSKYQWAMVDGQWRKSQSVGL*

>**Chyp_GJRE01151720 _4** TSA: **Calanus hyperboreus** TRINITY_DN4891_c0_g1_i2_Chype_021, **Len=622aa**
MSRSLHDAIKSGNEEEVIARLNMGEDVNQSFPPRHSTPLHTSIYCENEKMMQLLLGRGAA
VNQADREGVTPLATAISQDRKMMVKILTAAGGTKTPSSLPNLYENLDPLFSHPPPWSSDS
CNGIIEQKVVNNPQPKESLYGFLKKGSKVLVNKPEKIVSDKNKQSVLKIFNKKEVKNEIS
SDKPLEQNLLTTLTDDAQKEHCSQEKKEETNVKAIDESSATNIAVLESNGDKIFEKENKK
MSDNQVNKDETKTESKTNKLMKTFSNIFEKDNTEEQMKLKEEKEGGNVKGIPGQIAKLKS
FLPLNGNKQTTKKEYEDEKIEKSVTVKQRLFAALKMKKTERKEETIQKEDDIQIKNIEKT
EEPTQNEDNENEKSTKIETFKQRLFDNLKMKKKEKTIESTPNEDTEHGIKNINKCETMKQ
RLLTKIKKTERTEETAQNEGTIGSKINNGFEIFCSKLTKQKKDEEIEQNKDQTKDIKVSG
KIENFKSKLSISFKCEGGDNDKEERKKEKKSIIKSNNVSDFKHRLSSIGKLKKCPDKDGN
KITENGTTDNSNKMDKLKWRFSNVLRSKEESLENDVTKTENHVQHERSIKLGKEVHKNEC
AMSKFQWIMVDGQWMKSQSVGL*

>**Rgigas_GIVD01020829.**1_2 TSA: **Rhincalanus gigas** contig34794.1, **Len=575**
MSRNIHDAIRSGNMSEVEIRLNMGEDINQSFPPKYQTPLHAAVEMDDSTVVEVLLDRGAQ
HSVGDRSDVTSLSLAIRLERTEIVKLLERKGATTKSTSSQVPEERLLGRPPPWAVDGLET
MKDNIEVSTDDDNSAFAVGENEETVKSVQFVPRKIQLSGWLTPSWTVKKEEDKTANDVND
IEEDKTTYNEKDNESGKISKKKNLERVNVIKDKLTVIKNKCTEKVSKKQDLDLDNTAMDG
KSNEGKTASKMSSNKDKSTSEARENKPVKSVNLSSWVTLPWTIEKVEDTVSKDVQEGEEF
DNQSQKIRKKTNIERVNTVKDKFAVIKNRCTGIIVSIGSQKNDTSSGEGGTSNNEVSEVI
TTSTPEQDEEAVKMIPRKVQLPDWLISHEGTKEGVKEQKLEKTEEGADITENEEVAIKSK
GQTVKDIKDKIAVFRNRCVGIVNTAESSDIEETNKLTMSGRIHNKLLLLTHKKNTVAIDE
DPDKGERYNLTKQNIIQTNEVTASNSEEITNNNMHKIHTKKRNVLRINKNDDETKDKVEK
EVDVKENDKPDNFVIAWLKSRQGIDLKFYTFRKKQ*

>**Eury_GBGO01039159.**1_2 TSA: **Eurytemora affinis** comp46836_c0_seq1 yellow is BLAST alignment segment;
MSLNLNDAAMAGNLEEVLIRLNLGEDVNQKYYPRYSTPLHDAVCCGREEVCKLLIERGAN
VNELDYKNMTPLKLAKRYGQDAIEGMLTAAGAKENVEPPKKTSITEDVHPPWVRKASRRE
LATA*

>**Mp2017_GJAO01063710.**1 _1 TSA: M**etridia pacifica** isolate Monoisolate TRINITY_DN15316_c0_g1_i2, yellow is BLAST alignment segment; 5'UTR: HR*QWTQRLQHTNSTAIDSELI*KSGSNN
MSANLNDAAMAGNMEEVLMRLNMGEDVNQKCYPRYSTPLHDATCCGRTEVVRVLLGRGAN
VNELDYKNSTPLKLATRYGQDDIVALLEAAGALSNVEPPKKTSVIDVDPPWVRKRSRVSR
QEEIAG*

>**Tigca_JW521714.**1_5 TSA: **Tigriopus californicus** SCN_Contig55568.Tcal3 mRNA sequence
SLNEAAMAGNLEEVTMRLNLGEDVNQKLYPRFSTPLHDATICGRIDVVKLLIERGADVNI

PDYKGMTPLKASRRYGQEEIEEMLVAQGAKLEVEVQKPKPCSDGVAPPWKRQISRRDEIS
SGQ*

>**Eucyc_GARW01019055** _5 TSA: **Eucyclops serrulatus** Eucyclops_serrulatus_contig_16507_1 transcribed RNA sequence; 5' UTR [note stoppered] VKVTTITKDKERDNK*KTILKF*ESLSFF*KKI*DK
MSASLNEACIAGNLEEVILRLNLGEDVNQKYFPRYTTPLHDATTCGRVQIARVLIERGAD
VNAKDYKGMTPLRLARRYGQDDIEEMLVAKGGRDEVDPPTTKKNSTGVDPPWVVKKVSRR
EGLITCGQH*

>**Cr_Eurypan_GFJG01087429**.1_1 TSA: **Eurypanopeus depressus** TR313277-c0_g1_i2 transcribed RNA sequence
NTSNERALTIMSASLNEAAAAGNMDEVLMRLNLGEDVNQKLFPRFSAPLHDAIICGRVEV
AKLLIKRGADINLPDYRGLTPLRLSQRYGQDEIEAVLAEAGALKEIEAKPKEKTVIGVQP
PWLVSSRKKRDEIAYGN*

>**Ar_Ano_GCYN01014357**.1_6 TSA: **Anoplogonius nigricollis** breed wildtype; len=222; 12K, 13R,
VVAARGGRFETVKLLLQSGANFNYKGRDGFTSLCEAAKAGHAEVVRLLLAEGAKVNEGNA
DNWTPLYKAAKAGKTDIVRLLLQKGAQANVANGNKWTPLHAAVEEGHEEIVRLLLAAGAE
VNVATTCHNWTPLHEAAAEEGRTDIAHLLLQKGAEPNVATHDTHWTPLHGAVFYRHEEIVR
LLLAAGAEVNARTTDGYTPLHWATARNHRSIADLLRAKGGVK*

========================== SF2A data ======== index ========

Data for supplementary figure SF2

Supplementary figure SF2 contains three taxonomic-similarity profiles and corresponding cladograms as additional examples of the approach to assessing novelty of non-annotated environmentally sensitive proteins laid out in Figure 4 of the main text. As in the main text, the bar graphs plot similarity (defined as $-\mathrm{Log}_{10}$(E-value)) of the *N. flemingeri* index sequence to top-hit transcripts in available TSA transcriptomes at different taxonomic distances. An increase in similarity of the BLAST E-value with increasing proximity to that of the taxonomic coverages indicated at the top is hypothesized to be the taxon of evolutionary emergence of each non-annotated cluster (red arrow and broken line) for 2 taxonomic coverages: *Neocalanus* genera (panels A & B), and "suborder" Myelinata (panel C). For the superfamily Augaptiloidea, similarities for the two species receiving hits, *Metridia pacifica* and *Pleuromamma xiphias* did not agree, so both values are shown on the bar plots with labeled arrows. The corresponding cladograms were based on only the *Metridia* sequence, so the branch in Panel A, nested within the Calanidae, is misplaced phylogenetically. Numbers at nodes in cladograms are MrBayes support levels (not RAxML). The tBLASTn results tables for the three panels follow below:

Data for supplementary figure SF2A (Protein GHLB01008164)

Neocalanus_GHLB01008164 (DN18692)

tBLASTn-scan hits - SF2A

Taxonomic category	Species	Co	Max score	Total score	Cov.	E-value	% ID	Acc L	Accession
neofl_CV-2015-GAK1-S83R1	Nf DN18692	Nf	475	475	100%	1e-169	94.17%	839	GHLB01008164.1
nplum-male-2015-R1	TR23091	Np	304	304	80%	9e-100	71.28%	1400	GJRU01045693.1
n-cris_CV2017_82-S6	DN21932	Nc	394	394	100%	1e-137	75.97%	906	GJRH01049230.1
TSA Megacalanoidea	*C. glacialis*	Ca	134	134	63%	5e-34	45.62%	1464	GJQS01003145.1

c-marsh_CV2017	Cm DN27097		125	125	60%	3e-30	45.39%	2088	GJRF01030004.1
TSA Eucalanoidea	*R. gigas*	Eu	78.6	78.6	62%	3e-14	32.90%	1134	GIVD01061107.1
ebungii_CV2017	no signif								
TSA Centropagoidea	Labma TR54691	Cn	52.0	52.0	59%	6e-05	30.67%	2105	GFWO01130581.1
TSA Augaptiloidea	*P. xiphias*		68.6	68.6	60%	9e-12†	32.67%	498	GFCI01509983.1
mpacifica_AF2017	DN24091_c0_g1_i2	Au	112	112	61%	1e-25†	42.68%	1896	GJAO01099549.1
	DN17728_c0_g1_i2		61.2	61.2	60%	5e-08‡	26.21%	2909	GJAO01040690.1
TSA Harpacticoida	*Tisbe*	Ha	57.0	57.0	53%	4e-07	31.06%	660	GCIT01015978.1
TSA Cyclopoida	*Apocyclops*	Cy	55.1	55.1	72%	3e-06	30.69%	950	GHAJ01024858.1
TSA Crustacea - not Cope.	*Eurypanopeus*	Cr	47.0	47.0	35%	5e-04	34.83%	307	GFJG01055337.1
TSA Arthropoda not Crust.	No signif								
nr/nt Arthropoda not Crust	*Limulus*	Ar*	52.0	52.0	45%	9e-04	29.31%	5757	XM_022394239.1
TSA Protostomia not Arth									
Top NCBI nr/nt	*Branchiostoma*				57%	2e-08	34.06%	2159	XM_035833808.

† Au - Note discrepancy between the two augaptiloideans (*P. xiphias* and *M. pacifica*) show with arrows on SF2-A1
‡ This *Metridia* sequence seemed to fit the expected pattern better than GJAO01099549.
* Ar category: no hits in BLAST-scan of TSA for Fig. SF2A; *Limulus* hit in nr/nt used to root cladogram

PROTEIN SEQUENCES:

>**Nf-ref_GHLB01008164** TSA: **Neocalanus flemingeri** TRINITY_DN18692_c0_g1_i1.p1 Neo Spatial&3year DEG 240aa; underlined is *Neocalanus*/calanid conserved motif

```
MKVAIICIVVMAYLLADTSGQETFEIKCGWTRMDNVEEKLDELKEEVITRMDIVEQKMDD
LKQLLEEVIANNKVNPAPMVDAGNIIPIPAHPQCKMDYTTWTEDWRMVRYSTQPHDPKHC
DQNPASSTNTGLTSGWHRFVFNGVHARIPTQAPAYQYQGFAQTCGTNRASWMDGSLPSLG
EPPNNVTMKFSGGDKAEYNTAGTKPAKVVACLDGNLHTFYLYHLVPVRDCYGGYCATTVF*
```

>**Np2015_GJRU01045693** TSA: **Neocalanus plumchrus** TR23091_c0_g3_i4

```
MVNLPFLLLFCALPISSSSPDTNTPTLSEAVKIIQVQAIQALNAKIAEQEILIKKKEDET
RGLKNVIAKQKTEKETSIKENEEKISGLEKQVEDSGVLVKFMMTVLKHSEELMIERQNMI
TAQGIKLKEHQNQNQVLLRDQEIQTEIITNQNNVKANLKRLVGIDNQLISAYKEIESSKP
SENCEVLPYVAGLAQTVGTQKKEISNLKTVMKTEAKIAEQLEKMKEQMAKLSNEESDWDA
AIHYQNIIKKQSNGIRQLLTLASYINIQSVEHFSHDDAGNIIPVPGHPQCKMDYTTWTEE
WRMVRYSTQAHDDMHCDKNPASSTNSGLTSGWHRFMFNGVHARIPTQAPAKQYQGYAQTC
GTNGASWMDGSLPYLGEPPNNVIMKFSGNGNPEYATSGRKPGKVVACLDGDLNTFYLYHL
VPLQDCYIGYCATNVF*
```

>**Nc2017_GJRH01049230** TSA: **Neocalanus cristatus** DN21932_c2_g2_i2; YSCG

```
MKVAIICVVVMAYLLVDTTGQETFEFKCGVEELQENVMTRMDTLDGKLTRMDNVEEKLDE
LKEEMITRMDIVEEKMDDLKQLLEEVIANNKVNPAPMVDAGNIIPIPAHPQCKMDYTTWT
EDWRLVRYSTQAHDPKHCDNPASSTSTGLTSGWHRFVFNGVHARIPTQAPASQYLGYAQT
CGTNRASWMDGSLPSLGEPPNDVTMIFSGNGKAEANTTYRKPSKVVACLDNNDQTFYLYH
LVSVYSCYIAYCATTVF*
```

>**Ca_Cglac_GJQS01003145** TSA: **Calanus glacialis**[Lizano] 282aa

```
MTTTIYTPTTTTTSTTTTTTSLTSTITSTKTTTTSTTFMTYTTTSTTTTTTSTTTTTTST
TTTTTSITSTTTTTTSTTTTTTSTTTTTTSMTKTIKSTTTTAKSSSEDLIRALKRVVVKI
EYVVKIDYVINMAGRKARAGNQDCSWLDTRLDELIFLLSGIDPDDITEQQKEDALEIGVD
IFGTVIGVCSTSEKAALQEDKSKLEIIICKLGKKSDCTTTSTITTLTTTTSTSTIDIMAT
TGDPQCDRSYITLTDDWRRIEYGVKHPDYKCDQAGSWFSHTGLTAGWYRFYFPSAPYAKI
```

PTTAPLKQYVGFDHQSCGTNAVSWMDDSLPAIGQPPKDVTINFAWHANYRKGFRDYSKGY
PTPAKIVACPAGSETMYLYFLSPTPDCYLAYCAL*

Cm2017_GJRF01030004 no annotation

>**Eu_Rgigas_GIVD01061107** [Eu] TSA: **Rhincalanus gigas** contig103802.1, transcribed RNA sequence
NLQTCSVRTCTDGRPSVWVDKPVYPGQCNCCKFEDNMVEPGYTKAVNKHFLAVCREGEIL
YRKRGTEEEGGEGGGGGGEGVGVGGGEDGGGGPDTTTEKTKKNVTTSTTTTSTTTTSTTT
STTMTTTSTATITSTTTSTTTSTAITTTSTTTTTTSATTSTTTTTTSTTTTTTSTTTTTP
NKSAGQCSEPYTELKEVWRKVNYTPGGATPCDFLHCDRRMEGWYESEFTAGWYRFVEPAG
VSLPMAPPVMSGKGDPCEVCQTKGSAWISERRHPTVGEDVMDVTFYFAWAGKENLSDGLS
TKGQVVACQDQDNQVFYLYKLAPVTDCELAYCAIGA*

>**Cn_Labma_GFWO01130581** [Cn] TSA: **Labidocera madurae** TR54691_c1_g2_i2 TSA: TR54691:c1_g2_i2 transcribed RNA sequence *QGNEK
MAKNLKTPETLPWIPPGQTKEETFNKFAHKKGSIASRINQEVAAKKSSISNLSKKSVSLN
RSGSSSNILSLSDSGRGSQLTTSDRNELKDKIVEIFEEDENEYDSNGENRFNDSGRSSSG
SDSSPRLRKRDQYKTSFHSDSDNDSLKENKSQPKNHLRNKVADFITNDGKFRWMKDVRTP
LDVRALTPNTAEMRGYQAMRRELDQEERRPGSLESRKSIRYHDTEDPRHRSYPTDLNQSF
TTYSVSPRYKLSDPNQRPGDVTEAWLVFPEGRVPQNGSHVGDYPSPRSKLSIGSQPLPVN
GRLSVRDYPSPRRIPGEYPRIGEKGVGRAIPVNLASSVGSMLKNSFANINSVRETPVETF
MTEEDTDRILRQEKSKSRCPTWVWGFLICLAVFLLIAIIGTVTGVMTAAEANSANLMLS
NQPGPGDNSPCKDYVALTEKWRRIIGKGPSQTGQPYSCDNPFKEGWYRFDGEAGSRLADN
TNPPSWEDCGTSRVGWLSGSHPEISDGTVNRTLCVTAFNGNCEVKMPNGQVRGCPDPRGG
VFFVYYLVQPRISCNWGYCAVGEPPS*

>**Mp2017_GJAO01099549** [Au] TSA: **Metridia_pacifica top hit** DN24091_c0_g1_i2 **Len=616aa; grey highlights: 3 FN3 - Fibronectin type III domains (via Prosite)**; annotates as Down syndrome cell adhesion molecule-like protein 1 homolog
MLDMGIQLLCLLVLGLPNQVSLQDRFDLSANHGIANADVPDPPYGLKVLEKSAMTVKFSW
QSPYNGNFDITRYLIEYKVSTGTWKNDIDRVLVMGDETIARVFSLHPATSYQFRIVAQNL
IGYSGPSEIITIETSETKPTGPPVDVRLTALDQHTLQVNWKPPLKEHWNGAILGYYVGYK
KTSKGDDKPFVFETVEYRKLKQGTEHTLEISNLDASTKYAIIVQAFNKIGQGPISEQAIM
FTTEGVLSVDGPPSPQARIKGGKPTIPDALSFLEVSAGSVTLHLDAWSDGGSALIYFEVE
YKPKEQRGWTLVSNNVQPRGNFVVLDLIPATWYNLKVTAHNNAGRSIAEYEFATLTTEGE
MITSEAPPTTESTPEAIQLLKTNIELMETMHNEMKIMHNDLGRMTEINNILLNARIDTLE
ALILGEWSSWEGCRADQHQSRERVQVPVLRNCSTTNHPQCQRHFSTLSDEWRRVEYTSNP
TRYSNQRNDNTITNSGSEQTMRWFRFSFPNSPNGTYAWIPTSPPAQKYLTGSPGKTCGIL
LSSWMDGSLPSQGEPPKSVQIKFAHRSVNSKSYRKSAKVVACYDDDMNIFYLYGLHNTLY
ADAGYCATTGDDKEIV*

>**Pxiph_GFCI01509983** _2 TSA: **Pleuromamma xiphias** TRINITY_DN273476_c0_g1_i1 transcribed RNA sequence - NCBI BLAST alignment with Nf_ref in green/yellow
ENGPECYSYTALTDSWRKIDYNNGGPLGPYKCDTGLVKKWYRFEGEAGSKLPTERDPTGK
ITSGKNICGTAATSWMTSAHPQPKDQIVTRNVCFEYGDKGCYFSDDFTIRVRACIDYDLS
VYYIYELLPPPDCYYAYCALP*

>**Mp2017_GJAO01040690** _1 TSA: Metridia pacifica isolate Monoisolate TRINITY_DN17728_c0_g1_i2, transcribed RNA sequence [grey highlight: ADAM_metalloprotein binding site no bHLH motif found by Prosite]
AHHDCRLNYLLQNSNMEMIYLLLFFMVFHLVIEANPSDSLKEDSEILKASLPLFTWIDDV
KIKVEFPDGDLDVIVLWKAEKIAGIEVPCLWDGTMEEEPASDVVVSGCHNDPLVSMTIHS
NRLIGRTMFNIRNGATEEIQNPYANNHAINELDDYGDEKIQRIKRSLFEDYVEEDYADYA
ADGHIDPPPRLNLDMKLGFEESLKKHLGGETAARNYVIEVFTHAKSYMKHQSLGTQLNLN
LVEEPTFVPNKNWKARRQYMVEAKTVKTNESVPIIFFCYDVEDDTFMGISKIRGACDPTG
GVGIVKHRENVQLAANTLNH**E**HG**H**LVGLQHDQAEDNKNKEEAINNPCHGNGLMSEISPSE

RPSQFSYCSARRFKLWWNNYGTDCLTEAKIPLNLKTCPYPEPSTIAPCECKVDTNTYQVY
LFCNTMNQNLATTLATSFGCMNKIHRLEINMMGSQLDCTFGRENIGVLELAYFQLYNISK
INSDIDANAFAGSSNSLKEIKIQRLPEMEAGGRTIKVEALSNLLALENIQLGEDFKTLQT
KSFSNLPSILKLSIEGVKIVQSKAFHQMPALKKLDFTKCLVEIISTEAFFNLPSLTTIIF
NTGTITTLQTKSFSGLPSLLKIDMSESKIHTLPTNSFHNLNKLSIIDISNNLISTIPTNA
FSNLPALTKIDLSNNKIATIDNNAMTSLPKLVLLDLSKNEFKEMGKSLENIKNSDLVVNL
TVTKIKFLTEESFGPFINNIVNLGGKGFIDVSTLEIPCRCDINWLFSQQNMLGPSKFHNA
VCEEGHTINEVDSNLLKRFCTDSCKLQQKKLQALSENGPECNSYKSLTESWRRIDYQNRG
RKTTHCDDKANKFVKAWYRFEGDAGSKLPTTSAPTGIPTINSMICGTHRTSYMTTEHPEP
SDGIVQRKICFEWSTNKCDQHSTIRVKACHSNDLSIYYVYELIPQTGCHNAYCALP*

>**Ha_Tisbe GCIT01015978** [Ha] TSA: **Tisbe furcata** Harpacticoida strain wildtype C181110_a_5_0_l_660, transcribed RNA sequence
EKEDDVSWEEMRSRKRKVAVMAAVATAVGLIILGAVIVGGAVAMTKGGTSTPSSTINSAS
SSAATTDSGGTKTGGGTGGTADSLSDNTPCLEYTKLTDDWRNIEGQNIRTVIDKGYHCDM
YNKATGRGLQDGWYRFDGDAGTYMPTVPPGSETCGTSKVGWMDGNHPLISEGTLERKFCF
HWQSECEMSFQGKVRTCEDQDNNIFYVYYLKTSNAPHCQX

>**Cy_Apocyc GHAJ01024857** [Cy] TSA: **Apocyclops royi** TRINITY_DN40583_c0_g1_i1, transcribed RNA sequence; underlined portion corresponds part of the sequence included in the BLAST alignment GNFRGNQNCPGCPIFQFPNFHNQGFQGFPGNQGFPGNQGIPGNQGFPGNQGFPGNQGFPG
NQGFPGSHGYLEQAYQGLNYVNQGHQGYPQGLNNSFDSRAKISHTFHGKQSQTDGQFSEF
GDDMGFSPSSMGYNSVHFERSRQSRMSQNGPSRNEAWVIFPKNSESNMSEQVTAYQAKPL
NLASSISSRLRESFAQINRSSTPVEPTVRSQSADPLVDDLSDEERTRKKRRNALIAGGAA
CCGTILLAIILMLALILGTMSGSSSSGNIQSPDYYDGSGANNDTKPCKTYKSLTESWRRI
VGYGPLKTGAPYHCDMPRFGSSQTGIQEGWYRFMGEAGTHLATSSPGAFSPESGTLKEVC
GTSLVGWMNGQHPKIYEGIVERNFCFEWESGTCQYGYKSMVRTCPDPTGGPSDTFYVYYL
KIPRDIQCNFGYCAEP*

>**Ar_Limulus XM_022394239** _3 PREDICTED: Limulus polyphemus von Willebrand factor D and EGF domain-containing protein-like (LOC106466266), mRNA; 1,531aa; underlined portion corresponds part of the sequence included in the BLAST alignment (the rest was in the 5'UTR
MARIPTSCPEVNHCGTQAPIWLPMNISLQAGQVHQVNYCTTWVIPGSQSSGCCTFRDFLK
VKNCGSFLVYHLRPTRGCHMAYCAEIVELSETVIGLSRPPRIRPRVIQQEIVLICEFDIH
VNGVSVGRTSELDKFVSISWFKLDEENKRRIHLQTFQATSKQDELLVVGKHAHLGEMVLC
MVKLQHKNTKSVRNSSLRSEPFYLGIRAEPQNLVISENGSFHPITLTSSLPYRCTKHDYK
GCKLLIQLRTRAMSSSIQSSDVALSSCIVHLPVREQCQKNCGTGRFFVTAIQDLIEESST
KNIIEAKLLGVEDSLWTEYKIPDVEVTIQDIPFGWCYSFTEPHYITFDSRRFDYYKTGTF
VLYRNTLRNFEVHARTWLCDSSHKSVSCNCGFAALENNDLVRVDVCGPKLLNTTPSIFMT
SLGNTEPEAKIIKSKQGKILTVLFQSGSFVRAIFEKWGISIVLRVPGLDFGHTRGLCGIF
DKDPTNDFHDTDGNILDSDVNKFFSIWSLDSGESLFDEIPYETFTSSNHHSCMCSSPVFK
PTSHSNRNLGVSKYSVFSQNGYVFHNHHECNSSNNLNCVKSCEVPKILDFHHLLYHIDIT
KTVVLEKGYKIVEENGEVRDININKRYSGISNEFYWLNNKSSGYNETDIKPDVKKSSSYF
LEIPLQRDRPFSTENSKSQIVKPFQFKNVTKRKFQHTREKWDIKSRKERAPRGDIPVHMV
LPFTSKSDLALFPSSESVTRWPTPSGRTKEKIVNICTDVLTNSTIAQACSQYLEKHMLEA
IGICVLDVTLTDEPDWAKYSIPLLESRCEEEVLRQHVDLTKDEKGLYLQDIIRALSCPEG
CNDHGQCVEENCLCDKDYYGIDCSLYKGSEEIFTLQEAGIEGLCDDLTSSCSQVRVTGSN
FKASLTFSCKVTLVKFDNNTGDFHIAGTISILSASYLDNQTIQCPLPDTAERRITAGSET
TWEIKISNDHVEFSTPVYVTVYNSSCQSCVPGHPPLCKVHDLLGHDAVLAWTHLQAPYLK
DLCMAPILVHQHSTLTNLVTRLSQSGLSMIEVLPCKCENKGKCVKSSYYKAGNGNYTCSC
NPGFTGEFCEFEVDPCAPNPCSHGMCIISSKGFQCVCYVGFSGEFCEIQSDPCVHNPCDH
GLCIIDGKGFQCICDIGFSGEFCEVGIDPCLSNPCYHGVCVGRGSGFQCICDVGFSGLFC
EEQQDKCTSKPCYPGVTCSSVDGHFICGTCPAGMTGNGVSCFVALCHLTCLNGGLCFNQD
SCWCPRGFTGNRCETAVCDPPCINGGRCLGGNKCACQYGYVGRICETAVCSRSCQNGGLC
KTPNKCSCLSGWRGPTCEEPVCDPVCQNGGTCSQPNLCTCPPGFFGVDCSSSICHPPCHN
GGHCMRHNICSCPKGWIGARCWIPVCEPRCLNGGRCWKPNKCSCPSGWRGRRCHKATCLV
RCRNGGTCVKPNICVCPPGFKGLHCEEPTCSESCYHGGKCVSPGNCLCYPGYSGINCEIS

```
QKKNIHKRRYQIYRHRHGYRLQNLFTHVHDA*
```

NOTES:

1.This candidate novel, differentially-expressed protein is TransDecoder-complete, with the Met start being the first Met after a TAA stop in the same frame (position 4 in the nucleic acid sequence). This is important since the *N. flemingeri* sequence is substantially shorter than some of the homologs (perhaps due to missing an exons).Two calanoid homologs (*Calanus glacialis* and *Rhincalanus gigas*) were characterized at the protein level by long poly-threonine sequences, but this was not the case for the Neocalanus homologs, which makes cladogram formation a bit iffy.

2. **Augaptiloidea anomaly:** The main aberration, which shows up in both the BLAST-scan bar graph and the cladogram, is in the augaptiloidean category. Using the standard work-fpow described in the Methods, the top hit in the augaptiloidean taxonomic category was used for the bar graph and the cladogram (a *Metridia pacifica* sequence). This had a much higher similarity score (-log10(E-value) = 25) than expected for its phylogenetic distance from *N. flemingeri*, causing an incongruously prominent bar and the corresponding branch of the cladogram to be incorrectly nested within the Calanidae. There were in all 5 *Metridia* hits (all Trinity DN17728) before any other species (*Rhincalanus gigas*), 4 with E≤2e-24, 152aa alignment piece with N. flemingeri bHLH motif region. The one with a more appropriate similarity (E=6e-16) had almost the same match pattern as the others as far as it went, but its alignment piece was truncated at ~92 aa , giving it a higher E-value (lower similarity). However, the top hit for the augaptiloidean*, Pleuromamma xiphias,* had a more appropriate similarity score (11) and an alignment piece that covered the same motif-range as did that for *Metridia.* This had lower identity (33% vs 43%) as did the top hit for *Rhincalanus*. This sequence had a SwissProt BLASTx annotation as a Down Syndrome protein, annotation being lacking for the *Neocalanus* homologs. There is, however, a "hidden" *Metridia* protein with a more appropriate match: GJAO01040690. The match pattern seems to follow that of *P. xiphias*and *R. gigas*, albeit with only 26% identity. ReBLAST of the *Metridia* sequence brings up E0.0 hits with *P. xiphias and* a 4e-81 with *N. flemingeri* (GFUD01011651; 6e-75 in Nf2019 GJRT01033647 annotating as G-protein coupled receptor GRL101), so this is a respectable calanoid protein. There appeared to be no *P. xiphias* or *R. gigas* sequences that matched the high-similarity *Metridia* sequence.

3. **Outgroup:** The search for a suitable outgroup was difficult. The sole crustacean hit (*Eurypanopeus*) was a short partial protein sequence. No arthropod hits were found in TSA. The closest non-arthropod sequence in a BLAST was to a cephalochordate (Amphioxus: *Branchiostoma blecheri*) but it did not provide a good root for the protein cladogram.

4. The best outgroup found was in NCBI nr/nt from *Limulus polyphemus* (XM_022394239.1 E-value=8e-04).   Its cladogram was relatively well behaved in terms of matching the evolutionary relationships among the various taxonomic categories.

5. **Annotation:** Motif search did turn up (also via a SwissProt protein BLAST) a *Branchiostoma* hit (A0A6P4ZKW5) "Neurogenic locus notch homolog protein 1-like (mabe E-vale = 1.4e-10? not sure what that number applies to), also with mention of "Uromodulin-like" and "von Willebrand factor D and EGF domain-containing protein." The *Limulus* homolog also annotated as von Willebrand factor D / EGF domain protein.

6. **Motifs:** The C-terminal consensus motif (x-padded t ocorrespond to EMBOSS-Cons):

```
DAGNIIPiPaHPQCKMDYTTWTEdWRmVRxYSxxxTQaHDpkHCD-NPASSTntGxxLTS
GWHRFvFxxxxNGVHARIPTQAPA-QYqGyAxQTCGTNrASWMDGxxSLPsLGEPPNnVT
MKFSGngkaEynTagrKP-KVVACLDgnl-TFYLYHLVpvdCYGGYCATtVF*

or as generated by EMBOSS-Cons:
>EMBOSS0001
xxxNiiPxpxhPQCkKdYTTxTEDWRRVxxYSxxxTQxxDpxHCDQnxassTNTGxxLTS
GWYRFVFxxxxNGxHArIPTxaPAxQYxxGyxxQTCGTNxASWMDGxSLPSIGEPPxDVT
MxFSxxxxSexSxsxxKpxKVVACxDxDxnTFYLYHLvPvdCylGYCAtxxx*****
```

CLUSTAL/MAFFT

```
Nf          -DA---------GNIIPIPAHPQCKMDYTTWTEDWRMV-RYS---TQPHDPKHCDQNPAS
Np          -DA---------GNIIPVPGHPQCKMDYTTWTEEWRMV-RYS---TQAHDDMHCDKNPAS
Nc          VDA---------GNIIPIPAHPQCKMDYTTWTEDWRLV-RYS---TQAHDPKHCD-NPAS
Ca          ------------IDIMATTGDPQCDRSYITLTDDWRRI-EYG---VKHPDYK-CDQAGSW
Eu          -------------TTTPNKSAGQCSEPYTELKEVWRKV-NYTPGGATPCDFLHCDRRMEG
Cn          MTAAEANSANLMLSNQPGPGDNSPCKDYVALTEKWRRIIGKGP--SQTGQPYSCDNP---
Mp          --------------------HPQCQRHFSTLSDEWRRV-EYT---SNPTRYS-NQRNDNT
```

```
Cy        --------------GSGANNDTKPCKTYKSLTESWRRIVGYGP--LKTGAPYHCDMPRFG
                                .    :   .: ** :                :

Nf        STNTG--LTSGWHRFVF----NGVHARIPTQAPAYQY-QGF-AQTCGTNRASWMDG-SLP
Np        STNSG--LTSGWHRFMF----NGVHARIPTQAPAKQY-QGY-AQTCGTNGASWMDG-SLP
Nc        STSTG--LTSGWHRFVF----NGVHARIPTQAPASQY-LGY-AQTCGTNRASWMDG-SLP
Ca        FSHTG--LTAGWYRFYFP---SAPYAKIPTTAPLKQY-VGFDHQSCGTNAVSWMDD-SLP
Eu        WYESE--FTAGWYRFVEP---AG--VSLPMAPPVMSG-KGDPCEVCQTKGSAWISERRHP
Cn        -------FKEGWYRFDGE---AGSRLADNTNPPS--------WEDCGTSRVGWLSG-SHP
Mp        ITNSGSEQTMRWFRFSFPNSPNGTYAWIPTSPPAQKYLTGSPGKTCGILLSSWMDG-SLP
Cy        SSQTG--IQEGWYRFMGE---AGTHLATSSPGAFSPE-SGTLKEVCGTSLVGWMNG-QHP
                     *.**       .         .          : *     .*:.    *

Nf        SLGEPPNNVTMKFSGGDK-----AEYNTAGTKPAKVVACLDGNL---HTFYLYHLV-PV-
Np        YLGEPPNNVIMKFSGNGN-----PEYATSGRKPGKVVACLDGDL---NTFYLYHLV-PL-
Nc        SLGEPPNDVTMIFSGNGK-----AEANTTYRKPSKVVACLDNND---QTFYLYHLV-SV-
Ca        AIGQPPKDVTINFAWHANYRKGFRDYSKGYPTPAKIVACPAGS----ETMYLYFLS-PT-
Eu        TVGEDVMDVTFYFAWAGK-----ENLSDGLSTKGQVVACQDQDN---QVFYLYKLA-PV-
Cn        EISDGTVNRTLCVTAF-------NGNCEVKMPNGQVRGCPDPRG---GVFFVYYLVQPR-
Mp        SQGEPPKSVQIKFAHR-------SVNSKSYRKSAKVVACYDDDM---NIFYLYGLH-NT-
Cy        KIYEGIVERNFCFEWE-------SGTCQYGYKS-MVRTCPDPTGGPSDTFYVYYLKIPRD
             :   .  : .                      :  *          :::* *

Nf        RDCYGGYCATTVF*-----
Np        QDCYIGYCATNVF*-----
Nc        YSCYIAYCATTVF*-----
Ca        PDCYLAYCAL*--------
Eu        TDCELAYCAIGA*------
Cn        ISCNWGYCAVGEPPS*---
Mp        LYADAGYCATTGDDKEIV*
Cy        IQCNFGYCAEP*-------
                 .  .***
```

is present in the three *Neocalanus* species, but not in the non-*Neocalanus* homologs. Its evolution in the *Neocalanus* genus may be hypothesized to explain the abrupt change in E-value observed for this genus in the BLAST-scan. The sequence starting "PQC" (second green highlight) has good identity in all 4 calanid homologs (and also *Metridia*). The lower case letters in the 1st consensus indicate the consensus of 2 out of 3 among the *Neocalanus* sequences; hyphens indicate differences among all 3.

NOTE that conserved shared motifs like these are strong promotors of good BLAST-scan similarities. Another good example is the ankyrin motif in the main text Figure 4C and 5D.

8. **Interesting feature**: The portion of the *Limulus* molecule that gave rise to its identification as a homolog was based on nucleotide sequences (tBLASTn), and its BLAST alignment was restricted to the 5' end of the long 3K nucleotide sequence and included part of the 5' UTR of the translated sequence (i.e. the N-terminal of the protein). However, its use as an outgroup for the cladogram was based on the protein sequence (ORF), and thus excluded this UTR region. Its alignment with the other protein homologs (using MAFFT), was at the C-terminal end of the sequence, a peculiar switch

BLAST alignment:

```
Query  132  LTSGWHRFVFNGVHARIPTQAPAYQYQGFAQTCGTNRASWMDGSLPSLGEPPNNVTMKFS  191
            L  GW++F  NG  ARIPT  P   +      CGT    W+          P N++++
Sbjct  228  LEHGWYKFEINGTMARIPTSCPEVNH------CGTQAPIWL----------PMNISLQAG  359

Query  192  GGDKAEYNTA----GTKPAKVVACLD----GNLHTFYLYHLVPVRDCYGGYCATTV  239
               +  Y T     G++ +      D     N  +F +YHL P R C+  YCA  V
Sbjct  360  QVHQVNYCTTWVIPGSQSSGCCTFRDFLKVKNCGSFLVYHLRPTRGCHMAYCAEIV  527
```

MAFFT alignment (part) - portions aligned in all sequence in yellow); Blue highlights the unannotated motif found in this protein: the PQC motif characterizes the Calanidae sequences and the DAG all *Neocalanus* seuqences

```
Nf-ref_GHL -DAGNI--------------------IPIPA----------------------------------HPQCK------------------------------------------MDYTTWTE
Np2015_GJR -DAGNI--------------------IPVPG----------------------------------HPQCK------------------------------------------MDYTTWTE
Nc2017_GJR -DAGNI--------------------IPIPA----------------------------------HPQCK------------------------------------------MDYTTWTE
Ca_GJQS010 -STIDI--------------------MATTG----------------------------------DPQCD------------------------------------------RSYITLTD
Rgigas_GIV --------------------------TPNKS----------------------------------AGQCS------------------------------------------EPYTELKE
Labma_GFWO -NSANL----------------MLSNQPGPG----------------------------------DNSPC------------------------------------------KDYVALTE
Mp2017_GJA -SRERVQVP-------------VLRNCSTTN----------------------------------HPQCQ------------------------------------------RHFSTLSD
Tisbe_GCIT INSASS----------------SAATTDSGGTKTGGGTGGTAD-------------------SLSDNTPC------------------------------------------LEYTKLTD
Limulus_XM NNTGDFHIAGTISILSASYLDNQTIQCPLPDTAERRITAGSETTWEIKISNDHVEFSTPVYVTVYNSSCQSCVPGHPPLCKVHDLLGHDAVLAWTHLQAPYLKDLCMAPILVHQHSTLTN
        918

                                                              .    .:
Nf-ref_GHL DWRMVRYS---------TQPHDPKHCDQNPASSTNTG---------LTSGWHRFVF----------------------------NGVHARIP----------------------------
```

```
Np2015_GJR EWRMVRYS---------TQAHDDMHCDKNPASSTNSG---------LTSGWHRFMF----------------------------NGVHARIP----------------------------
Nc2017_GJR DWRLVRYS---------TQAHDPKHCD-NPASSTSTG---------LTSGWHRFVF----------------------------NGVHARIP----------------------------
Ca_GJQS010 DWRRIEYG---------VK-HPDYKCDQAGSWFSHTG---------LTAGWYRFYFP---------------------------SAPYAKIP----------------------------
Rgigas_GIV VWRKVNYTP------GGATPCDFLHCDRRMEGWYESE---------FTAGWYRFVEP---------------------------AG--VSLP----------------------------
Labma_GFWO KWRRIIGKG-------PSQTGQPYSCD--------NP---------FKEGWYRFDGE---------------------------AG--SRLAD---------------------------
Mp2017_GJA EWRRVEYTS------NPTR----YSNQRNDNTITNSGSE-------QTMRWFRFSFP---NSP---------------------NGTYAWIP----------------------------
Tisbe_GCIT DWRNIEGQN------IRTVIDKGYHCDMY-NKATGRG---------LQDGWYRFDGD---------------------------AG--TYMP----------------------------
Limulus_XM LVTRLSQSGLSMIEVLPCKCENKGKCVKSSYYKAGNGNYTCSCNPGFTGEFCEFEVDPCAPNPCSHGMCIISSKGFQCVCYVGFSGEFCEIQSDPCVHNPCDHGLCIIDGKGFQCICDIG
               :                                             : .*
                  .    :

Nf-ref_GHL --------------------------------------------------TQAPAY----------------------------------------------------------------
Np2015_GJR --------------------------------------------------TQAPAK----------------------------------------------------------------
Nc2017_GJR --------------------------------------------------TQAPAS----------------------------------------------------------------
Ca_GJQS010 --------------------------------------------------TTAPLK----------------------------------------------------------------
Rgigas_GIV --------------------------------------------------MAPPVM----------------------------------------------------------------
Labma_GFWO -------------------------------------------------NTNPPS-----------------------------------------------------------------
Mp2017_GJA --------------------------------------------------TSPPAQ----------------------------------------------------------------
Tisbe_GCIT --------------------------------------------------TVPPG-----------------------------------------------------------------
Limulus_XM FSGEFCEVGIDPCLSNPCYHGVCVGRGSGFQCICDVGFSGLFCEEQQDKCTSKPCYPGVTCSSVDGHFICGTCPAGMTGNGVSCFVALCHLTCLNGGLCFNQDSCWCPRGFTGNRCETAV
                                                                *

Nf-ref_GHL -------------------QYQ-GF-AQTCGTN--------------------RASWM-DGSLPSLGEPPNNVTMKF-------------------------------------------
Np2015_GJR -------------------QYQ-GY-AQTCGTN--------------------GASWM-DGSLPYLGEPPNNVIMKF-------------------------------------------
Nc2017_GJR -------------------QYL-GY-AQTCGTN--------------------RASWM-DGSLPSLGEPPNDVTMIF-------------------------------------------
Ca_GJQS010 -------------------QYV-GFDHQSCGTN--------------------AVSWM-DDSLPAIGQPPKDVTINF-------------------------------------------
Rgigas_GIV -------------------SGK-GDPCEVCQTK--------------------GSAWISERRHPTVGEDVMDVTFYF-------------------------------------------
Labma_GFWO --------------------------WEDCGTS--------------------RVGWL-SGSHPEISDGTVNRTLCV-------------------------------------------
Mp2017_GJA -------------------KYLTGSPGKTCGIL--------------------LSSWM-DGSLPSQGEPPKSVQIKF-------------------------------------------
Tisbe_GCIT --------------------------SETCGTS--------------------KVGWM-DGNHPLISEGTLERKFCF-------------------------------------------
Limulus_XM CDPPCINGGRCLGGNKCACQY--GYVGRICETAVCSRSCQNGGLCKTPNKCSCLSGWR----GPTCEEPVCDPVCQNGGTCSQPNLCTCPPGFFGVDCSSSICHPPCHNGGHCMRHNICS
                                      . *                         .*      *   :   .

Nf-ref_GHL -----------------------------------SGGDK----------AEYNTAGTKPAKVVACLDGNLHTFYLYHLV-PVRD----------------CYGGYCATTVF*-------
Np2015_GJR -----------------------------------SGNGN----------PEYATSGRKPGKVVACLDGDLNTFYLYHLV-PLQD----------------CYIGYCATNVF*-------
Nc2017_GJR -----------------------------------SGNGK----------AEANTTYRKPSKVVACLDNNDQTFYLYHLV-SVYS----------------CYIAYCATTVF*-------
Ca_GJQS010 -----------------------------------AWHANYR--KG---FRDYSKGYPTPAKIVACPAGS-ETMYLYFLS-PTPD----------------CYLAYCAL*----------
Rgigas_GIV -----------------------------------AWAGK----------ENLSDGLSTKGQVVACQDQDNQVFYLYKLA-PVTD----------------CELAYCAIGA*--------
Labma_GFWO ----------------------------------TAFNGN----------CEVKM---PNGQVRGCPDPRGGVFFVYYLVQPRIS----------------CNWGYCAVGEPPS*-----
Mp2017_GJA -----------------------------------AHRSV------------NSKSYRKSAKVVACYDDDMNIFYLYGLH-NTLY----------------ADAGYCATTGDDKEIV*--
Tisbe_GCIT -----------------------------------HWQSE----------CEMSF----QGKVRTCEDQDNNIFYVYYLK----T----------------SNAPHCQX-----------
Limulus_XM CPKGWIGARCWIPVCEPRCLNGGRCWKPNKCSCPSGWRGR-RCHKATCLVRCRNGGTCVKPNICVCPPG----FKGLHCEEPTCSESCYHGGKCVSPGNCLCYPGYSGINCEISQKKNIH
                                                                        ::  *       :                           .   :.

Limulus_XM KRRYQIYRHRHGYRLQNLFTHVHDA*
```

========================== **SF2B data** ========= index ==========

tBLASTn-scan GHLB01035467 Fig S2B [old 3B]

Top hit	Species	Co	Max score	Total score	Cov.	E-value	% ID	Acc L	Accession
neofl_CV-2015-GAK1-S83R1	Nf DN15108	Nf	296	296	100%	8e-102	100%	568	GHLB01035467.1
nplum-male-2015-R1	Np TR31750	Np	270	270	100%	1e-91	90.97%	579	GJRU01062432.1
n-cris_CV2017_82-S6	Nc DN22932	Nc	266	266	100%	2e-90	89.58%	512	GJRH01042229.1
TSA Megacalanoidea	*C. propinquus*		131	131	99%	4e-37	50.34%	487	GIVX01010805.1
c-marsh_CV2017_n2-62-S4†	DN24902_C0_g1_i4	Ca	117	117	98%	2e-31	46.53%	573	GJRF01060545.1
TSA Eucalanoidea	*R. gigas* [Tar.]	Eu	102	102	95%	1e-25	46.10%	533	GIVD01132990.1
Clc01 - R. gigs-IT	no hit								no hit[18581]
ebungii_CV2017	no hit								
TSA Centropagoidea	no signif	Cn							
TSA Augaptiloidea	*P. xiphias*	Au	134	134	99%	2e-38	51.03%	486	GFCI01260610.1
mpacifica_AF2017	Mp DN18322		84.0	84.0	95%	1e-18	37.91%	498	GJAO01001344.1
TSA Harpacticoida	no signif	Ha							
TSA Cyclopoida	no signif	Cy							
TSA Crustacea - not Cope.	*Pontastacus*	Cr	54.7	54.7	81%	6e-07	33.88%	574	GBEI01040337.1
TSA Arthropoda not Crust.	*Premolis**	Ar	47.0	47.0	54%	8e-04	33.33%	433	GFNN01015450.1
Top NCBI nr/nt	no signif								

Protostome [outgrp] TSA	*Saccostrea*		73.9	73.9	83%	4e-13	35.48%	1065	GGZL01202397.1
Protostome [outgrp] UniPr	*Crassostrea*					3e-12			**tr|K1PJB8**

**Premolis semirufa*is a noctuoid moth

† Note the match is with the alternate *C. marshallae* transcriptome; homolog not present in Cm2018

SEQUENCS:

>**Nf-ref_**GHLB01035467 ***N. flemingeri*** DN15108_c1_g1_i2 144aa; Myristoylation sites in bold C-terminal unknown motif=CGPNYCCC
MGKIILVFSCLLAPTLG**GQKYNL**EEMATTACV**GIAPSG**YMTVVAVRRWCNAVAPDCNTVC
ANAAPFQNGPANDGFTCFNSIHVYKKRPSLGKRPGPASGAESNPPPNVGKYGPIIHKYGF
NQ**GNIDAC**TTRSCGPNYCCCSGTY*

>**Np2015_**GJRU01062432 ***N. plumchrus*** TR31750_c0_g1_i1 .p1 _len:145
MDKIILVLSCLLAPALG**G**P**KYN**LEEMATSACV**GIAPSG**YWTVVAVRRWCNAVAPDCNTVC
NNAAPFQNGPANQGFTCFDSLHVYKKRPSLGKRPGPASGAESNPPPNVGKYGPIIHKYGF
NRP**NIDAC**TTRSCGPNYCCCSATY*

>**Nc2017_**GJRH01042229 ***N. cristatus*** DN22932_c0_g2_i1.p1 _len:145
MGKIILVLTCLLAPAQGGPKYNLEEMATSACVGIAPSGYWTVVAVRRWCNAVAPDCNTVC
KNAVPFQNGPANQGFTCFDSIHVYKKRPSLGKRPGPASGAESNPPPKVGKYGPIIHKYGF
NLPNIDACTTRSCGPNYCCCSATY*

>**Ca_Cm2018_**GJRF01060545 ***C. marshallae*** DN24902_c0_g1_i4.p1 len:151 [partial]
LKSTSKVKETRRIHFKKMKRVIPFLAFLPALVLACYDLDGMAVTACTGLTLAARKIGTVV
AVRRQCKNGVANCKTLCEEADAFNNGAANGGFDCFDSLHVYANRPILSQRTTTGEPTPPI
DAGKYGPIIHRYGSCTSTHCGPNYCCCIGKR*

>**Eu_Rhin_**GIVD01132990 TSA: **Rhincalanus gigas** contig232663.1, [Tarrant]
MNYGTIFLSCLFTLGQASYDLDEMATSACVGLSIGHGKVGTVVAVRKSCLLFVDCRNICA
EFTEHFENGEANGWSCFDALHVYKNRPNPAPNDVEKYGPVIHRYGSCSEAFCGPNYCCCN
GIA*

>**Au_Mp2017_**GJAO01001344 ***Metridia pacifica*** DN18322_c1_g1_i1.p1
MVSMIIVATTLSYFTGVIMASSDSYNYLDEMATSACVGMSVSHGQKGHITAVRRNCSSNY
VDCAQVCTNAATNNPGTGKQKGEWECFDSLHVYKNHPQLSDNSGSYTDSGKVGPVIYRYG
SCAGSYCGPNYCCCVAN*

>**Cr_**GBEI01040337 TSA: **Pontastacus leptodactylus** Pl_CLC_40523 transcribed RNA sequence; partial
KTSVHSEHKDYHAKRKISTRQKMKYTSALAILALLIGAAIAEENESENVYQPVNEKRTDG
RSYSNSLYYNYYDEIATSVCSSVPNRSGMRFAVRRKCPSTSTCQQICTGAKVTAQLQWSW
TKHMTFDCIESLHIYKNRPVLSPDSNADTGKIGLLVWRYHTCNMASCGPNYCCCQKR*

>**Ar_Prem_GFNN01015450**.1_3 TSA: Premolis semirufa Psemirufa15497 transcribed RNA sequence 433nt; 133 aa ORF complete **FIXED;**
MAAKLVYLAITIFAIFSSTEGRFKSYFNNYLDDMAMSICASFNLHKSARMYAVRRKCGEA
LTCQQICTSKNFRKQAAYANIACLESLHVYKQTRELAENSQADTDVHIVGPQIHRYNTCH
TNGCGPNYCCCTS*

Protostome sequence that also provided a satisfactory outgroup, but more distant than *Limulus*
>**Crass_tr|K1PJB8 Crassostrea gigas**|K1PJB8_CRAGI Guanylate cyclase [E=2.6e-12 higher scoring SP outgroups [e-18] all "uncharacterized"

```
MKDILSNDSFTLNWDFRASLVCDIIQAMDYIHNSNIKYHGHLTSMNCVIDSRFVLKVTEF
GIQSLRDFDIDITKKESLWVAPEAIRQQNAVKSIQEMQYADVYSFAVILYEILSRKEPFE
DDQEFLTFKEIIMKIKYVEETPFRPRLDADSDKDMVSLMRICWDENPKSRPTFSTIKKEA
TRLKWDKTGDKFLDNLLSRMEEYANNLEDLVEERTQAFLDEKRRAEELLYQVLPRSVADE
LKNGRMVNPEAFACVTVYFSDIVGFTSLSSESTPMQIVDLLNDLYTCFDKIIENFDVYKV
ETIGDAYMVVSGLPVRNGNKHVEEIAKMSTIILDNVKNFKIRHKPNVQLRARIGLHSGPV
CAGVVGRKMPRYCLFGDTVNTASRMESTGEGAHAFDADAIAQAACAASSPGGFVSAIRRT
CSGTQASCNTICSNAISGMRAIYGNQGSTTGTCFQAFHFYYKHATLKPEEKGKALLAMHR
YRDGCNSTSCGPNFCCCKA
```

NOTES:

1. **Motifs:** The primary *N. flemingeri* sequence had 3 putative myristoylation sites identified by MotifScan. However, these lacked one of the residues in the second position expected according to Maurer-Stroh,-Eisenhaber & Eisenhaber 2002 (EDRHPFYW).

2. **Novel motif:** In addition to the putative myristoylation sites, almost all hits (not the *Premolis* sequence, which was C-terminal truncated) had a justa-C-terminal C-nx-CGPNYCCC motif that was not identified in any of the motif-finder portals. This was also accompanied by a set of 5 conserved cysteine residues marked in blue.

3. Annotation: No SwissProt BLASTx annontations for the homologs among the new calanoid transcritomes; A distant *Crassostrea* homolog annotates as guanylate cyclase

4. **Aberrations:** As in Panel A, the Augaptiloidean peak in the bar graph stood out as well above other distant taxa. The correct taxon's position for Augaptiloidea in the phylogenetic cladogram was maintained by using the *Metridia* sequence instead. It had a much more reasonable (i.e. "expected") similarity index.

5. Absent hits: No hits for *E. bungii*, Centroapagoidea, Cyclopoida or Harpacticoida, which is odd given the broad taxonomic distribution of the C-nx-CGPNYCCC motif

6. Frame shift: The single Arthropod/not-crustacean hit was for the moth *Premolis*. However, owing to a frame shift in the nucleotide sequence, the translated nucleotide sequence did not possess the characteristic C_terminal motif. When the frame-shift was corrected by the insertion of A241 in the nucleotide sequence (GFNN01015450.1) (approximately - exact location unknown) it provided a repaired protein with the expected motif and 6 extra cysteines in place.

7. Ancestral protein: Putative homologs (based on the C-terminal motif) outside of the Arthropoda were longer and possessed a few more cysteines, possibly key to protein folding.

Supplementary Information SD4

========================== **SF2C data** =========== index =========

GHLB01048443 [old 3E]

Above normal charged residues

tBLASTn-scan GHLB01048443_DN8569_c0_g1_i1 [Not among DEGs? - see Panel 6h in SF2x_ExtraBars.pptx]
[old 3E]

Top hit 2022-05-14	Species	Cov.	E-value	% ID	Acc L	Accession	Max score	Total score
neofl_CV-2015-GAK1-S83R1	Nf DN8569	100%	1e-200	70.37%	1656	GHLB01048443.1	586	586
nplum-male-2015-R1	Np TR18067	100%	1e-200	70.14%	1557	GJRU01034954.1	585	585
n-cris_CV2017_82-S6	Nc DN10642	100%	1e-200	69.05%	1579	GJRH01032237.1	572	572
TSA Megacalanoidea	*C. hyperboreus*	100%	6e-166	60.83%	1921	GJRE01249458.1	487	487
c-marsh_CV2018_n1-21-S1	DN20562	100%	2e-155	58.29%	1664	GJRL01081603.1	458	458
TSA Eucalanoidea	*R. gigas*	100%	2e-84	41.23%	2098	GIVD01021832.1	279	279
Clc01	Rgigas-IT		1e-73	42%	1343	DN8630_c0_g1_i1		
ebungii_CV2017	Eb	100%	9e-85	40.00%	1550	GJRG01021917.1	275	275
TSA Centropagoidea	*Eurytemora*	82%	6e-39	30.46%	1538	GBGO01040053.1	154	154
TSA Augaptiloidea	*P. xiphias*	15%	8e-09	42.47%	2450	GFCI01522782.1	66.2	66.2
mpacifica_AF2017	Mp 16575	14%	9e-09	47.62%	2450	GJAO01114355.1	65.9	65.9
TSA Harpacticoida	*Tigriopus*	16%	1e-12	50.00%	4644	JW508314.1	78.2	78.2
TSA Cyclopoida	*Apocyclops*	15%	5e-11	47.30%	1147	GHAJ01020944.1	71.6	71.6
TSA Crustacea - not Cope.	*Triops*	16%	5e-10	43.06%	4076	GEHY01001251.1	71.2	71.2
TSA Arthropoda not Crust.	*Catonia nava*[1]	21%	2e-09	35.48%	2732	GEHT01059744.1	70.9	70.9
Top NCBI nr/nt	*E. affinis* myc proto-oncogene protein-like	82%	8e-40*	30.46%	1575	XM_023468985.1	154	154

```
>Nf-ref_GHLB01048443_Neocalanus flemingeri DN8569_c0_g1_i1  [40K+16R+10H=66+;45D+25E=70 -
/432→ little net charge, but N-terminal is strongly acidic and the C-terminal basic; 334-
384=Myc bHLH motif
METSIHDMSIVPEIGDFDFDITTMDTFDDLDISMENFDIVESASDPFGDFSLEQSLNLVE
LNNVKDLLIDDIYSSGDSDSSSILDDFDIKETIKQDCMWSSAHDTNSKSLNIKHKSRSLL
DSKLSLTPPTSYINQHLQIFDTPIPSDDDSSSNDGSSDEIDVVSGYSETLLKNSCESISS
GDHSYTSGQGKSCSLLTPPESSEDEDSSQGIYISKAAQDILKARKTGLKEIENDRFNKVV
KSILLKSSSKSLSTTSIDKAKFKFSISMNSNSKTSLLRRKSKLDKKSSRTTISSYQGVNE
NKQTLKFSHGFQNKQSNRVTHLDRVNKDKTNHKEARDVHNQMERQRRTDLKNAFDQLKDY
VPTIANSDRASKQMVLDKAIEHCKTLKMKENSVREQRKNIVQRNELLKKKLALLESQIAS
CQVENADWEIQW*
```

>Np2017_GJRU01034954 Neocalanus plumchrus TR18067_c0_g1_i1 Neocalanus plumchrus

```
METSIHDMSIVPEIGDFDFDLTTMDTFDDLDISMENFDIVESASDPFGDFSLEQSLNLVE
LNNVKDLLIDDIYSSGDSDSSSILDDFDIKETIKQDCMWSSAHDTNSKSLNIKHKSRSLL
DNKLSLTPPTSYINQHLQIFDTPIPSDDDSSSNDGSSDEIDVVSGYSETLLKNSCESISS
GDHSYTSGQGKSCSLLTPPESSEDEDSSQGIYISKAAQDILKARKTGLKEIENDRFNKVV
KSILLKSSSKSLSTTSIDKAKFKFSISMNSNSKTSLLRRKSKLDKKSSRTTISSYQGVNE
NKQTLKFSHGFQNKQTNRVTHLDRVNKDKTNHKEARDVHNQMERQRRTDLKNAFDQLKDY
VPTIANSDRASKQMVLDKAIEHCKTLKMKENSVREQRKNIVQRNELLKKKLALLESQIAS
CQVENADWEIQW*
```

>Nc2017_GJRH01032237 *Neocalanus cristatus* DN10642_c0_g1_i1 *Neocalanus cristatus*
METSIHDMSIVPEIGDFDFDLTTMDTFDDLDISMENFDIVESASDPFGDFSLEQSLNLVE
LNNVKDLLIDDIYSSGDSDSSSILDDFDIKETIKQDCMWSSAHDVNSKSLNIKHKSRSLL
DSKLSLTPPTSYINQHLQIFDTPIPSDDDSSSNDGSSDEIDVVSGYSETLLKNSCESISS
GDHSYTSGQGKSSSLLTPPESSEDEDSSQGIYISKAAQDILKARKTGLKEIENDRFNKVV
KSILLKSSSKSLSTTSIDKAKFKFSISMNSNSKTSLLRRKSKLDKKSSRTIISSSYQGVK
ENKQTLKFSHGFQNKETNRVTHLDRVNKDKTNHKEARDVHNQMERQRRTDLKNAFDQLKD
YVPTIANSDRASKQMVLDKAIEHCKTLKMKENSVREQRKNIVQRNELLKKKLALLESQIA
SCQ**VENADWEIQW***

>Cm2018_GJRL01081603 *Calanus marshallae* DN20562_c0_g1_i1 *Calanus marshallae*
METCIHDMSIVPEIGDFDFDLTTMDTFDDLDISMENFDIVDAARDPFGDFSLEQSLNLVE
LNNAKNLLVDDFNSSGDSDGSSFLGDFDIKETIKQDCMWSSAHDINSKSVNINHKSRSQL
DSLLSLTPPTSYINPNLMMFDTPLPSSDDDSSSTDSSSDEIDVVSGFNQSLLKSSIESSS
SGDHCYTSGHGKSSSMLTPPESSEDEDSSHDIFKSKAAQDILKTGKSVLKEIENDRFNKA
FKSILLKSSSKSVSTTKIDKAKFKFSICMNSNKKTSLLRNKSKQDKKSSRTTVSSYQGIK
ENKQTLKFSHGFQNKQHNRVTHLDGASREKSNHKEARDVHNQMERQRRTDLKNAFDQLKD
FVPTIANSDRASKQMVLDKAMDHCKGLKMKEIKVREERKNIVQRNELLKKKLALLESQIA
SCQVENADWEIQGW*

>Eb2017_GJRG01021917 *Eucalanus bungii* DN17891_c0_g1_i1 *Eucalanus bungii*
MEMGLHGFGVVPDIGDFDIDLSAMDTFDDLNIDIDNFSMVEAGGDPFGDFSLEQSLNIVN
ELHNVNDISSDDFKNTSILDDFDLKEPIKKDCMWSSNVQKNAKSPQSVQIKKNNSTLHHT
GALSLTPPSSYINQHLRSFHTPLPSSDDDDSSTNSSDCEVETDTDCSRIHEYSSSSGDHS
YTSDHPSTDCGKALAGMLLTPPESSEDEDTGTNTPFNCSQIQYQQKNGLKEVENDRFNKV
VRSILLKNSHKSKPKTNTEKAKFKISIRMPSDNSSVFKTKSKQEKKGTTSYTSSSRHLTK
SYQNIKENKQRLIKIGYNDKQKLDERNRPNLESRLSEGQEGRDKTSHREARDVHNQMERQ
RRTDLKNAYETLKDFVPTIANSDRASKQMILDKAIEHCKTMKKKEATTRGEKQNLVQKNE
TLKKKLALLQSQLTSCHLENASWEIQGW*

>Eury_GBGO01040053 Eurytemora affinis comp47289_c0_seq1 transcribed RNA sequence
MDFCPRGTVPDIGDLNFSMDSMDTFHDIDIKMEDIEAQEDFHMEHSLSMFEFDTMKDLTL
DIDAVVRSAEKRSILDELDTKDKIRQDCMWSTGQNLAKLFKPDDKADRVKPYTSLLNEEM
SLTPPTSYINEYLKHFETPLPSDEESSCSSGDEIDVVSDWSTVIMEQGCRLSDQTVLTSS
VSSKDHCYTSRNLSCTLTPPESSEDEDSIQGYYSSKPSSPEFKIRNRPHQGVEADRLNLA
VKSIMDSNPSLRKQNSSKAKFTFKIKIKTSLKHSREPRWGSNIHQLSRSEKPRLSVLKTS
ENIERPKDARDLHNYMERQRRTELKNAYDIVKTCVPTISSSDRVSKQMILDKAIEFCRSL
RQTESLAHKQRRMLMEKNTELKKKLKTLQLRASLSH*

>Mp2017_GJAO01114355 Metridia pacifica DN16575_c0_g1_i1, Metridia pacifica
MAPSSDDIWDTLKMENFDVKMENFEVDLQSLKMDNFDIKMEDFDMSGENRFEVNTPPMSP
EWKNDEKDIHELFYNFREKPGFEASAVVPDIGDIEFDPATINDLSDVKFDFDSIMDSFPS
SDNSSDSSNYCNLEELENEEAVRYDCMWSAVGPKPSSTIPTTPQTIITTSTPSRHTSTSP
SRKRERNASVTISSMLEEFLSADDDNLVQTTLSSSPLLSSSPLSTYTCTPVTSNLSSSTN
LACLDTPLYSEVSDLGEMVEISYSDNDADDEIDVVSDASPTCNQSLLLQKSDSRVRVVRS
IKHKQASSSSSTSSQNNNSSDSQSDKLYGDHCYVISNSTPVHRSVDPSILGILTPTESSE
DDDSSSSSSSSNFHTLVKSQHGYKYRYHIRGDAKKRQLVRSSQSLIRKPGSPESSSTASK
SQTNNQTKFKFHMKFKSHKPRSILRQKSQVSQYGKRKAAVKNSFCPNPTQQIRVRQTKVV
PISSLPPPHTSSSLKRSRESKDASATLSKQSRRELQSPTKIQASSPIQPDKNQEVRDLHN
TMERQRRVELKDALEEVKLSVPSIAQSERASKLTILTKATDYCNNLSSRSVKLRRDLANE
KNRQMQLKKKLRALQAEFSKSVVV*

>**Tigca_JW508314** Tigriopus californicus SD_Contig36566.Tcal3 [missing VENADWEI of VENADWEI**QW**
MSISLLKKNLPPACSTTVSLFPEEQLLDPTATGSSSFSFSSSSSLSSSSSSSLSEFCSSP
LGTTQCLDPSDLKMEFLDSLDTCTVGSWGSHNLNLNLMEDQGSFNLWQELEQTTSPSSTG

TPLASPLSSPANSLVDDPLGPHVASASDLMDFMEQADLPQLAEQMLQNNDAFTLLHDLEL
ESLESQDRDSLSVNGGDEAIKADLMWSSTVSNHKRNRDVSLTLSECAEALFKDVDLLGSS
PPLIGISPHNVLGVKTEASDSDHEDEEIDVVSDDSASTVSAYSTGSSFSPTSSTSTYSRS
TIRPASGKVAPAPSRPFNVKAGRSLLRNRQQPQATMPAPRPSVTGHPIVIDHMNGDHCYF
QVRPPVSPASNNGILTPNESSDDEDQILRMEHGTVLQRGQKRKLSPSRLPHQTGANNTSS
NNRSRTNSMSENVKFKFRMKFQSNSPQRRSLLAMNNRHHLKRKSAVNNSHCPPSPIKEGQ
VQEGEGNVGIISPRSMSATPGLKTSSSLSKKARNNSGGSHNGSISNGSTSNGGGIGDTQK
CREIRDLHNSMERQRRVDLRNNFDQLKEVVPELADVDKASKLNILNKASEFCRQLTAADS
RLKRDRETIQSRNLALKRKLQQLMASYPSGSQGSSRSVGRISVFSRH*

>**Apocy_GHAJ01020944** Apocyclops royi TRINITY_DN56014_c0_g1_i2
KSSGSQHSLLAVNNRPHHIRRKSASMNTHCPEPIGHHQLKAEPQKASPVKGGMRNVKSSR
TSSGSSRSAPSNDQKNREIRDLHNSMERQRRVDLRHALDQLKSVVPELADVEKASKLNIL
NKASEYCKLLSGIDAKLRKEAEKEQARNAMLKKKLQAYQNQFGNTVRLSSGRIALVQSRS
SF*

>**Triops_GEHY01001251** Triops newberryi Transcript_001252 transcribed RNA sequence is OK
MTPANMAVFDSDPCFLKDDSAYSTSPGLATGLGPGLGLTPSLLCDWNKVHCDDASLLSKD
ENLFDSIWDGCFDLPTPPISPSRERESSSALGLANDELNFGELDLDLADLLGADDNTDPS
QFFPDILSSEADDAPSIDELLASAAILSTSTTSPTFPQTPDSSFTSSPSPILDTSSTDLL
SASSTTHVTVTSTPFATYPVTKCTREVRELRHDCMWSGLCPSEEHRREKETKKMVSSWHT
DKERRLASTVILSANSLDATLPPFTTTSLSKNVSMSSTSSRFQVCSRPETPSSLSESEYD
HEETVEDHLRSSKQRAQQLNSRLGLSSSSELDLDDEEEDEEEDDQDSRLKPESEDEVDGS
DDSRFLHPGSVQLALRTPTKHAPPQHTVLPDHSYYSHSDHSYHSVKRPGKVVTENLGIQT
PSDSEEEIDVVSLGKPSHAYLTARPSSFVLQHSKNETTTEVARPGGQNPLLVRKMLPANP
SALVRKQLQLAVASAAHQKKSNTSGGMTTGSTESRCVKIGGGASGAMTTVVRRTSGDEAK
SHSSTKRASEAASYTRSVGRPPKRARLSGQEHCSEPSSPRKRSSRAGSDSEDGMPGEKRN
MHNSMERQRRVDLRNAFDHLRALLPDLSSDRAAKVVILKKAASHCHNLAVNEQRLQAEKE
SLLKKQEQLRKKLEQMQRRR*

>**Catonia_GEHT01059744** Catonia nava [hemiptera] breed wildtype voucher P106
SSSSSNSNSTSSSNSSNASNTNNNNSNSDTNSTISNNVNNNKIRTAQLTQQTSTQSSNSSINNNN
NNNSNSQNSNNSSSSSVVQTIEVPSVCGSVRRVIVQKTVGRSLLIKSRTKSTSDGVNNET
NSSNNTSSSSNNNTNSLLTAKNNNNNNTITNNTSVLPHPMPPVSLSRPETPQSLSESEDD
SCQLDFSSVFHNIDNIDNLKPPQGDLWDEVLAAVEHEQANGIEIPPISSDNESNNKVIVN
KSSDHITINNSILLTPTKSVCSSPSSSSSSSPSSSPIKEPSTNNLLAAATTVVSDHSYDK
RAWIRHEEWRLGVQTPSDSEEEIDVVSLFERSRNSSRSSSTSSNHSSSTTLPTNPSAKDQ
EEIQEITTSKLLSASRAVSRKLRSKITTRTKSFHSIGKRRLPSDSEDSKSSTRFSKKPRI
HYKPCKSRFATDSESDSKEKRDMHNNMERMRRIDLRNSFEELRALVPTLANKERAPKVVI
LQDAAGYCTDLKIQSRQLASQVAALRKEQERLRATVSGLRRSLAACR*

NOTES:

1. Oddly high number of charged residues, but acidic pretty much balance basic Acidic ones are concentrated tat the N-terminal ehalf; basic ones in the C-terminal half.

2. Motifs: bHLH at C-terminal end

========================== **SF 2D data** ======= index ==========

GHLB01xxxxxx [old G]

BLAST-scan

Cala emergence, not Neo

Top hit	Species	Cov.	E-value	% ID	Acc L	Accession	Notes
neofl_CV-2015-GAK1-	Nf-ref DN16566	100%	1e-200	100.00%	1426	GHLB01018689.1	

S83R1							
nplum-male-2015-R1	Np TR9451	79%	5e-118	77.43%	2192	GJRU01017700.1	
n-cris_CV2017_82-S6	Nc DN21743	69%	6e-105	57.41%	1487	GJRH01085855.1	
TSA Megacalanoidea	*C. hyperb.*	57%	5e-74	50.20%	915	GJRE01316709.1	
c-marsh_CV2018_n1-21-S1	Cm DN24075	50%	1e-63	50.00%	742	GJRL01078666.1	
		26%	4e-07	33.33%	490	GIVD01142170.1	
		30%	8e-07	29.93%	688	GIVD01105742.1	
TSA Eucalanoidea	*R. gigas*	30%	1e-06	30.61%	1781	GIVD01029981.1	
ebungii_CV2017							
TSA Centropagoidea	*Temora long.*	23%	5e-05	30.48%	4581	~~GINW01427017.1~~*<br>GINW01427017.1<br>GGQN02427017.1	pau<br>same #<br>same as ↑!!
TSA Augaptiloidea	*P. xiphias*	26%	0.001	26.40%	798	GFCI01264398.1	
		31%	6e-09	32.21%	649	GJAO01092585.1	
mpacifica_AF2017	Mp DN25370	30%	1e-08	31.97%	747	GJAO01092583.1	
		31%	7e-08	30.00%	923	GJAO01016368.1	still short
TSA Harpacticoida	no signif						
TSA Cyclopoida	no signif						
**Outgroup selection:**							
TSA Crustacea - not Cope .	*Glyptelasma*	25%	2e-04	31.25%	548	GIJX01023704.1	
TSA Arthropoda not Crust.	*Amblyoma*	13%	0.027	38.60%	792	GFAC01005385.1	
Top NCBI nr/nt	*Erpetoichthys*	25%	6e-04	26.55%	3987	XM_028819743.1	nuc. GTPase SLIP-GC-like

*** Original hit crossed out; gives "not found" error; identical accession # is valid, plus the second DIFFERENT number yields the same sequence from the same group (Ghent) - the duplication & error are enigmatic 2023-01-27**

>**Nf-ref_GHLB01018689_DN16566_c1_g1_i2**.p1 TRINITY_DN16566_c1_g1~~TRINITY_DN16566_c1_g1_i2.p1 ORF type:complete len:412 (+),score=103.00 TRINITY_DN16566_c1_g1_i2:50-1285(+); Prosite IG-like domain 26-130, grey uhighlight
MACLGIFVMWLIVPSMCVQMDWGDNPDLVEESVTIQGREHIKLLVALVGGHIDLECQVLL
TSHPISKISWKLNGASINNSEKYIVESNQDGVFVEEHFKIDNVTEDMDGTTLTCGYAKGN
YADRVEAVLTVFKLDIEGDICKKCTGNLKLIFKESRRSSQDETNMDRRIRAKIAELTFVP
LDEITVEKDEYSVTLPIDIVKKNQEIIAMKPELFKNVVDPSQGGTALIIIFVAMILGIPV
FAYLYHQCCGIGKKHFLHKRKLPWHDIECDDLKEPGFRACREAGCEMCVHINKVSGTNII
KKCCSGGTLEIKDEINCRDNYIVYILSCTKPNCMKQEIRDTKSSGVEMFRKRRKECQHFQ
MTGHTEEDMEFVVVATEGNDIKRQKIRNTLIKTYKIRNDLNEPEAELIQLV*

>**Np2015_GJRU01017700** _2 TSA: **Neocalanus plumchrus** isolate Monoisolate TR9451:c0_g1_i1, transcribed RNA sequence; yellow is region of MAFFT-alignment with Nf_ref
TKLILLDSLLQEIMDMLAEGHCYCNLDWFRNGPNLTTLRETSKYLLVEELMAELEKDRSS
RVILFSGTYGDEFGYSASTTRPRVQDNEQRFKGRGNGSILTTSNAICESLVYEKLMAELT
KDRNSQLIVLTMKWCSASTTRLGVQENEQEKASCRKPKIIVPCTNYRETLARECADYDWD
YLHADDNGSTYQVLYHSFTDVPNNLNEEDEVTATGSTDNGQWKRVFILFLTPPILMLNEE
QNTQMKQRYNSMIEYLLSAQDISANSHDKTTNESIDETVTSQEIKNNEIVNKSANSQERT
TKESVNKSANLQERTTNGSLDNKTVGIGLRAPCGEARRPSASFLFVVLWLLASPVSCVQM
DWGDNPNLVRESKPIQGREHIKLFVVMLGEHIDLECQVLFTSQPIGKISWKLNGATITNS

EKYIVDTNEDGVFVEEHFRVANVTEDMDGSTLTCGYAKGEYADRVEAILTVFKLDIEGDI
CENCNGNLKLTFKESRRSSQNETNVNRRIKAKIAELTNVHLDDITVEKDEYSVTLSLDIV
KRNKETIAMKPELFKNVVDPRQGGTALILGIIIILPFGCSGGTLKIEDPIKCTDTNIVYV
LSCIKKNCMKQHIGETTLSGVEMLSKLKNDCHHFQLKGHNNDDMEFVVVAAHFDSMKREK
TRNNLINTYKINNMNEPEADPLVSIF*CNNSFNN*KNPTKSNRILKNTKIQKIQKIQKIQ
KIQKIQKIQKX

>**Nc2017_GJRH01085855** _6 **TSA: Neocalanus cristatus** isolate Monoisolate TRINITY_DN21743_c0_g6_i1, transcribed RNA sequence; 189*3 nt 3'UTR removed
DRA*TVVEAFLTVSKLDIEGDICETCDGDLKLIFKESRRSSQYETNVDSRIKAKIAELTN
VHLDEITVDSITSSRHPPSKRIEYSVTLALDNVKKNHKIMAMKLEFLKNVGKTGQGATAW
QWILVGVWCLVFGAMILLFGYLYRVLFLPRRNVGSDDLEGPGFRACGEAGCELCVHITKV
LNRASGGTKVVTKVAIHRSGNNIKQKCLSRGTLKIEDQIKCTDKNIVYILSCTKKNCMKQ
DIGETKSSGLEMLRKRRNECQHFQLPGHTEDDMEFVVVASVSYESERQKIRNKLIEPYKI
RNDLNEPETELILFV*

>**Chyp_GJRE01316709** _1 **TSA: Calanus hyperboreus** TRINITY_DN9249_c0_g3_i5_Chype_030, transcribed RNA sequence; 3=prime partial; missing ~130 3' aa's in MAFFT 18*3nt 5' UTR: RPS*YCLIHFYKRKQPDV
MKSNTLRVFFVMSLTLSVMCEQMDWGDNPDLVEEADPIQGRE
HIKLMVVMVGDSIDLECHVAFTSKPVSEIRWKIDGKRETKSDPPIIEAKNGEVFVEDHLK
LDNLTEDMDGSTVSCEYAKGQYGGSVEAVLQVFKLEIETSKDICETCEGDIKLVFKESKT
SSPAEATVDERIKVKIKEMTNLKLDDIKVDNSGYSVTLPIGTAQTNQAILDMNPTLIQDG
TPVSDNLNKCKCDSVPSPRDDASSTFLVPLIIGIATVGVILLLGGIWVSPLRGGKWGWRR
YQGMI

~~>**Rgigas_GIVD01142170**.1_1 TSA: Rhincalanus gigas contig250109.1, transcribed RNA sequence; internal partial but closer to N-terminal than 9981.~~
~~KRFEQEQEVDNVVGLNIISKEAIINPVTESMDSGKIKCEYDNPGGEKGSVNITLSVWKMT~~
~~MEATTDVCENKNGDITVIFEESKQWSVDETNVKNRVQEKIKKDLNITKQLTDVKEKNMFT~~
~~TILSVNIVKGNSHLRTINDNIKYNGTTIPGSLCFAVDGKWEEWX~~

>**Rgigas_GIVD01029981** _1 TSA: **Rhincalanus gigas** contig50368.1, transcribed RNA sequence; yellow BLAST aligned region; underlined extends identity with **GIVD01105742**; 34*3 nt 5'UTR VH**FKDCHLS*YMYES*YW*LKEKLLNRGYKPA; 307*3 3' UTR
MLNDAMKYGLELNREDSLRKVERENKTENRVRYTITFDPKLTPLSPILVKNWKVMLDSDQ
RLKKRFPAPPMACLKRGPNLAEELIRAKVPRPIKQYTTRATVVGFRSCKGGRKQCSLCPL
TGAASDGRTIIQEVKIHHSGEIIQIKETITCKDSFVKYILTCTKPGCKKQYAGLTTRLAY
LRFDEHLASIMDPNTKCPVGLHWQEPGHRFQDLEFIPIEKLGGSRDTAILRQRESDLINR
LDLIRKGLNRQL*

>**Tlong_GINW01427017 TSA: Temora longicornis** TRINITY_DN455883_c1_g1_i1, transcribed RNA sequence 5' partial [truncated purposely - compound protein]
MKNHEYENKVEELKIKVEELFHLRQLLESANSDNENKELELQEKSVLLKNMTTQLKEANT
ILTEKETVLNSLQTELDSVKSQLCQAETENSSKQGNIVRTKEELSQFKEKLNLAEEEIVE
KEKQLEIQETLLSKSKMSLANAHKELKKRDTEIQLKIEEISSITSNYQNAREELTKKDAE
IKKRDVKLTEIGEQLARTEQESSAREMNLRKREEELSLLLQQLEVAKVSLSTAESRLKAR
EEDLIKRNQEIHAVKARLDHTLQESVRKEEELEKLREEKESVQVVDGSPGFAKCDKEGCG
MCAHVSPVNAVKSTVTGQSVGINCAVDCSTRHVVYLITCTKCKKQFVGHTQRSLAEAYLE
HINIVNNRITNQPTGRHFTLPGHNAGHMNVAPIEKVFNRSKDSLKQRESEWIKEFGTLVP
RGMNIKS*

~~>**Mp2017_GJAO01092585** _1 **TSA: Metridia pacifica** isolate Monoisolate TRINITY_DN25370_c0_g1_i2, transcribed RNA sequence~~
~~HQSGDWKNY*KIEQGFRSCRGGRKQCSLCPFTGAASDGRTIIQEVLIHHSGEILSIKENI~~
~~TCKDSFVKYILTCTKPGCKKQYAGLTTRLAYLRFDEHLASIMDPNTKCPVGLHWQEPGHR~~
~~AQDLEFIPIEKLGGSRDPAILRQRESDLINRLDLIRKGLNRQL*NFCLLLPDIFLYFQIM~~

~~FF*RCQKFDENIKENDIN*ICSFFGCDEVEKPNRCEX~~

~~>**Mp2017_GJAO01092583.1** _3 TSA: Metridia pacifica isolate Monoisolate TRINITY_DN25370_c0_g1_i1, transcribed RNA sequence~~
~~SDQRLKQPFPAPPMACLRRGQNLAEELIRAKVPRPLKQYPTRTTVVGFRSCRGGRKQCSL~~
~~CPFTGAASDGRTIIQEVLIHHSGEILSIKENITCKDSFVKYILTCTKPGCKKQYACLTTR~~
~~LAYLRFDEHLASIMDPNTKCPVGLHWQEPGHRAQDLEFIPIEKLGGSRDPAILRQRESDL~~
~~INRLDLIRKGLNRQL*~~

>**Mp2017_GJAO01016368.1** _5 TSA: **Metridia pacifica** isolate Monoisolate TRINITY_DN24246_c0_g5_i1, transcribed RNA sequence; 5'UTR: KLHCLSITL*KK; 3' UTR 37+*3 nt; underlined=conserved; lacking 5' piece wrt Nf-ref; unannotated in SwissProt BLASTx
MLDDTLEAARNLNRDQLLQKVSRGPGEDRVRYIVTYDPRLPAIPAILAQSWQTMVERDPR
LLPIFTKPPQACYKRGPNLANHIIRSKLTQPTTVSTRSTTGAREVGVRSCAQGGRRQGCR
LCVRGHLGVASNHRSVIKEVKIIHSGETIQIKENLSCTTTDVLYILSCTKPGCGKQYGGE
TGRAVYLRYVEHEDDARDPNTTKNIGLHFQLPGHSIKHMEMIPVEKVRGGVAVRKIRELA
MIRKHKLASHLGLNTQA*

>**Ar_Ambl_GFAC01005385**.1_1 TSA: **Amblyomma aureolatum** Ambaur-76 mRNA sequence; 5' partial
DDFGCHAKQLKTSLLLKEYPASIVDDAILKARALDRNKVLSSHKSQDNERQTNLVLTHSS
NMPRVNNILSKHFNIIQQSSHLSLIFNQPLRAVYRRRRNLKDILVKSKTTSNTNRNPGCQ
PCRKPRCKVCSHVRTTNIAKGTFSDFTFRIKETLNCDNKNVVYMLHCSVCGMEYIGQTGT
PFRLRFNNHKAHIHALPNLPFSRHLCLPHHTFESIQVILLQSGFQTSRQREQRESYFIHK
FRTLTHGKNENLGNFAFLKSLPN*

NOTES:

1. Motifs: Prosite IG-like domain, residues 26-130.

Supplementary Information SF1

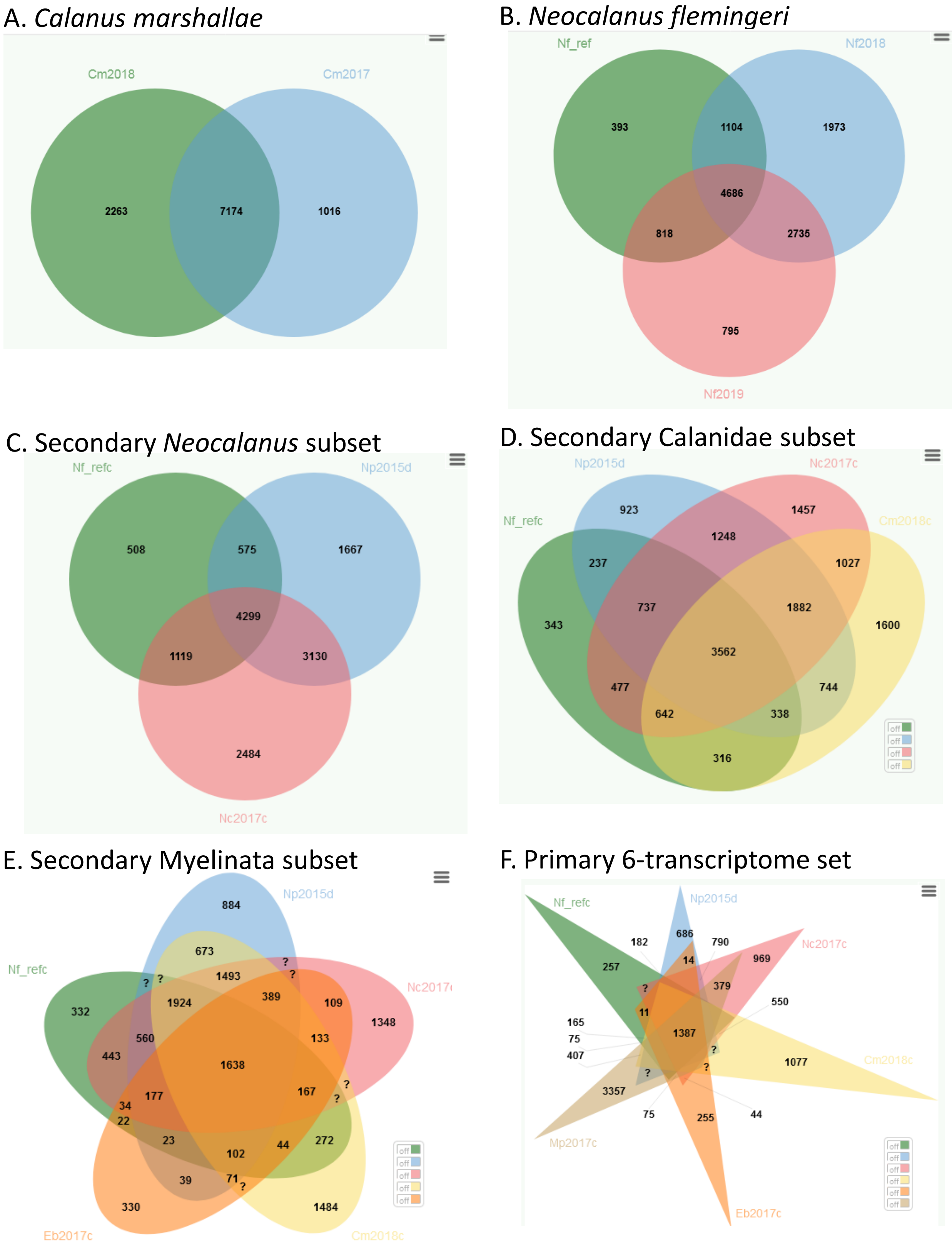

OrthoVenn2 diagrams of homology clusters from Table 5. Numbers inside diagram indicate number of clusters in each field. The number in the central intersection field is the number of clusters containing members from all of the species contributing to the cluster-set (= number in "# in 'all' category of subset" in Table 5). A. Primary homology clusters for 2 *C. marshallae* transcriptomes (Cm2017 and Cm2018); B. Primary homology clusters for 3 *N. flemingeri* transcriptomes; C-E: Secondary hetero-specific cluster subsets (columns 3-5, Table 5) selected from the 6-calanoid primary set of Panel F (column 6). F. Primary homology clusters for 6-species Calanoida transcriptomes.

Supplementary Information SF2 - 3 additional examples of BLAST-scans and cladograms of non-annotated environmentally-reactive proteins

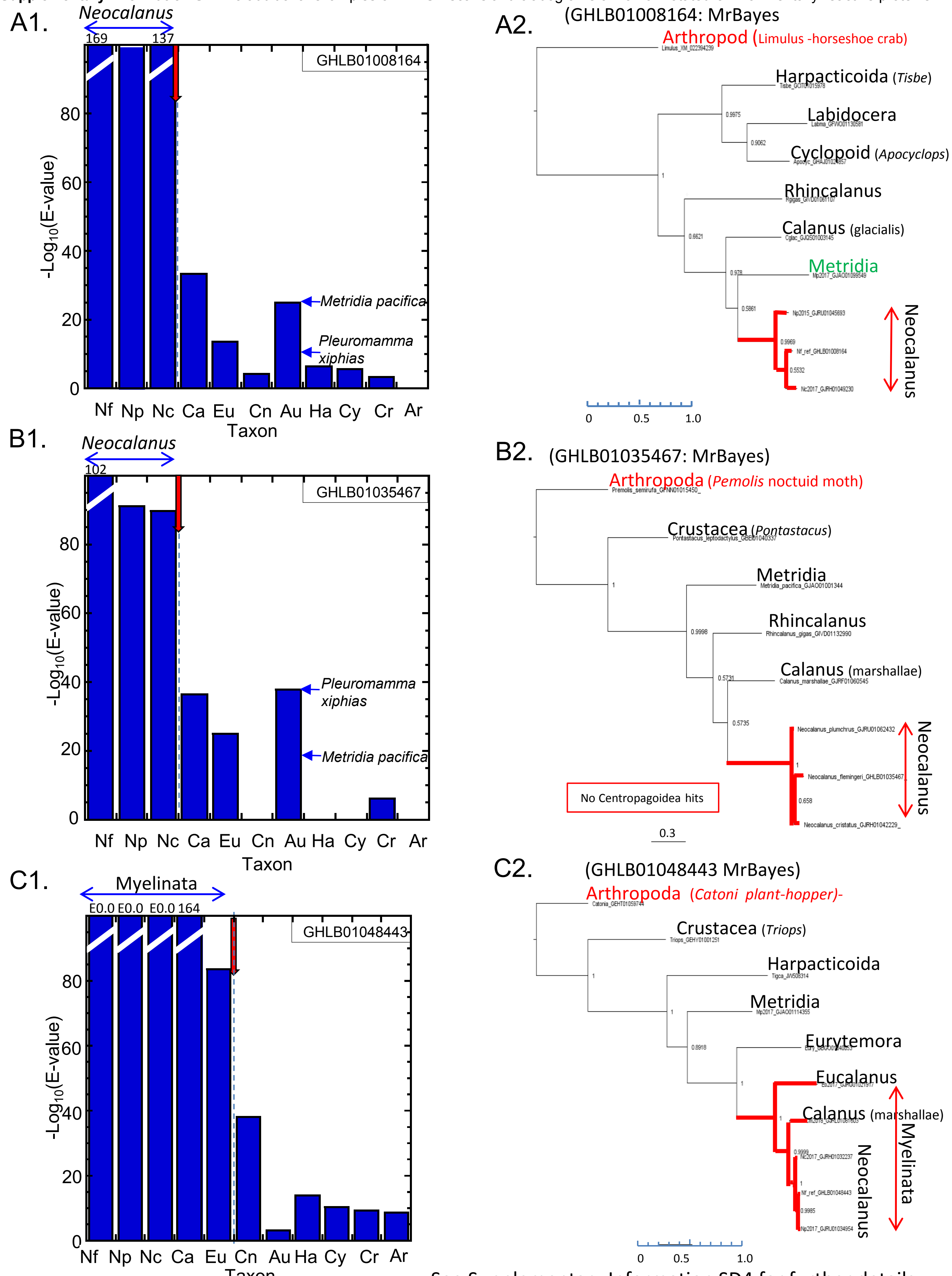

See Supplementary Information SD4 for further details

Supplement ST1
Cluster compositions for 3 taxonomic coverages of Secondary Subsets" Myelinata, Calanidae and Neocalanus (Table 5). Clusters containing transcripts from species outside of a coverage have been purged (see Methods)

Myel_cluster_nameprotein_list
cluster90
Cm2018|GJRL01061096;Cm2018|GJRL01061094;Cm2018|GJRL01061095;Cm2018|GJRL01061098;Nf_ref|GHLB01030079;Nf_ref|GHLB01030075;Nc2017|GJRH01029670;Eb2017|GJRG01013483;Eb2017|GJRG01013482;Eb2017|GJRG01013481;Eb2017|GJRG01013480;Eb2017|GJRG01013477;Eb2017|GJRG01013473;Eb2017|GJRG01013472;Eb2017|GJRG01013492;Eb2017|GJRG01013485;Eb2017|GJRG01013484;Eb2017|GJRG01028361;Eb2017|GJRG01028360;Eb2017|GJRG01028359;Eb2017|GJRG01028362;Np2015e|GJRU01037059;Nf_ref|GHLB01030083;Nf_ref|GHLB01030077;Nc2017|GJRH01075772;Nc2017|GJRH01075769
cluster197
Cm2018|GJRL01060286;Cm2018|GJRL01065745;Cm2018|GJRL01060280;Eb2017|GJRG01036617;Eb2017|GJRG01036618;Eb2017|GJRG01036607;Eb2017|GJRG01036608;Eb2017|GJRG01036612;Eb2017|GJRG01036614;Np2015e|GJRU01010748;Np2015e|GJRU01010747;Np2015e|GJRU01010743;Nf_ref|GHLB01001903;Np2015e|GJRU01010745;Nf_ref|GHLB01001905;Nf_ref|GHLB01001904;Nc2017|GJRH01009272;Nc2017|GJRH01009271;Np2015e|GJRU01067550;Eb2017|GJRG01036609;Eb2017|GJRG01036611;Eb2017|GJRG01036616
cluster199
Cm2018|GJRL01031420;Cm2018|GJRL01032219;Cm2018|GJRL01028369;Cm2018|GJRL01032769;Cm2018|GJRL01032931;Cm2018|GJRL01031411;Cm2018|GJRL01031424;Cm2018|GJRL01059119;Cm2018|GJRL01084451;Cm2018|GJRL01087515;Cm2018|GJRL01028370;Cm2018|GJRL01028371;Eb2017|GJRG01029728;Nc2017|GJRH01005139;Nf_ref|GHLB01033879;Np2015e|GJRU01006862;Eb2017|GJRG01029729;Nc2017|GJRH01005140;Nc2017|GJRH01005131;Nf_ref|GHLB01033885;Nf_ref|GHLB01033891;Nf_ref|GHLB01025039
cluster228
Np2015e|GJRU01062316;Cm2018|GJRL01088831;Cm2018|GJRL01088838;Cm2018|GJRL01088832;Cm2018|GJRL01088835;Cm2018|GJRL01088837;Cm2018|GJRL01088833;Cm2018|GJRL01088834;Cm2018|GJRL01088836;Eb2017|GJRG01020408;Nc2017|GJRH01020498;Nf_ref|GHLB01015281;Nc2017|GJRH01020492;Nc2017|GJRH01020496;Nc2017|GJRH01020494;Eb2017|GJRG01020415;Eb2017|GJRG01020418;Eb2017|GJRG01020420;Nc2017|GJRH01020493;Nc2017|GJRH01020495;Nc2017|GJRH01020497
cluster291
Cm2018|GJRL01017031;Cm2018|GJRL01017021;Cm2018|GJRL01017028;Nf_ref|GHLB01033922;Nf_ref|GHLB01033921;Nf_ref|GHLB01033920;Nf_ref|GHLB01033919;Nf_ref|GHLB01033930;Nf_ref|GHLB01033928;Np2015e|GJRU01043146;Np2015e|GJRU01043144;Np2015e|GJRU01043142;Eb2017|GJRG01036588;Nc2017|GJRH01045663;Nc2017|GJRH01045657;Nf_ref|GHLB01033927;Nf_ref|GHLB01033929;Nf_ref|GHLB01033918;Nf_ref|GHLB01033923;Nf_ref|GHLB01033926
cluster361
Nc2017|GJRH01062067;Nc2017|GJRH01062063;Nf_ref|GHLB01011867;Nf_ref|GHLB01011862;Nf_ref|GHLB01011853;Nf_ref|GHLB01011851;Cm2018|GJRL01050097;Nf_ref|GHLB01011869;Eb2017|GJRG01010106;Np2015e|GJRU01055609;Nf_ref|GHLB01035723;Nc2017|GJRH01053142;Np2015e|GJRU01029564;Nc2017|GJRH01062062;Eb2017|GJRG01010099;Eb2017|GJRG01010107;Nc2017|GJRH01053147;Nc2017|GJRH01053153;Eb2017|GJRG01010104

cluster445
Cm2018|GJRL01087160;Cm2018|GJRL01087162;Cm2018|GJRL01087163;Cm2018|GJRL01087164;Cm2018|GJRL01087165;Cm2018|GJRL01087167;Cm2018|GJRL01087152;Cm2018|GJRL01087155;Cm2018|GJRL01087156;Cm2018|GJRL01087157;Cm2018|GJRL01087158;Np2015e|GJRU01056057;Eb2017|GJRG01033067;Nf_ref|GHLB01035965;Np2015e|GJRU01056056;Nc2017|GJRH01058626;Nc2017|GJRH01058628;Nc2017|GJRH01058625
cluster526
Eb2017|GJRG01003591;Nf_ref|GHLB01002915;Cm2018|GJRL01051558;Cm2018|GJRL01051557;Cm2018|GJRL01051556;Cm2018|GJRL01051555;Cm2018|GJRL01051554;Np2015e|GJRU01038338;Np2015e|GJRU01038337;Np2015e|GJRU01038336;Np2015e|GJRU01038334;Nc2017|GJRH01049249;Nc2017|GJRH01049248;Nc2017|GJRH01049247;Nc2017|GJRH01049246;Nc2017|GJRH01049245;Nc2017|GJRH01049244
cluster533
Cm2018|GJRL01025629;Cm2018|GJRL01025641;Cm2018|GJRL01025642;Cm2018|GJRL01025643;Cm2018|GJRL01025626;Cm2018|GJRL01025645;Cm2018|GJRL01025627;Cm2018|GJRL01025628;Cm2018|GJRL01025630;Cm2018|GJRL01025631;Cm2018|GJRL01025632;Cm2018|GJRL01025633;Cm2018|GJRL01025634;Nf_ref|GHLB01048976;Np2015e|GJRU01033026;Nc2017|GJRH01060123;Eb2017|GJRG01022056
cluster538
Cm2018|GJRL01006941;Nf_ref|GHLB01018285;Np2015e|GJRU01042844;Nc2017|GJRH01053351;Nc2017|GJRH01053356;Nf_ref|GHLB01018286;Nc2017|GJRH01053353;Nc2017|GJRH01053352;Nf_ref|GHLB01018287;Nc2017|GJRH01063767;Eb2017|GJRG01034216;Eb2017|GJRG01034211;Eb2017|GJRG01034206;Np2015e|GJRU01050058;Cm2018|GJRL01011523;Np2015e|GJRU01010951;Eb2017|GJRG01030899
cluster667
Cm2018|GJRL01040114;Cm2018|GJRL01040109;Cm2018|GJRL01040116;Cm2018|GJRL01040126;Eb2017|GJRG01011272;Eb2017|GJRG01011271;Nf_ref|GHLB01005350;Np2015e|GJRU01053765;Np2015e|GJRU01053760;Np2015e|GJRU01053767;Np2015e|GJRU01053761;Np2015e|GJRU01053763;Np2015e|GJRU01053764;Nc2017|GJRH01047957;Nf_ref|GHLB01005348;Nc2017|GJRH01047959
cluster670
Cm2018|GJRL01000290;Cm2018|GJRL01000286;Cm2018|GJRL01000287;Eb2017|GJRG01035774;Eb2017|GJRG01035771;Eb2017|GJRG01035779;Nf_ref|GHLB01045722;Np2015e|GJRU01027816;Nc2017|GJRH01052953;Eb2017|GJRG01035770;Eb2017|GJRG01035769;Eb2017|GJRG01035778;Nf_ref|GHLB01045719;Nf_ref|GHLB01045721;Nf_ref|GHLB01045720;Nf_ref|GHLB01045718
cluster674
Np2015e|GJRU01037084;Eb2017|GJRG01003900;Nf_ref|GHLB01044503;Cm2018|GJRL01018476;Nc2017|GJRH01066459;Np2015e|GJRU01070345;Eb2017|GJRG01003906;Nf_ref|GHLB01023605;Nc2017|GJRH01079353;Eb2017|GJRG01003901;Eb2017|GJRG01003904;Eb2017|GJRG01003907;Cm2018|GJRL01018471;Cm2018|GJRL01018479;Cm2018|GJRL01018474;Cm2018|GJRL01018478
cluster829
Cm2018|GJRL01020901;Cm2018|GJRL01020897;Cm2018|GJRL01020900;Cm2018|GJRL01020905;Eb2017|GJRG01037766;Eb2017|GJRG01037765;Eb2017|GJRG01037764;Eb2017|GJRG01037763;Eb2017|GJRG01037762;Eb2017|GJRG01037761;Eb2017|GJRG01037760;Nf_ref|GHLB01001196;Np2015e|GJRU01000618;Nc2017|GJRH01034888;Nc2017|GJRH01034887
cluster835
Cm2018|GJRL01035314;Cm2018|GJRL01035315;Cm2018|GJRL01035317;Eb2017|GJRG01028224;Eb2017|GJRG01028223;Eb2017|GJRG01028219;Eb2017|GJRG01028222;Eb2017|GJRG01028218;Eb2017|GJRG01028217;Eb2017|GJRG01028220;Np2015e|GJRU0

1037751;Cm2018|GJRL01014169;Nc2017|GJRH01006742;Cm2018|GJRL01014167;Nf_ref|GHLB01046154
cluster840
Cm2018|GJRL01042355;Eb2017|GJRG01002923;Eb2017|GJRG01002917;Nf_ref|GHLB01030520;Np2015e|GJRU01071306;Nc2017|GJRH01017811;Np2015e|GJRU01071304;Np2015e|GJRU01071305;Np2015e|GJRU01071307;Np2015e|GJRU01071308;Np2015e|GJRU01071303;Nf_ref|GHLB01030523;Nf_ref|GHLB01030519;Nf_ref|GHLB01030518;Nc2017|GJRH01017806
cluster980
Eb2017|GJRG01035990;Nf_ref|GHLB01002114;Nf_ref|GHLB01002113;Nf_ref|GHLB01002111;Nf_ref|GHLB01002107;Nf_ref|GHLB01002105;Nf_ref|GHLB01002104;Np2015e|GJRU01071298;Np2015e|GJRU01071296;Np2015e|GJRU01071295;Np2015e|GJRU01071294;Cm2018|GJRL01070625;Np2015e|GJRU01024457;Nc2017|GJRH01084917
cluster1021
Np2015e|GJRU01062458;Np2015e|GJRU01062460;Cm2018|GJRL01083853;Cm2018|GJRL01083849;Cm2018|GJRL01083850;Nf_ref|GHLB01002637;Nc2017|GJRH01034282;Nc2017|GJRH01034279;Cm2018|GJRL01071404;Eb2017|GJRG01032698;Nc2017|GJRH01034284;Nc2017|GJRH01034283;Nf_ref|GHLB01002636;Np2015e|GJRU01007160
cluster1053
Cm2018|GJRL01080798;Cm2018|GJRL01080795;Cm2018|GJRL01080797;Cm2018|GJRL01080800;Cm2018|GJRL01080801;Cm2018|GJRL01080802;Eb2017|GJRG01025491;Nf_ref|GHLB01003504;Nf_ref|GHLB01003503;Nf_ref|GHLB01003502;Nf_ref|GHLB01003501;Np2015e|GJRU01060759;Nc2017|GJRH01063918;Np2015e|GJRU01060758
cluster1069
Np2015e|GJRU01023894;Cm2018|GJRL01075001;Eb2017|GJRG01033044;Nf_ref|GHLB01020123;Nc2017|GJRH01047744;Np2015e|GJRU01023893;Eb2017|GJRG01033041;Eb2017|GJRG01033050;Eb2017|GJRG01033052;Nf_ref|GHLB01020133;Nc2017|GJRH01047740;Nf_ref|GHLB01020119;Eb2017|GJRG01033037;Nf_ref|GHLB01020135
cluster1071
Nc2017|GJRH01076903;Nc2017|GJRH01076905;Nc2017|GJRH01076906;Np2015e|GJRU01058950;Nc2017|GJRH01076907;Cm2018|GJRL01051050;Np2015e|GJRU01058949;Cm2018|GJRL01051051;Nf_ref|GHLB01018849;Nf_ref|GHLB01018851;Eb2017|GJRG01033414;Eb2017|GJRG01033412;Eb2017|GJRG01033411;Nc2017|GJRH01076910
cluster1301
Cm2018|GJRL01082245;Cm2018|GJRL01082238;Cm2018|GJRL01075423;Cm2018|GJRL01075420;Cm2018|GJRL01075422;Cm2018|GJRL01075425;Cm2018|GJRL01075426;Eb2017|GJRG01038099;Nf_ref|GHLB01004183;Nf_ref|GHLB01004182;Np2015e|GJRU01032407;Nc2017|GJRH01013552;Nc2017|GJRH01013553
cluster1326
Nc2017|GJRH01010939;Nc2017|GJRH01010941;Nc2017|GJRH01010946;Cm2018|GJRL01055038;Cm2018|GJRL01055043;Np2015e|GJRU01037442;Np2015e|GJRU01037439;Np2015e|GJRU01037440;Eb2017|GJRG01037198;Nf_ref|GHLB01026090;Eb2017|GJRG01037202;Nf_ref|GHLB01026087;Nf_ref|GHLB01026089
cluster1334
Nc2017|GJRH01004742;Eb2017|GJRG01002223;Np2015e|GJRU01016806;Np2015e|GJRU01016804;Nf_ref|GHLB01018545;Nf_ref|GHLB01018544;Nf_ref|GHLB01018540;Cm2018|GJRL01016729;Nc2017|GJRH01004740;Nc2017|GJRH01004737;Nf_ref|GHLB01018546;Nf_ref|GHLB01018539;Nf_ref|GHLB01018542
cluster1640
Cm2018|GJRL01066234;Cm2018|GJRL01066220;Eb2017|GJRG01009899;Eb2017|GJRG01009895;Eb2017|GJRG01009893;Nf_ref|GHLB01011186;Np2015e|GJRU01065027;Np2015e|GJRU01065026;Nc2017|GJRH01086037;Eb2017|GJRG01009892;Eb2017|GJRG01009896;Eb2017|GJRG01009897

cluster1641
Cm2018|GJRL01007290;Cm2018|GJRL01007291;Cm2018|GJRL01007293;Nf_ref|GHLB01006463;Nf_ref|GHLB01006462;Np2015e|GJRU01055523;Nc2017|GJRH01046905;Eb2017|GJRG01004166;Eb2017|GJRG01004160;Eb2017|GJRG01004159;Eb2017|GJRG01004158;Nc2017|GJRH01046904
cluster1649
Cm2018|GJRL01009339;Cm2018|GJRL01009332;Cm2018|GJRL01009333;Cm2018|GJRL01009334;Eb2017|GJRG01028336;Nf_ref|GHLB01032638;Nf_ref|GHLB01032637;Nf_ref|GHLB01032636;Nf_ref|GHLB01032635;Np2015e|GJRU01048745;Nc2017|GJRH01023110;Nc2017|GJRH01023109
cluster1672
Nc2017|GJRH01052262;Nc2017|GJRH01052265;Np2015e|GJRU01073421;Nf_ref|GHLB01003404;Np2015e|GJRU01073420;Nf_ref|GHLB01003402;Nf_ref|GHLB01003401;Nf_ref|GHLB01003400;Eb2017|GJRG01027715;Cm2018|GJRL01088499;Eb2017|GJRG01038109;Nc2017|GJRH01052264
cluster2008
Cm2018|GJRL01000664;Cm2018|GJRL01000662;Cm2018|GJRL01000663;Eb2017|GJRG01000400;Nf_ref|GHLB01002484;Np2015e|GJRU01070327;Nc2017|GJRH01057572;Np2015e|GJRU01070328;Np2015e|GJRU01070325;Eb2017|GJRG01000399;Np2015e|GJRU01070329
cluster2042
Cm2018|GJRL01051610;Cm2018|GJRL01051609;Eb2017|GJRG01028213;Nf_ref|GHLB01006660;Np2015e|GJRU01012584;Nc2017|GJRH01016682;Eb2017|GJRG01016822;Eb2017|GJRG01027663;Eb2017|GJRG01032729;Nf_ref|GHLB01006657;Eb2017|GJRG01027653
cluster2069
Np2015e|GJRU01008718;Nc2017|GJRH01028684;Np2015e|GJRU01008717;Eb2017|GJRG01010896;Eb2017|GJRG01010893;Nf_ref|GHLB01034156;Cm2018|GJRL01084084;Cm2018|GJRL01084082;Cm2018|GJRL01084083;Eb2017|GJRG01010891;Nf_ref|GHLB01034153
cluster2369
Cm2018|GJRL01043457;Eb2017|GJRG01005977;Cm2018|GJRL01043443;Cm2018|GJRL01043448;Eb2017|GJRG01005975;Cm2018|GJRL01043442;Nf_ref|GHLB01021759;Np2015e|GJRU01045619;Nc2017|GJRH01041908;Nc2017|GJRH01041907
cluster2393
Eb2017|GJRG01004878;Np2015e|GJRU01046652;Nc2017|GJRH01055204;Nc2017|GJRH01055199;Nc2017|GJRH01055202;Nc2017|GJRH01055203;Nf_ref|GHLB01049819;Cm2018|GJRL01085842;Nf_ref|GHLB01049820;Np2015e|GJRU01046653
cluster2475
Nf_ref|GHLB01037604;Cm2018|GJRL01071405;Eb2017|GJRG01011827;Np2015e|GJRU01027928;Nc2017|GJRH01068539;Nc2017|GJRH01068535;Nc2017|GJRH01068525;Nc2017|GJRH01068528;Nc2017|GJRH01068534;Nc2017|GJRH01068518
cluster2543
Cm2018|GJRL01040541;Cm2018|GJRL01040542;Np2015e|GJRU01023886;Eb2017|GJRG01020767;Nf_ref|GHLB01022600;Nc2017|GJRH01042183;Nf_ref|GHLB01022599;Nc2017|GJRH01033882;Nc2017|GJRH01033884;Np2015e|GJRU01015461
cluster2544
Cm2018|GJRL01066234;Cm2018|GJRL01066231;Cm2018|GJRL01066235;Eb2017|GJRG01009897;Eb2017|GJRG01009893;Nf_ref|GHLB01011185;Np2015e|GJRU01065027;Np2015e|GJRU01065025;Nc2017|GJRH01086053;Nc2017|GJRH01086037
cluster2573
Cm2018|GJRL01061005;Cm2018|GJRL01061007;Cm2018|GJRL01061008;Eb2017|GJRG01033565;Np2015e|GJRU01055347;Eb2017|GJRG01004818;Nf_ref|GHLB01004818;Np2015e|GJRU01055346;Nc2017|GJRH01072410;Nf_ref|GHLB01004819

cluster2587
Cm2018|GJRL01050205;Cm2018|GJRL01050204;Eb2017|GJRG01010702;Nf_ref|GHLB01050497;Np2015e|GJRU01031941;Nc2017|GJRH01069730;Eb2017|GJRG01010694;Np2015e|GJRU01031940;Nf_ref|GHLB01050498;Nc2017|GJRH01069742
cluster2589
Cm2018|GJRL01035382;Cm2018|GJRL01035376;Eb2017|GJRG01018169;Nc2017|GJRH01068099;Nc2017|GJRH01068091;Nf_ref|GHLB01046273;Np2015e|GJRU01009891;Np2015e|GJRU01033867;Nf_ref|GHLB01046262;Eb2017|GJRG01018168
cluster2599
Cm2018|GJRL01004261;Eb2017|GJRG01023855;Nf_ref|GHLB01037848;Np2015e|GJRU01068439;Nc2017|GJRH01026281;Nc2017|GJRH01026282;Nf_ref|GHLB01037843;Nf_ref|GHLB01037846;Nc2017|GJRH01028419;Nc2017|GJRH01028425
cluster2602
Cm2018|GJRL01081542;Eb2017|GJRG01034633;Nf_ref|GHLB01032095;Np2015e|GJRU01055268;Nc2017|GJRH01016881;Nc2017|GJRH01016879;Nc2017|GJRH01016878;Nc2017|GJRH01016877;Nc2017|GJRH01016875;Nc2017|GJRH01016874
cluster2611
Cm2018|GJRL01063035;Nf_ref|GHLB01018977;Nc2017|GJRH01071194;Nf_ref|GHLB01018972;Nf_ref|GHLB01018973;Np2015e|GJRU01055453;Np2015e|GJRU01055454;Nc2017|GJRH01039177;Eb2017|GJRG01009086;Eb2017|GJRG01009082
cluster2630
Nc2017|GJRH01036907;Nc2017|GJRH01036906;Eb2017|GJRG01014471;Nf_ref|GHLB01047048;Np2015e|GJRU01050369;Cm2018|GJRL01067373;Np2015e|GJRU01050368;Np2015e|GJRU01050372;Np2015e|GJRU01050373;Eb2017|GJRG01014469
cluster2633
Nc2017|GJRH01021877;Eb2017|GJRG01026369;Eb2017|GJRG01026368;Nf_ref|GHLB01046960;Np2015e|GJRU01017471;Cm2018|GJRL01010456;Np2015e|GJRU01026321;Np2015e|GJRU01026320;Nf_ref|GHLB01022057;Cm2018|GJRL01059399
cluster3221
Cm2018|GJRL01068422;Cm2018|GJRL01068416;Nf_ref|GHLB01002691;Nf_ref|GHLB01002695;Np2015e|GJRU01049640;Nc2017|GJRH01043741;Eb2017|GJRG01019084;Nf_ref|GHLB01002687;Nc2017|GJRH01043744
cluster3227
Cm2018|GJRL01000222;Cm2018|GJRL01000225;Cm2018|GJRL01000223;Eb2017|GJRG01000459;Nf_ref|GHLB01002237;Np2015e|GJRU01041021;Nc2017|GJRH01036053;Nc2017|GJRH01036051;Np2015e|GJRU01041022
cluster3251
Cm2018|GJRL01076576;Cm2018|GJRL01076575;Eb2017|GJRG01027703;Nf_ref|GHLB01001304;Np2015e|GJRU01004033;Nc2017|GJRH01088477;Nc2017|GJRH01088476;Eb2017|GJRG01027709;Eb2017|GJRG01027710
cluster3255
Cm2018|GJRL01058712;Cm2018|GJRL01058711;Eb2017|GJRG01014193;Nf_ref|GHLB01050470;Np2015e|GJRU01042512;Nc2017|GJRH01082929;Nf_ref|GHLB01050469;Nf_ref|GHLB01050468;Nf_ref|GHLB01050471
cluster3258
Cm2018|GJRL01056069;Cm2018|GJRL01056067;Eb2017|GJRG01021547;Nf_ref|GHLB01000387;Nf_ref|GHLB01000386;Nf_ref|GHLB01000384;Nf_ref|GHLB01000383;Np2015e|GJRU01059106;Nc2017|GJRH01002440
cluster3279
Cm2018|GJRL01043319;Cm2018|GJRL01043310;Eb2017|GJRG01029522;Eb2017|GJRG01029520;Nf_ref|GHLB01020184;Np2015e|GJRU01016239;Nc2017|GJRH01069939;Nc2017|GJRH01069937;Nc2017|GJRH01069933
cluster3292
Cm2018|GJRL01009114;Cm2018|GJRL01009113;Eb2017|GJRG01005292;Eb2017|

GJRG01005291;Nf_ref|GHLB01036221;Np2015e|GJRU01062658;Nc2017|GJRH01024062
;Np2015e|GJRU01062653;Np2015e|GJRU01062655
cluster3299
Cm2018|GJRL01033956;Eb2017|GJRG01007345;Eb2017|GJRG01007332;Eb2017|
GJRG01007331;Eb2017|GJRG01007327;Nf_ref|GHLB01004612;Np2015e|GJRU01054053
;Np2015e|GJRU01054052;Nc2017|GJRH01036385
cluster3325
Nc2017|GJRH01073934;Nc2017|GJRH01073932;Eb2017|GJRG01025683;Nf_ref|
GHLB01017912;Np2015e|GJRU01027588;Cm2018|GJRL01029436;Cm2018|GJRL01029435
;Cm2018|GJRL01029439;Eb2017|GJRG01025682
cluster3333
Nc2017|GJRH01038875;Nc2017|GJRH01038873;Nc2017|GJRH01038874;Eb2017|
GJRG01034307;Nf_ref|GHLB01043872;Nf_ref|GHLB01043871;Np2015e|GJRU01001626
;Cm2018|GJRL01038689;Cm2018|GJRL01038688
cluster3349
Nc2017|GJRH01051931;Eb2017|GJRG01021792;Eb2017|GJRG01021791;Nf_ref|
GHLB01016012;Nf_ref|GHLB01016011;Nf_ref|GHLB01016009;Np2015e|GJRU01055253
;Cm2018|GJRL01038605;Cm2018|GJRL01038603
cluster3730
Cm2018|GJRL01058350;Np2015e|GJRU01018318;Cm2018|GJRL01058363;Eb2017
|GJRG01037236;Nf_ref|GHLB01034043;Nf_ref|GHLB01034039;Nc2017|GJRH01007927
;Nc2017|GJRH01007926
cluster3758
Np2015e|GJRU01055685;Cm2018|GJRL01016197;Cm2018|GJRL01016196;Nc2017
|GJRH01049295;Nc2017|GJRH01049294;Nf_ref|GHLB01008897;Nf_ref|GHLB01008898
;Eb2017|GJRG01021890
cluster3932
Eb2017|GJRG01003488;Nf_ref|GHLB01022893;Np2015e|GJRU01022055;Nc2017
|GJRH01055621;Cm2018|GJRL01086678;Cm2018|GJRL01086676;Cm2018|GJRL01086677
;Cm2018|GJRL01086679
cluster3949
Eb2017|GJRG01021542;Eb2017|GJRG01021544;Np2015e|GJRU01022486;Nf_ref
|GHLB01021052;Nc2017|GJRH01038176;Np2015e|GJRU01022487;Cm2018|GJRL0107929
9;Nc2017|GJRH01025702
cluster4079
Cm2018|GJRL01063251;Cm2018|GJRL01063248;Eb2017|GJRG01023728;Nf_ref|
GHLB01030668;Nf_ref|GHLB01030667;Np2015e|GJRU01011090;Nc2017|GJRH01021507
;Nc2017|GJRH01021506
cluster4108
Cm2018|GJRL01008100;Cm2018|GJRL01008099;Cm2018|GJRL01008101;Nf_ref|
GHLB01036994;Np2015e|GJRU01060825;Np2015e|GJRU01060824;Nc2017|GJRH0105987
0;Eb2017|GJRG01011724
cluster4184
Cm2018|GJRL01060776;Eb2017|GJRG01019396;Eb2017|GJRG01019394;Eb2017|
GJRG01019393;Nf_ref|GHLB01019991;Np2015e|GJRU01036873;Nc2017|GJRH01059185
;Np2015e|GJRU01036872
cluster4257
Nc2017|GJRH01016693;Eb2017|GJRG01007649;Nf_ref|GHLB01009141;Np2015e
|GJRU01036682;Np2015e|GJRU01036681;Np2015e|GJRU01036680;Cm2018|GJRL010082
55;Eb2017|GJRG01007648
cluster4258
Nc2017|GJRH01037347;Eb2017|GJRG01002092;Eb2017|GJRG01002090;Eb2017|
GJRG01002089;Eb2017|GJRG01002086;Nf_ref|GHLB01019227;Np2015e|GJRU01031937
;Cm2018|GJRL01044355

cluster4279
Np2015e|GJRU01052139;Eb2017|GJRG01001802;Nf_ref|GHLB01000940;Cm2018|GJRL01058411;Nc2017|GJRH01040069;Eb2017|GJRG01001801;Nf_ref|GHLB01000939;Nc2017|GJRH01040070
cluster4783
Np2015e|GJRU01051910;Cm2018|GJRL01015312;Eb2017|GJRG01002793;Nf_ref|GHLB01040565;Nc2017|GJRH01050504;Nf_ref|GHLB01040564;Nc2017|GJRH01050505
cluster5214
Cm2018|GJRL01053809;Cm2018|GJRL01053810;Cm2018|GJRL01053817;Eb2017|GJRG01015358;Nf_ref|GHLB01019980;Np2015e|GJRU01073527;Nc2017|GJRH01057601
cluster5243
Cm2018|GJRL01016358;Cm2018|GJRL01016357;Eb2017|GJRG01007481;Nf_ref|GHLB01045853;Np2015e|GJRU01020759;Nc2017|GJRH01025556;Nf_ref|GHLB01045852
cluster5299
Cm2018|GJRL01027135;Cm2018|GJRL01027136;Eb2017|GJRG01026847;Nf_ref|GHLB01031805;Np2015e|GJRU01048075;Nc2017|GJRH01082611;Eb2017|GJRG01026848
cluster5311
Eb2017|GJRG01030835;Cm2018|GJRL01031004;Nf_ref|GHLB01037544;Np2015e|GJRU01015284;Nc2017|GJRH01020237;Eb2017|GJRG01030838;Eb2017|GJRG01030839
cluster5362
Cm2018|GJRL01035582;Eb2017|GJRG01028010;Nf_ref|GHLB01043699;Nf_ref|GHLB01043697;Np2015e|GJRU01033086;Nc2017|GJRH01042440;Eb2017|GJRG01028011
cluster5478
Np2015e|GJRU01041304;Eb2017|GJRG01033031;Nf_ref|GHLB01019816;Cm2018|GJRL01041045;Cm2018|GJRL01041032;Nc2017|GJRH01090649;Nc2017|GJRH01090644
cluster5959
Eb2017|GJRG01023646;Cm2018|GJRL01080004;Cm2018|GJRL01080003;Nf_ref|GHLB01002886;Np2015e|GJRU01045591;Nc2017|GJRH01072956
cluster6045
Eb2017|GJRG01014978;Cm2018|GJRL01065758;Cm2018|GJRL01065759;Nf_ref|GHLB01027357;Np2015e|GJRU01037030;Nc2017|GJRH01042543
cluster6047
Eb2017|GJRG01018140;Cm2018|GJRL01081621;Nf_ref|GHLB01006669;Np2015e|GJRU01011708;Nc2017|GJRH01045552;Nc2017|GJRH01045553
cluster6066
Eb2017|GJRG01031675;Np2015e|GJRU01068024;Np2015e|GJRU01068023;Nf_ref|GHLB01007711;Cm2018|GJRL01042513;Nc2017|GJRH01003791
cluster6785
Cm2018|GJRL01067656;Cm2018|GJRL01067664;Eb2017|GJRG01020852;Nf_ref|GHLB01028670;Np2015e|GJRU01040738;Nc2017|GJRH01071126
cluster6795
Cm2018|GJRL01082325;Eb2017|GJRG01037952;Nf_ref|GHLB01027356;Np2015e|GJRU01042893;Nc2017|GJRH01027162;Nc2017|GJRH01027157
cluster6816
Cm2018|GJRL01045762;Eb2017|GJRG01001321;Nf_ref|GHLB01036288;Np2015e|GJRU01032065;Nc2017|GJRH01020229;Eb2017|GJRG01001320
cluster6825
Cm2018|GJRL01071100;Eb2017|GJRG01008847;Nf_ref|GHLB01045862;Np2015e|GJRU01042489;Nc2017|GJRH01019829;Nc2017|GJRH01019828
cluster6862
Cm2018|GJRL01069902;Eb2017|GJRG01000782;Nf_ref|GHLB01035406;Np2015e|GJRU01041517;Nc2017|GJRH01071041;Nc2017|GJRH01071039

cluster6873
Cm2018|GJRL01067696;Eb2017|GJRG01024983;Nf_ref|GHLB01043990;Np2015e|GJRU01064397;Np2015e|GJRU01064396;Nc2017|GJRH01069803
cluster6952
Nc2017|GJRH01000583;Nc2017|GJRH01000582;Eb2017|GJRG01026522;Nf_ref|GHLB01016056;Np2015e|GJRU01036712;Cm2018|GJRL01035445
cluster6980
Nc2017|GJRH01066427;Nc2017|GJRH01066426;Eb2017|GJRG01008545;Nf_ref|GHLB01036240;Np2015e|GJRU01028990;Cm2018|GJRL01017967
cluster6984
Nc2017|GJRH01058456;Nc2017|GJRH01058455;Eb2017|GJRG01000339;Nf_ref|GHLB01031133;Np2015e|GJRU01020373;Cm2018|GJRL01023918
cluster6994
Nc2017|GJRH01057352;Eb2017|GJRG01021430;Eb2017|GJRG01021429;Nf_ref|GHLB01011148;Np2015e|GJRU01057802;Cm2018|GJRL01069571
cluster7012
Nc2017|GJRH01084404;Eb2017|GJRG01031030;Nf_ref|GHLB01000192;Np2015e|GJRU01011365;Cm2018|GJRL01065760;Np2015e|GJRU01011364
cluster7041
Np2015e|GJRU01012334;Np2015e|GJRU01012333;Eb2017|GJRG01022183;Nf_ref|GHLB01003455;Cm2018|GJRL01079973;Nc2017|GJRH01062989
cluster7079
Np2015e|GJRU01003362;Eb2017|GJRG01008871;Nf_ref|GHLB01005605;Cm2018|GJRL01070529;Nc2017|GJRH01065542;Eb2017|GJRG01008867
cluster7085
Np2015e|GJRU01025870;Eb2017|GJRG01007012;Nf_ref|GHLB01004345;Nf_ref|GHLB01004344;Cm2018|GJRL01019481;Nc2017|GJRH01051790
cluster7659
Np2015e|GJRU01024766;Eb2017|GJRG01029687;Nf_ref|GHLB01023503;Cm2018|GJRL01005795;Nc2017|GJRH01002830
cluster7670
Eb2017|GJRG01031835;Np2015e|GJRU01005087;Nf_ref|GHLB01023560;Cm2018|GJRL01077407;Nc2017|GJRH01035067
cluster8427
Cm2018|GJRL01076092;Eb2017|GJRG01019051;Np2015e|GJRU01008657;Nc2017|GJRH01052864;Nf_ref|GHLB01021285
cluster8428
Cm2018|GJRL01037753;Eb2017|GJRG01019061;Nf_ref|GHLB01027415;Np2015e|GJRU01019671;Nc2017|GJRH01050867
cluster8429
Cm2018|GJRL01084263;Eb2017|GJRG01012575;Nf_ref|GHLB01018345;Np2015e|GJRU01028998;Nc2017|GJRH01055153
cluster8485
Cm2018|GJRL01036720;Eb2017|GJRG01008350;Nf_ref|GHLB01036296;Np2015e|GJRU01000155;Nc2017|GJRH01013973
cluster8533
Cm2018|GJRL01001603;Eb2017|GJRG01005026;Nf_ref|GHLB01015102;Np2015e|GJRU01002775;Nc2017|GJRH01065089
cluster8563
Cm2018|GJRL01036121;Eb2017|GJRG01034395;Nf_ref|GHLB01036972;Np2015e|GJRU01014839;Nc2017|GJRH01015233
cluster8731
Nc2017|GJRH01032237;Eb2017|GJRG01021917;Nf_ref|GHLB01048443;Np2015e|GJRU01034954;Cm2018|GJRL01081603

cluster8736
Nc2017|GJRH01024611;Eb2017|GJRG01008714;Nf_ref|GHLB01037049;Np2015e|GJRU01063829;Cm2018|GJRL01053320
cluster8770
Nc2017|GJRH01035864;Eb2017|GJRG01014047;Nf_ref|GHLB01023234;Np2015e|GJRU01006249;Cm2018|GJRL01029222
cluster8896
Np2015e|GJRU01040717;Eb2017|GJRG01034965;Nf_ref|GHLB01003649;Cm2018|GJRL01052796;Nc2017|GJRH01060301
cluster8897
Np2015e|GJRU01025133;Eb2017|GJRG01036765;Nf_ref|GHLB01029994;Cm2018|GJRL01015342;Nc2017|GJRH01052674
cluster8912
Np2015e|GJRU01056239;Eb2017|GJRG01033015;Nf_ref|GHLB01011644;Cm2018|GJRL01073441;Nc2017|GJRH01006018

Cala_cluster_nameprotein_list
cluster43
Cm2018|GJRL01013146;Cm2018|GJRL01013142;Cm2018|GJRL01013143;Cm2018|GJRL01013145;Cm2018|GJRL01013147;Cm2018|GJRL01013152;Cm2018|GJRL01013156;Cm2018|GJRL01013157;Nf_ref|GHLB01019108;Np2015e|GJRU01007443;Np2015e|GJRU01007442;Nc2017|GJRH01033518;Nc2017|GJRH01033517;Nc2017|GJRH01033513;Nc2017|GJRH01033510;Nc2017|GJRH01033524;Nc2017|GJRH01033523;Nc2017|GJRH01033519;Nc2017|GJRH01033526;Nc2017|GJRH01033509;Nc2017|GJRH01033514;Np2015e|GJRU01007445;Np2015e|GJRU01007441;Np2015e|GJRU01007440;Np2015e|GJRU01007439;Nf_ref|GHLB01019115;Nf_ref|GHLB01019113;Nf_ref|GHLB01019110;Nf_ref|GHLB01019111;Nf_ref|GHLB01019109;Nf_ref|GHLB01019116;Nf_ref|GHLB01019107
cluster76
Nc2017|GJRH01079266;Nc2017|GJRH01079260;Nc2017|GJRH01079261;Nc2017|GJRH01079262;Nc2017|GJRH01079263;Nc2017|GJRH01079264;Nc2017|GJRH01079265;Nc2017|GJRH01079267;Nc2017|GJRH01079268;Nc2017|GJRH01079269;Nc2017|GJRH01079251;Nc2017|GJRH01079270;Nc2017|GJRH01079271;Nc2017|GJRH01079272;Nc2017|GJRH01079273;Nc2017|GJRH01079252;Nc2017|GJRH01079253;Nc2017|GJRH01079254;Nc2017|GJRH01079255;Nc2017|GJRH01079256;Nc2017|GJRH01079257;Nc2017|GJRH01079258;Nc2017|GJRH01079259;Nf_ref|GHLB01005920;Np2015e|GJRU01004724;Cm2018|GJRL01016199;Cm2018|GJRL01016200
cluster131
Cm2018|GJRL01055887;Cm2018|GJRL01055880;Cm2018|GJRL01055891;Cm2018|GJRL01055878;Cm2018|GJRL01055879;Cm2018|GJRL01055888;Cm2018|GJRL01055889;Cm2018|GJRL01055890;Cm2018|GJRL01055885;Cm2018|GJRL01055892;Cm2018|GJRL01055877;Nf_ref|GHLB01048951;Nc2017|GJRH01013059;Np2015e|GJRU01060272;Np2015e|GJRU01060270;Nf_ref|GHLB01048953;Nf_ref|GHLB01048952;Np2015e|GJRU01060268;Np2015e|GJRU01060266;Nc2017|GJRH01013057;Nc2017|GJRH01013056;Nc2017|GJRH01013055;Np2015e|GJRU01060273;Np2015e|GJRU01060271
cluster164
Cm2018|GJRL01031029;Cm2018|GJRL01031037;Cm2018|GJRL01031028;Cm2018|GJRL01031030;Cm2018|GJRL01031031;Cm2018|GJRL01031033;Cm2018|GJRL01031034;Cm2018|GJRL01031035;Np2015e|GJRU01021200;Np2015e|GJRU01021199;Nc2017|GJRH01039049;Nf_ref|GHLB01006001;Nf_ref|GHLB01006000;Nf_ref|GHLB01005999;Nf_ref|GHLB01005994;Np2015e|GJRU01021204;Np2015e|GJRU01021201;Nf_ref|GHLB01006002;Nf_ref|GHLB01005998;Nf_ref|GHLB01005996;Nf_ref|GHLB01005995;Nc2017|GJRH01039048;Nf_ref|GHLB01006003

cluster201
Cm2018|GJRL01070947;Cm2018|GJRL01070945;Cm2018|GJRL01070946;Cm2018|GJRL01070949;Cm2018|GJRL01070950;Cm2018|GJRL01070936;Cm2018|GJRL01070937;Cm2018|GJRL01070939;Cm2018|GJRL01070940;Cm2018|GJRL01070941;Cm2018|GJRL01070942;Nf_ref|GHLB01049128;Np2015e|GJRU01052280;Np2015e|GJRU01052279;Np2015e|GJRU01052278;Np2015e|GJRU01052277;Nc2017|GJRH01036678;Nc2017|GJRH01036676;Nc2017|GJRH01036669;Nc2017|GJRH01036667;Nc2017|GJRH01036681;Nc2017|GJRH01036679
cluster235
Cm2018|GJRL01040309;Nf_ref|GHLB01021705;Nf_ref|GHLB01021704;Np2015e|GJRU01072683;Nc2017|GJRH01056395;Nc2017|GJRH01056394;Nc2017|GJRH01056392;Nc2017|GJRH01056389;Nc2017|GJRH01056400;Nc2017|GJRH01056396;Nc2017|GJRH01056397;Nc2017|GJRH01056398;Nc2017|GJRH01056399;Nc2017|GJRH01056401;Nc2017|GJRH01056387;Nc2017|GJRH01056388;Nc2017|GJRH01056390;Nc2017|GJRH01056391;Nc2017|GJRH01056393;Nf_ref|GHLB01021703;Np2015e|GJRU01072682
cluster292
Cm2018|GJRL01080052;Nf_ref|GHLB01007780;Np2015e|GJRU01070238;Np2015e|GJRU01070237;Np2015e|GJRU01070236;Np2015e|GJRU01070235;Np2015e|GJRU01070234;Np2015e|GJRU01070233;Np2015e|GJRU01070232;Np2015e|GJRU01070231;Np2015e|GJRU01070230;Nc2017|GJRH01076805;Nc2017|GJRH01076804;Nc2017|GJRH01076802;Nc2017|GJRH01076801;Nc2017|GJRH01076799;Nc2017|GJRH01076798;Nc2017|GJRH01076809;Nc2017|GJRH01076808;Nc2017|GJRH01076807
cluster356
Cm2018|GJRL01052070;Cm2018|GJRL01052078;Cm2018|GJRL01052080;Cm2018|GJRL01052068;Cm2018|GJRL01052069;Cm2018|GJRL01052071;Cm2018|GJRL01052072;Cm2018|GJRL01052073;Cm2018|GJRL01052075;Nf_ref|GHLB01040122;Nf_ref|GHLB01040119;Nf_ref|GHLB01040116;Np2015e|GJRU01015119;Nc2017|GJRH01002233;Nc2017|GJRH01002229;Nc2017|GJRH01002228;Nc2017|GJRH01002225;Nc2017|GJRH01002224;Nc2017|GJRH01002220
cluster359
Cm2018|GJRL01049638;Cm2018|GJRL01049650;Cm2018|GJRL01049649;Np2015e|GJRU01054483;Nc2017|GJRH01062239;Nc2017|GJRH01062253;Nc2017|GJRH01062250;Nc2017|GJRH01062249;Nc2017|GJRH01062241;Nc2017|GJRH01062240;Nf_ref|GHLB01045090;Nc2017|GJRH01062243;Nc2017|GJRH01062251;Nc2017|GJRH01062246;Np2015e|GJRU01054485;Nc2017|GJRH01062254;Nc2017|GJRH01062256;Nc2017|GJRH01062245;Np2015e|GJRU01054481
cluster442
Cm2018|GJRL01033584;Cm2018|GJRL01033578;Cm2018|GJRL01033585;Cm2018|GJRL01033586;Cm2018|GJRL01033587;Cm2018|GJRL01033588;Nf_ref|GHLB01028285;Np2015e|GJRU01019299;Nc2017|GJRH01056005;Nf_ref|GHLB01028288;Nc2017|GJRH01033050;Nc2017|GJRH01033049;Np2015e|GJRU01044319;Np2015e|GJRU01044313;Nf_ref|GHLB01014898;Nf_ref|GHLB01014897;Nf_ref|GHLB01014900;Nf_ref|GHLB01014901
cluster662
Cm2018|GJRL01007552;Cm2018|GJRL01007547;Cm2018|GJRL01007550;Cm2018|GJRL01007556;Cm2018|GJRL01007557;Cm2018|GJRL01007559;Cm2018|GJRL01007551;Cm2018|GJRL01007555;Cm2018|GJRL01007553;Nf_ref|GHLB01012081;Np2015e|GJRU01015801;Nc2017|GJRH01027074;Np2015e|GJRU01015808;Np2015e|GJRU01015805;Nc2017|GJRH01018523;Nc2017|GJRH01018509
cluster668
Cm2018|GJRL01078780;Cm2018|GJRL01078771;Cm2018|GJRL01078786;Cm2018|GJRL01078782;Cm2018|GJRL01078766;Cm2018|GJRL01078772;Cm2018|GJRL01078774;Cm2018|GJRL01078776;Np2015e|GJRU01009839;Np2015e|GJRU01009835;Nf_ref|GHLB

01048203;Nf_ref|GHLB01048202;Nc2017|GJRH01042497;Np2015e|GJRU01009837;Np2015e|GJRU01009836;Np2015e|GJRU01009834
cluster669
Cm2018|GJRL01004581;Cm2018|GJRL01004594;Cm2018|GJRL01004597;Cm2018|GJRL01004598;Cm2018|GJRL01004585;Cm2018|GJRL01004586;Cm2018|GJRL01004587;Cm2018|GJRL01004588;Nf_ref|GHLB01024006;Np2015e|GJRU01061996;Nc2017|GJRH01009207;Np2015e|GJRU01061995;Np2015e|GJRU01061998;Np2015e|GJRU01062000;Np2015e|GJRU01062001;Nc2017|GJRH01009210
cluster825
Cm2018|GJRL01041638;Cm2018|GJRL01041633;Cm2018|GJRL01041635;Cm2018|GJRL01041636;Cm2018|GJRL01041637;Cm2018|GJRL01041626;Nf_ref|GHLB01046449;Np2015e|GJRU01013347;Nc2017|GJRH01067260;Np2015e|GJRU01013344;Np2015e|GJRU01013343;Np2015e|GJRU01013342;Np2015e|GJRU01013354;Np2015e|GJRU01013353;Nf_ref|GHLB01046447
cluster833
Cm2018|GJRL01012765;Cm2018|GJRL01012754;Cm2018|GJRL01012757;Cm2018|GJRL01012759;Cm2018|GJRL01012760;Cm2018|GJRL01012762;Cm2018|GJRL01012763;Cm2018|GJRL01012766;Nf_ref|GHLB01042639;Np2015e|GJRU01040984;Nc2017|GJRH01036534;Nc2017|GJRH01036531;Np2015e|GJRU01040983;Nf_ref|GHLB01042641;Nf_ref|GHLB01042638
cluster837
Cm2018|GJRL01082152;Cm2018|GJRL01082149;Np2015e|GJRU01065915;Np2015e|GJRU01065913;Np2015e|GJRU01065911;Np2015e|GJRU01065910;Np2015e|GJRU01065909;Np2015e|GJRU01065908;Nf_ref|GHLB01037404;Nf_ref|GHLB01037400;Np2015e|GJRU01065912;Nc2017|GJRH01060832;Nc2017|GJRH01060831;Nf_ref|GHLB01037406;Nf_ref|GHLB01037401
cluster839
Cm2018|GJRL01002153;Cm2018|GJRL01002152;Nf_ref|GHLB01022617;Nf_ref|GHLB01022607;Nf_ref|GHLB01022606;Np2015e|GJRU01052744;Np2015e|GJRU01052743;Np2015e|GJRU01052742;Nc2017|GJRH01005812;Nc2017|GJRH01005807;Nf_ref|GHLB01022608;Np2015e|GJRU01052746;Nf_ref|GHLB01022621;Nf_ref|GHLB01022625;Nf_ref|GHLB01022615
cluster979
Np2015e|GJRU01043966;Np2015e|GJRU01043965;Np2015e|GJRU01043964;Cm2018|GJRL01076590;Cm2018|GJRL01076594;Nf_ref|GHLB01048096;Nf_ref|GHLB01048095;Nc2017|GJRH01041726;Nc2017|GJRH01041734;Nc2017|GJRH01041735;Nc2017|GJRH01041727;Nc2017|GJRH01041729;Nc2017|GJRH01041731;Nc2017|GJRH01041733
cluster1044
Nf_ref|GHLB01004061;Np2015e|GJRU01026585;Np2015e|GJRU01026573;Nf_ref|GHLB01004060;Cm2018|GJRL01018454;Cm2018|GJRL01018459;Cm2018|GJRL01018460;Cm2018|GJRL01018451;Cm2018|GJRL01018453;Cm2018|GJRL01018456;Nc2017|GJRH01028508;Nc2017|GJRH01028505;Nc2017|GJRH01028501;Nc2017|GJRH01028499
cluster1049
Cm2018|GJRL01005510;Cm2018|GJRL01005511;Cm2018|GJRL01005514;Cm2018|GJRL01005515;Cm2018|GJRL01005516;Cm2018|GJRL01005517;Cm2018|GJRL01005518;Nf_ref|GHLB01036846;Nf_ref|GHLB01036847;Np2015e|GJRU01035388;Nc2017|GJRH01004457;Nc2017|GJRH01004459;Nc2017|GJRH01004458;Nc2017|GJRH01004456
cluster1051
Cm2018|GJRL01070961;Cm2018|GJRL01070958;Cm2018|GJRL01070959;Cm2018|GJRL01070960;Cm2018|GJRL01070962;Np2015e|GJRU01063493;Nc2017|GJRH01087139;Nf_ref|GHLB01004627;Nf_ref|GHLB01004625;Nf_ref|GHLB01004623;Np2015e|GJRU01063492;Nc2017|GJRH01087137;Nc2017|GJRH01087138;Nf_ref|GHLB01004624
cluster1063
Cm2018|GJRL01009908;Cm2018|GJRL01009909;Nc2017|GJRH01024890;Nc2017|

GJRH01024889;Nc2017|GJRH01024892;Nc2017|GJRH01024897;Nc2017|GJRH01024899;Nf_ref|GHLB01018307;Np2015e|GJRU01038426;Np2015e|GJRU01038442;Nf_ref|GHLB01018313;Nf_ref|GHLB01018308;Nf_ref|GHLB01018315;Nf_ref|GHLB01018305
cluster1222
Cm2018|GJRL01088886;Cm2018|GJRL01088889;Cm2018|GJRL01088890;Cm2018|GJRL01088881;Cm2018|GJRL01088882;Cm2018|GJRL01088884;Cm2018|GJRL01088885;Np2015e|GJRU01007102;Np2015e|GJRU01007100;Np2015e|GJRU01007099;Nf_ref|GHLB01045738;Nc2017|GJRH01033272;Np2015e|GJRU01007101
cluster1297
Cm2018|GJRL01065014;Cm2018|GJRL01042788;Cm2018|GJRL01065013;Cm2018|GJRL01042786;Cm2018|GJRL01042787;Nc2017|GJRH01070777;Nc2017|GJRH01070771;Nc2017|GJRH01070773;Nc2017|GJRH01070774;Np2015e|GJRU01004068;Np2015e|GJRU01004069;Nf_ref|GHLB01014127;Np2015e|GJRU01004067
cluster1305
Cm2018|GJRL01040725;Cm2018|GJRL01040721;Cm2018|GJRL01040722;Cm2018|GJRL01040723;Cm2018|GJRL01040724;Cm2018|GJRL01040726;Cm2018|GJRL01040727;Np2015e|GJRU01025624;Nf_ref|GHLB01003446;Nc2017|GJRH01010562;Nc2017|GJRH01010554;Nf_ref|GHLB01003436;Nf_ref|GHLB01003433
cluster1308
Cm2018|GJRL01025521;Cm2018|GJRL01025512;Cm2018|GJRL01025515;Cm2018|GJRL01025517;Cm2018|GJRL01025518;Cm2018|GJRL01025520;Cm2018|GJRL01025522;Nf_ref|GHLB01042860;Np2015e|GJRU01032902;Np2015e|GJRU01032900;Np2015e|GJRU01032899;Np2015e|GJRU01032901;Nc2017|GJRH01081720
cluster1312
Cm2018|GJRL01008531;Cm2018|GJRL01008529;Cm2018|GJRL01008530;Nf_ref|GHLB01041758;Np2015e|GJRU01036451;Nc2017|GJRH01023646;Nc2017|GJRH01023644;Nf_ref|GHLB01041759;Nf_ref|GHLB01041757;Nc2017|GJRH01023643;Nc2017|GJRH01023642;Nc2017|GJRH01023641;Nc2017|GJRH01023645
cluster1518
Nc2017|GJRH01013605;Nc2017|GJRH01013607;Nc2017|GJRH01013603;Nc2017|GJRH01013604;Nc2017|GJRH01013608;Nc2017|GJRH01013615;Nc2017|GJRH01013619;Cm2018|GJRL01041824;Np2015e|GJRU01047005;Nf_ref|GHLB01014253;Np2015e|GJRU01047006;Cm2018|GJRL01041830
cluster1531
Nf_ref|GHLB01021940;Np2015e|GJRU01033831;Nc2017|GJRH01066768;Np2015e|GJRU01033830;Nf_ref|GHLB01021938;Cm2018|GJRL01015335;Cm2018|GJRL01015334;Cm2018|GJRL01015338;Cm2018|GJRL01015339;Cm2018|GJRL01015340;Nf_ref|GHLB01021942;Nf_ref|GHLB01021933
cluster1618
Nc2017|GJRH01071925;Nc2017|GJRH01071919;Nc2017|GJRH01071921;Nc2017|GJRH01071922;Nc2017|GJRH01071924;Nc2017|GJRH01071926;Nc2017|GJRH01071923;Nc2017|GJRH01071927;Nf_ref|GHLB01002498;Cm2018|GJRL01069323;Np2015e|GJRU01038011;Nf_ref|GHLB01002489
cluster1634
Cm2018|GJRL01038607;Cm2018|GJRL01038609;Cm2018|GJRL01038611;Nf_ref|GHLB01007143;Nf_ref|GHLB01007141;Np2015e|GJRU01019583;Np2015e|GJRU01019581;Nc2017|GJRH01082911;Nc2017|GJRH01082927;Np2015e|GJRU01019587;Np2015e|GJRU01019585;Np2015e|GJRU01019582
cluster1639
Cm2018|GJRL01001018;Cm2018|GJRL01001019;Nf_ref|GHLB01047756;Np2015e|GJRU01036054;Nc2017|GJRH01020705;Nc2017|GJRH01020703;Nc2017|GJRH01020701;Nc2017|GJRH01020700;Nc2017|GJRH01020693;Nc2017|GJRH01020690;Nf_ref|GHLB01047758;Nf_ref|GHLB01047935

cluster1662
Cm2018|GJRL01026335;Cm2018|GJRL01026332;Cm2018|GJRL01026333;Cm2018|GJRL01026334;Np2015e|GJRU01032060;Np2015e|GJRU01032061;Nf_ref|GHLB01036217;Nc2017|GJRH01086162;Nc2017|GJRH01086158;Nf_ref|GHLB01036216;Nf_ref|GHLB01036211;Nc2017|GJRH01086161
cluster1664
Cm2018|GJRL01060582;Nf_ref|GHLB01011164;Np2015e|GJRU01062133;Np2015e|GJRU01062132;Nc2017|GJRH01023038;Np2015e|GJRU01062135;Np2015e|GJRU01062136;Np2015e|GJRU01062138;Nc2017|GJRH01023035;Nf_ref|GHLB01011163;Nf_ref|GHLB01011165;Nf_ref|GHLB01011166
cluster1980
Np2015e|GJRU01042916;Nc2017|GJRH01031819;Np2015e|GJRU01042915;Cm2018|GJRL01009217;Cm2018|GJRL01009215;Nf_ref|GHLB01036940;Nc2017|GJRH01031817;Nc2017|GJRH01031820;Nc2017|GJRH01031818;Nf_ref|GHLB01036941;Nf_ref|GHLB01036942
cluster2007
Cm2018|GJRL01014616;Cm2018|GJRL01014619;Nf_ref|GHLB01032218;Np2015e|GJRU01044449;Nc2017|GJRH01064979;Nc2017|GJRH01064977;Np2015e|GJRU01044450;Nc2017|GJRH01064975;Nc2017|GJRH01064976;Nc2017|GJRH01064978;Np2015e|GJRU01044451
cluster2015
Cm2018|GJRL01054948;Cm2018|GJRL01054951;Cm2018|GJRL01054950;Cm2018|GJRL01054952;Nc2017|GJRH01091572;Np2015e|GJRU01019159;Nc2017|GJRH01022333;Nf_ref|GHLB01011506;Np2015e|GJRU01048207;Np2015e|GJRU01019158;Nf_ref|GHLB01011505
cluster2017
Cm2018|GJRL01007640;Cm2018|GJRL01007635;Cm2018|GJRL01007636;Cm2018|GJRL01007638;Cm2018|GJRL01007637;Nf_ref|GHLB01016629;Nf_ref|GHLB01016627;Np2015e|GJRU01016729;Nc2017|GJRH01008791;Nf_ref|GHLB01016628;Np2015e|GJRU01016725
cluster2024
Cm2018|GJRL01054426;Cm2018|GJRL01054429;Cm2018|GJRL01054433;Nf_ref|GHLB01002864;Np2015e|GJRU01031822;Nc2017|GJRH01081722;Nc2017|GJRH01081721;Nc2017|GJRH01081723;Nf_ref|GHLB01002856;Nf_ref|GHLB01002861;Nc2017|GJRH01081724
cluster2025
Cm2018|GJRL01063961;Cm2018|GJRL01063959;Cm2018|GJRL01063962;Cm2018|GJRL01063965;Nc2017|GJRH01022662;Nc2017|GJRH01022661;Nc2017|GJRH01022660;Nc2017|GJRH01022659;Nf_ref|GHLB01026180;Np2015e|GJRU01062852;Np2015e|GJRU01062851
cluster2032
Cm2018|GJRL01065302;Cm2018|GJRL01065301;Cm2018|GJRL01022156;Cm2018|GJRL01022159;Cm2018|GJRL01022174;Np2015e|GJRU01023420;Np2015e|GJRU01023419;Nc2017|GJRH01011548;Nc2017|GJRH01011559;Nf_ref|GHLB01027288;Np2015e|GJRU01023418
cluster2039
Cm2018|GJRL01005182;Cm2018|GJRL01005184;Cm2018|GJRL01005185;Cm2018|GJRL01005186;Cm2018|GJRL01005175;Cm2018|GJRL01005176;Cm2018|GJRL01005181;Nf_ref|GHLB01025721;Np2015e|GJRU01017735;Nc2017|GJRH01091595;Nc2017|GJRH01091594
cluster2049
Cm2018|GJRL01019057;Nf_ref|GHLB01019616;Np2015e|GJRU01066084;Nc2017|GJRH01075937;Np2015e|GJRU01066081;Np2015e|GJRU01066083;Nc2017|GJRH010759

39;Nc2017|GJRH01075940;Np2015e|GJRU01066082;Np2015e|GJRU01066085;Nc2017|G
JRH01075942
cluster2053
Cm2018|GJRL01055700;Nf_ref|GHLB01019360;Nf_ref|GHLB01019359;Np2015e
|GJRU01021616;Np2015e|GJRU01021614;Nc2017|GJRH01066720;Np2015e|GJRU010216
13;Np2015e|GJRU01021615;Nf_ref|GHLB01019354;Nf_ref|GHLB01019358;Np2015e|G
JRU01021617
cluster2059
Nc2017|GJRH01076643;Nc2017|GJRH01076641;Nc2017|GJRH01076642;Np2015e
|GJRU01033584;Np2015e|GJRU01033580;Cm2018|GJRL01055000;Nf_ref|GHLB0101288
3;Cm2018|GJRL01054995;Np2015e|GJRU01033581;Np2015e|GJRU01033583;Nf_ref|GH
LB01012884
cluster2538
Cm2018|GJRL01038368;Cm2018|GJRL01038361;Nf_ref|GHLB01050046;Np2015e
|GJRU01055665;Nc2017|GJRH01085255;Np2015e|GJRU01055664;Nf_ref|GHLB0105004
3;Nf_ref|GHLB01050053;Nc2017|GJRH01085253;Nf_ref|GHLB01050048
cluster2540
Cm2018|GJRL01073923;Cm2018|GJRL01073919;Cm2018|GJRL01073921;Cm2018|
GJRL01073925;Nf_ref|GHLB01016651;Nf_ref|GHLB01016649;Nf_ref|GHLB01016646;
Np2015e|GJRU01045704;Nc2017|GJRH01083845;Nc2017|GJRH01083844
cluster2551
Cm2018|GJRL01035229;Cm2018|GJRL01085638;Cm2018|GJRL01035230;Nc2017|
GJRH01002065;Nf_ref|GHLB01012766;Np2015e|GJRU01064234;Np2015e|GJRU0106423
3;Nf_ref|GHLB01012767;Nc2017|GJRH01002059;Np2015e|GJRU01003814
cluster2556
Cm2018|GJRL01002645;Cm2018|GJRL01002646;Cm2018|GJRL01002648;Cm2018|
GJRL01002654;Nc2017|GJRH01072851;Nf_ref|GHLB01030844;Nf_ref|GHLB01030843;
Np2015e|GJRU01057864;Np2015e|GJRU01057863;Nc2017|GJRH01072854
cluster2569
Cm2018|GJRL01046303;Cm2018|GJRL01046305;Np2015e|GJRU01050505;Np2015
e|GJRU01050503;Np2015e|GJRU01050502;Nc2017|GJRH01079396;Nc2017|GJRH010793
94;Nc2017|GJRH01079393;Np2015e|GJRU01050501;Nf_ref|GHLB01036930
cluster2572
Cm2018|GJRL01076719;Cm2018|GJRL01076718;Cm2018|GJRL01076720;Nf_ref|
GHLB01007682;Nc2017|GJRH01028950;Np2015e|GJRU01038152;Np2015e|GJRU0103814
6;Np2015e|GJRU01038158;Nf_ref|GHLB01007678;Np2015e|GJRU01038147
cluster2578
Cm2018|GJRL01065280;Cm2018|GJRL01065278;Cm2018|GJRL01065279;Cm2018|
GJRL01065283;Np2015e|GJRU01043420;Nf_ref|GHLB01009810;Nf_ref|GHLB01009809
;Nc2017|GJRH01073722;Nc2017|GJRH01073725;Np2015e|GJRU01043419
cluster2584
Cm2018|GJRL01022949;Cm2018|GJRL01022947;Cm2018|GJRL01022953;Nf_ref|
GHLB01004675;Np2015e|GJRU01029232;Nc2017|GJRH01065413;Nc2017|GJRH01065405
;Nc2017|GJRH01065400;Nc2017|GJRH01065399;Nc2017|GJRH01065393
cluster2605
Cm2018|GJRL01020446;Nf_ref|GHLB01002391;Nf_ref|GHLB01002404;Np2015e
|GJRU01060932;Nc2017|GJRH01009293;Nc2017|GJRH01009292;Nc2017|GJRH01009291
;Nc2017|GJRH01009298;Nf_ref|GHLB01002402;Nf_ref|GHLB01002398
cluster2640
Np2015e|GJRU01058957;Nf_ref|GHLB01018815;Nf_ref|GHLB01018808;Nf_ref
|GHLB01018814;Nf_ref|GHLB01018817;Cm2018|GJRL01035569;Nc2017|GJRH01009193
;Nc2017|GJRH01009191;Nc2017|GJRH01009187;Nc2017|GJRH01009183
cluster3219
Cm2018|GJRL01009827;Cm2018|GJRL01009826;Cm2018|GJRL01009829;Cm2018|

GJRL01009830;Cm2018|GJRL01009832;Cm2018|GJRL01009833;Nf_ref|GHLB01048052;Np2015e|GJRU01022339;Nc2017|GJRH01029462
cluster3222
Cm2018|GJRL01070964;Cm2018|GJRL01070965;Cm2018|GJRL01070970;Nf_ref|GHLB01043064;Np2015e|GJRU01008667;Nc2017|GJRH01006217;Nc2017|GJRH01006214;Np2015e|GJRU01008668;Np2015e|GJRU01008665
cluster3235
Cm2018|GJRL01015094;Cm2018|GJRL01015092;Cm2018|GJRL01015093;Nf_ref|GHLB01005500;Nf_ref|GHLB01005497;Np2015e|GJRU01054393;Nc2017|GJRH01043663;Np2015e|GJRU01054394;Nf_ref|GHLB01005501
cluster3241
Cm2018|GJRL01031079;Cm2018|GJRL01031099;Cm2018|GJRL01031093;Nf_ref|GHLB01046953;Np2015e|GJRU01055719;Nc2017|GJRH01046503;Nc2017|GJRH01046498;Nc2017|GJRH01046511;Nf_ref|GHLB01046954
cluster3256
Cm2018|GJRL01071810;Cm2018|GJRL01071805;Cm2018|GJRL01071806;Cm2018|GJRL01071807;Cm2018|GJRL01071808;Cm2018|GJRL01071809;Nf_ref|GHLB01002854;Np2015e|GJRU01041507;Nc2017|GJRH01077452
cluster3257
Cm2018|GJRL01012705;Cm2018|GJRL01012697;Cm2018|GJRL01012699;Cm2018|GJRL01012702;Nf_ref|GHLB01023254;Np2015e|GJRU01023205;Nc2017|GJRH01003286;Nc2017|GJRH01003285;Nc2017|GJRH01003284
cluster3282
Cm2018|GJRL01000752;Cm2018|GJRL01000751;Nf_ref|GHLB01017279;Np2015e|GJRU01045641;Nc2017|GJRH01071275;Np2015e|GJRU01045640;Np2015e|GJRU01045642;Np2015e|GJRU01045645;Nc2017|GJRH01054440
cluster3303
Cm2018|GJRL01069341;Nf_ref|GHLB01036301;Np2015e|GJRU01065906;Nc2017|GJRH01061958;Np2015e|GJRU01065904;Np2015e|GJRU01065905;Np2015e|GJRU01065907;Nf_ref|GHLB01036300;Nc2017|GJRH01061956
cluster3308
Cm2018|GJRL01005801;Nf_ref|GHLB01050574;Nf_ref|GHLB01050573;Nf_ref|GHLB01050572;Np2015e|GJRU01026266;Nc2017|GJRH01044606;Nc2017|GJRH01044605;Nc2017|GJRH01044604;Nc2017|GJRH01044603
cluster3309
Cm2018|GJRL01035757;Nf_ref|GHLB01035490;Np2015e|GJRU01057754;Nc2017|GJRH01002082;Nc2017|GJRH01002081;Nc2017|GJRH01002080;Nc2017|GJRH01002079;Nf_ref|GHLB01035489;Nf_ref|GHLB01012359
cluster3319
Nc2017|GJRH01067339;Nc2017|GJRH01067334;Nc2017|GJRH01067340;Nf_ref|GHLB01004998;Nf_ref|GHLB01004996;Np2015e|GJRU01066278;Np2015e|GJRU01066277;Cm2018|GJRL01024376;Cm2018|GJRL01024375
cluster3323
Nc2017|GJRH01082768;Nc2017|GJRH01082766;Nc2017|GJRH01082771;Nf_ref|GHLB01020283;Np2015e|GJRU01042856;Cm2018|GJRL01024965;Nf_ref|GHLB01020293;Cm2018|GJRL01024958;Nf_ref|GHLB01020287
cluster3330
Nc2017|GJRH01055015;Nc2017|GJRH01055016;Nc2017|GJRH01055005;Nc2017|GJRH01055012;Nc2017|GJRH01055013;Nf_ref|GHLB01005002;Np2015e|GJRU01073733;Cm2018|GJRL01086337;Nf_ref|GHLB01005000
cluster3336
Nc2017|GJRH01029761;Nc2017|GJRH01029757;Nc2017|GJRH01029758;Nc2017|GJRH01029759;Nc2017|GJRH01029760;Nf_ref|GHLB01037653;Np2015e|GJRU01022051;Np2015e|GJRU01022049;Cm2018|GJRL01032374

cluster3351
Nc2017|GJRH01056214;Nf_ref|GHLB01033933;Np2015e|GJRU01053331;Np2015e|GJRU01053330;Np2015e|GJRU01053329;Np2015e|GJRU01053328;Cm2018|GJRL01086311;Cm2018|GJRL01086309;Nc2017|GJRH01056218
cluster3366
Np2015e|GJRU01072559;Np2015e|GJRU01072560;Np2015e|GJRU01072561;Nf_ref|GHLB01016127;Nf_ref|GHLB01016126;Cm2018|GJRL01049979;Nc2017|GJRH01017204;Nc2017|GJRH01017203;Nf_ref|GHLB01016128
cluster3764
Np2015e|GJRU01052234;Cm2018|GJRL01009950;Cm2018|GJRL01009948;Cm2018|GJRL01009949;Cm2018|GJRL01009951;Nf_ref|GHLB01030591;Nc2017|GJRH01046949;Nf_ref|GHLB01030590
cluster3914
Np2015e|GJRU01071921;Np2015e|GJRU01071920;Np2015e|GJRU01071914;Np2015e|GJRU01071915;Cm2018|GJRL01027085;Nf_ref|GHLB01039861;Nc2017|GJRH01032976;Nc2017|GJRH01032988
cluster4083
Cm2018|GJRL01038460;Cm2018|GJRL01038455;Cm2018|GJRL01038459;Nf_ref|GHLB01004220;Np2015e|GJRU01027573;Np2015e|GJRU01027572;Nc2017|GJRH01044643;Nc2017|GJRH01044642
cluster4084
Cm2018|GJRL01071353;Cm2018|GJRL01071343;Cm2018|GJRL01071348;Cm2018|GJRL01071360;Nf_ref|GHLB01003074;Np2015e|GJRU01046503;Np2015e|GJRU01046502;Nc2017|GJRH01040406
cluster4087
Cm2018|GJRL01027045;Cm2018|GJRL01027044;Nf_ref|GHLB01042325;Nf_ref|GHLB01042324;Np2015e|GJRU01047404;Cm2018|GJRL01027041;Nc2017|GJRH01025834;Nc2017|GJRH01025831
cluster4097
Cm2018|GJRL01057956;Cm2018|GJRL01057955;Nf_ref|GHLB01044505;Np2015e|GJRU01057721;Nc2017|GJRH01008670;Nf_ref|GHLB01044506;Nf_ref|GHLB01044507;Nf_ref|GHLB01044524
cluster4099
Cm2018|GJRL01012320;Cm2018|GJRL01012333;Nf_ref|GHLB01021116;Nf_ref|GHLB01021115;Np2015e|GJRU01056654;Nc2017|GJRH01088182;Np2015e|GJRU01056656;Np2015e|GJRU01056655
cluster4103
Cm2018|GJRL01080528;Cm2018|GJRL01080523;Cm2018|GJRL01080532;Nf_ref|GHLB01026237;Np2015e|GJRU01030954;Nc2017|GJRH01041624;Nc2017|GJRH01041619;Np2015e|GJRU01030955
cluster4107
Cm2018|GJRL01043525;Cm2018|GJRL01043524;Cm2018|GJRL01043531;Cm2018|GJRL01043532;Nf_ref|GHLB01015445;Np2015e|GJRU01017395;Np2015e|GJRU01017394;Nc2017|GJRH01007560
cluster4114
Cm2018|GJRL01020336;Cm2018|GJRL01020335;Cm2018|GJRL01020338;Cm2018|GJRL01020342;Cm2018|GJRL01020343;Np2015e|GJRU01055275;Nc2017|GJRH01085983;Nf_ref|GHLB01009605
cluster4128
Cm2018|GJRL01037349;Cm2018|GJRL01037350;Cm2018|GJRL01037351;Cm2018|GJRL01037352;Np2015e|GJRU01067052;Nc2017|GJRH01044684;Nf_ref|GHLB01019327;Nf_ref|GHLB01019328
cluster4132
Cm2018|GJRL01037470;Cm2018|GJRL01037471;Nf_ref|GHLB01048795;Np2015e

|GJRU01035211;Nc2017|GJRH01051894;Np2015e|GJRU01035213;Np2015e|GJRU01035212;Nc2017|GJRH01051893
cluster4157
Cm2018|GJRL01013203;Cm2018|GJRL01013200;Cm2018|GJRL01013201;Cm2018|GJRL01013202;Np2015e|GJRU01047681;Np2015e|GJRU01047680;Nc2017|GJRH01069186;Nf_ref|GHLB01042536
cluster4158
Cm2018|GJRL01040521;Cm2018|GJRL01040522;Cm2018|GJRL01040511;Cm2018|GJRL01040516;Cm2018|GJRL01040519;Nf_ref|GHLB01015023;Nc2017|GJRH01084553;Np2015e|GJRU01047339
cluster4165
Cm2018|GJRL01043482;Cm2018|GJRL01043476;Cm2018|GJRL01043477;Cm2018|GJRL01043481;Cm2018|GJRL01043485;Nf_ref|GHLB01016442;Np2015e|GJRU01032424;Nc2017|GJRH01069434
cluster4166
Cm2018|GJRL01058786;Cm2018|GJRL01058784;Nf_ref|GHLB01039628;Np2015e|GJRU01053995;Nc2017|GJRH01062640;Nc2017|GJRH01062639;Nf_ref|GHLB01039631;Nf_ref|GHLB01039634
cluster4167
Cm2018|GJRL01002925;Cm2018|GJRL01002926;Nf_ref|GHLB01015025;Nf_ref|GHLB01015024;Nc2017|GJRH01032562;Nc2017|GJRH01032560;Np2015e|GJRU01053894;Cm2018|GJRL01002927
cluster4183
Cm2018|GJRL01052740;Nf_ref|GHLB01039636;Np2015e|GJRU01052727;Nc2017|GJRH01034590;Nc2017|GJRH01034591;Nf_ref|GHLB01039630;Np2015e|GJRU01052728;Np2015e|GJRU01052726
cluster4194
Cm2018|GJRL01013556;Nf_ref|GHLB01012052;Np2015e|GJRU01066692;Nc2017|GJRH01085486;Cm2018|GJRL01013555;Nf_ref|GHLB01012053;Np2015e|GJRU01066691;Nc2017|GJRH01085485
cluster4200
Cm2018|GJRL01062831;Nf_ref|GHLB01018745;Np2015e|GJRU01062002;Nc2017|GJRH01075401;Np2015e|GJRU01062003;Np2015e|GJRU01062004;Np2015e|GJRU01062006;Nc2017|GJRH01075402
cluster4207
Cm2018|GJRL01017195;Nf_ref|GHLB01008833;Nf_ref|GHLB01008832;Np2015e|GJRU01070197;Nc2017|GJRH01071915;Nc2017|GJRH01071906;Nc2017|GJRH01071903;Nc2017|GJRH01071902
cluster4224
Nc2017|GJRH01033676;Nc2017|GJRH01033671;Nc2017|GJRH01033672;Nc2017|GJRH01033673;Nf_ref|GHLB01016523;Nf_ref|GHLB01016522;Np2015e|GJRU01015971;Cm2018|GJRL01083690
cluster4233
Nc2017|GJRH01009791;Nc2017|GJRH01009793;Nc2017|GJRH01009796;Nc2017|GJRH01009797;Nc2017|GJRH01009798;Nf_ref|GHLB01016354;Cm2018|GJRL01013375;Np2015e|GJRU01063651
cluster4251
Nc2017|GJRH01007163;Nc2017|GJRH01007165;Np2015e|GJRU01037367;Np2015e|GJRU01037368;Nf_ref|GHLB01027391;Nc2017|GJRH01007164;Cm2018|GJRL01059322;Cm2018|GJRL01059328
cluster4256
Nc2017|GJRH01023617;Nf_ref|GHLB01000690;Np2015e|GJRU01056097;Cm2018|GJRL01071337;Cm2018|GJRL01071336;Cm2018|GJRL01071338;Cm2018|GJRL01058152;Np2015e|GJRU01056098

cluster4268
Np2015e|GJRU01051278;Np2015e|GJRU01051275;Np2015e|GJRU01051276;Np2015e|GJRU01051277;Np2015e|GJRU01051279;Nf_ref|GHLB01001634;Cm2018|GJRL01014988;Nc2017|GJRH01052705
cluster4282
Np2015e|GJRU01002690;Nf_ref|GHLB01018706;Nf_ref|GHLB01018703;Cm2018|GJRL01064336;Nc2017|GJRH01046005;Cm2018|GJRL01064334;Nc2017|GJRH01045998;Nc2017|GJRH01046012
cluster4729
Np2015e|GJRU01016344;Cm2018|GJRL01063809;Cm2018|GJRL01063810;Cm2018|GJRL01063814;Cm2018|GJRL01063815;Nf_ref|GHLB01045795;Nc2017|GJRH01083652
cluster4786
Np2015e|GJRU01001407;Nc2017|GJRH01004075;Cm2018|GJRL01032347;Cm2018|GJRL01032348;Cm2018|GJRL01032349;Nf_ref|GHLB01037300;Nf_ref|GHLB01037308
cluster4789
Nf_ref|GHLB01007237;Np2015e|GJRU01016133;Cm2018|GJRL01041126;Nc2017|GJRH01073716;Nc2017|GJRH01073715;Nc2017|GJRH01073714;Nc2017|GJRH01073713
cluster4991
Cm2018|GJRL01047984;Cm2018|GJRL01047982;Np2015e|GJRU01073349;Nf_ref|GHLB01011936;Nc2017|GJRH01069506;Nf_ref|GHLB01011937;Np2015e|GJRU01073348
cluster5163
Nf_ref|GHLB01032710;Np2015e|GJRU01059289;Nc2017|GJRH01056822;Cm2018|GJRL01079312;Cm2018|GJRL01079313;Cm2018|GJRL01079316;Cm2018|GJRL01079317
cluster5187
Cm2018|GJRL01030072;Cm2018|GJRL01030075;Cm2018|GJRL01030076;Nf_ref|GHLB01025108;Np2015e|GJRU01061334;Nc2017|GJRH01049764;Np2015e|GJRU01061335
cluster5197
Cm2018|GJRL01074650;Cm2018|GJRL01074639;Cm2018|GJRL01074640;Cm2018|GJRL01074644;Nf_ref|GHLB01030589;Np2015e|GJRU01020944;Nc2017|GJRH01003780
cluster5202
Cm2018|GJRL01075269;Cm2018|GJRL01075268;Cm2018|GJRL01075270;Nf_ref|GHLB01007911;Np2015e|GJRU01031608;Np2015e|GJRU01031604;Nc2017|GJRH01075656
cluster5207
Cm2018|GJRL01028304;Cm2018|GJRL01028305;Cm2018|GJRL01028306;Np2015e|GJRU01044593;Nc2017|GJRH01076201;Nf_ref|GHLB01031011;Nc2017|GJRH01076206
cluster5217
Cm2018|GJRL01053730;Cm2018|GJRL01053731;Cm2018|GJRL01053732;Nf_ref|GHLB01006645;Np2015e|GJRU01065065;Nc2017|GJRH01040596;Nc2017|GJRH01040586
cluster5222
Cm2018|GJRL01047614;Cm2018|GJRL01047617;Nf_ref|GHLB01025158;Np2015e|GJRU01006562;Nc2017|GJRH01006957;Nc2017|GJRH01006956;Nf_ref|GHLB01025157
cluster5225
Cm2018|GJRL01058671;Cm2018|GJRL01058670;Cm2018|GJRL01058672;Cm2018|GJRL01058673;Nf_ref|GHLB01041557;Np2015e|GJRU01024146;Nc2017|GJRH01025781
cluster5226
Cm2018|GJRL01036463;Cm2018|GJRL01036460;Cm2018|GJRL01036461;Cm2018|GJRL01036462;Nf_ref|GHLB01021069;Np2015e|GJRU01036795;Nc2017|GJRH01063823
cluster5244
Cm2018|GJRL01079160;Cm2018|GJRL01079165;Cm2018|GJRL01079169;Nc2017|GJRH01018979;Np2015e|GJRU01018305;Nc2017|GJRH01018975;Nf_ref|GHLB01014312

cluster5245
Cm2018|GJRL01020783;Cm2018|GJRL01020780;Cm2018|GJRL01020781;Cm2018|GJRL01020782;Nf_ref|GHLB01049944;Np2015e|GJRU01026963;Nc2017|GJRH01042669
cluster5251
Cm2018|GJRL01082052;Cm2018|GJRL01082054;Cm2018|GJRL01082056;Cm2018|GJRL01082061;Nf_ref|GHLB01016743;Np2015e|GJRU01018644;Nc2017|GJRH01015994
cluster5260
Cm2018|GJRL01084137;Cm2018|GJRL01084138;Nf_ref|GHLB01036740;Np2015e|GJRU01029101;Nc2017|GJRH01086108;Np2015e|GJRU01029100;Nf_ref|GHLB01036739
cluster5267
Cm2018|GJRL01087000;Cm2018|GJRL01087001;Nf_ref|GHLB01003498;Np2015e|GJRU01060988;Nc2017|GJRH01083205;Np2015e|GJRU01060984;Nf_ref|GHLB01003497
cluster5276
Cm2018|GJRL01062191;Cm2018|GJRL01062189;Cm2018|GJRL01062194;Nf_ref|GHLB01047695;Np2015e|GJRU01059964;Np2015e|GJRU01059963;Nc2017|GJRH01005287
cluster5285
Cm2018|GJRL01085121;Cm2018|GJRL01085123;Cm2018|GJRL01085126;Nf_ref|GHLB01036101;Np2015e|GJRU01058209;Nc2017|GJRH01076342;Nf_ref|GHLB01036100
cluster5286
Cm2018|GJRL01013040;Cm2018|GJRL01013043;Nf_ref|GHLB01027384;Nc2017|GJRH01003698;Np2015e|GJRU01060983;Np2015e|GJRU01060982;Nf_ref|GHLB01051459
cluster5289
Cm2018|GJRL01061140;Cm2018|GJRL01061142;Nf_ref|GHLB01002502;Np2015e|GJRU01016669;Nc2017|GJRH01057898;Nc2017|GJRH01057897;Nf_ref|GHLB01002501
cluster5294
Cm2018|GJRL01072128;Cm2018|GJRL01072129;Nf_ref|GHLB01021289;Np2015e|GJRU01041201;Np2015e|GJRU01041200;Nc2017|GJRH01051470;Nc2017|GJRH01051469
cluster5303
Cm2018|GJRL01073204;Cm2018|GJRL01073220;Nf_ref|GHLB01046083;Nf_ref|GHLB01046081;Np2015e|GJRU01009544;Nc2017|GJRH01054602;Nc2017|GJRH01054595
cluster5319
Cm2018|GJRL01007740;Nf_ref|GHLB01001805;Np2015e|GJRU01011495;Np2015e|GJRU01011491;Nc2017|GJRH01075660;Np2015e|GJRU01011494;Np2015e|GJRU01011497
cluster5339
Cm2018|GJRL01052104;Nf_ref|GHLB01000481;Nc2017|GJRH01086901;Nc2017|GJRH01086898;Nc2017|GJRH01086888;Nc2017|GJRH01086882;Np2015e|GJRU01066314
cluster5343
Cm2018|GJRL01027535;Nf_ref|GHLB01014111;Np2015e|GJRU01058138;Nc2017|GJRH01083854;Nc2017|GJRH01083852;Np2015e|GJRU01058134;Np2015e|GJRU01058136
cluster5355
Cm2018|GJRL01075000;Nf_ref|GHLB01020125;Nf_ref|GHLB01020120;Nc2017|GJRH01047751;Np2015e|GJRU01023899;Np2015e|GJRU01023898;Np2015e|GJRU01023895
cluster5372
Cm2018|GJRL01026878;Nf_ref|GHLB01019940;Nf_ref|GHLB01019937;Np2015e|GJRU01020690;Np2015e|GJRU01020689;Nc2017|GJRH01083488;Nc2017|GJRH01083490

cluster5388
Nc2017|GJRH01045018;Nc2017|GJRH01045017;Nc2017|GJRH01045019;Nc2017|GJRH01045020;Nf_ref|GHLB01013128;Np2015e|GJRU01004506;Cm2018|GJRL01070730
cluster5408
Nc2017|GJRH01052509;Nc2017|GJRH01052504;Nc2017|GJRH01052511;Nc2017|GJRH01052514;Nf_ref|GHLB01008754;Np2015e|GJRU01070108;Cm2018|GJRL01084967
cluster5414
Nc2017|GJRH01039361;Nc2017|GJRH01039357;Nf_ref|GHLB01028689;Nf_ref|GHLB01028688;Np2015e|GJRU01036821;Np2015e|GJRU01036820;Cm2018|GJRL01002612
cluster5420
Nc2017|GJRH01025676;Nc2017|GJRH01025678;Nf_ref|GHLB01022372;Np2015e|GJRU01019122;Np2015e|GJRU01019121;Cm2018|GJRL01087146;Cm2018|GJRL01087137
cluster5464
Np2015e|GJRU01072632;Np2015e|GJRU01072633;Np2015e|GJRU01072634;Nf_ref|GHLB01006219;Nc2017|GJRH01064161;Nc2017|GJRH01064157;Cm2018|GJRL01086214
cluster6041
Cm2018|GJRL01024407;Cm2018|GJRL01032325;Nf_ref|GHLB01038515;Nc2017|GJRH01049058;Np2015e|GJRU01031280;Nc2017|GJRH01007761
cluster6325
Np2015e|GJRU01057137;Nc2017|GJRH01083557;Cm2018|GJRL01000962;Cm2018|GJRL01000963;Nf_ref|GHLB01035484;Nf_ref|GHLB01035482
cluster6348
Nf_ref|GHLB01005345;Nc2017|GJRH01000127;Np2015e|GJRU01028149;Cm2018|GJRL01051949;Cm2018|GJRL01051936;Cm2018|GJRL01051942
cluster6487
Np2015e|GJRU01033068;Cm2018|GJRL01012464;Np2015e|GJRU01033069;Nf_ref|GHLB01014470;Nc2017|GJRH01038336;Nf_ref|GHLB01014466
cluster6621
Cm2018|GJRL01074573;Cm2018|GJRL01074577;Np2015e|GJRU01040631;Np2015e|GJRU01040630;Nf_ref|GHLB01000237;Nc2017|GJRH01030046
cluster6641
Cm2018|GJRL01025073;Cm2018|GJRL01025072;Nf_ref|GHLB01014482;Np2015e|GJRU01022173;Nc2017|GJRH01072247;Nc2017|GJRH01072248
cluster6651
Cm2018|GJRL01030106;Cm2018|GJRL01030116;Nf_ref|GHLB01045980;Np2015e|GJRU01016135;Nc2017|GJRH01090839;Np2015e|GJRU01016136
cluster6659
Cm2018|GJRL01000139;Cm2018|GJRL01000137;Nf_ref|GHLB01003709;Np2015e|GJRU01042495;Nc2017|GJRH01054487;Np2015e|GJRU01042494
cluster6661
Cm2018|GJRL01076496;Cm2018|GJRL01076497;Nf_ref|GHLB01043425;Nf_ref|GHLB01043424;Np2015e|GJRU01029218;Nc2017|GJRH01071931
cluster6668
Cm2018|GJRL01020355;Cm2018|GJRL01020354;Nf_ref|GHLB01034045;Np2015e|GJRU01018429;Nc2017|GJRH01087726;Np2015e|GJRU01018428
cluster6669
Cm2018|GJRL01027565;Cm2018|GJRL01027564;Np2015e|GJRU01030958;Nf_ref|GHLB01028725;Nc2017|GJRH01014276;Nc2017|GJRH01014277
cluster6687
Cm2018|GJRL01079217;Cm2018|GJRL01079215;Cm2018|GJRL01079216;Nf_ref|GHLB01000928;Nc2017|GJRH01034781;Np2015e|GJRU01028271

cluster6701
Cm2018|GJRL01032402;Cm2018|GJRL01032401;Nf_ref|GHLB01042364;Np2015e|GJRU01017962;Nc2017|GJRH01045775;Nc2017|GJRH01045772
cluster6704
Cm2018|GJRL01018701;Cm2018|GJRL01018684;Cm2018|GJRL01018698;Nf_ref|GHLB01006605;Np2015e|GJRU01031933;Nc2017|GJRH01048275
cluster6715
Cm2018|GJRL01040295;Cm2018|GJRL01040294;Nf_ref|GHLB01003619;Np2015e|GJRU01022783;Nc2017|GJRH01006353;Np2015e|GJRU01022784
cluster6732
Cm2018|GJRL01074598;Cm2018|GJRL01074595;Nf_ref|GHLB01028716;Np2015e|GJRU01033074;Nc2017|GJRH01007827;Np2015e|GJRU01033073
cluster6734
Cm2018|GJRL01068395;Cm2018|GJRL01068387;Cm2018|GJRL01068390;Nf_ref|GHLB01044232;Np2015e|GJRU01033161;Nc2017|GJRH01056782
cluster6735
Cm2018|GJRL01015547;Cm2018|GJRL01015539;Cm2018|GJRL01015541;Nf_ref|GHLB01026370;Np2015e|GJRU01043086;Nc2017|GJRH01068185
cluster6749
Cm2018|GJRL01017314;Cm2018|GJRL01017315;Nf_ref|GHLB01032645;Np2015e|GJRU01065416;Nc2017|GJRH01073647;Np2015e|GJRU01065417
cluster6750
Cm2018|GJRL01020517;Cm2018|GJRL01020513;Cm2018|GJRL01020518;Nf_ref|GHLB01003646;Np2015e|GJRU01008096;Nc2017|GJRH01051573
cluster6768
Cm2018|GJRL01083297;Cm2018|GJRL01083294;Nf_ref|GHLB01023638;Np2015e|GJRU01005994;Nc2017|GJRH01068852;Nc2017|GJRH01068845
cluster6771
Cm2018|GJRL01079811;Cm2018|GJRL01079813;Nf_ref|GHLB01022949;Np2015e|GJRU01064821;Nc2017|GJRH01023592;Nf_ref|GHLB01022948
cluster6773
Cm2018|GJRL01069982;Cm2018|GJRL01069979;Nf_ref|GHLB01016077;Np2015e|GJRU01007412;Nc2017|GJRH01077494;Np2015e|GJRU01007413
cluster6782
Cm2018|GJRL01024465;Cm2018|GJRL01024466;Nf_ref|GHLB01042348;Np2015e|GJRU01067351;Np2015e|GJRU01067350;Nc2017|GJRH01010046
cluster6789
Cm2018|GJRL01036015;Nf_ref|GHLB01012840;Nf_ref|GHLB01012839;Np2015e|GJRU01037324;Nc2017|GJRH01079296;Nc2017|GJRH01079290
cluster6792
Cm2018|GJRL01051664;Nf_ref|GHLB01050649;Np2015e|GJRU01011345;Nc2017|GJRH01086417;Nc2017|GJRH01086415;Nc2017|GJRH01086418
cluster6803
Cm2018|GJRL01015828;Nf_ref|GHLB01000409;Np2015e|GJRU01071171;Np2015e|GJRU01071170;Np2015e|GJRU01071169;Nc2017|GJRH01006322
cluster6805
Cm2018|GJRL01001829;Nf_ref|GHLB01006477;Np2015e|GJRU01012702;Np2015e|GJRU01012701;Nc2017|GJRH01003817;Nc2017|GJRH01003816
cluster6814
Cm2018|GJRL01009804;Nf_ref|GHLB01028697;Nf_ref|GHLB01028696;Np2015e|GJRU01029463;Np2015e|GJRU01029462;Nc2017|GJRH01036448
cluster6815
Cm2018|GJRL01081453;Nf_ref|GHLB01028787;Np2015e|GJRU01019210;Np2015e|GJRU01019209;Nc2017|GJRH01087418;Nc2017|GJRH01087417

cluster6828
Cm2018|GJRL01053728;Nf_ref|GHLB01044806;Np2015e|GJRU01029231;Nc2017|GJRH01018895;Np2015e|GJRU01029230;Nc2017|GJRH01018908
cluster6832
Cm2018|GJRL01049981;Nf_ref|GHLB01007144;Np2015e|GJRU01015678;Nc2017|GJRH01035971;Np2015e|GJRU01025541;Np2015e|GJRU01025539
cluster6846
Cm2018|GJRL01020594;Nf_ref|GHLB01014603;Np2015e|GJRU01021782;Nc2017|GJRH01089432;Nc2017|GJRH01089428;Nf_ref|GHLB01014601
cluster6849
Cm2018|GJRL01075250;Nf_ref|GHLB01025601;Np2015e|GJRU01040936;Nc2017|GJRH01052862;Nc2017|GJRH01052860;Nc2017|GJRH01052859
cluster6851
Cm2018|GJRL01075730;Nf_ref|GHLB01019355;Np2015e|GJRU01060411;Nc2017|GJRH01043375;Np2015e|GJRU01060409;Np2015e|GJRU01060410
cluster6869
Cm2018|GJRL01008728;Nf_ref|GHLB01014198;Nf_ref|GHLB01014197;Np2015e|GJRU01061017;Nc2017|GJRH01048266;Np2015e|GJRU01061021
cluster6870
Cm2018|GJRL01055051;Nf_ref|GHLB01001679;Np2015e|GJRU01056607;Nc2017|GJRH01051071;Nc2017|GJRH01051070;Np2015e|GJRU01056606
cluster6892
Cm2018|GJRL01079872;Nf_ref|GHLB01014174;Np2015e|GJRU01028058;Nc2017|GJRH01011078;Nc2017|GJRH01011081;Nf_ref|GHLB01014171
cluster6895
Cm2018|GJRL01079035;Nf_ref|GHLB01035908;Np2015e|GJRU01011960;Nc2017|GJRH01002348;Np2015e|GJRU01011959;Nf_ref|GHLB01017143
cluster6955
Nc2017|GJRH01078948;Nc2017|GJRH01078951;Nf_ref|GHLB01036131;Np2015e|GJRU01012378;Np2015e|GJRU01012377;Cm2018|GJRL01066411
cluster6972
Nc2017|GJRH01061451;Nc2017|GJRH01061454;Nc2017|GJRH01061456;Nf_ref|GHLB01036383;Np2015e|GJRU01024195;Cm2018|GJRL01044368
cluster7017
Nc2017|GJRH01059334;Cm2018|GJRL01075594;Np2015e|GJRU01055977;Nf_ref|GHLB01042880;Nc2017|GJRH01059336;Nf_ref|GHLB01042879
cluster7034
Np2015e|GJRU01041409;Np2015e|GJRU01041407;Cm2018|GJRL01027915;Nc2017|GJRH01069936;Nf_ref|GHLB01001131;Nc2017|GJRH01069935
cluster7064
Np2015e|GJRU01027879;Np2015e|GJRU01027878;Cm2018|GJRL01065640;Nf_ref|GHLB01017133;Nc2017|GJRH01008187;Nf_ref|GHLB01017132
cluster7070
Np2015e|GJRU01058755;Np2015e|GJRU01058749;Nf_ref|GHLB01025346;Cm2018|GJRL01074751;Cm2018|GJRL01074749;Nc2017|GJRH01015756
cluster7071
Np2015e|GJRU01065427;Np2015e|GJRU01065428;Nf_ref|GHLB01019165;Cm2018|GJRL01070890;Nc2017|GJRH01050862;Nc2017|GJRH01050861
cluster7083
Np2015e|GJRU01055420;Nf_ref|GHLB01030454;Cm2018|GJRL01036983;Np2015e|GJRU01055418;Nc2017|GJRH01042121;Nc2017|GJRH01042112
cluster7566
Np2015e|GJRU01047265;Nc2017|GJRH01015911;Nc2017|GJRH01015906;Nf_ref|GHLB01039033;Cm2018|GJRL01016098

cluster7594
Np2015e|GJRU01020935;Cm2018|GJRL01045703;Nf_ref|GHLB01005406;Nc2017|GJRH01081641;Nc2017|GJRH01081642
cluster7633
Nf_ref|GHLB01021733;Nc2017|GJRH01007090;Nc2017|GJRH01007089;Np2015e|GJRU01022569;Cm2018|GJRL01048433
cluster7876
Cm2018|GJRL01010679;Nf_ref|GHLB01048650;Np2015e|GJRU01064877;Nc2017|GJRH01082713;Nc2017|GJRH01082711
cluster8081
Cm2018|GJRL01060648;Nf_ref|GHLB01000347;Np2015e|GJRU01056135;Nc2017|GJRH01068551;Nc2017|GJRH01068550
cluster8089
Nf_ref|GHLB01022583;Cm2018|GJRL01051961;Np2015e|GJRU01035395;Nc2017|GJRH01054455;Nc2017|GJRH01054456
cluster8198
Np2015e|GJRU01035969;Cm2018|GJRL01057464;Nf_ref|GHLB01042975;Nc2017|GJRH01078964;Nc2017|GJRH01078963
cluster8205
Cm2018|GJRL01022171;Cm2018|GJRL01022169;Nf_ref|GHLB01009036;Np2015e|GJRU01004919;Nc2017|GJRH01084281
cluster8210
Cm2018|GJRL01074427;Cm2018|GJRL01074432;Nf_ref|GHLB01005042;Np2015e|GJRU01005089;Nc2017|GJRH01023159
cluster8248
Cm2018|GJRL01055035;Cm2018|GJRL01055036;Nf_ref|GHLB01009571;Np2015e|GJRU01065299;Nc2017|GJRH01011971
cluster8256
Cm2018|GJRL01047344;Cm2018|GJRL01047343;Nf_ref|GHLB01009657;Np2015e|GJRU01062434;Nc2017|GJRH01025780
cluster8258
Cm2018|GJRL01077595;Cm2018|GJRL01077594;Nf_ref|GHLB01005661;Np2015e|GJRU01042470;Nc2017|GJRH01048579
cluster8269
Cm2018|GJRL01075158;Cm2018|GJRL01075159;Nf_ref|GHLB01001141;Np2015e|GJRU01051332;Nc2017|GJRH01015815
cluster8377
Cm2018|GJRL01017415;Cm2018|GJRL01017414;Nf_ref|GHLB01006315;Nc2017|GJRH01075734;Np2015e|GJRU01057695
cluster8389
Cm2018|GJRL01035702;Cm2018|GJRL01035701;Nf_ref|GHLB01011394;Np2015e|GJRU01043110;Nc2017|GJRH01077515
cluster8401
Cm2018|GJRL01078431;Cm2018|GJRL01078435;Np2015e|GJRU01037883;Nc2017|GJRH01062152;Nf_ref|GHLB01036340
cluster8403
Cm2018|GJRL01087342;Cm2018|GJRL01087344;Nf_ref|GHLB01032111;Np2015e|GJRU01019690;Nc2017|GJRH01016450
cluster8444
Cm2018|GJRL01045889;Nf_ref|GHLB01012887;Np2015e|GJRU01040851;Nc2017|GJRH01087071;Nc2017|GJRH01087070
cluster8450
Cm2018|GJRL01055245;Nf_ref|GHLB01044325;Np2015e|GJRU01019345;Nc2017|GJRH01039631;Nc2017|GJRH01039630

cluster8452
Cm2018|GJRL01041657;Nf_ref|GHLB01026112;Np2015e|GJRU01009681;Np2015e|GJRU01009680;Nc2017|GJRH01058796
cluster8456
Cm2018|GJRL01014349;Nf_ref|GHLB01046894;Np2015e|GJRU01005811;Np2015e|GJRU01005810;Nc2017|GJRH01057986
cluster8468
Cm2018|GJRL01061670;Nf_ref|GHLB01044430;Np2015e|GJRU01028724;Nc2017|GJRH01077273;Nf_ref|GHLB01044429
cluster8487
Cm2018|GJRL01043628;Nf_ref|GHLB01024382;Np2015e|GJRU01044094;Np2015e|GJRU01044093;Nc2017|GJRH01052668
cluster8489
Cm2018|GJRL01087480;Nf_ref|GHLB01013037;Np2015e|GJRU01061124;Nc2017|GJRH01077455;Nc2017|GJRH01077454
cluster8490
Cm2018|GJRL01009382;Nc2017|GJRH01005617;Nc2017|GJRH01005614;Nf_ref|GHLB01030244;Np2015e|GJRU01047027
cluster8506
Cm2018|GJRL01066842;Nf_ref|GHLB01032724;Np2015e|GJRU01054369;Nc2017|GJRH01050762;Nc2017|GJRH01050761
cluster8510
Cm2018|GJRL01005889;Nf_ref|GHLB01046038;Np2015e|GJRU01063025;Nc2017|GJRH01035532;Nc2017|GJRH01035530
cluster8519
Cm2018|GJRL01017934;Nf_ref|GHLB01000371;Np2015e|GJRU01027605;Nc2017|GJRH01027551;Nc2017|GJRH01027548
cluster8524
Cm2018|GJRL01014662;Nf_ref|GHLB01026059;Np2015e|GJRU01055511;Nc2017|GJRH01059174;Nc2017|GJRH01059164
cluster8530
Cm2018|GJRL01001546;Np2015e|GJRU01036288;Np2015e|GJRU01036287;Nc2017|GJRH01030247;Nf_ref|GHLB01030212
cluster8534
Cm2018|GJRL01045494;Nf_ref|GHLB01036204;Np2015e|GJRU01056597;Nc2017|GJRH01037691;Nc2017|GJRH01037692
cluster8535
Cm2018|GJRL01032572;Nf_ref|GHLB01033633;Np2015e|GJRU01069907;Nc2017|GJRH01072522;Np2015e|GJRU01069908
cluster8537
Cm2018|GJRL01081336;Nf_ref|GHLB01012837;Np2015e|GJRU01011906;Nc2017|GJRH01020919;Nc2017|GJRH01020918
cluster8547
Cm2018|GJRL01037704;Nf_ref|GHLB01015478;Np2015e|GJRU01008581;Nc2017|GJRH01084584;Np2015e|GJRU01008580
cluster8560
Cm2018|GJRL01032275;Nf_ref|GHLB01023156;Np2015e|GJRU01044073;Nc2017|GJRH01071218;Np2015e|GJRU01044072
cluster8561
Cm2018|GJRL01063640;Nf_ref|GHLB01030713;Np2015e|GJRU01063398;Nc2017|GJRH01079821;Nc2017|GJRH01079819
cluster8562
Cm2018|GJRL01065507;Nf_ref|GHLB01049744;Nf_ref|GHLB01049739;Np2015e|GJRU01018362;Nc2017|GJRH01086320

cluster8569
Cm2018|GJRL01074005;Nf_ref|GHLB01046017;Nf_ref|GHLB01046016;Np2015e|GJRU01043334;Nc2017|GJRH01066458
cluster8574
Cm2018|GJRL01002131;Nf_ref|GHLB01009795;Np2015e|GJRU01033526;Nc2017|GJRH01023164;Np2015e|GJRU01033527
cluster8604
Nc2017|GJRH01060208;Nc2017|GJRH01060207;Nf_ref|GHLB01009653;Np2015e|GJRU01021591;Cm2018|GJRL01025317
cluster8688
Nc2017|GJRH01010474;Nc2017|GJRH01010479;Nf_ref|GHLB01019309;Np2015e|GJRU01032658;Cm2018|GJRL01008681
cluster8699
Nc2017|GJRH01071423;Nc2017|GJRH01071422;Nf_ref|GHLB01030192;Np2015e|GJRU01065142;Cm2018|GJRL01075558
cluster8729
Nc2017|GJRH01002571;Nf_ref|GHLB01011516;Np2015e|GJRU01006148;Np2015e|GJRU01006147;Cm2018|GJRL01031914
cluster8735
Nc2017|GJRH01025735;Nf_ref|GHLB01001136;Np2015e|GJRU01019152;Cm2018|GJRL01063142;Nf_ref|GHLB01001135
cluster8739
Nc2017|GJRH01085807;Nf_ref|GHLB01032213;Np2015e|GJRU01069955;Cm2018|GJRL01056147;Cm2018|GJRL01056143
cluster8747
Nc2017|GJRH01034690;Nf_ref|GHLB01026546;Np2015e|GJRU01028239;Cm2018|GJRL01044710;Np2015e|GJRU01028238
cluster8748
Cm2018|GJRL01043571;Nc2017|GJRH01006192;Nf_ref|GHLB01015290;Nf_ref|GHLB01015289;Np2015e|GJRU01051269
cluster8751
Nc2017|GJRH01087572;Nf_ref|GHLB01009253;Np2015e|GJRU01016251;Cm2018|GJRL01028527;Np2015e|GJRU01039123
cluster8792
Np2015e|GJRU01019270;Np2015e|GJRU01019271;Nf_ref|GHLB01022002;Cm2018|GJRL01055984;Nc2017|GJRH01064314
cluster8820
Np2015e|GJRU01037295;Np2015e|GJRU01037292;Nf_ref|GHLB01026795;Cm2018|GJRL01085607;Nc2017|GJRH01087208
cluster8863
Np2015e|GJRU01041576;Np2015e|GJRU01041577;Nf_ref|GHLB01031136;Cm2018|GJRL01034183;Nc2017|GJRH01037780
cluster8872
Np2015e|GJRU01055649;Nf_ref|GHLB01023573;Cm2018|GJRL01014800;Nc2017|GJRH01059298;Nc2017|GJRH01059296
cluster8879
Np2015e|GJRU01017544;Nf_ref|GHLB01025853;Cm2018|GJRL01077927;Nc2017|GJRH01002435;Cm2018|GJRL01077928
cluster8882
Np2015e|GJRU01049567;Nf_ref|GHLB01047490;Np2015e|GJRU01049569;Cm2018|GJRL01053876;Nc2017|GJRH01005289
cluster8885
Np2015e|GJRU01059954;Nf_ref|GHLB01001890;Cm2018|GJRL01031003;Nc2017|GJRH01034212;Nc2017|GJRH01034207

cluster8886
Np2015e|GJRU01065764;Nf_ref|GHLB01000861;Cm2018|GJRL01024057;Nc2017|GJRH01035647;Nc2017|GJRH01035646
cluster8893
Np2015e|GJRU01004153;Nf_ref|GHLB01004185;Cm2018|GJRL01078913;Nc2017|GJRH01059614;Cm2018|GJRL01078914
cluster8899
Np2015e|GJRU01052858;Nf_ref|GHLB01001824;Cm2018|GJRL01085992;Nc2017|GJRH01058740;Nc2017|GJRH01058739
cluster8900
Np2015e|GJRU01007499;Nf_ref|GHLB01004592;Cm2018|GJRL01068783;Nc2017|GJRH01071868;Nf_ref|GHLB01004591
cluster8914
Np2015e|GJRU01067522;Nf_ref|GHLB01037072;Cm2018|GJRL01084648;Nc2017|GJRH01080712;Nc2017|GJRH01080710
cluster8915
Np2015e|GJRU01037181;Cm2018|GJRL01056495;Nc2017|GJRH01076896;Nf_ref|GHLB01040551;Nf_ref|GHLB01040554
cluster9488
Cm2018|GJRL01039813;Nc2017|GJRH01047237;Nf_ref|GHLB01023281;Np2015e|GJRU01025070
cluster9504
Cm2018|GJRL01057991;Np2015e|GJRU01056081;Nf_ref|GHLB01050578;Nc2017|GJRH01081315
cluster9527
Nf_ref|GHLB01051281;Cm2018|GJRL01011192;Np2015e|GJRU01016989;Nc2017|GJRH01066115
cluster9559
Cm2018|GJRL01061340;Nf_ref|GHLB01025047;Np2015e|GJRU01012579;Nc2017|GJRH01050946
cluster9587
Cm2018|GJRL01015551;Nc2017|GJRH01082290;Nf_ref|GHLB01021145;Np2015e|GJRU01001486
cluster9638
Np2015e|GJRU01037178;Cm2018|GJRL01085886;Nf_ref|GHLB01023649;Nc2017|GJRH01051416
cluster9650
Cm2018|GJRL01013907;Np2015e|GJRU01057578;Nf_ref|GHLB01049706;Nc2017|GJRH01000529
cluster10125
Nf_ref|GHLB01008952;Cm2018|GJRL01050177;Np2015e|GJRU01037327;Nc2017|GJRH01028274
cluster10166
Np2015e|GJRU01070703;Cm2018|GJRL01056640;Nf_ref|GHLB01033722;Nc2017|GJRH01073084
cluster10477
Cm2018|GJRL01024418;Nf_ref|GHLB01022705;Np2015e|GJRU01009521;Nc2017|GJRH01040434
cluster10481
Cm2018|GJRL01069793;Nf_ref|GHLB01045050;Np2015e|GJRU01046789;Nc2017|GJRH01064471
cluster10491
Cm2018|GJRL01062407;Nf_ref|GHLB01046725;Np2015e|GJRU01041459;Nc2017|GJRH01005632

cluster10493
Cm2018|GJRL01080306;Nf_ref|GHLB01014345;Np2015e|GJRU01014074;Nc2017|GJRH01075524
cluster10495
Cm2018|GJRL01022882;Nf_ref|GHLB01007763;Np2015e|GJRU01007889;Nc2017|GJRH01087679
cluster10506
Cm2018|GJRL01065256;Nf_ref|GHLB01001639;Np2015e|GJRU01027400;Nc2017|GJRH01058231
cluster10516
Cm2018|GJRL01035650;Nf_ref|GHLB01003634;Np2015e|GJRU01064695;Nc2017|GJRH01081177
cluster10524
Cm2018|GJRL01026755;Nf_ref|GHLB01046678;Np2015e|GJRU01005004;Nc2017|GJRH01049479
cluster10527
Cm2018|GJRL01050960;Nf_ref|GHLB01031002;Np2015e|GJRU01025401;Nc2017|GJRH01042717
cluster10528
Cm2018|GJRL01042814;Nf_ref|GHLB01016652;Np2015e|GJRU01007493;Nc2017|GJRH01008281
cluster10543
Cm2018|GJRL01057444;Nf_ref|GHLB01027279;Np2015e|GJRU01063663;Nc2017|GJRH01016437
cluster10545
Cm2018|GJRL01014280;Nf_ref|GHLB01005545;Np2015e|GJRU01056951;Nc2017|GJRH01056761
cluster10550
Cm2018|GJRL01016132;Nf_ref|GHLB01032691;Np2015e|GJRU01042192;Nc2017|GJRH01086453
cluster10551
Cm2018|GJRL01045205;Nf_ref|GHLB01012959;Np2015e|GJRU01041076;Nc2017|GJRH01003774
cluster10555
Cm2018|GJRL01054584;Nf_ref|GHLB01036688;Np2015e|GJRU01009702;Nc2017|GJRH01077809
cluster10560
Cm2018|GJRL01022805;Nf_ref|GHLB01044195;Np2015e|GJRU01043096;Nc2017|GJRH01024625
cluster10563
Cm2018|GJRL01026926;Nf_ref|GHLB01023244;Np2015e|GJRU01015367;Nc2017|GJRH01071937
cluster10575
Cm2018|GJRL01056931;Nf_ref|GHLB01021067;Np2015e|GJRU01029259;Nc2017|GJRH01065220
cluster10593
Cm2018|GJRL01001498;Nf_ref|GHLB01020715;Np2015e|GJRU01028588;Nc2017|GJRH01063800
cluster10619
Cm2018|GJRL01045847;Nf_ref|GHLB01006732;Np2015e|GJRU01056491;Nc2017|GJRH01088049
cluster10634
Cm2018|GJRL01003561;Nf_ref|GHLB01015215;Np2015e|GJRU01056068;Nc2017|GJRH01043490

cluster10635
Cm2018|GJRL01020706;Nf_ref|GHLB01009565;Np2015e|GJRU01031843;Nc2017
|GJRH01089520
cluster10639
Cm2018|GJRL01035770;Nf_ref|GHLB01007777;Np2015e|GJRU01040966;Nc2017
|GJRH01060959
cluster10640
Cm2018|GJRL01013472;Nf_ref|GHLB01008975;Np2015e|GJRU01029534;Nc2017
|GJRH01050899
cluster10641
Cm2018|GJRL01043326;Nf_ref|GHLB01025357;Np2015e|GJRU01054227;Nc2017
|GJRH01050780
cluster10658
Cm2018|GJRL01049111;Nf_ref|GHLB01025318;Np2015e|GJRU01023423;Nc2017
|GJRH01004538
cluster10664
Cm2018|GJRL01037136;Nf_ref|GHLB01000886;Np2015e|GJRU01054819;Nc2017
|GJRH01040333
cluster10668
Cm2018|GJRL01052188;Nf_ref|GHLB01010524;Np2015e|GJRU01006154;Nc2017
|GJRH01052614
cluster10678
Cm2018|GJRL01012741;Nf_ref|GHLB01041605;Np2015e|GJRU01018829;Nc2017
|GJRH01025972
cluster10684
Cm2018|GJRL01059748;Nf_ref|GHLB01001550;Np2015e|GJRU01072882;Nc2017
|GJRH01000599
cluster10691
Cm2018|GJRL01022242;Nf_ref|GHLB01024853;Np2015e|GJRU01016769;Nc2017
|GJRH01073898
cluster10694
Cm2018|GJRL01005776;Nf_ref|GHLB01026705;Np2015e|GJRU01023626;Nc2017
|GJRH01057932
cluster10695
Cm2018|GJRL01017735;Nf_ref|GHLB01035854;Np2015e|GJRU01062489;Nc2017
|GJRH01091024
cluster10697
Cm2018|GJRL01072401;Nf_ref|GHLB01026623;Np2015e|GJRU01035843;Nc2017
|GJRH01006730
cluster10710
Cm2018|GJRL01017280;Nf_ref|GHLB01009123;Np2015e|GJRU01019179;Nc2017
|GJRH01086983
cluster10713
Cm2018|GJRL01018974;Nf_ref|GHLB01016430;Np2015e|GJRU01032258;Nc2017
|GJRH01055540
cluster10914
Nc2017|GJRH01008344;Nf_ref|GHLB01035085;Np2015e|GJRU01004342;Cm2018
|GJRL01065008
cluster10924
Nc2017|GJRH01073822;Nf_ref|GHLB01030178;Np2015e|GJRU01041085;Cm2018
|GJRL01076981
cluster10930
Nc2017|GJRH01000316;Nf_ref|GHLB01018124;Np2015e|GJRU01057413;Cm2018
|GJRL01027311

cluster10935
Nc2017|GJRH01035354;Nf_ref|GHLB01006190;Np2015e|GJRU01025196;Cm2018|GJRL01044842
cluster10938
Nc2017|GJRH01003772;Nf_ref|GHLB01026209;Np2015e|GJRU01032648;Cm2018|GJRL01039497
cluster10945
Nc2017|GJRH01003397;Nf_ref|GHLB01003152;Np2015e|GJRU01072297;Cm2018|GJRL01003567
cluster10954
Nc2017|GJRH01051155;Nf_ref|GHLB01001537;Np2015e|GJRU01034266;Cm2018|GJRL01066988
cluster10967
Nc2017|GJRH01024642;Nf_ref|GHLB01018192;Np2015e|GJRU01033230;Cm2018|GJRL01005969
cluster10974
Nc2017|GJRH01012962;Nf_ref|GHLB01007392;Np2015e|GJRU01060387;Cm2018|GJRL01051149
cluster10976
Nc2017|GJRH01036265;Nf_ref|GHLB01021880;Np2015e|GJRU01047623;Cm2018|GJRL01076417
cluster10978
Nc2017|GJRH01033908;Nf_ref|GHLB01031063;Np2015e|GJRU01037644;Cm2018|GJRL01070627
cluster10981
Nc2017|GJRH01051874;Nf_ref|GHLB01036988;Np2015e|GJRU01043075;Cm2018|GJRL01023958
cluster10982
Nc2017|GJRH01044939;Nf_ref|GHLB01036095;Np2015e|GJRU01020075;Cm2018|GJRL01070690
cluster11173
Np2015e|GJRU01029535;Nf_ref|GHLB01016287;Cm2018|GJRL01039569;Nc2017|GJRH01030480
cluster11202
Np2015e|GJRU01027264;Nf_ref|GHLB01021286;Cm2018|GJRL01027020;Nc2017|GJRH01063394
cluster11203
Np2015e|GJRU01060473;Nf_ref|GHLB01001708;Cm2018|GJRL01078314;Nc2017|GJRH01072613
cluster11216
Np2015e|GJRU01036296;Nf_ref|GHLB01028589;Cm2018|GJRL01045848;Nc2017|GJRH01073184
cluster11217
Np2015e|GJRU01069321;Nf_ref|GHLB01036914;Cm2018|GJRL01022738;Nc2017|GJRH01010953
cluster11218
Np2015e|GJRU01053020;Nf_ref|GHLB01021693;Cm2018|GJRL01007243;Nc2017|GJRH01040883
cluster11221
Np2015e|GJRU01006666;Nf_ref|GHLB01042424;Cm2018|GJRL01021353;Nc2017|GJRH01034880
cluster11226
Np2015e|GJRU01054968;Nf_ref|GHLB01004479;Cm2018|GJRL01031543;Nc2017|GJRH01033466

cluster11227
Np2015e|GJRU01033420;Nf_ref|GHLB01021232;Cm2018|GJRL01066824;Nc2017|GJRH01044697
cluster11246
Np2015e|GJRU01057794;Nf_ref|GHLB01003944;Cm2018|GJRL01059682;Nc2017|GJRH01015017
cluster11248
Np2015e|GJRU01031657;Nf_ref|GHLB01050582;Cm2018|GJRL01072481;Nc2017|GJRH01069794
cluster11253
Np2015e|GJRU01006555;Nf_ref|GHLB01015212;Cm2018|GJRL01059208;Nc2017|GJRH01015149
cluster11254
Np2015e|GJRU01022967;Nf_ref|GHLB01045886;Cm2018|GJRL01001514;Nc2017|GJRH01062248
cluster11262
Np2015e|GJRU01022509;Nf_ref|GHLB01032166;Cm2018|GJRL01044385;Nc2017|GJRH01000562
cluster11265
Np2015e|GJRU01065129;Nf_ref|GHLB01021063;Cm2018|GJRL01070323;Nc2017|GJRH01060062
cluster11267
Np2015e|GJRU01064443;Nf_ref|GHLB01050790;Cm2018|GJRL01079979;Nc2017|GJRH01058137
cluster11271
Np2015e|GJRU01054834;Nf_ref|GHLB01036459;Cm2018|GJRL01024018;Nc2017|GJRH01022863
cluster11274
Np2015e|GJRU01042643;Nf_ref|GHLB01017452;Cm2018|GJRL01044777;Nc2017|GJRH01065710

Neo_cluster_name protein_list
cluster203
Nc2017|GJRH01002408;Nc2017|GJRH01002417;Np2015e|GJRU01024278;Np2015e|GJRU01024288;Np2015e|GJRU01024291;Np2015e|GJRU01024292;Np2015e|GJRU01024296;Np2015e|GJRU01024297;Np2015e|GJRU01024277;Np2015e|GJRU01024282;Np2015e|GJRU01024283;Np2015e|GJRU01024289;Np2015e|GJRU01024300;Np2015e|GJRU01024301;Np2015e|GJRU01024280;Np2015e|GJRU01024287;Np2015e|GJRU01024279;Np2015e|GJRU01024281;Np2015e|GJRU01024284;Np2015e|GJRU01024276;Nc2017|GJRH01011481;Nf_ref|GHLB01010636
cluster360
Nc2017|GJRH01012907;Nc2017|GJRH01012913;Nc2017|GJRH01012915;Nc2017|GJRH01012919;Nc2017|GJRH01012922;Nc2017|GJRH01012904;Nc2017|GJRH01012905;Nc2017|GJRH01012908;Nc2017|GJRH01012910;Nc2017|GJRH01012911;Nc2017|GJRH01012912;Nf_ref|GHLB01047524;Nf_ref|GHLB01047522;Nf_ref|GHLB01047520;Nf_ref|GHLB01047518;Np2015e|GJRU01027078;Np2015e|GJRU01027080;Np2015e|GJRU01027077;Np2015e|GJRU01027075
cluster362
Nc2017|GJRH01036857;Nc2017|GJRH01036856;Nc2017|GJRH01036858;Nc2017|GJRH01036859;Nc2017|GJRH01036860;Nc2017|GJRH01036862;Np2015e|GJRU01069566;Nf_ref|GHLB01020635;Nf_ref|GHLB01020618;Nf_ref|GHLB01020634;Nf_ref|GHLB01020629;Nf_ref|GHLB01020627;Nf_ref|GHLB01020624;Nf_ref|GHLB01020621;Nf_re

f|GHLB01020620;Nf_ref|GHLB01020633;Nf_ref|GHLB01020631;Nf_ref|GHLB0102063
0;Nf_ref|GHLB01020628
cluster540
Nc2017|GJRH01070320;Nc2017|GJRH01070319;Nc2017|GJRH01070322;Nc2017|
GJRH01070323;Np2015e|GJRU01061654;Np2015e|GJRU01061651;Np2015e|GJRU010616
49;Np2015e|GJRU01061645;Np2015e|GJRU01061655;Np2015e|GJRU01061652;Np2015e
|GJRU01061646;Nf_ref|GHLB01029509;Nc2017|GJRH01070324;Nc2017|GJRH01070318
;Nf_ref|GHLB01029512;Nf_ref|GHLB01029513;Nf_ref|GHLB01029514
cluster1040
Nc2017|GJRH01088177;Nc2017|GJRH01088168;Nc2017|GJRH01088165;Nc2017|
GJRH01088172;Nc2017|GJRH01088166;Np2015e|GJRU01029343;Np2015e|GJRU0102934
2;Np2015e|GJRU01029345;Np2015e|GJRU01029346;Np2015e|GJRU01029347;Np2015e|
GJRU01029348;Nf_ref|GHLB01010338;Nf_ref|GHLB01010339;Np2015e|GJRU01029344
cluster1318
Nc2017|GJRH01043243;Nc2017|GJRH01043248;Nc2017|GJRH01043247;Nc2017|
GJRH01043244;Nc2017|GJRH01043242;Nc2017|GJRH01043241;Nc2017|GJRH01043253;
Nc2017|GJRH01043251;Nc2017|GJRH01043254;Np2015e|GJRU01028043;Nf_ref|GHLB0
1035502;Nf_ref|GHLB01035498;Np2015e|GJRU01028044
cluster1323
Nc2017|GJRH01089905;Nc2017|GJRH01089906;Nc2017|GJRH01089910;Nc2017|
GJRH01089893;Nc2017|GJRH01089895;Nc2017|GJRH01089897;Nc2017|GJRH01089898;
Nc2017|GJRH01089900;Nc2017|GJRH01089901;Nc2017|GJRH01089904;Nf_ref|GHLB01
049043;Nf_ref|GHLB01049042;Np2015e|GJRU01008881
cluster1331
Nc2017|GJRH01003977;Nc2017|GJRH01003970;Nc2017|GJRH01003971;Nc2017|
GJRH01003973;Nc2017|GJRH01003975;Nc2017|GJRH01003976;Nf_ref|GHLB01016593;
Nf_ref|GHLB01016591;Nf_ref|GHLB01016585;Np2015e|GJRU01071095;Np2015e|GJRU
01071093;Np2015e|GJRU01071092;Np2015e|GJRU01071094
cluster2058
Nc2017|GJRH01073024;Nc2017|GJRH01073023;Nf_ref|GHLB01033450;Np2015e
|GJRU01062470;Np2015e|GJRU01062466;Np2015e|GJRU01062464;Np2015e|GJRU01062
469;Np2015e|GJRU01062463;Np2015e|GJRU01062465;Np2015e|GJRU01062467;Np2015
e|GJRU01062468
cluster2067
Np2015e|GJRU01009078;Np2015e|GJRU01009077;Nc2017|GJRH01089363;Nf_re
f|GHLB01033989;Nc2017|GJRH01089357;Nf_ref|GHLB01033996;Nf_ref|GHLB0103399
5;Nf_ref|GHLB01033993;Nf_ref|GHLB01033992;Nf_ref|GHLB01033987;Nf_ref|GHLB
01034002
cluster2377
Nc2017|GJRH01088679;Nc2017|GJRH01088680;Np2015e|GJRU01044141;Np2015
e|GJRU01044134;Np2015e|GJRU01044135;Np2015e|GJRU01044138;Nf_ref|GHLB01009
063;Nf_ref|GHLB01009058;Nf_ref|GHLB01009060;Nf_ref|GHLB01009064
cluster2613
Nc2017|GJRH01042807;Nc2017|GJRH01042808;Nc2017|GJRH01042809;Nc2017|
GJRH01042812;Nc2017|GJRH01042814;Nc2017|GJRH01042815;Np2015e|GJRU01055147
;Np2015e|GJRU01055146;Nf_ref|GHLB01051394;Nf_ref|GHLB01051393
cluster2623
Nc2017|GJRH01005007;Nc2017|GJRH01005004;Nf_ref|GHLB01009111;Np2015e
|GJRU01066252;Np2015e|GJRU01066250;Np2015e|GJRU01066254;Np2015e|GJRU01066
253;Np2015e|GJRU01066249;Nf_ref|GHLB01009113;Np2015e|GJRU01066251
cluster2627
Nc2017|GJRH01013241;Nc2017|GJRH01013238;Nc2017|GJRH01070149;Np2015e
|GJRU01050312;Np2015e|GJRU01050321;Np2015e|GJRU01050317;Np2015e|GJRU01050
318;Np2015e|GJRU01050319;Nf_ref|GHLB01029643;Nf_ref|GHLB01029649

cluster2639
Np2015e|GJRU01009886;Np2015e|GJRU01009888;Np2015e|GJRU01009887;Np2015e|GJRU01009889;Np2015e|GJRU01009884;Np2015e|GJRU01009885;Nf_ref|GHLB01036028;Nf_ref|GHLB01036029;Nc2017|GJRH01059222;Nc2017|GJRH01059223
cluster2932
Nf_ref|GHLB01008164;Np2015e|GJRU01045690;Nc2017|GJRH01049229;Np2015e|GJRU01045698;Np2015e|GJRU01045697;Np2015e|GJRU01045692;Np2015e|GJRU01045691;Np2015e|GJRU01045693;Np2015e|GJRU01045699
cluster3326
Nc2017|GJRH01054408;Nc2017|GJRH01054407;Nf_ref|GHLB01008024;Np2015e|GJRU01020203;Np2015e|GJRU01020198;Np2015e|GJRU01020202;Np2015e|GJRU01020199;Np2015e|GJRU01020200;Np2015e|GJRU01020201
cluster3342
Nc2017|GJRH01015892;Nc2017|GJRH01015888;Nc2017|GJRH01015889;Nc2017|GJRH01015890;Nc2017|GJRH01015891;Nf_ref|GHLB01027664;Np2015e|GJRU01034200;Np2015e|GJRU01034199;Np2015e|GJRU01034198
cluster3353
Nc2017|GJRH01073020;Nf_ref|GHLB01036959;Nf_ref|GHLB01036957;Np2015e|GJRU01052268;Np2015e|GJRU01052267;Np2015e|GJRU01052269;Nf_ref|GHLB01036955;Nf_ref|GHLB01036956;Nf_ref|GHLB01036958
cluster4196
Nf_ref|GHLB01024871;Nf_ref|GHLB01024870;Np2015e|GJRU01050711;Np2015e|GJRU01050710;Np2015e|GJRU01021042;Np2015e|GJRU01021043;Nc2017|GJRH01070129;Nc2017|GJRH01070138
cluster4201
Np2015e|GJRU01014540;Np2015e|GJRU01014541;Nf_ref|GHLB01001706;Nc2017|GJRH01013263;Nc2017|GJRH01013260;Nc2017|GJRH01013268;Nc2017|GJRH01013261;Nc2017|GJRH01013269
cluster4212
Nc2017|GJRH01015959;Nc2017|GJRH01015953;Nc2017|GJRH01015954;Nc2017|GJRH01015956;Nc2017|GJRH01015957;Nc2017|GJRH01015961;Nf_ref|GHLB01000460;Np2015e|GJRU01004417
cluster4216
Nc2017|GJRH01078982;Nc2017|GJRH01078981;Nc2017|GJRH01078979;Nc2017|GJRH01078980;Nc2017|GJRH01078983;Np2015e|GJRU01011572;Nf_ref|GHLB01009472;Np2015e|GJRU01011571
cluster4220
Nc2017|GJRH01033815;Nc2017|GJRH01033816;Nc2017|GJRH01033819;Nf_ref|GHLB01004586;Np2015e|GJRU01069666;Np2015e|GJRU01069665;Nf_ref|GHLB01004589;Nf_ref|GHLB01004588
cluster4227
Nc2017|GJRH01023759;Nc2017|GJRH01023761;Nc2017|GJRH01023762;Nc2017|GJRH01023763;Nf_ref|GHLB01036114;Nf_ref|GHLB01036111;Np2015e|GJRU01046681;Nf_ref|GHLB01036113
cluster4231
Nc2017|GJRH01043271;Nc2017|GJRH01043267;Nc2017|GJRH01043270;Nc2017|GJRH01043274;Nc2017|GJRH01043276;Nf_ref|GHLB01044514;Np2015e|GJRU01057724;Np2015e|GJRU01057720
cluster4234
Nc2017|GJRH01040507;Nc2017|GJRH01040506;Nc2017|GJRH01040508;Nc2017|GJRH01040512;Nc2017|GJRH01040513;Np2015e|GJRU01035915;Np2015e|GJRU01035914;Nf_ref|GHLB01011470
cluster4273
Np2015e|GJRU01060102;Np2015e|GJRU01060105;Nf_ref|GHLB01025062;Nc201

7|GJRH01089844;Nc2017|GJRH01089851;Nc2017|GJRH01089847;Nc2017|GJRH0108985
5;Nc2017|GJRH01089846
cluster4726
Nf_ref|GHLB01002734;Nc2017|GJRH01037143;Nc2017|GJRH01037145;Np2015e
|GJRU01046979;Np2015e|GJRU01046980;Np2015e|GJRU01046978;Np2015e|GJRU01046
977
cluster4751
Nf_ref|GHLB01010156;Nf_ref|GHLB01010157;Nf_ref|GHLB01010158;Nf_ref|
GHLB01010160;Nc2017|GJRH01076435;Np2015e|GJRU01018210;Np2015e|GJRU0101820
7
cluster5314
Np2015e|GJRU01073355;Nc2017|GJRH01068495;Nc2017|GJRH01068496;Nc2017
|GJRH01068497;Nc2017|GJRH01068501;Nf_ref|GHLB01005611;Nf_ref|GHLB01005606
cluster5378
Nf_ref|GHLB01005989;Np2015e|GJRU01057842;Nc2017|GJRH01011002;Nc2017
|GJRH01010995;Nc2017|GJRH01010996;Nc2017|GJRH01010999;Nf_ref|GHLB01005990
cluster5380
Nc2017|GJRH01046910;Nc2017|GJRH01046913;Nc2017|GJRH01046914;Nc2017|
GJRH01046915;Nf_ref|GHLB01028031;Nf_ref|GHLB01028034;Np2015e|GJRU01068761
cluster5411
Nc2017|GJRH01051239;Nc2017|GJRH01051238;Nc2017|GJRH01051240;Nc2017|
GJRH01051241;Nf_ref|GHLB01003643;Nf_ref|GHLB01003641;Np2015e|GJRU01027896
cluster5424
Nc2017|GJRH01091141;Nf_ref|GHLB01003454;Np2015e|GJRU01016907;Np2015
e|GJRU01016906;Np2015e|GJRU01016909;Np2015e|GJRU01016912;Np2015e|GJRU0101
6908
cluster5446
Np2015e|GJRU01046035;Np2015e|GJRU01046034;Np2015e|GJRU01046037;Nf_r
ef|GHLB01034705;Nc2017|GJRH01063388;Nc2017|GJRH01063390;Nf_ref|GHLB010347
06
cluster5458
Np2015e|GJRU01023322;Np2015e|GJRU01023323;Np2015e|GJRU01023324;Nf_r
ef|GHLB01003674;Nc2017|GJRH01041668;Nc2017|GJRH01041666;Nc2017|GJRH010416
60
cluster5467
Np2015e|GJRU01055187;Np2015e|GJRU01055188;Nf_ref|GHLB01004958;Nf_re
f|GHLB01004957;Nc2017|GJRH01000565;Nc2017|GJRH01000564;Nc2017|GJRH0100056
3
cluster5469
Np2015e|GJRU01040356;Nf_ref|GHLB01022819;Np2015e|GJRU01034930;Nf_re
f|GHLB01022818;Nf_ref|GHLB01022816;Nc2017|GJRH01005449;Nc2017|GJRH0100544
6
cluster5475
Np2015e|GJRU01014535;Nf_ref|GHLB01006979;Nc2017|GJRH01002031;Nf_ref
|GHLB01006975;Nf_ref|GHLB01006981;Nf_ref|GHLB01006973;Nf_ref|GHLB01006977
cluster5944
Np2015e|GJRU01047135;Np2015e|GJRU01047134;Nc2017|GJRH01063349;Nf_re
f|GHLB01014663;Nc2017|GJRH01063351;Nf_ref|GHLB01014664
cluster6067
Nc2017|GJRH01041873;Np2015e|GJRU01038062;Np2015e|GJRU01038060;Np201
5e|GJRU01038057;Nf_ref|GHLB01033336;Nc2017|GJRH01041868
cluster6288
Nf_ref|GHLB01027927;Np2015e|GJRU01020027;Np2015e|GJRU01020028;Np201
5e|GJRU01020029;Nf_ref|GHLB01027931;Nc2017|GJRH01045821

cluster6799
Nc2017|GJRH01083002;Nf_ref|GHLB01021878;Nf_ref|GHLB01021877;Np2015e|GJRU01019410;Np2015e|GJRU01019408;Np2015e|GJRU01019409
cluster6810
Nc2017|GJRH01018382;Nc2017|GJRH01018381;Nf_ref|GHLB01019901;Np2015e|GJRU01030971;Np2015e|GJRU01030974;Nf_ref|GHLB01019909
cluster6827
Np2015e|GJRU01010571;Nc2017|GJRH01008691;Np2015e|GJRU01010572;Nf_ref|GHLB01016361;Nf_ref|GHLB01016359;Nf_ref|GHLB01016366
cluster6921
Nc2017|GJRH01036936;Nc2017|GJRH01036931;Nc2017|GJRH01036934;Nf_ref|GHLB01025992;Np2015e|GJRU01045519;Np2015e|GJRU01045518
cluster6942
Nc2017|GJRH01021484;Nc2017|GJRH01021485;Nf_ref|GHLB01010306;Nf_ref|GHLB01010304;Np2015e|GJRU01073309;Nc2017|GJRH01073446
cluster6949
Nc2017|GJRH01013631;Nc2017|GJRH01013633;Nf_ref|GHLB01011073;Nf_ref|GHLB01011069;Np2015e|GJRU01036361;Np2015e|GJRU01036360
cluster6950
Nc2017|GJRH01061234;Nc2017|GJRH01061225;Np2015e|GJRU01051520;Np2015e|GJRU01051519;Nf_ref|GHLB01002166;Nf_ref|GHLB01002171
cluster6957
Nc2017|GJRH01036738;Nc2017|GJRH01036744;Nf_ref|GHLB01014400;Nf_ref|GHLB01014391;Np2015e|GJRU01014758;Np2015e|GJRU01014757
cluster6965
Nc2017|GJRH01046926;Nc2017|GJRH01046925;Nc2017|GJRH01046934;Nc2017|GJRH01046939;Np2015e|GJRU01042507;Nf_ref|GHLB01043065
cluster6973
Nc2017|GJRH01058331;Nc2017|GJRH01058337;Nf_ref|GHLB01035855;Np2015e|GJRU01055414;Np2015e|GJRU01055413;Nf_ref|GHLB01035856
cluster6982
Nc2017|GJRH01080831;Nc2017|GJRH01080830;Nc2017|GJRH01080832;Nf_ref|GHLB01005631;Np2015e|GJRU01032981;Np2015e|GJRU01032979
cluster6992
Nc2017|GJRH01044077;Np2015e|GJRU01060108;Np2015e|GJRU01060107;Nf_ref|GHLB01024334;Nf_ref|GHLB01024332;Nf_ref|GHLB01024326
cluster6993
Nc2017|GJRH01071805;Np2015e|GJRU01060156;Np2015e|GJRU01060155;Nf_ref|GHLB01016641;Nc2017|GJRH01071807;Nc2017|GJRH01071800
cluster7015
Nc2017|GJRH01088252;Nf_ref|GHLB01014079;Np2015e|GJRU01060563;Np2015e|GJRU01060564;Np2015e|GJRU01060565;Nf_ref|GHLB01014080
cluster7038
Np2015e|GJRU01055623;Np2015e|GJRU01055622;Nf_ref|GHLB01014846;Nc2017|GJRH01069429;Nf_ref|GHLB01014844;Nf_ref|GHLB01014845
cluster7049
Np2015e|GJRU01015927;Np2015e|GJRU01015920;Np2015e|GJRU01015925;Np2015e|GJRU01015926;Nf_ref|GHLB01044243;Nc2017|GJRH01008840
cluster7053
Np2015e|GJRU01027425;Np2015e|GJRU01027424;Nf_ref|GHLB01002974;Nc2017|GJRH01007963;Nc2017|GJRH01007962;Nf_ref|GHLB01002972
cluster7061
Np2015e|GJRU01044463;Np2015e|GJRU01044461;Np2015e|GJRU01044462;Nf_ref|GHLB01047719;Nc2017|GJRH01045760;Nc2017|GJRH01045758

cluster7075
Np2015e|GJRU01039122;Nf_ref|GHLB01011900;Nf_ref|GHLB01011899;Nc2017|GJRH01071138;Nc2017|GJRH01071141;Nc2017|GJRH01071139
cluster7529
Nf_ref|GHLB01018085;Nc2017|GJRH01061590;Np2015e|GJRU01027410;Np2015e|GJRU01027408;Np2015e|GJRU01027409
cluster7590
Nf_ref|GHLB01028249;Np2015e|GJRU01007448;Nc2017|GJRH01029186;Nf_ref|GHLB01028244;Np2015e|GJRU01007447
cluster7939
Nf_ref|GHLB01039939;Nc2017|GJRH01036431;Np2015e|GJRU01035880;Np2015e|GJRU01035875;Np2015e|GJRU01035877
cluster7992
Nc2017|GJRH01044991;Nc2017|GJRH01044999;Nf_ref|GHLB01004007;Np2015e|GJRU01015193;Np2015e|GJRU01015188
cluster8187
Nf_ref|GHLB01022093;Np2015e|GJRU01011239;Nc2017|GJRH01077250;Nc2017|GJRH01077249;Nc2017|GJRH01077251
cluster8498
Nf_ref|GHLB01018689;Nf_ref|GHLB01018691;Np2015e|GJRU01070684;Np2015e|GJRU01070683;Nc2017|GJRH01085855
cluster8502
Nf_ref|GHLB01051090;Np2015e|GJRU01033152;Np2015e|GJRU01033148;Nc2017|GJRH01053065;Np2015e|GJRU01033149
cluster8587
Np2015e|GJRU01057398;Np2015e|GJRU01057395;Nc2017|GJRH01062743;Nf_ref|GHLB01009033;Np2015e|GJRU01057394
cluster8606
Nc2017|GJRH01073443;Nc2017|GJRH01073442;Nf_ref|GHLB01018394;Nf_ref|GHLB01018391;Np2015e|GJRU01031561
cluster8626
Nc2017|GJRH01064250;Nc2017|GJRH01064251;Nf_ref|GHLB01037074;Nc2017|GJRH01064252;Np2015e|GJRU01003580
cluster8627
Nc2017|GJRH01037444;Nc2017|GJRH01037447;Np2015e|GJRU01011420;Nc2017|GJRH01037439;Nf_ref|GHLB01041718
cluster8639
Nc2017|GJRH01079436;Nc2017|GJRH01079435;Nc2017|GJRH01079437;Nf_ref|GHLB01042971;Np2015e|GJRU01038594
cluster8643
Nc2017|GJRH01021511;Nc2017|GJRH01021508;Nf_ref|GHLB01050525;Np2015e|GJRU01060875;Np2015e|GJRU01060876
cluster8655
Nc2017|GJRH01038356;Nc2017|GJRH01038358;Nf_ref|GHLB01010130;Nf_ref|GHLB01010128;Np2015e|GJRU01072836
cluster8669
Nc2017|GJRH01025689;Nc2017|GJRH01025690;Nf_ref|GHLB01012721;Np2015e|GJRU01028146;Nf_ref|GHLB01012726
cluster8694
Nc2017|GJRH01039789;Nc2017|GJRH01039786;Nf_ref|GHLB01032578;Np2015e|GJRU01057157;Nf_ref|GHLB01032575
cluster8710
Nc2017|GJRH01068194;Nc2017|GJRH01068195;Nc2017|GJRH01068201;Nf_ref|GHLB01004964;Np2015e|GJRU01025746

cluster8712
Nc2017|GJRH01077433;Nc2017|GJRH01077427;Nc2017|GJRH01077432;Nf_ref|GHLB01011519;Np2015e|GJRU01005851
cluster8737
Nc2017|GJRH01054438;Nf_ref|GHLB01017281;Np2015e|GJRU01045648;Nc2017|GJRH01054443;Nc2017|GJRH01054437
cluster8766
Nc2017|GJRH01008708;Nf_ref|GHLB01050595;Np2015e|GJRU01025956;Np2015e|GJRU01025955;Nf_ref|GHLB01050598
cluster8815
Np2015e|GJRU01057270;Np2015e|GJRU01057268;Np2015e|GJRU01057273;Nf_ref|GHLB01018695;Nc2017|GJRH01053516
cluster8823
Np2015e|GJRU01001077;Np2015e|GJRU01001076;Nf_ref|GHLB01001568;Nc2017|GJRH01063629;Nf_ref|GHLB01001569
cluster8837
Np2015e|GJRU01007549;Np2015e|GJRU01007551;Nf_ref|GHLB01001713;Nc2017|GJRH01016591;Nf_ref|GHLB01001712
cluster8845
Np2015e|GJRU01063835;Np2015e|GJRU01063836;Np2015e|GJRU01063838;Nc2017|GJRH01044963;Nf_ref|GHLB01001137
cluster8846
Np2015e|GJRU01041544;Np2015e|GJRU01041545;Nf_ref|GHLB01039499;Nc2017|GJRH01038672;Nc2017|GJRH01038670
cluster8854
Np2015e|GJRU01043131;Np2015e|GJRU01043132;Nf_ref|GHLB01032650;Nc2017|GJRH01052165;Nf_ref|GHLB01032649
cluster8860
Np2015e|GJRU01021685;Np2015e|GJRU01021683;Nf_ref|GHLB01004737;Nc2017|GJRH01050383;Nf_ref|GHLB01004739
cluster8877
Np2015e|GJRU01042506;Nf_ref|GHLB01043066;Nc2017|GJRH01046937;Nf_ref|GHLB01043062;Nf_ref|GHLB01043063
cluster8880
Np2015e|GJRU01062827;Nc2017|GJRH01048957;Nc2017|GJRH01048956;Np2015e|GJRU01062828;Nf_ref|GHLB01030829
cluster8901
Np2015e|GJRU01034323;Nf_ref|GHLB01043708;Nc2017|GJRH01078030;Nc2017|GJRH01078032;Nf_ref|GHLB01043707
cluster8904
Np2015e|GJRU01024923;Nf_ref|GHLB01017252;Nc2017|GJRH01077794;Nc2017|GJRH01077795;Nf_ref|GHLB01017254
cluster8908
Np2015e|GJRU01073805;Nc2017|GJRH01056157;Np2015e|GJRU01073804;Nf_ref|GHLB01051436;Nc2017|GJRH01056156
cluster9503
Np2015e|GJRU01062432;Nc2017|GJRH01042229;Nc2017|GJRH01042231;Nf_ref|GHLB01035467
cluster9592
Np2015e|GJRU01056558;Nc2017|GJRH01041048;Nc2017|GJRH01041049;Nf_ref|GHLB01013027
cluster9624
Np2015e|GJRU01041320;Np2015e|GJRU01041319;Nc2017|GJRH01040370;Nf_ref|GHLB01044420

cluster9861
Nc2017|GJRH01020893;Np2015e|GJRU01019319;Np2015e|GJRU01019318;Nf_ref|GHLB01024209
cluster9892
Np2015e|GJRU01058028;Nf_ref|GHLB01021751;Nc2017|GJRH01015859;Nc2017|GJRH01015857
cluster10117
Np2015e|GJRU01068073;Nc2017|GJRH01016924;Nc2017|GJRH01016925;Nf_ref|GHLB01026228
cluster10693
Nf_ref|GHLB01009970;Nc2017|GJRH01046790;Nc2017|GJRH01046789;Np2015e|GJRU01032544
cluster10700
Nc2017|GJRH01019383;Nc2017|GJRH01019385;Np2015e|GJRU01040264;Nf_ref|GHLB01012626
cluster10736
Nc2017|GJRH01035184;Nc2017|GJRH01035185;Nf_ref|GHLB01028691;Np2015e|GJRU01022936
cluster10747
Nc2017|GJRH01069213;Nc2017|GJRH01069214;Np2015e|GJRU01010979;Nf_ref|GHLB01043659
cluster10753
Nc2017|GJRH01039915;Nc2017|GJRH01039916;Nf_ref|GHLB01051405;Np2015e|GJRU01012172
cluster10759
Nc2017|GJRH01004052;Nc2017|GJRH01004056;Nf_ref|GHLB01026600;Np2015e|GJRU01028715
cluster10764
Nc2017|GJRH01063496;Nc2017|GJRH01063497;Nf_ref|GHLB01042781;Np2015e|GJRU01031246
cluster10803
Nc2017|GJRH01060976;Nc2017|GJRH01060977;Np2015e|GJRU01029189;Nf_ref|GHLB01041010
cluster10806
Nc2017|GJRH01047091;Nc2017|GJRH01047082;Nf_ref|GHLB01024954;Np2015e|GJRU01029429
cluster10814
Nc2017|GJRH01012976;Nc2017|GJRH01012977;Nf_ref|GHLB01029004;Np2015e|GJRU01024668
cluster10843
Nc2017|GJRH01079919;Nc2017|GJRH01079916;Nf_ref|GHLB01015429;Np2015e|GJRU01030933
cluster10854
Nc2017|GJRH01089435;Nc2017|GJRH01089434;Np2015e|GJRU01021783;Nf_ref|GHLB01014602
cluster10904
Nc2017|GJRH01052218;Nc2017|GJRH01052221;Nf_ref|GHLB01027914;Np2015e|GJRU01055589
cluster10906
Nc2017|GJRH01073874;Nf_ref|GHLB01030656;Nf_ref|GHLB01030655;Np2015e|GJRU01008167
cluster10920
Nc2017|GJRH01068180;Nf_ref|GHLB01019786;Nf_ref|GHLB01019803;Np2015e|GJRU01060025

cluster10943
Nc2017|GJRH01023566;Nf_ref|GHLB01005320;Np2015e|GJRU01057187;Np2015e|GJRU01057189
cluster10953
Nc2017|GJRH01081804;Nf_ref|GHLB01026723;Np2015e|GJRU01063780;Nf_ref|GHLB01026722
cluster10973
Nc2017|GJRH01077967;Nf_ref|GHLB01044826;Np2015e|GJRU01007920;Nf_ref|GHLB01044827
cluster11018
Np2015e|GJRU01067469;Np2015e|GJRU01067468;Nf_ref|GHLB01015513;Nc2017|GJRH01071334
cluster11031
Np2015e|GJRU01072519;Np2015e|GJRU01072520;Nf_ref|GHLB01024031;Nc2017|GJRH01037548
cluster11036
Np2015e|GJRU01052816;Np2015e|GJRU01052817;Nf_ref|GHLB01001197;Nc2017|GJRH01087808
cluster11045
Np2015e|GJRU01027601;Np2015e|GJRU01027602;Nf_ref|GHLB01027320;Nc2017|GJRH01073561
cluster11051
Np2015e|GJRU01044140;Np2015e|GJRU01044143;Nf_ref|GHLB01009061;Nc2017|GJRH01088678
cluster11079
Np2015e|GJRU01005039;Np2015e|GJRU01005038;Nf_ref|GHLB01035766;Nc2017|GJRH01015946
cluster11105
Np2015e|GJRU01035809;Np2015e|GJRU01035811;Nf_ref|GHLB01016700;Nc2017|GJRH01018784
cluster11112
Np2015e|GJRU01048803;Np2015e|GJRU01048802;Nf_ref|GHLB01025363;Nc2017|GJRH01073370
cluster11120
Np2015e|GJRU01028534;Np2015e|GJRU01028532;Nf_ref|GHLB01033230;Nc2017|GJRH01073192
cluster11129
Np2015e|GJRU01041273;Np2015e|GJRU01041274;Nf_ref|GHLB01022398;Nc2017|GJRH01014361
cluster11148
Np2015e|GJRU01062654;Np2015e|GJRU01062656;Nf_ref|GHLB01036222;Nc2017|GJRH01024063
cluster11158
Np2015e|GJRU01029432;Np2015e|GJRU01029433;Nf_ref|GHLB01049082;Nc2017|GJRH01057640
cluster11166
Np2015e|GJRU01057123;Nf_ref|GHLB01030322;Nc2017|GJRH01032661;Nf_ref|GHLB01030325
cluster11167
Np2015e|GJRU01002679;Nf_ref|GHLB01024822;Nf_ref|GHLB01024813;Nc2017|GJRH01084127
cluster11170
Np2015e|GJRU01000053;Nf_ref|GHLB01023517;Nc2017|GJRH01051227;Nf_ref|GHLB01023518

cluster11172
Np2015e|GJRU01062504;Nf_ref|GHLB01018511;Nc2017|GJRH01052346;Nc2017|GJRH01052343
cluster11184
Np2015e|GJRU01000945;Nf_ref|GHLB01043725;Nf_ref|GHLB01043724;Nc2017|GJRH01020632
cluster11188
Np2015e|GJRU01003940;Nf_ref|GHLB01005880;Nc2017|GJRH01003940;Nc2017|GJRH01003939
cluster11199
Np2015e|GJRU01026017;Nf_ref|GHLB01040251;Nc2017|GJRH01047362;Nf_ref|GHLB01040255
cluster11200
Np2015e|GJRU01002217;Nf_ref|GHLB01005279;Nc2017|GJRH01018015;Nc2017|GJRH01018014
cluster11205
Np2015e|GJRU01029187;Nf_ref|GHLB01004936;Nc2017|GJRH01036865;Nf_ref|GHLB01004934
cluster11206
Np2015e|GJRU01035217;Nf_ref|GHLB01008657;Nc2017|GJRH01020722;Nc2017|GJRH01020721
cluster11208
Np2015e|GJRU01069307;Nf_ref|GHLB01043916;Nf_ref|GHLB01043915;Nc2017|GJRH01022320
cluster11219
Np2015e|GJRU01013961;Nf_ref|GHLB01002202;Nc2017|GJRH01070663;Nc2017|GJRH01070659
cluster11223
Np2015e|GJRU01007614;Nf_ref|GHLB01046959;Nc2017|GJRH01059343;Np2015e|GJRU01007613
cluster11233
Np2015e|GJRU01053516;Nf_ref|GHLB01048513;Nc2017|GJRH01024079;Np2015e|GJRU01053516
cluster11234
Np2015e|GJRU01046363;Nf_ref|GHLB01005900;Nc2017|GJRH01055786;Nf_ref|GHLB01005901
cluster11245
Np2015e|GJRU01000012;Nf_ref|GHLB01044460;Nc2017|GJRH01048962;Nf_ref|GHLB01044459
cluster11257
Np2015e|GJRU01033799;Nf_ref|GHLB01001644;Nc2017|GJRH01058544;Nc2017|GJRH01058543
cluster11260
Np2015e|GJRU01038591;Nf_ref|GHLB01042973;Nc2017|GJRH01079439;Nc2017|GJRH01079438
cluster11263
Np2015e|GJRU01026019;Nf_ref|GHLB01040250;Nc2017|GJRH01047367;Nf_ref|GHLB01040254
cluster11264
Np2015e|GJRU01039307;Nf_ref|GHLB01007940;Nf_ref|GHLB01007939;Nc2017|GJRH01076316
cluster11897
Nf_ref|GHLB01050575;Nc2017|GJRH01081317;Np2015e|GJRU01056080

cluster11961
Nc2017|GJRH01059410;Np2015e|GJRU01020335;Nf_ref|GHLB01015300
cluster12012
Nf_ref|GHLB01009486;Np2015e|GJRU01067713;Nc2017|GJRH01016335
cluster12376
Nf_ref|GHLB01047911;Nc2017|GJRH01075495;Np2015e|GJRU01069757
cluster12889
Nf_ref|GHLB01032180;Np2015e|GJRU01018652;Nc2017|GJRH01004670
cluster12890
Np2015e|GJRU01057326;Nc2017|GJRH01029532;Nf_ref|GHLB01014675
cluster12899
Nc2017|GJRH01074621;Nf_ref|GHLB01027362;Np2015e|GJRU01024914
cluster12915
Nc2017|GJRH01075295;Np2015e|GJRU01001558;Nf_ref|GHLB01036346
cluster12970
Nf_ref|GHLB01005492;Np2015e|GJRU01068169;Nc2017|GJRH01084804
cluster13036
Np2015e|GJRU01063597;Nf_ref|GHLB01015491;Nc2017|GJRH01078213
cluster13093
Nf_ref|GHLB01019064;Np2015e|GJRU01055411;Nc2017|GJRH01060716
cluster13123
Nc2017|GJRH01045594;Np2015e|GJRU01025615;Nf_ref|GHLB01040295
cluster13160
Np2015e|GJRU01061638;Nf_ref|GHLB01043705;Nc2017|GJRH01083933
cluster13161
Nf_ref|GHLB01001844;Nc2017|GJRH01069601;Np2015e|GJRU01064699
cluster13420
Nc2017|GJRH01013691;Nf_ref|GHLB01049482;Np2015e|GJRU01041566
cluster13424
Nc2017|GJRH01034853;Nf_ref|GHLB01041343;Np2015e|GJRU01063500
cluster13427
Nc2017|GJRH01057606;Nf_ref|GHLB01026879;Np2015e|GJRU01043095
cluster13428
Nc2017|GJRH01003929;Nf_ref|GHLB01036380;Np2015e|GJRU01057276
cluster13432
Nc2017|GJRH01035931;Nf_ref|GHLB01030846;Np2015e|GJRU01025152
cluster13449
Nc2017|GJRH01000181;Nf_ref|GHLB01041379;Np2015e|GJRU01055084
cluster13453
Nc2017|GJRH01015990;Nf_ref|GHLB01016736;Np2015e|GJRU01018645
cluster13458
Nc2017|GJRH01006917;Nf_ref|GHLB01001335;Np2015e|GJRU01047259
cluster13461
Nc2017|GJRH01025808;Nf_ref|GHLB01006009;Np2015e|GJRU01070049
cluster13462
Nc2017|GJRH01077808;Nf_ref|GHLB01036689;Np2015e|GJRU01009703
cluster13464
Nc2017|GJRH01076945;Nf_ref|GHLB01042432;Np2015e|GJRU01072356
cluster13465
Nc2017|GJRH01084425;Nf_ref|GHLB01003591;Np2015e|GJRU01015018
cluster13467
Nc2017|GJRH01048996;Nf_ref|GHLB01023152;Np2015e|GJRU01030962
cluster13472
Nc2017|GJRH01052349;Nf_ref|GHLB01018518;Np2015e|GJRU01062501

cluster13486
Nc2017|GJRH01039298;Nf_ref|GHLB01013771;Np2015e|GJRU01064084
cluster13500
Nc2017|GJRH01031752;Nf_ref|GHLB01034689;Np2015e|GJRU01036843
cluster13534
Nc2017|GJRH01021431;Nf_ref|GHLB01043999;Np2015e|GJRU01047776
cluster13539
Nc2017|GJRH01000084;Nf_ref|GHLB01046298;Np2015e|GJRU01023638
cluster13545
Nc2017|GJRH01066331;Nf_ref|GHLB01000117;Np2015e|GJRU01020323
cluster13574
Nc2017|GJRH01086782;Nf_ref|GHLB01004202;Np2015e|GJRU01034912
cluster13812
Np2015e|GJRU01006212;Nf_ref|GHLB01000458;Nc2017|GJRH01011500
cluster13813
Np2015e|GJRU01047725;Nf_ref|GHLB01026157;Nc2017|GJRH01068491
cluster13818
Np2015e|GJRU01055936;Nf_ref|GHLB01011299;Nc2017|GJRH01058894
cluster13820
Np2015e|GJRU01031809;Nf_ref|GHLB01033675;Nc2017|GJRH01087634
cluster13825
Np2015e|GJRU01004824;Nf_ref|GHLB01023921;Nc2017|GJRH01081833
cluster13834
Np2015e|GJRU01044596;Nf_ref|GHLB01011933;Nc2017|GJRH01050288
cluster13837
Np2015e|GJRU01046077;Nf_ref|GHLB01012160;Nc2017|GJRH01035731
cluster13840
Np2015e|GJRU01014514;Nf_ref|GHLB01004729;Nc2017|GJRH01075557
cluster13841
Np2015e|GJRU01065306;Nf_ref|GHLB01009208;Nc2017|GJRH01015094
cluster13846
Np2015e|GJRU01041497;Nf_ref|GHLB01048159;Nc2017|GJRH01051504
cluster13854
Np2015e|GJRU01030298;Nc2017|GJRH01055128;Nf_ref|GHLB01030060
cluster13857
Np2015e|GJRU01072489;Nf_ref|GHLB01021242;Nc2017|GJRH01023146
cluster13861
Np2015e|GJRU01046889;Nf_ref|GHLB01042433;Nc2017|GJRH01035960
cluster13862
Np2015e|GJRU01016449;Nf_ref|GHLB01046124;Nc2017|GJRH01027145
cluster13871
Np2015e|GJRU01053037;Nf_ref|GHLB01031067;Nc2017|GJRH01058471
cluster13877
Np2015e|GJRU01064181;Nf_ref|GHLB01027249;Nc2017|GJRH01011914
cluster13882
Np2015e|GJRU01072266;Nf_ref|GHLB01037252;Nc2017|GJRH01055696
cluster13886
Np2015e|GJRU01001432;Nf_ref|GHLB01000705;Nc2017|GJRH01059643
cluster13890
Np2015e|GJRU01065730;Nf_ref|GHLB01011604;Nc2017|GJRH01081106
cluster13893
Np2015e|GJRU01031733;Nf_ref|GHLB01003651;Nc2017|GJRH01045658
cluster13895
Np2015e|GJRU01006225;Nf_ref|GHLB01005112;Nc2017|GJRH01052617

cluster13902
Np2015e|GJRU01017925;Nf_ref|GHLB01032000;Nc2017|GJRH01059830
cluster13909
Np2015e|GJRU01062397;Nf_ref|GHLB01033348;Nc2017|GJRH01004718
cluster13915
Np2015e|GJRU01033257;Nf_ref|GHLB01023917;Nc2017|GJRH01026004
cluster13916
Np2015e|GJRU01046821;Nf_ref|GHLB01043845;Nc2017|GJRH01022666
cluster13918
Np2015e|GJRU01045951;Nf_ref|GHLB01003466;Nc2017|GJRH01013527
cluster13927
Np2015e|GJRU01019752;Nf_ref|GHLB01001320;Nc2017|GJRH01022399
cluster13934
Np2015e|GJRU01007947;Nf_ref|GHLB01014172;Nc2017|GJRH01011076
cluster13937
Np2015e|GJRU01017765;Nf_ref|GHLB01025645;Nc2017|GJRH01091429
cluster13944
Np2015e|GJRU01008145;Nf_ref|GHLB01050816;Nc2017|GJRH01072945
cluster13945
Np2015e|GJRU01041826;Nf_ref|GHLB01004732;Nc2017|GJRH01072551
cluster13953
Np2015e|GJRU01002746;Nf_ref|GHLB01046759;Nc2017|GJRH01081579
cluster13954
Np2015e|GJRU01069637;Nf_ref|GHLB01011640;Nc2017|GJRH01036721
cluster13957
Np2015e|GJRU01031222;Nf_ref|GHLB01034768;Nc2017|GJRH01058784
cluster13960
Np2015e|GJRU01019648;Nf_ref|GHLB01026125;Nc2017|GJRH01061265
cluster13962
Np2015e|GJRU01053932;Nf_ref|GHLB01001668;Nc2017|GJRH01051085
cluster13963
Np2015e|GJRU01011596;Nf_ref|GHLB01045957;Nc2017|GJRH01084305
cluster13969
Np2015e|GJRU01046931;Nf_ref|GHLB01005732;Nc2017|GJRH01060691
cluster13973
Np2015e|GJRU01053915;Nf_ref|GHLB01040159;Nc2017|GJRH01089137
cluster13982
Np2015e|GJRU01057891;Nf_ref|GHLB01011087;Nc2017|GJRH01041309
cluster13984
Np2015e|GJRU01072739;Nf_ref|GHLB01001640;Nc2017|GJRH01067372
cluster13985
Np2015e|GJRU01011809;Nf_ref|GHLB01001527;Nc2017|GJRH01003648
cluster13987
Np2015e|GJRU01014589;Nf_ref|GHLB01037388;Nc2017|GJRH01038891
cluster13988
Np2015e|GJRU01041020;Nf_ref|GHLB01002242;Nc2017|GJRH01036048
cluster13995
Np2015e|GJRU01011516;Nf_ref|GHLB01004811;Nc2017|GJRH01008587
cluster13998
Np2015e|GJRU01053879;Nf_ref|GHLB01006036;Nc2017|GJRH01070860
cluster14004
Np2015e|GJRU01062324;Nf_ref|GHLB01013455;Nc2017|GJRH01065701
cluster14008
Np2015e|GJRU01005392;Nf_ref|GHLB01004468;Nc2017|GJRH01015225

```
cluster14009
      Np2015e|GJRU01031722;Nf_ref|GHLB01009588;Nc2017|GJRH01091463
cluster14011
      Np2015e|GJRU01003363;Nf_ref|GHLB01025808;Nc2017|GJRH01057412
cluster14012
      Np2015e|GJRU01034667;Nf_ref|GHLB01048002;Nc2017|GJRH01040052
cluster14021
      Np2015e|GJRU01067733;Nf_ref|GHLB01038419;Nc2017|GJRH01016722
cluster14022
      Np2015e|GJRU01056227;Nf_ref|GHLB01032685;Nc2017|GJRH01080031
cluster14026
      Np2015e|GJRU01009808;Nf_ref|GHLB01000942;Nc2017|GJRH01077690
cluster14032
      Np2015e|GJRU01016699;Nf_ref|GHLB01035858;Nc2017|GJRH01029409
cluster14037
      Np2015e|GJRU01065440;Nf_ref|GHLB01001570;Nc2017|GJRH01063783
```

Supplement ST2: NA-DEGs.

Non-annotated transcripts (predicted proteins) from 3 taxonomic coverages identified as differentially expressed in two previous studies (Ref column): 1= Roncalli et al 2022; 2=Roncalli et al 2019. NCBI accession numbers (Col. 1) and corresponding TRINITY-assigned codes from the *Neocalanus flemingeri* reference transcriptome, Nf_ref (Col. 2). These correspond to the numbers given for the "Secondary subsets" in Table 5 "#DEGs" of the main text.

MYELINATA TAXONOMIC COVERAGE

Accession	Trinity ID	Ref	Notes
GHLB01050470	TRINITY_DN14181_c0_g1_i3	1	
GHLB01016012	TRINITY_DN14993_c2_g3_i3	1	
GHLB01005350	TRINITY_DN16262_c0_g2_i1	1	
GHLB01034156	TRINITY_DN17072_c0_g1_i5	1	
GHLB01019980	TRINITY_DN17627_c0_g2_i1	1	
GHLB01037848	TRINITY_DN17750_c0_g1_i6	1	
GHLB01037604	TRINITY_DN17770_c1_g1_i1	1	
GHLB01004818	TRINITY_DN14474_c0_g1_i1	1,2	
GHLB01033879	TRINITY_DN17033_c1_g1_i4	1,2	
GHLB01030079	TRINITY_DN17856_c2_g1_i4	1,2	
GHLB01036972	TRINITY_DN11494_c0_g1_i1	2	
GHLB01027356	TRINITY_DN12211_c0_g1_i1	2	
GHLB01036221	TRINITY_DN14374_c0_g2_i1	2	
GHLB01021759	TRINITY_DN15749_c0_g2_i1	2	
GHLB01040565	TRINITY_DN16365_c0_g2_i2	2	
GHLB01019227	TRINITY_DN17240_c1_g2_i1	2	
GHLB01002484	TRINITY_DN17505_c0_g1_i5	2	
GHLB01002637	TRINITY_DN17529_c0_g1_i2	2	
GHLB01019991	TRINITY_DN17624_c1_g1_i1	2	
GHLB010318051	TRINITY_DN17910_c1_g23_i1	2	
GHLB01048443	TRINITY_DN8569_c0_g1_i1	2	Fig SF2C
GHLB01009141	TRINITY_DN9059_c0_g1_i1	2	

CALANIDAE TAXONOMIC COVERAGE

Accession	Trinity ID	Ref	Notes
GHLB01023281	TRINITY_DN10810_c0_g1_i2	1	
GHLB01036988	TRINITY_DN11462_c0_g1_i1	1	
GHLB01021116	TRINITY_DN11524_c0_g1_i2	1	
GHLB01021145	TRINITY_DN11597_c0_g1_i1	1	
GHLB01036340	TRINITY_DN11616_c0_g3_i1	1	
GHLB01015290	TRINITY_DN12035_c0_g1_i2	1	
GHLB01001136	TRINITY_DN12166_c0_g1_i2	1	
GHLB01000928	TRINITY_DN13569_c0_g1_i1	1	
GHLB01050574	TRINITY_DN14116_c0_g1_i3	1	
GHLB01043425	TRINITY_DN15085_c0_g1_i2	1	
GHLB01006732	TRINITY_DN15234_c1_g2_i3	1	
GHLB01006477	TRINITY_DN15299_c0_g1_i1	1	
GHLB01030591	TRINITY_DN15456_c1_g1_i2	1	
GHLB01042880	TRINITY_DN15643_c0_g8_i2	1	
GHLB01028285	TRINITY_DN15953_c0_g1_i2	1	
GHLB01046449	TRINITY_DN16108_c0_g1_i3	1	

GHLB01014470	TRINITY_DN16616_c0_g1_i3	1	
GHLB01019108	TRINITY_DN17211_c0_g2_i1	1	
GHLB01047490	TRINITY_DN17329_c0_g1_i1	1	
GHLB01002864	TRINITY_DN17576_c3_g1_i4	1	
GHLB01031136	TRINITY_DN17955_c1_g16_i	1	
GHLB01042536	TRINITY_DN28542_c0_g1_i1	1	
GHLB01028725	TRINITY_DN9720_c0_g1_i1	1	
GHLB01045090	TRINITY_DN10712_c0_g1_i1	1,2	
GHLB01008975	TRINITY_DN13923_c0_g1_i1	1,2	Fig. 4B
GHLB01044195	TRINITY_DN14879_c0_g1_i1	1,2	
GHLB01035484	TRINITY_DN15123_c0_g1_i5	1,2	
GHLB01016743	TRINITY_DN15856_c0_g1_i8	1,2	
GHLB01014198	TRINITY_DN16621_c0_g2_i4	1,2	
GHLB01047756	TRINITY_DN17370_c5_g4_i1	1,2	
GHLB01002854	TRINITY_DN17576_c2_g1_i2	1,2	
GHLB01015445	TRINITY_DN7048_c0_g1_i1	1,2	
GHLB01033633	TRINITY_DN8009_c0_g1_i1	1,2	Fig. 4A
GHLB01001824	TRINITY_DN10220_c0_g1_i1	2	
GHLB01037072	TRINITY_DN11469_c0_g1_i1	2	
GHLB01036383	TRINITY_DN11625_c0_g1_i1	2	
GHLB01027384	TRINITY_DN12213_c0_g1_i1	2	
GHLB01045738	TRINITY_DN13629_c0_g2_i1	2	
GHLB010016791	TRINITY_DN13882_c0_g1_i1	2	
GHLB01036204	TRINITY_DN14367_c0_g2_i2	2	
GHLB01016127	TRINITY_DN14934_c0_g1_i2	2	
GHLB01035490	TRINITY_DN15131_c0_g1_i2	2	
GHLB01021705	TRINITY_DN15724_c0_g1_i3	2	
GHLB01005406	TRINITY_DN16267_c0_g1_i1	2	
GHLB01040122	TRINITY_DN16343_c4_g4_i4	2	
GHLB01019360	TRINITY_DN17212_c1_g3_i5	2	
GHLB01007143	TRINITY_DN17444_c0_g5_i2	2	
GHLB01002498	TRINITY_DN17537_c1_g5_i1	2	
GHLB01002502	TRINITY_DN17549_c1_g1_i2	2	
GHLB01020125	TRINITY_DN17685_c0_g1_i7	2	
GHLB01037300	TRINITY_DN17712_c1_g1_i2	2	
GHLB01028589	TRINITY_DN20587_c0_g1_i1	2	
GHLB01046725	TRINITY_DN3485_c0_g1_i1	2	
GHLB01025721	TRINITY_DN4865_c0_g1_i1	2	
GHLB01012081	TRINITY_DN6441_c0_g1_i2	2	
GHLB01035085	TRINITY_DN8232_c0_g1_i1	2	
GHLB01009253	TRINITY_DN9022_c1_g1_i1	2	
GHLB01007777	TRINITY_DN9687_c1_g1_i1	2	
GHLB01031063	TRINITY_DN9865_c0_g1_i1	2	

NEOCALANUS TAXONOMIC COVERAGE

Accession	Trinity ID	Ref	Notes
GHLB01043659	TRINITY_DN10068_c0_g1_i1	1	
GHLB01049082	TRINITY_DN11168_c0_g1_i1	1	
GHLB01036380	TRINITY_DN11656_c0_g1_i1	1	
GHLB01036346	TRINITY_DN11661_c0_g1_i1	1	
GHLB01015300	TRINITY_DN12037_c0_g1_i1	1	
GHLB01004964	TRINITY_DN13156_c0_g1_i2	1	
GHLB01004936	TRINITY_DN13178_c0_g1_i3	1	
GHLB01009061	TRINITY_DN13978_c0_g1_i4	1	
GHLB01036028	TRINITY_DN14378_c0_g1_i1	1	
GHLB01004729	TRINITY_DN14477_c0_g1_i2	1	
GHLB01022819	TRINITY_DN15528_c0_g1_i4	1	
GHLB01016736	TRINITY_DN15856_c0_g1_i1	1	
GHLB01003454	TRINITY_DN16034_c0_g7_i2	1	
GHLB01046124	TRINITY_DN16150_c2_g1_i1	1	
GHLB01040251	TRINITY_DN16302_c0_g1_i3	1	
GHLB01040295	TRINITY_DN16318_c0_g1_i1	1	
GHLB01051090	TRINITY_DN16482_c0_g1_i3	1	
GHLB01010156	TRINITY_DN16824_c2_g3_i1	1	
GHLB01010338	TRINITY_DN16885_c1_g5_i2	1	
GHLB01019064	TRINITY_DN17293_c2_g2_i1	1	
GHLB01030060	TRINITY_DN17826_c1_g1_i1	1	
GHLB01009970	TRINITY_DN3616_c0_g1_i1	1	
GHLB01015513	TRINITY_DN7089_c0_g1_i1	1	
GHLB01042433	TRINITY_DN10458_c0_g2_i1	1,2	
GHLB01045957	TRINITY_DN10502_c0_g1_i1	1,2	
GHLB01049043	TRINITY_DN11155_c0_g1_i2	1,2	
GHLB01041343	TRINITY_DN1214_c0_g1_i1	1,2	
GHLB01001527	TRINITY_DN13861_c0_g1_i5	1,2	
GHLB01041718	TRINITY_DN14094_c0_g1_i3	1,2	
GHLB010047321	TRINITY_DN14404_c0_g1_i1	1,2	
GHLB01035467	TRINITY_DN15108_c1_g1_i2	1,2	Fig SF2B
GHLB01011933	TRINITY_DN15359_c0_g1_i5	1,2	
GHLB01028031	TRINITY_DN15921_c0_g1_i1	1,2	Fig. 4C
GHLB01051436	TRINITY_DN16457_c0_g1_i1	1,2	
GHLB01018689	TRINITY_DN16566_c1_g1_i2	1,2	
GHLB01014663	TRINITY_DN16626_c0_g1_i4	1,2	
GHLB01019901	TRINITY_DN17687_c1_g2_i1	1,2	
GHLB01008164	TRINITY_DN18692_c0_g1_i1	1,2	Fig. SF2A
GHLB01013771	TRINITY_DN3979_c0_g1_i2	1,2	
GHLB01000117	TRINITY_DN866_c0_g1_i1	1,2	
GHLB01013027	TRINITY_DN10658_c0_g1_i2	2	
GHLB01023152	TRINITY_DN10836_c0_g1_i1	2	
GHLB01005880	TRINITY_DN11053_c0_g1_i1	2	
GHLB01041379	TRINITY_DN1239_c0_g1_i1	2	
GHLB01012721	TRINITY_DN12403_c0_g1_i1	2	
GHLB01026125	TRINITY_DN12701_c0_g1_i1	2	
GHLB01001570	TRINITY_DN13816_c0_g1_i1	2	
GHLB01001640	TRINITY_DN13835_c0_g1_i1	2	

GHLB01044460	TRINITY_DN14809_c0_g1_i2	2
GHLB01044514	TRINITY_DN14861_c0_g1_i6	2
GHLB01011640	TRINITY_DN15366_c0_g1_i2	2
GHLB01030656	TRINITY_DN15416_c0_g1_i2	2
GHLB01016700	TRINITY_DN15824_c1_g4_i1	2
GHLB01028249	TRINITY_DN15956_c0_g1_i7	2
GHLB01046298	TRINITY_DN16199_c0_g2_i2	2
GHLB01005492	TRINITY_DN16234_c1_g1_i5	2
GHLB01005320	TRINITY_DN16258_c0_g1_i3	2
GHLB01018394	TRINITY_DN16526_c1_g2_i3	2
GHLB01018511	TRINITY_DN16537_c0_g1_i1	2
GHLB01018518	TRINITY_DN16537_c0_g2_i1	2
GHLB01039499	TRINITY_DN16918_c0_g2_i3	2
GHLB01002734	TRINITY_DN17571_c0_g2_i3	2
GHLB01002166	TRINITY_DN17584_c0_g2_i1	2
GHLB01019786	TRINITY_DN17636_c1_g1_i2	2
GHLB01029509	TRINITY_DN17843_c0_g1_i3	2
GHLB01026879	TRINITY_DN18775_c0_g1_i1	2
GHLB01003591	TRINITY_DN2248_c0_g1_i1	2
GHLB01050816	TRINITY_DN26199_c0_g1_i1	2
GHLB01032000	TRINITY_DN27360_c0_g1_i1	2
GHLB01001320	TRINITY_DN6542_c0_g1_i1	2
GHLB01044826	TRINITY_DN7179_c0_g1_i1	2
GHLB01048159	TRINITY_DN7412_c0_g1_i1	2
GHLB01009588	TRINITY_DN7654_c0_g1_i1	2
